# Regioselective [3 + 2] cycloaddition reactions of the phosphorus and arsenic analogues of the thiocyanate anion

**DOI:** 10.1039/d6sc01985d

**Published:** 2026-04-24

**Authors:** Marc Baltrun, David Hanneberg, Florian Hett, Michael Seidl, Florian Weigend, Stephan Hohloch

**Affiliations:** a Department for General, Inorganic and Theoretical Chemistry, University of Innsbruck Innrain 80 – 82 6020 Innsbruck Austria Stephan.Hohloch@uibk.ac.at; b Institute for Quantum Materials and Technologies (IQMT), Karlsruhe Institute of Technology (KIT) Kaiserstr. 12 76131 Karlsruhe Germany Florian.Weigend@kit.edu; c Fachbereich Chemie und Wissenschaftliches Zentrum für Materialwissenschaften (WZMW), Philipps-Universität Mar-burg Hans-Meerwein-Straße 4 35043 Marburg Germany

## Abstract

Salt metathesis reactions of tris-amide zirconium iodide complex ([NRR′]_3_ZrI (1-I) (with NRR′ = 3,5-Xylyl-*tert*-butylamide, N(Xyl)(^*t*^Bu)) and sodium 2-phosphaethynthiolate (Na(diox)_3_SCP) or 2-arsaethynthiolate (Na(diox)_3_SCAs) in THF result in a [3 + 2] cycloaddition of two SCE (E = P, As) units forming five membered 2-thio-1,3,4-thiadiphosphole and 2-thio-1,3,4-thiadiarsole heterocycles with an exocyclic sulfur atom, bridging two zirconium fragments (general formula (N(RR′)_3_Zr(κ-C,S-(SCE)_2_-Zr(NRR′)_3_ with E = P (2-PP) or As (3-AsAs)). The reactions are regioselective and only the P,P/As,As isomers are formed in THF and quantum chemical investigations suggest a concerted ring formation in line with a [3 + 2] cycloaddition reaction. Switching the solvent to toluene, salt metathesis with NaSCP results in the selective formation of the second possible [3 + 2] cycloaddition regioisomer 2-SP, with a 3-thio-1,2,4-thiadiphosphole bridge, while for NaSCAs both regioisomers with an As–As bond (3-AsAs) and an S–As bond (3-SAs; 3-thio-1,2,4-thiadiarsole bridge) are observed. Attempts to obtain the free diphosphole/diarsole rings using methyl triflate resulted in the cleavae of only one zirconium center, giving access to the triflate complex 1-OTf and the mono-metallated diphospholes/diarsoles 4-PP, 4-PS and 5-AsAs respectively.

## Abstract 2

The regioselective zirconium induced [3 + 2] cycloadditon of two [SCP]^–^ or [SCAs]^–^ anions leading to 2-thio-1,3,4-thiadipnictoles or 3-thio-1,2,4-thiadipnictoles is reported.

## Graphical abstract

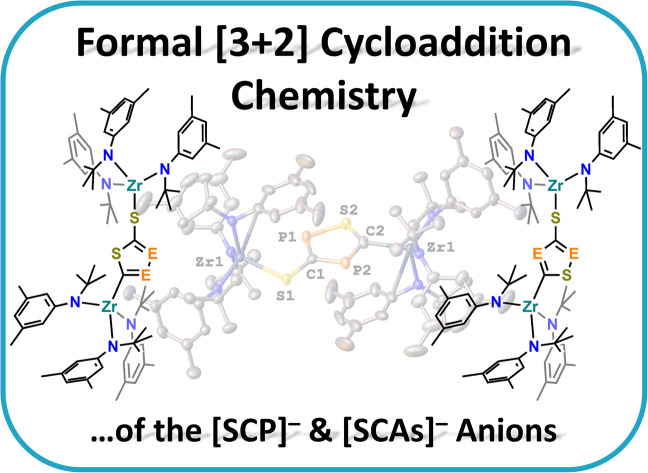

## Introduction

**Fig. 1:**
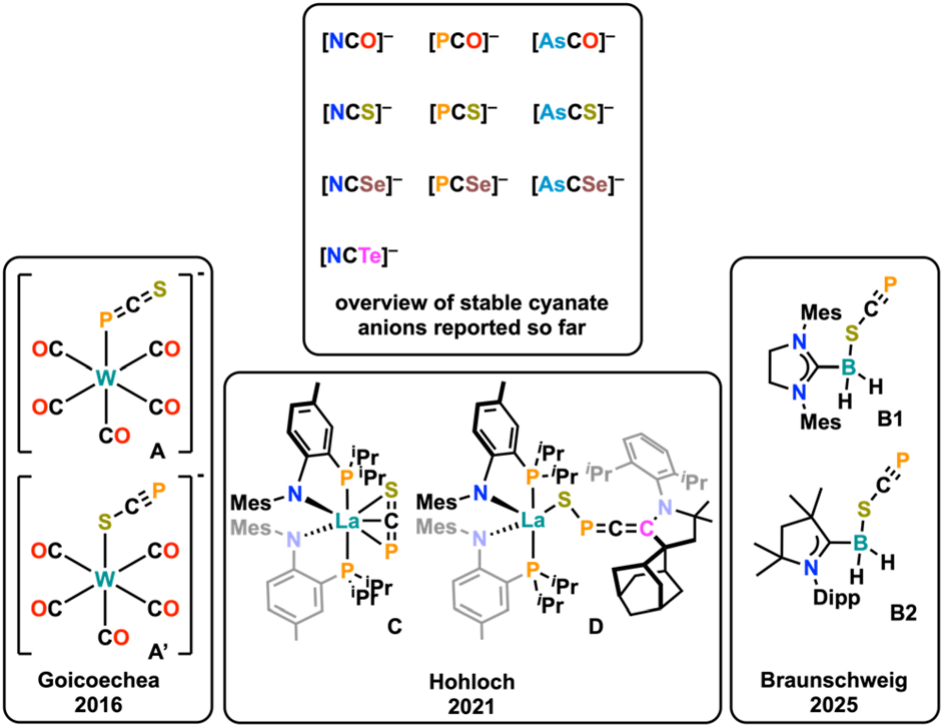
Matrix of known [ChCPn]^−^ (heavy) cyanate anions with Ch = chalcogen atom, O, S, Se, Te and Pn = Pnictogen atom, N, P, As) (top). Examples of phosphaethynthiolate complexes reported (bottom).

The chemistry of cyanates has unarguably led to the development of a plethora of milestones in chemical history, creating a large variety of textbook examples.^1,2^ For instance, cyanates were used to introduce the concepts of constitution isomerism by Liebig and Gay-Lussac on silver cyanate (AgOCN) *vs.* silver fulminate (AgONC),^3^ the theory of ambiphilic ligands using thiocyanates [SCN]^−^^4^ and Wöhler's connection between inorganic and organic chemistry by the synthesis of urea from ammonium cyanate.^5^ Since the early days of cyanate anions, chemists endeavored the exchange of either oxygen or nitrogen in the [OCN]^−^ anion by heavier analogues of the chalcogen or pnictide series, which led among others to the development of the selenocyanate [SeCN]^−^^6^ and the tellurocyanate [TeCN]^−^ anions.^7^ Turning to pnictogen atom exchange, one milestone was the synthesis of the phosphaethynolate anion [OCP]^−^. Its synthesis being already indicated in 1894 (but falsely identified as Na(CP)),^8^ Becker and co-workers were the first to describe its targeted synthesis in the form of [Li(DME)_2_][OCP].^9^ Shortly after, Westerhausen *et al.* synthesized the bis-[OCP]^−^ salts of the alkaline earth metals Mg, Ca, Sr, and Ba with the general formula [M(DME)_2_](OCP)_2_] (M = Alkaline Earth Metal).^10^ However, these salts were not stable^9,10^ and it took another decade until Grützmacher^11^ and Goicoechea^12^ introduced the stable sodium and potassium salts, which led to a renaissance of heavy cyanate chemistry and a flourishing (phosphorus) chemistry was introduced around this molecule in the past decade.^13–16^ Soon after, Goicoechea and Hinz also introduced the arsenic congener [OCAs]^−^^17^ which has also been widely used in (coordination) chemistry.^2^ Finally, in 2018, Goicoechea, Tambornino and Hinz introduced a general method to also synthesize the heaviest cyanates known so far by introducing sulfur or selenium,^18^ resulting in the synthesis of the phosphaethynthiolate [SCP]^−^^19^ (also originally synthesized by Becker as its Li(DME)_3_ salt),^20^ the arsaethynthiolate [SCAs]^−^, the phosphaethynselenolate [SeCP]^−^ and the arsaethynselenolate [SeCAs]^−^ anions (Fig. 1, top). Contrasting the chemistry of their oxygen congeners ([OCP]^−^ and [OCAs]^−^),^2,14,15^ almost no coordination chemistry of these anions has been described so far. In fact, except for one example from our group (Fig. 1, bottom, *vide infra*), only two other examples have been reported.^19,21^ The first example of [SCP]^−^ coordination was mentioned by Goicoechea and co-workers,^19^ describing its (unselective) coordination towards tungsten, giving the coordination isomers A and A′, which cannot be separated. The second example was reported only recently by Braunschweig and co-workers,^21^ showing the stable κ^1^-S coordination of the [SCP]^−^ anion towards a NHC/CAAC stabilized borane B. Notably, for the even heavier [SCAs]^−^ anion, no examples on any reactivity or coordination has been reported yet. However, theoretical calculations have shown that these anions should exhibit prominent reactivity in a variety of cycloaddition reactions.^22^

We have recently synthesized the first example of a stable lanthanide complex of the [SCP]^−^ anion (Complex C, Fig. 1),^23^ using a bis-PN chelated lanthanide center.^24^ Therein, the [SCP]^−^ anion was neither found to adopt a κ^1^-S nor a κ^1^-P end-on coordination mode, but instead an unprecedented *η*^3^-side-on coordination mode was found.^23^ The complex was indefinitely stable and reacted with cyclic alkyl amino carbenes (CAACs) under the formation of a CAAC-stabilized heavy fulminate-type anion in complex D (Fig. 1), which stated the first defined reactivity of a heavier homologue of the thiocyanate anion. Inspired by these results we further aimed to explore the coordination motifs and chemistry of the phosphaethynthiolate anion ([SCP]^−^) as well as its arsenic analogue, the arsaethynthiolate anion ([SCAs]^−^) towards early transition metals, namely zirconium.^18^ Here, we report the unprecedented and regioselective [3 + 2] cycloaddition reaction between two heavy cyanate anions, giving access to thiadiphosphole and thiadiarsole bridging ligands, as well as the attempts to isolate their “free” form through cleavage from the zirconium centers using methyl triflate.

## Results and discussion

**Scheme 1:**
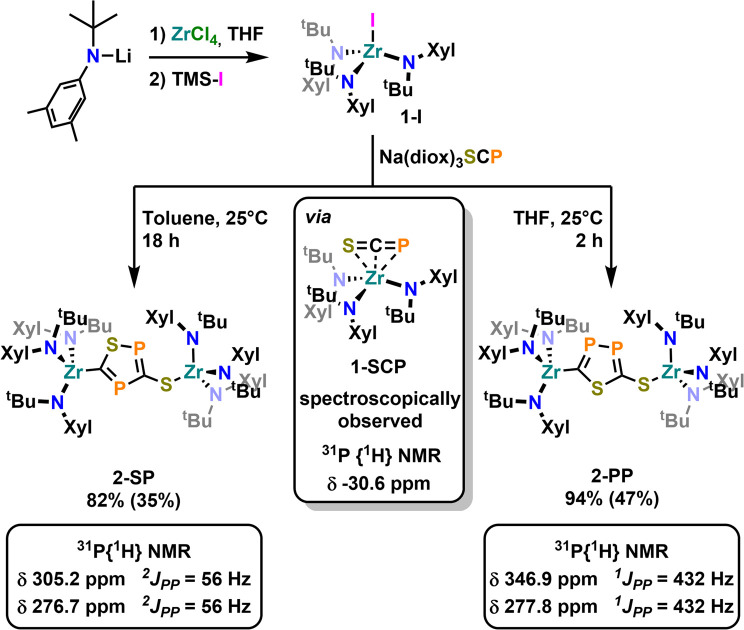
Synthesis of the iodide complex 1-I acting as a starting material for salt metathesis reaction in this manuscript, along with the regioselective cyclisation reactivity observed in its salt metathesis reaction with Na(diox)_3_SCP leading to the two heterocycles 2-SP and 2-PP. Yields in brackets indicate crystalline yields.

**Scheme 2:**
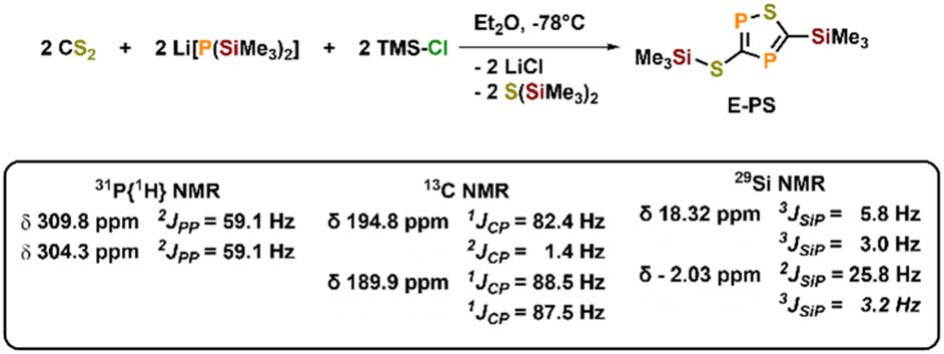
Synthesis of a silylated 3-thio-1,2,4-thiadiphosphole E-PS reported by Appel and Moors in 1986.

To further examine the chemistry of the heavy thiocyanates we synthesized the tetra-coordinated zirconium amide complex (NRR′)_3_ZrI (1-I) (R = ^*t*^Bu, R′ = Xylyl), supported by the amide ligand 3,5-Xylyl-*tert*-butyl-amide.^25^ The complex can be synthesized in two steps, from the reaction of the lithium amide with ZrCl_4_ and subsequent exchange of the remaining chloride ligand by iodide using trimethylsilyl iodide (yield 60%, Scheme 1). Salt metathesis of the iodo complex 1-I with Na(diox)_3_SCP in toluene resulted in the initial formation of a new species showing a ^31^P NMR resonance at −30.6 ppm. Given the similarity of this value to complex C (−44.9 ppm),^23^ we attribute this resonance to the SCP complex 1-SCP (Scheme 1). Despite numerous attempts we have not been able to grow any crystals of this species, since it only exists “transiently” and reacts to a new compound 2-SP within several minutes, showing two ^31^P{^1^H} NMR resonances at intense low-field shifts of 305.2 and 276.7 ppm with a ^2^*J*_PP_ coupling of 56 Hz (Fig. S32). In addition, the ^1^H NMR (Fig. S30) reveals two signal sets for the ligand, indicating the formation of a dimeric species in solution. This is further corroborated by the observation of two distinct ^13^C{^1^H} NMR resonances at 235.6 (doublet, ^1^*J*_CP_ = 103.3 Hz) and 199.4 ppm (doublet of doublets, ^1^*J*_CP_ = 86.1 Hz; ^1^*J*_CP_ = 97.5 Hz; Fig. S31). Comparison of these NMR signals to the literature showed, that these are in the similar range of a 5-(trimethylsilyl)-3-((trimethylsilyl)thio)-1,2,4-thiadiphosphole E-PS (Scheme 2)^26^ originally synthesized by Appel and Moors starting from CS_2_, Li[P(SiMe_3_)_2_] and TMS-Cl. Furthermore, calculations on the ^31^P NMR shifts (exact two-component decoupling (X2C) including spin–orbit coupling,^27^ see SI for further information on calculations) of such a putative 3-thio-1,2,4-thiadiphosphole gave values of 300 and 293 ppm, and a ^2^*J*_PP_ coupling constant of 55 Hz, which agrees well with the observed coupling constant of 56 Hz (*vide supra*).

**Fig. 2:**
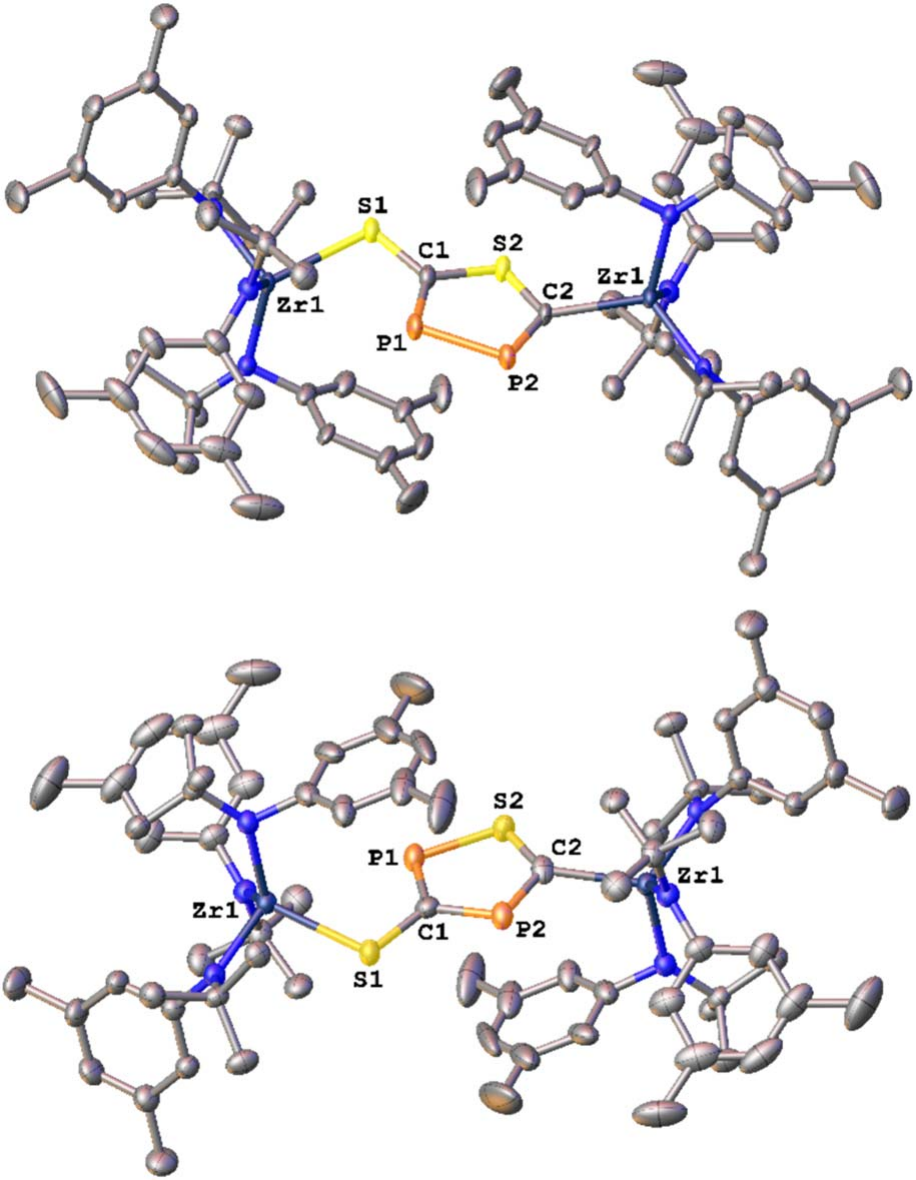
Molecular structures of the two possible regioisomers 2-PP (top) and 2-SP (bottom) observed after the salt metathesis reaction between 1-I and Na(diox)_3_SCP in THF or toluene respectively. Hydrogen atoms are omitted for clarity, all ellipsoids are shown at a probability level of 50%.

The formation of the 3-thio-1,2,4-thiadiphosphole heterocycle was confirmed by X-ray diffraction analysis of single crystals of 2-SP grown by slow evaporation of a diethyl ether solution at room temperature (Fig. 2, bottom). The analysis revealed the presence of a five-membered heterocycle with an *exo*-cyclic sulfur atom, which most likely forms *via* an autogenic [3 + 2] cycloaddition reaction of a heavy cyanate. The cycle bridges two zirconium centers in an κ^1^-S and a κ^1^-C bound fashion with Zr1–S1 and Zr1–C2 distances of 2.554(1) and 2.297(8) Å respectively. The latter is in the range of typical Zr–C_aryl_/Zr–C_alkyl_ distances,^28^ which is in line with a carbanionic donor atom. As indicated by ^31^P{^1^H} NMR spectroscopy, the two phosphorus atoms are separated by a carbon atom (C1), which results in the observed ^2^*J*_PP_ coupling of 56 Hz (*vide supra*). The phosphor sulfur P1–S2 distance is 2.074(5) Å, which is in between a P–S single and double bond (P–S = 2.10–2.31 Å;^29,30^ P=S = 1.95–2.03 Å,^31^ indicating electronic delocalization within the cycle. This is further supported by the calculation of localized^32^ molecular orbitals (LMOs, Fig. S90, for details of the calculation see SI): they reveal five σ-type orbitals representing the single bonds in the ring, and additionally three π-type orbitals. Two of them represent π-bonds mainly between one P and the neighbored C atom, but with a certain delocalization to the second P atom. The third one represents the free electron pair at S, but again somewhat delocalized to the neighbors. Overall, this suggests “close to double” P–C bonds and “more than single” for the other bonds in the ring. The C–S bond to the exocyclic sulfur atom was found to be 1.752(8) Å, which is representative for a C–S single bond,^29,33^

**Fig. 3:**
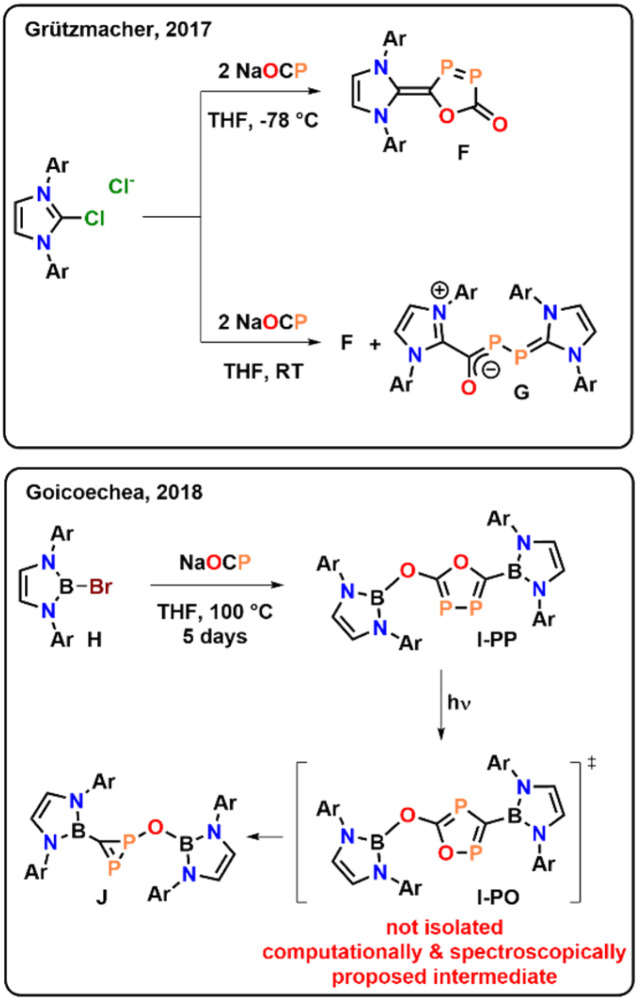
Previously reported couplings of the phosphaethynolate anion [OCP]^−^ mediate by NHCs or boranes (Ar = 2,6-diisopropylphenyl).

It should be noted at this point of the discussion, that Grützmacher^34^ and Goicoechea,^35^ have previously reported related cyclization reactions of the phosphaethynthiolate anion [OCP]^−^. However, contrasting the present case, neither of them led to thermally (or photo-) stable cyclic compounds and both examples decompose within days. Grützmacher reports the NHC-mediated coupling of two phosphaethynolate anions to obtain compound F (Fig. 3), which however can only be selectively obtained if the reaction is performed at very low-temperature (−78 °C) and decomposes within a few days.^34^ If the reaction is performed at room temperature, the zwitter ionic species G is observed as a main product. Turning to Goicoechea's examples,^35^ the group reported the synthesis of the bromoborane H and its thermal conversion (100 °C) in the presence of Na[OCP] to I-PP within 5 days, in which new P–P bond is formed (Fig. 3). However, I-PP was found to be highly light sensitive and decomposes under concomitant CO elimination to the new diphosphirene J. The authors spectroscopically see an intermediate in this transformation, which they computationally assign as the photo-isomerized product I-OP. Nevertheless, as noted, the authors were not able to structurally characterize and unambiguously validate this intermediate and its instability towards light preluded its isolation.

Inspired by the fact that in related [OCP]^−^ cyclization reactions a P–P heterocycle is postulated as a transitional product, we questioned if a selective synthesis of the second regioisomer of the autogenic [3 + 2] cycloaddition reaction with a 2-thio-1,3,4-thiadiphosphole bridge (2-PP) instead to the observed 3-thio-1,2,4-thiadiphosphole bridge in 2-SP is possible. A cyclisation to the putative regioisomer 2-PP would also be highly beneficial as the synthesis of related thia-1,2-diphospholes has so far not been accomplished selectively.^36^ Targeting the synthesis of this species 2-PP, we found that if the salt metathesis reaction between 1-I and Na(diox)_3_SCP is performed in THF instead of toluene, the reaction almost instantly gives access to a new product (Scheme 1). Notably, the ^31^P{^1^H} NMR shows the clean formation of a new product showing distinct low-field shifts at 346.8 and 276.8 ppm with a ^1^*J*_PP_ coupling of 432 Hz (Fig. S25), which is strongly indicative of the successful formation of 2-PP. Quantum chemical calculations of a putative 2-thio-1,3,4-thiadi-phosphole proposed ^31^P NMR shifts of 361 and 280 ppm, with a ^1^*J*_PP_ coupling constant of 422 Hz, well-fitting the observed values (*vide supra*; See SI for further information on NMR data calculations). Similar to the ^1^H NMR of 2-SP, the ^1^H NMR of the reaction in THF (Fig. S23) shows the presence of two signal sets for the ligand, further supporting the formation of a dimeric species in solution. Furthermore, the ^13^C NMR spectrum of 2-PP shows a doublet at 227.8 (^1^*J*_CP_ = 117.2 Hz) and a doublet of doublets at 195.5 ppm (^1^*J*_CP_ = 93.3 Hz; ^2^*J*_CP_ = 6.7 Hz; Fig. S24). Ultimate proof for the formation of the regioisomer 2-PP was delivered by X-ray diffraction analysis on single crystals grown from slow evaporation of a diethyl ether solution at room temperature (Fig. 2, top). This revealed the formation of a new P–P bond showing a distance of 2.123(3) Å, which is in between a P–P single and double bond (P–P = 2.23–2.26 Å,^37^ P=P = 2.03–2.05 Å^38^) also indicating an electronic delocalization in the heterocycle (compare P–P in cyclo-P_5_ ligands: 2.11 Å in (P_5_)FeCp,^39^ 2.14–2.16 Å in [(P_5_)_2_Ti]^2-^^40^ and 2.12–2.13 Å substituted [cylco-C_2_^R^P_3_]^−^ ligands^41^). Beyond this, the other bonding parameters are comparable to the regioisomer 2-SP and can be found in the SI (Tables S1 and S2). Notably all attempts to convert 2-SP into 2-PP (or *vice versa*) have failed and once formed, the two heterocycles are thermally stable up to at least 100 °C in solution over days.

**Scheme 3:**
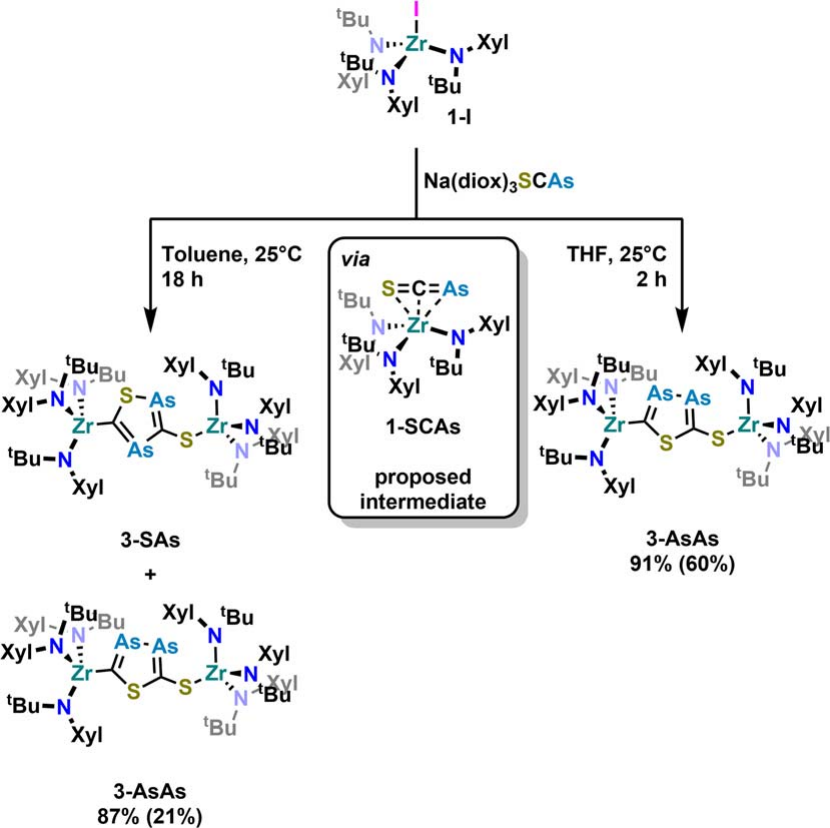
Salt metathesis reaction between 1-I and Na(diox)_3_SCAs and subsequent cyclisation of the [SCAs]^−^ anion to form a new arsenic containing heterocycle. While the reaction is regioselective in THF leading to 3-AsAs, inseparable mixtures of both isomers 3-AsAs and 3-SAs (mixed yield: 87%, ratio 3-AsAs : 3SAs = 53 : 47) are observed in toluene. Yields in brackets indicate crystalline yields.

**Fig. 4:**
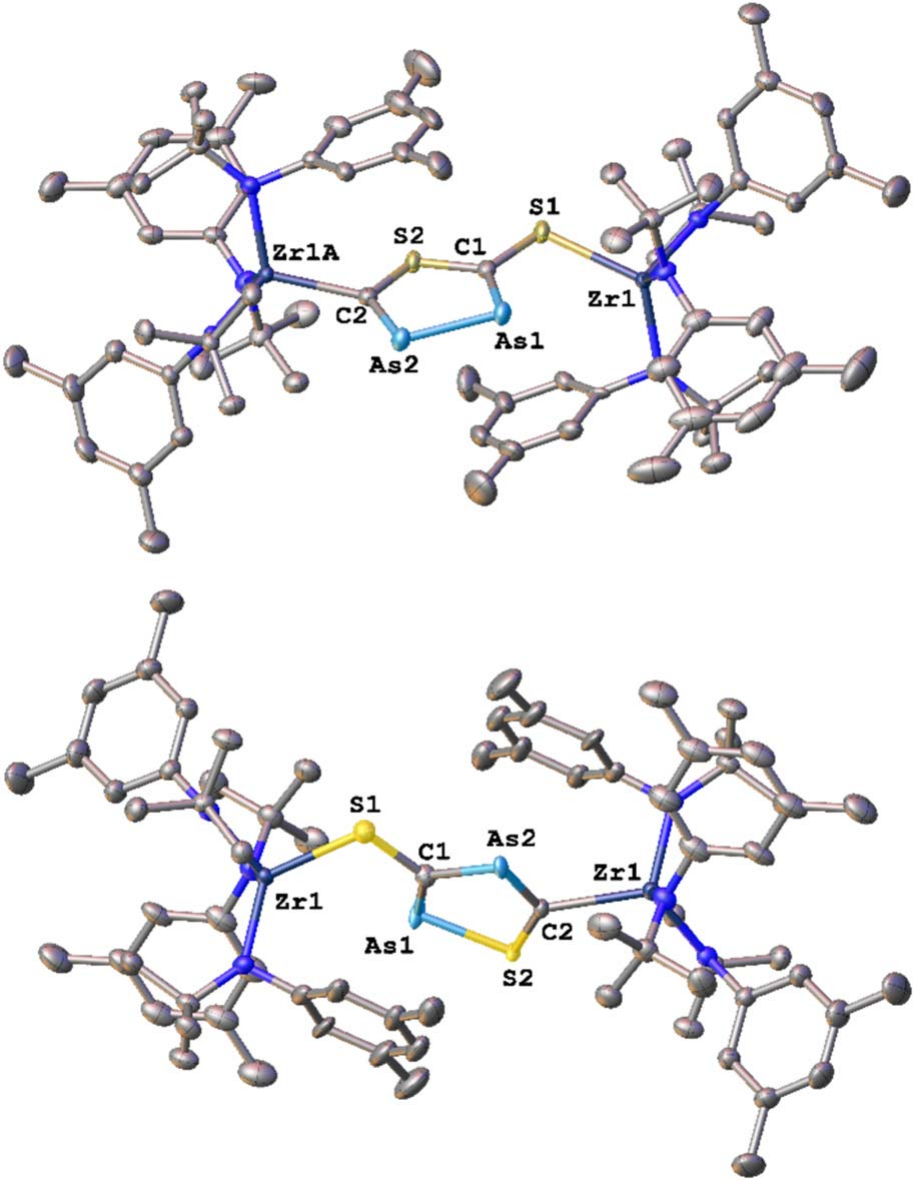
Molecular structures of the two possible [3 + 2] cycloaddition regioisomers 3-AsAs (top) and 3-SAs (bottom) observed after the salt metathesis reaction between 1-I and Na(diox)_3_SCAs in THF or toluene, respectively. Please note that the structure of 3-SAs was obtained from a crystalline mixture of 3-AsAs and 3-SAs, in which the positions of the respective heterocycles superimpose each other (ration in crystals 60 : 40). Hydrogen atoms are omitted for clarity, all ellipsoids are shown at a probability level of 50%.

**Fig. 5:**
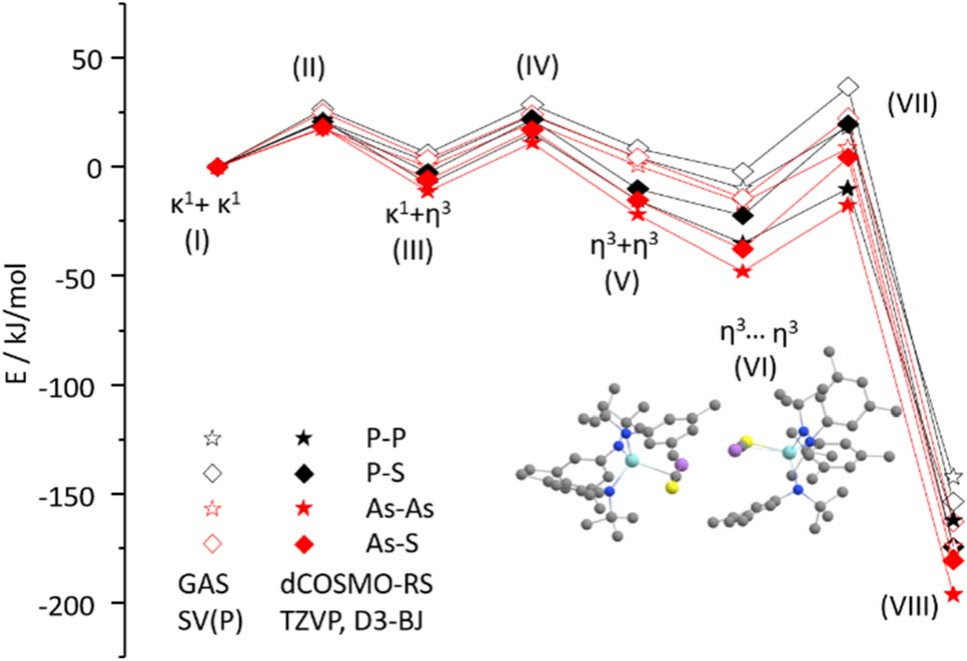
Energy profile for the reaction starting from two monomers with SCP/SCAs in κ^1^-S coordination *via η*^3^ coordination and ring closure to compounds 2-PP, 2-SP, 3-AsAs or 3-SAs at PBE level. Open symbols refer to gas phase calculations with the SV(P) basis, filled symbols to such with the TZVP basis with additional employment of the dCOSMO-RS model with the *σ*-profile for THF at 25 °C and the D3-BJ dispersion correction. The plotted structure is that of state VI, where the two monomers form a (weakly bound) dimer.

With these results in hand, we wanted to investigate whether this reactivity translates to even heavier cyanates. Thus, we synthesized the arsaethynthiolate anion [SCAs]^−^ in the form of Na(diox)_3_SCAs.^18^ To our delight, if the reaction between 1-I and Na(diox)_3_SCAs is performed in THF (Scheme 3) the formation of a clean single product is observed by ^1^H NMR spectroscopy (Fig. S37). The ^13^C{^1^H} NMR resonances were found at 241.3 and 206.1 ppm respectively (Fig. S38). X-ray diffraction analysis performed on single crystals grown from a concentrated hexane solution at room temperature, revealed solely the formation of the As–As isomer 3-AsAs showing an As–As distance of 2.3353(11) Å (Fig. 4). Similar to 2-PP, the As–As distance lies in between an As–As single and double bond (As–As = 2.42–2.48 Å;^42^ As=As = 2.26–2.31 Å).^43,44^ Similarly, the C–As bonds C1–As1 (1.862(10) Å) and C2–As2 (1.839(10) Å) are in between a single and a double bond (C–As = 1.98–2.07 Å;^43,45^ C=As = 1.81–1.88 Å;^46^ arsabenzene derivatives: 1.83–1.95 Å;^47^ arsa-cyclopentadienyl ligands 1.89–1.90 Å).^48^ Calculated LMOs (Fig. S90) and their atomic contributions are very similar to that of the corresponding P compounds and thus confirm these findings. The Zr1–S1 and the Zr1–C1 distance are found to be 2.570(2) and 2.294(8) Å and are comparable to 2-SP and 2-PP. With this result in hand, the final question was, if we could also selectively form the regioisomer 3-SAs. Unfortunately, if the salt metathesis reaction between 1-I and Na(diox)_3_SCAs is performed in toluene (which yielded the selective formation of 2-SP) a mixture of two different products is observed, in a ratio of 53 : 47 (3-AsAs : 3-SAs) by NMR spectroscopy (Scheme 3; Fig. S33 and S34). The major species could thereby be identified as 3-AsAs, wherefore we attribute the second species to the potential regioisomers 3-SAs. Carbon NMR analysis revealed the signals assigned for 3-AsAs (*vide supra*), and two additional singlets at 263.4 and 229.9 ppm, which we attribute to the 3-SAs regioisomer (Fig. S45). X-ray diffraction analysis of single crystals grown form diethyl ether further confirmed the presence of 3-AsAs in the crystal, along with the presence of the desired regioisomer 3-SAs (Fig. 4). Given the strong restraints, a discussion of the bond metrics in 3-SAs is not possible (see SI, Crystallographic Information for further information). Yet the model itself is reliable and the formation of the 3-thio-1,2,4-thiadiarsole heterocycle in 3-SAs can be unambiguously determined. To further elucidate the mechanism for the formation, we investigated the reaction from two separate κ^1^-S coordinated monomers to 2 and 3 for P and As by computational methods. Pathways for the ring formations were calculated without accounting for dispersion or environment effects at the economic dhf-SV(P)^49^/PBE^50^ level. For the identified stationary points additional single-point calculations were done with the larger dhf-TZVP basis^49^ and further employing the conductor-like screening model d-COSMO-RS^51^ employing the solvent-specific response function for THF at 25 °C (which accounts for the solvent's polarity) and/or employing the D3-BJ dispersion correction.^52^ Details on the computations may be found in the SI, as well as images of all stationary points (Fig. S91) and their energies for the different ways of calculation (Table S3), which are graphically shown in Fig. 5. Moreover, Cartesian coordinates are available in the file structures.txt, movies of the pathways in the files reaction_as-as.gif and reaction_as-s.gif.

As evident from Fig. 5, three transition states are passed, the first two (II, IV) for the rearrangement of each monomer from the κ^1^-S coordination to the *η*^3^ coordination, the third (VII) during the transformation of the two re-arranged monomers to the final compound. Without D3-BJ and dCOSMO-RS (open symbols in Fig. 5), the two different monomers are of almost the same energy and connected by low barriers (∼20 kJ mol^−1^).

The energy gain when approaching each other is small (energy difference between V and VI is ∼10 kJ mol^−1^) and the height of the third barrier is comparable to that of the first two. The energy gain of the reaction comes from the ring closure and amounts to 143 to 172 kJ mol^−1^ for the entire reaction, with the sequence As–As > As–S > S–P > P–P. Consideration of dispersion and environment produces rather subtle differences. First, the energies for the entire reaction are larger by ∼20 kJ mol^−1^, mainly due to the dispersion correction which increases the energy gain upon monomer approximation and leads to a slight preference of the *η*^3^ coordinating isomer over the κ^1^-S coordinating one. This means that rather state V than state I will be the starting point for the reactions, for which we get energy gains (energy difference between V and VIII) of 150 to 175 kJ mol^−1^, with the same sequence as before. This suggests that for thermodynamic reasons 3-AsAs is the (slightly) preferred product when using [SCAs]^−^, but 2-SP when using [SCP]^−^. The barrier heights are very similar to those obtained without employing D3-BJ and dCOSMO-RS. We may summarize that the three barriers for the ring formation in all cases can be easily surmounted under ambient conditions. Employing only D3-BJ but no dCOSMO-RS somewhat enhances the stabilization of states V to VIII but does not lead to qualitative changes; in passing we note that the simple COSMO model employed with a dielectric constant of 7.6 (THF) yields numbers between dCOSMO-RS and no COSMO, see Table S3. So, the calculations do not fully answer the question of the observed regioselectivity in different solvents. Given the fact that the calculations suggest 2-SP and 3-AsAs as the thermodynamic products of the reaction, we anticipate that regioisomer formation is largely controlled by kinetic effects which exponentially depend on the barrier height, and thus in a way that is too sensitive to be described with the comparably rough conductor-like screening model for solvation.

**Fig. 6:**
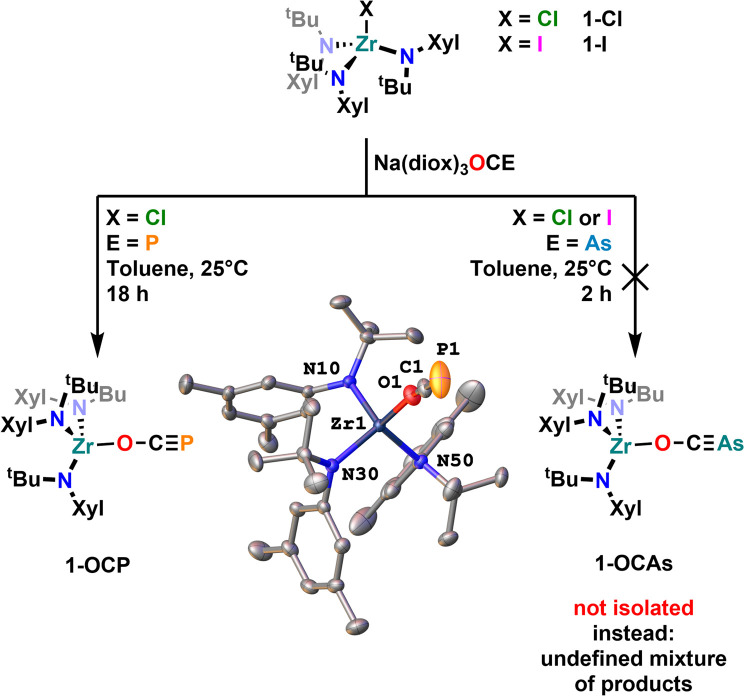
(Attempted) synthesis of the phospha- and arsaethynolate complexes 1-OCP and 1-OCAs and the molecular structure of 1-OCP. Hydrogen atoms have been omitted for clarity, ellipsoids are shown at a probability level of 50%.

To set all these results into relation to their lighter homologues, we also investigated the salt metathesis reaction between 1-Cl or 1-I and Na(diox)_3_OCP or Na(diox)_3_OCAs. This revealed unexpected and severe differences between the [OCP]^−^ and the [OCAs]^−^ anions as well as to the thio-analogues [SCP]^−^ and [SCAs]^−^. The reaction of Na(diox)_3_OCP with the halide complex 1-Cl gave clean access to the desired phosphaethynolate complex 1-OCP (Fig. 6). However, attempts to synthesize 1-OCAs*via* similar salt metathesis reactions resulted in the formation of wild mixtures (Fig. S22), from which so far no single product could be isolated. Investigations of the thermal stability of the complex 1-OCP further showed that even after prolonged time at 100 °C no degradation/further reaction is induced (Fig. S20 and S21), which contrasts Goicoechea's report on boron OCP compounds (*vide supra*, Fig. 3).^35^ These results furthermore clearly show that the reactivity of the lighter oxygen congeners [OCP]^−^ and [OCAs]^−^ is drastically different compared to their sulfur analogues [SCP]^−^ and [SCAs]^−^. Formation of the defined complex 1-OCP is indicated by ^1^H NMR spectroscopy and its spectrum shows only one set of signals (Fig. S13) indicating the formation of a κ^1^-O coordinated product.^13,53^ Furthermore, the ^31^P phosphorus resonance was found at −323.6 ppm (Fig. S15) which is indicative of a κ^1^-O coordination.^13,53^ Unambiguous proof for the formation of the phosphaethynolate complex 1-OCP was finally given by X-ray diffraction analysis on a crystal grown from a concentrated pentane solution. The oxygen-zirconium distance was found at 2.0590(13) Å, and the Zr1–O1–C1 angle is 158.23(14)°. The C–P bond was found at 1.548(2) Å. All these parameters (NMR and structure) are in well agreement with previously reported κ^1^-O coordinated phosphaethynolate complexes of the early transition metals.^13^

**Scheme 4:**
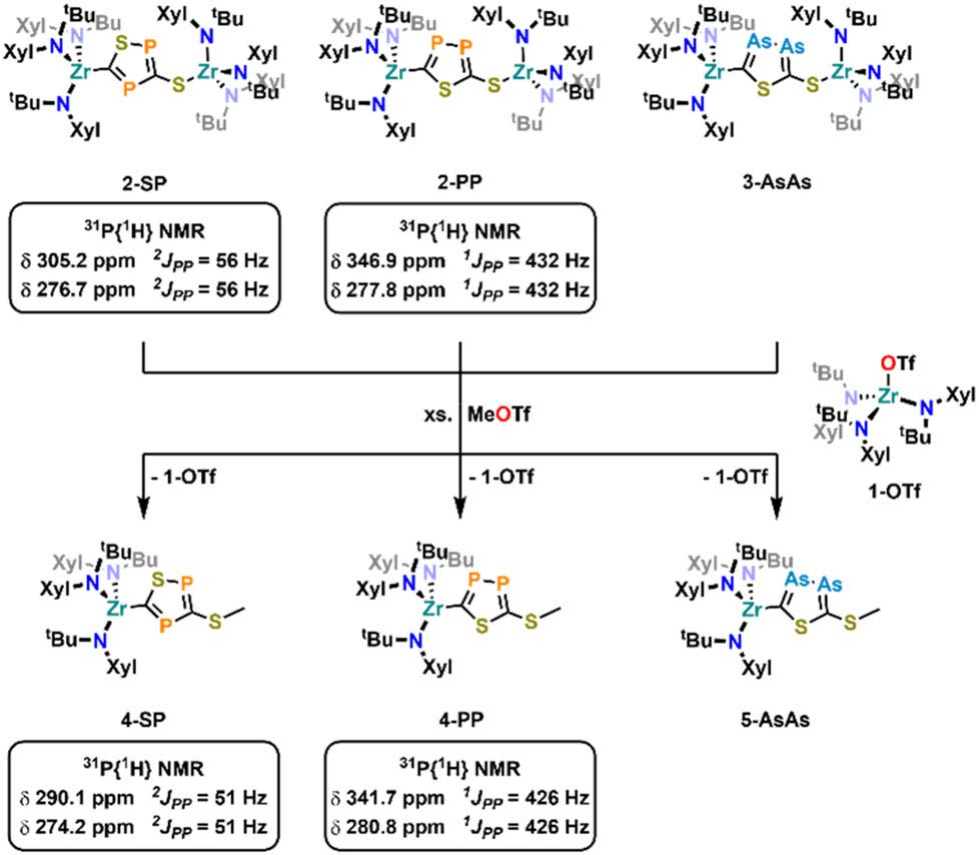
Attempted ring cleavage from the zirconium complexes using methyl triflate leading to the mono-methylated complexes 4-SP, 4-PP and 5-AsAs along with the “extrusion” of one equivalent of 1-OTf.

**Fig. 7:**
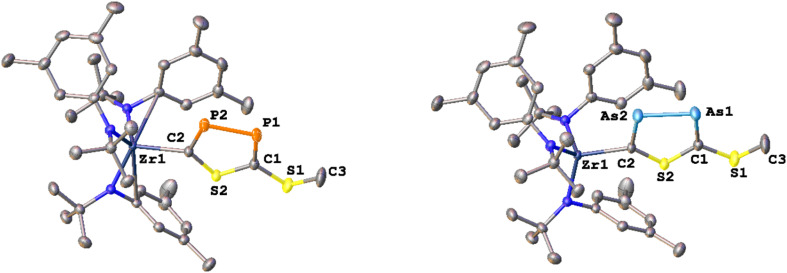
Molecular structure of 4-PP (left) and 5-AsAs (right). Hydrogen atoms have been omitted for clarity. Ellipsoids are shown at a probability level of 50%.

Finally, we attempted cleavage of the new formed heterocycles from the zirconium centers (Scheme 4). As an initial model reaction, we performed several test reactions using 2-PP as the substrate. While the addition of excess methyl iodide or TMS-Cl did not result in the formation of a new species, addition of approx. two equivalents of methyl triflate resulted in the formation of an equimolar mixture of two new species as observed by ^1^H NMR (Fig. S66). The ^31^P{^1^H} NMR of the crude mixture only shows the two resonances at 341.7 and 280.8 ppm (^1^*J*_PP_ = 426 Hz, Fig. S67), proving that the diphosphole is still intact after the reaction. In addition, a singlet resonance in the crude ^1^H NMR at 2.45 ppm, integrating for three protons, clearly indicates the installation of a methyl group on the molecule. However, this also indicates that only one side of the molecule has been cleaved. As mentioned, along with the formation of the new diphosphole moiety, the crude ^1^H NMR also indicates the formation of a second species, which we initially assigned as the triflate complex 1-OTf. Indeed, 1-OTf can be independently synthesized from 1-Cl through the addition of AgOTf (Fig. S46–S52, Molecular structure: Fig. S63) and its ^1^H-NMR characteristics fits well with one of the newly observed products (Fig. S66). Upon fractional crystallization, we were able to obtain pure fractions of the 2nd reaction product 4-PP (Fig. S53–S59). ^1^H NMR spectroscopic investigations of the product (Fig. S53) clearly show that one zirconium tris-amide fragment is still present, indicating that complete cleavage of the diphosphole is not possible even in the presence of an overstoichiometric amount of MeOTf. To answer the question, if the C- or S- terminus of the diphosphole ligand was methylated, we grew single crystals suitable for X-ray diffraction analysis from hexane at −40 °C (Fig. 7). The structure clearly reveals cleavage of the S-terminus giving access to a new thioether carbon–sulfur bond S1–C3 (1.787(2) Å). The zirconium carbon distance Zr1–C2 was found at 2.3194(16) Å, which is marginally longer compared to the parent complex 2-PP (2.294(5) Å). In contrast the bond parameters of the diphosphole ring in 4-PP remain mostly unchanged and are comparable to the parent complex 2-PP (compare Tables S1 and S2).

With these results in hand, we became interested if (partial) ring cleavage was also possible in the other diphosphole/diarsole complexes 2-SP and 3-AsAs. Indeed, crude NMR investigations after exposing 2-SP and 3-AsAs to approx. 2 equivalents of MeOTf revealed similar results as already described for 2-PP, with the crude reaction mixtures showing the formation of equimolar mixtures of 1-OTf and new complexes, most likely 4-SP and 5-AsAs (Fig. S68 and S70 respectively). Furthermore, the phosphorus NMR of crude 4-SP also shows the retention of the diphosphole heterocycle displaying two doublets at 290.1 and 274.2 ppm (^2^*J*_PP_ = 51 Hz, Fig. S69). The methyl group resonating at 2.46 ppm in the ^1^H NMR spectrum of 4-SP further indicates formation of a S-methylated heterocycle, similar to 4-PP (*δ*(CH_3_) = 2.45 ppm, *vide supra*). A similar resonance is found in the crude NMR of 5-AsAs (Fig. S60 and S70; 2.49 ppm) and we performed fractional crystallization of the reaction mixture to unambiguously proof *S*-methylation in 5-AsAs. X-ray quality crystals grown from concentrated hexane solution, clearly proof the formation of 5-AsAs (Fig. 7). A detailed discussion of the crystal/bonding parameters of the diarsole ring will be omitted here, since the differences between 5-AsAs/3-AsAs are only marginal (*vide supra* and Table S2). A detailed comparison of the bond parameters is presented in Tables S1 and S2 in the SI. Notably, TMS-OTf can also be applied as cleavage reagent, which gives access to the mono-silylated analogues of 4-PP and 4-SP as indicated by crude NMR analysis (see Fig. S71–S74), further indicating a surprising stability of the Zr–C bond in these complexes. However, it has to be stated, that the observed reactivity and the fact that only one zirconium atom can be cleaved off, somewhat hampers the general applicability of the new heterocycle in (coordination) chemistry. Future research in this field will be directed towards transmetallatable species, that allow a broader investigation of the spectroscopic, electrochemical and coordination properties of the new heterocycles.

## Conclusions

In conclusion we present the first (autogenic) [3 + 2] cycloaddition reactions with heavy cyanate anions [SCP]^−^ and [SCAs]^−^, mediated by zirconium. Regioselectivity of the observed cycloaddition reactions can be controlled by choice of solvent giving access to four (partly previously inaccessible) thiadiphosphole/thiadiarsole rings respectively. The straightforward and controllable access of all four heterocycles is notable, as simple synthetic routes to these entities have not yet been reported so far. All heterocycles described are thermally stable, with no signs of light-induced or thermal decomposition within several months.

This report highlights the differences between the well-explored phospha- and arsaethynolate anions [OCP]^−^ and [OCAs]^−^ compared to their heavier sulfur analogues. The fact that neither the [SCP]^−^ nor the [SCAs]^−^ anion can undergo facile CS extrusion blocks the most prominent decomposition path of their lighter analogues, which often extrude CO. Although full cleavage of both metal centers, giving access to the “free” heterocycles was not feasible under the conditions investigated, and only one zirconium center could be cleaved, the general accessibility of these heterocycles gives ample opportunities in chemical synthesis and potential new applications of them in (in-)organic, electro or medicinal chemistry.

## Author contributions

The project was designed by SH. Experimental work was performed by MB, DH and FH. X-Ray diffraction analysis was performed by MS and SH. Quantum chemical calculations were performed by FW. The manuscript was written by MB, MS, FW and SH. All authors have given approval to the final version of the manuscript.

## Conflicts of interest

There are no conflicts to declare.

## Supplementary Material

SC-OLF-D6SC01985D-s001

SC-OLF-D6SC01985D-s002

SC-OLF-D6SC01985D-s003

SC-OLF-D6SC01985D-s004

SC-OLF-D6SC01985D-s005

## Data Availability

CCDC 2365786, 2365789–2365793, 2518191, 2526017, 2526018 and 2530439 contain the supplementary crystallographic data for this paper.^54*a*–*j*^ All data is available free of charge from our side if requested. Raw data is stored on the university servers and can be accessed *via* us if necessary. Supplementary information: contains all ^1^H, ^13^C, ^31^P and 2D NMR spectra, IR and UV-vis data as well as further information regarding X-ray crystallography and computational investigations. *XYZ* coordinates are provided as a serparate SI file. See DOI: https://doi.org/10.1039/d6sc01985d.
