# Supplementary figures and images for: Regioselective [3 + 2] cycloaddition reactions of the phosphorus and arsenic analogues of the thiocyanate anion
Source: Chem Sci. 2026 Apr 24;17(22):10886–95. doi: 10.1039/d6sc01985d (PMC13175079; doi:10.1039/d6sc01985d)

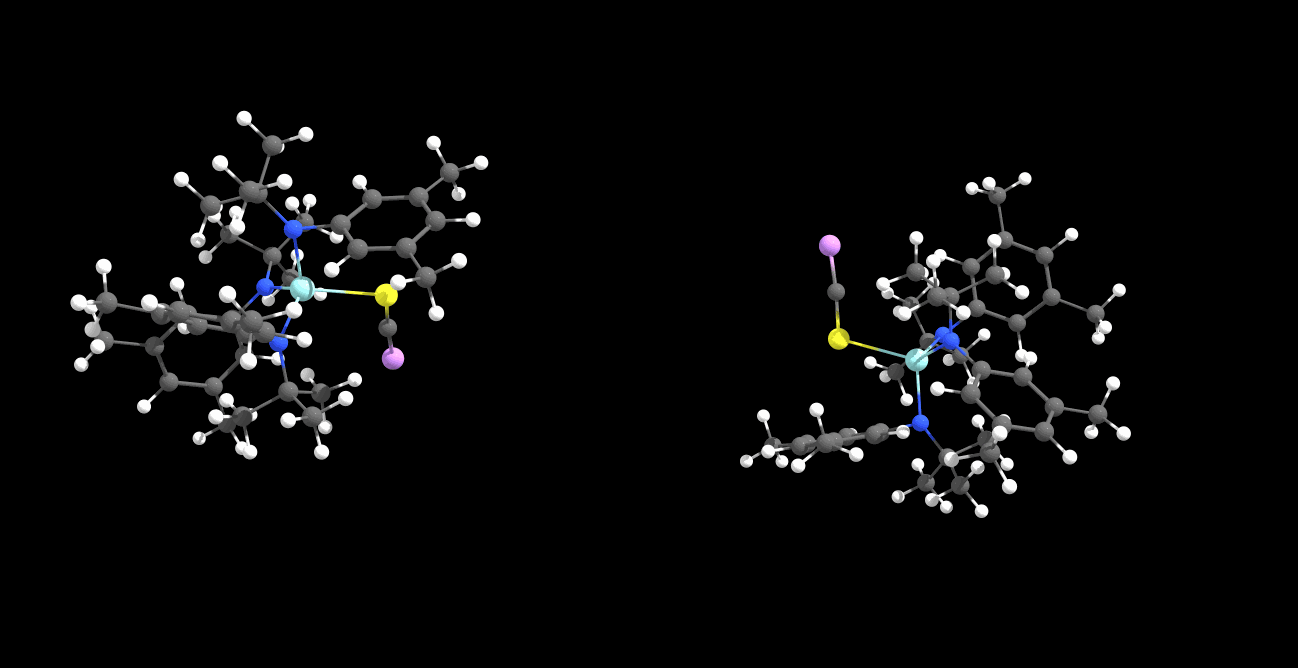

Supplement: SC-017-D6SC01985D-s001 [file SC-017-D6SC01985D-s001.gif]

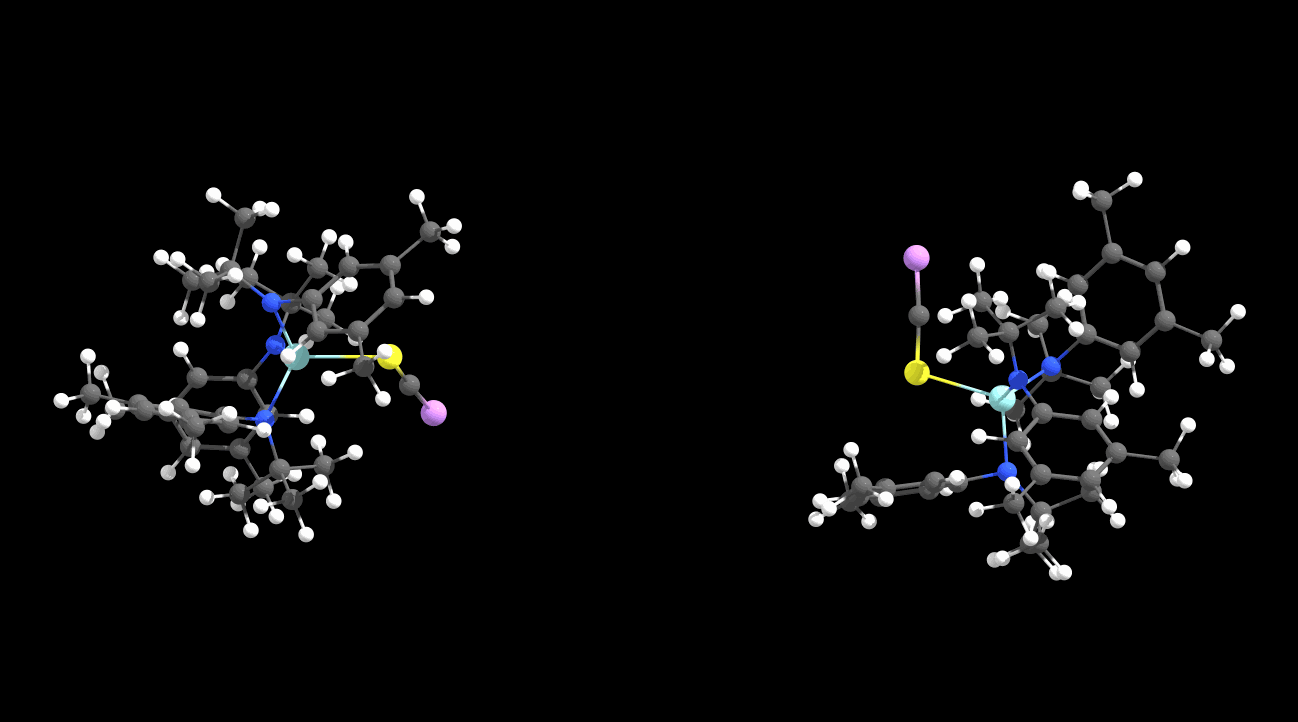

Supplement: SC-017-D6SC01985D-s002 [file SC-017-D6SC01985D-s002.gif]
