# Supplementary material for: Regioselective [3 + 2] cycloaddition reactions of the phosphorus and arsenic analogues of the thiocyanate anion
Source: Chem Sci. 2026 Apr 24;17(22):10886–95. doi: 10.1039/d6sc01985d (PMC13175079; doi:10.1039/d6sc01985d)
Supplement: SC-017-D6SC01985D-s004 [file SC-017-D6SC01985D-s004.pdf]

Supplementary Information (SI) for Chemical Science.
This journal is © The Royal Society of Chemistry 2026

Supporting Information

for

Regioselective [3+2] Cycloaddition Chemistry of the Phosphorus and Arsenic Analogues of the Thiocyanate Anion

Marc Baltrun, Florian Hett, Michael Seidl, Florian Weigend,* and Stephan Hohloch*

Table of Contents

Experimental Procedures 2

NMR spectroscopy 5

IR spectroscopy 79

UV-VIS-NIR Spectroscopy 82

Crystallographic Details 86

Computational Details / Methods 89

References 92

Experimental Procedures

General Methods

Unless stated otherwise, all transformations were conducted under an inert atmosphere using standard Schlenk techniques or an argon filled glove box (GS Mega line). High temperature reactions were carried out in preheated oil baths or aluminium blocks. Low temperature reactions were performed using a precooled aluminium block or dewar vessels filled with cooling mixtures consisting of ethanol/liquid nitrogen. Solvents for synthetic purposes were purified using an *MBraun* SPS system and stored over activated molecular sieves. Additionally, THF, 1,4-Dioxane, and 1,2-Dimethoxyethane (DME) were dried and distilled over sodium/benzophenone. Solvents used for aqueous workup or chromatography (technical grade) were used as received by a commercial supplier. All commercially available reagents were used as received. Deuterated solvents (benzene-d_6 and dichloromethane-d_2) were stored over activated molecular sieves. NMR spectra were recorded on *400 MHz Bruker Avance 4 Neo*, *600 MHz Avance II+*, and *700 MHz Avance 4 Neo* spectrometers. ^{1}H and ^{13}C{^{1}H} chemical shifts are reported in ppm and calibrated using residual solvent resonances. Elemental analyses (C, H, N) were performed on a *Vario Microtube* instrument. Unfortunately, due to the high air and moisture sensitivity and the sulfur amount in the majority of the samples, no satisfiable EA's could be obtained for the compounds.[1] ATR-Infrared spectroscopy was conducted on a *Bruker Alpha* IR spectrometer. UV-Vis-NIR spectra were recorded using an Avantes Ava-Spec NIR 1.7 spectrometer. [Na(Diox)$_3$][SCP] and [Na(Diox)$_3$][SCAs] was synthesized following literature known procedure. [2] Please note that the dioxane content of these salts might vary from batch to batch and need to be calculated by ^{1}H NMR spectroscopy in py-d$_5$ or THF-d$_8$ using naphthalene as an internal standard. Li[N(tBu)Xyl] · Et$_2$O was prepared as stated in the literature,[3] while for **1-Cl** a literature adapted procedure was used.[4]

X-ray crystallography

X-ray diffraction crystallography was performed at the University of Innsbruck (Bruker D8 Quest) and the University of Regensburg (Rigaku Synergy DW). All crystals were kept at 153(2) K (Bruker) or 100(2) K (Rigaku) throughout data collection. Data collection, refinement and reduction was performed using the ApexIV software (Bruker) or the CrysalisPro Software (Rigaku). All structures were solved with SHELXT[5] and refined using the OLEX 2 software package[6] using SHELXL least square refinements.[7] Strongly disordered solvent molecules have been removed using the SQUEEZE operation.[8] All nonhydrogen atoms were refined anisotropically, and hydrogen atoms were included at the geometrically calculated positions and refined using a riding model.

Synthetic procedures

Synthesis of 1-Cl

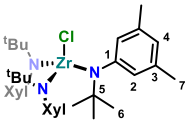

At ambient temperature, ZrCl$_4$ (1 eq., 5 mmol, 1.17 g) and Li[N(^{t}Bu)Xyl] · Et$_2$O (3.05 eq., 15.3 mmol, 3.92 g) were mixed in diethyl ether (30 ml). The mixture was stirred for 16 h before all volatiles were removed *in vacuo*. The residue was extracted using DCM (20 ml), and the filtrate was concentrated to ca. 6 ml and stored at 40 °C. After 24 h, the supernatant solution was removed and the colorless crystals were washed with cold pentane (-40 °C, 2x 5 ml) to obtain pure **2-Cl** (2.19 g, 3.34 mmol, 67%). Colorless crystals suitable for X-ray diffraction could be obtained from a concentrated diethyl ether solution stored at -40 °C. ^{1}H NMR (400 MHz, C$_6$D$_6$, 298 K): δ (ppm) = 6.76 (s, 9H, H^{2} and H^{4}), 2.25 (s, 18H, H^{7}), 1.28 (s, 27H, H^{6}). ^{13}C{^{1}H} NMR (101 MHz, C$_6$D$_6$, 298 K): δ (ppm) = 145.5 (C$_{Ar}$), 137.6 (C$_{Ar}$), 130.4 (C$_{Ar}$), 127.9 (C$_{Ar}$), 58.9 (C^{5}), 30.3 (C^{6}), 21.5 (C^{7}). ^{15}N NMR (41 MHz, C$_6$D$_6$, 298 K): δ (ppm) = 247.1. IR (cm^{-1}): 2971, 2952, 2912, 2863, 1599, 1586, 1454, 1386, 1356, 1290, 1239, 1211, 1178, 1153, 1043, 1019, 933, 890, 882, 849, 788, 717, 690, 576, 553, 486, 437, 414. Elem. Anal.(%) calc. for C$_{36}$H$_{54}$N$_3$Cl$_1$Zr$_1$: C 65.96 H 8.30 N 6.41; found: C 65.98 H 8.59 N 6.21.

A general denotation of the aromatic carbon resonances is used due to the overlap of resonances H^{2} and H^{4} as well as C$_{Ar}$(1-Cl) and C$_{Ar}$(C$_6$D$_6$). This complicates the clear assignment of resonances by 2D NMR spectra, given the available resolution.

Synthesis of 1-I

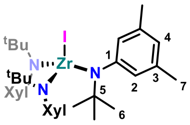

To a suspension of **1-Cl** (1 eq., 3.05 mmol, 2.0 g) in toluene (10 ml) was added TMS-I (3 eq., 9.15 mmol, 1.86 g) dropwisely. After 16 h at ambient temperatures, all volatiles were removed *in vacuo*. The residue was washed with hexane (2x 6 ml) thoroughly and dried *in vacuo* to obtain a colorless solid of pure **2-I** (2.04 g, 2.73 mmol, 90%). Colorless crystals suitable for X-ray diffraction could be obtained from a concentrated diethyl ether solution stored at -40 °C. ^{1}H NMR (400 MHz, C$_6$D$_6$, 298 K): δ (ppm) = 6.91 (s, 6H, H^{2}), 6.85 (s, 3H, H^{4}), 2.25 (s, 18H, H^{7}), 1.35 (s, 27H, H^{6}). ^{13}C{^{1}H} NMR (101 MHz, C$_6$D$_6$, 298 K): δ (ppm) = 139.9 (C^{1}), 138.2 (C^{3}), 132.9 (C^{2}), 129.6 (C^{4}), 59.7 (C^{5}), 30.5 (C^{6}), 21.4 (C^{7}). ^{15}N NMR (41 MHz, C$_6$D$_6$, 298 K): δ (ppm) = 250.7. IR (cm^{-1}): 3032, 2969, 2950, 2922, 2863, 2115, 1599, 1584, 1454, 1386, 1356, 1290, 1260, 1241, 1180, 1149, 1043, 1035, 1019, 978, 947, 931, 878, 845, 788, 712, 690, 676, 576, 559, 547, 539, 498, 486, 437, 416. UV-Vis-NIR: = 308 nm (ε = 37960 L mol^{-1} cm^{-1}). Elem. Anal.(%) calc. for C$_{36}$H$_{54}$N$_3$I$_1$Zr$_1$: C 57.89 H 7.29 N 5.63; found: C 58.49 H 7.49 N 5.46.

Synthesis of 1-OCP

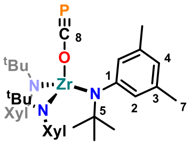

At ambient temperature, a solution of **1-Cl** (1 eq., 137 µmol, 90 mg) in toluene (3 ml) [Na(Diox)$_3$][OCP] (1.25 eq., 172 µmol, 60 mg) was added in solid form. Two drops of DME were added and the mixture was stirred for 16 h. After filtering off all solids, the filtrate was dried in vacuo and resolved in pentane (2 ml). Removing the solvent once again, an orange-red substance of pure **1-OCP** (57 mg, 84 µmol, 61%). 1H NMR (400 MHz, C6D6, 298 K):

δ (ppm) = 6.77 (s, 3H, H^4), 6.62 (s, 6H, H^2), 2.26 (s, 18H, H^7), 1.21 (s, 27H, H^6). $^{13}C\{^1H\}$ NMR (101 MHz, C_6D_6, 298 K): δ (ppm) = 144.6 (C^1), 141.5 (C^8), 138.1 (C^3), 129.9 (C^2), 128.6 (C^4), 59.0 (C^5), 29.9 (C^6), 21.6 (C^7). $^{31}P\{^1H\}$ NMR (162 MHz, C_6D_6, 298 K): δ (ppm) = -323.6. ^{15}N NMR (41 MHz, C_6D_6, 298 K): δ (ppm) = 245.6. IR (cm^{-1}): 2987, 2931, 2878, 1711, 1620, 1493, 1423, 1330, 1278, 1217, 1084, 976, 905, 835, 762, 605, 535, 462. UV-Vis-NIR: = 313 nm (ε = 2661 L mol^{-1} cm^{-1}). Elem. Anal.(%) calc. for $C_{37}H_{54}N_3O_1P_1Zr_1$: C 65.44 H 8.02 N 6.19; found: C 65.58 H 8.05 N 6.11.

Synthesis of 1-OTf

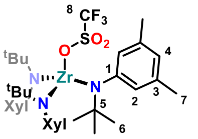

The addition of solid silver triflate (1.5 eq., 405 µmopl, 104 mg) to a colorless solution of **1-Cl** (1 eq., 270 µmol, 177 mg) in Et_2O (5 ml) resulted in a salmon suspension. After 30 minutes at ambient temperature, the suspension was filtered so separate the silver salts. The filtrate was evaporated, and the product was extracted using *n*-hexane (5 ml) to obtain a colorless foam of pure **1-OTf** (194 mg, 252 µmol, 93%). Few crystals suitable for sc-XRD were eventually obtained from a very concentrated hexane solution (<2 ml) after several days at -40 °C. ^1H NMR (400 MHz, C_6D_6, 298 K): δ (ppm) = 6.83 (s, 3H, H^4), 6.82 (s, 6H, H^2), 2.28 (s, 18H, H^7), 1.22 (s, 27H, H^6). $^{13}C\{^1H\}$ NMR (101 MHz, C_6D_6, 298 K): δ (ppm) = 142.15 (s, C^1), 138.73 (s, C^3), 130.66 (s, C^2), 129.64 (s, C^4), 120.48 (q, J = 318.7 Hz, C^8), 59.55 (C^5), 29.92 (C^6), 21.45 (C^7). Due to sensitivity of the compound in combination with its fluorine content no reliable elemental analysis could be obtained.

*The NMR nomenclature for **2-PP**, **2-SP**, **3-AsAs**, and **3-SAs** will differ from the previous as it is at least impossible to assign one set of ligand signals to the right nucleus connected to the cycle, sometimes it is even impossible to assign one sides aromatic group to the according alkyl group signals.*

Synthesis of 2-PP

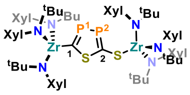

At ambient temperature, to **1-I** (1 eq., 200 µmol, 149 mg) and [Na(Diox)$_3$][SCP] (1.25 eq., 250 µmol, 90.6 mg) was added THF (4 ml) to immediately observe a suspension of intense red color. After stirring it for 4 h, the mixture was filtrated over celite and the filtrate evaporated to dryness (crude yield 94%). The red-brown solid was extracted using diethyl ether (12 ml) and the diethyl ether solution was concentrated to ca. 4 ml to observe the initial formation of crystals. After 3 days, the supernatant solution is removed and the crystals were carefully washed with hexane (2x 1ml) to obtain pure **2-PP** (65 mg, 93.5 µmol, 47 %). Yellow crystals suitable for X-ray diffraction could be obtained from slowly evaporating a concentrated diethyl ether solution at ambient temperature over several days. ^1H NMR (400 MHz, C_6D_6, 298 K): δ (ppm) = 7.08 (s, 6H, H_{Ar}), 7.05 (s, 6H, H_{Ar}), 6.80 (s, 3H, H_{Ar}), 6.72 (s, 3H, H_{Ar}), 2.30 (s, 18H, CH_{3Ar}), 2.29 (s, 18H, CH_{3Ar}), 1.39 (s, 27H, H_{tBu}), 1.30 (s, 27H, H_{tBu}). $^{13}C\{^1H\}$ NMR (151 MHz, C_6D_6, 298 K): δ (ppm) = 227.8 (d, $^1J_{CP}$ = 117.2 Hz, C^1), 195.5 (dd, $^1J_{CP}$ = 93.3 Hz, $^2J_{CP}$ = 6.7 Hz, C^2), 147.6 (s, C_{Ar}), 144.6 (s, C_{Ar}), 138.2 (s, C_{Ar}), 137.4 (s, C_{Ar}), 132.4 (s, C_{Ar}), 131.3 (s, C_{Ar}), 129.2 (s, C_{Ar}), 127.6 (s, C_{Ar}), 59.8 (s, CMe_3), 59.6 (s, CMe_3), 30.4 (s, CH_{3Ar}), 30.3 (s, CH_{3Ar}), 21.6 (s, CH_{3tBu}), 21.6 (CH_{3tBu}). $^{31}P\{^1H\}$ NMR (162 MHz, C_6D_6, 298 K): δ (ppm) = 346.8 (d, $^1J_{PP}$ = 433.3 Hz, P^1), 276.8 (d, $^1J_{PP}$ = 433.3 Hz, P^2) ^{15}N NMR (41 MHz, C_6D_6, 298 K): δ (ppm) = 242.9, 252.4. IR (cm^{-1}): 3232, 2967, 2916, 2867, 1644, 1599, 1519, 1464, 1411, 1390, 1364, 1341, 1223, 1182, 1129, 1039, 968, 933, 841, 821, 690, 647, 465, 420, 408. UV-Vis-NIR: = 315 nm (ε = 8510 L mol^{-1} cm^{-1}), 363 nm (ε = 7640 L mol^{-1} cm^{-1}). Elem. Anal.(%) calc. for $C_{74}H_{108}N_6P_2S_2Zr_2$: C 63.93 H 7.83 N 6.05; found: C 63.69 H 8.01 N 5.94.

Synthesis of 2-SP

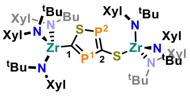

At ambient temperature, **1-I** (1 eq., 200 µmol, 149 mg) and [Na(Diox)$_3$][SCP] (1.25 eq., 250 µmol, 90.6 mg) was suspended in toluene (4 ml). After stirring for 16 h, the volume of the reaction mixture was increased to ca. 10 ml by adding toluene before it was filtered over celite. The orange filtrate was evaporated to dryness (crude yield: 82%) and extracted using diethyl ether (12 ml). The diethyl ether solution was concentrated to ca. 4 ml which already started crystallization. After 2 days, the supernatant solution was removed, the yellow crystals were carefully washed with hexane (2x 1 ml) to obtain pure **2-SP** (48 mg, 69 µmol, 35%). Yellow crystals suitable for X-ray diffraction could be obtained from slowly evaporating a concentrated diethyl ether solution at ambient temperature over several days. ^1H NMR (400 MHz, C_6D_6, 298 K): δ (ppm) = 7.05 (s, 6H, H_{Ar}), 7.04 (s, 6H, H_{Ar}), 6.78 (s, 3H, H_{Ar}), 6.71 (s, 3H, H_{Ar}), 2.31 (s, 18H, CH_{3Ar}), 2.29 (s, 18H, CH_{3Ar}), 1.35 (s, 27H, H_{tBu}), 1.30 (s, 27H, H_{tBu}). $^{13}C\{^1H\}$ NMR (151 MHz, C_6D_6, 298 K): δ (ppm) = 235.6 (d, $^1J_{CP}$ = 103.3 Hz, C^1), 199.4 (dd, $^1J_{CP}$ = 86.1 Hz, $^1J_{CP}$ = 97.5 Hz, C^2), 145.1 (s, C_{Ar}), 142.9 (s, C_{Ar}), 138.0 (s, C_{Ar}), 137.4 (s, C_{Ar}), 132.1 (s, C_{Ar}), 131.1 (s, C_{Ar}), 129.0 (s, C_{Ar}), 128.0 (s, C_{Ar}), 59.7 (s, CMe_3), 59.6 (s, CMe_3), 30.5 (s, CH_{3Ar}), 30.3 (s, CH_{3Ar}), 21.6 (s, CH_{3tBu}), 21.6 (s, CH_{3tBu}). $^{31}P\{^1H\}$ NMR (162 MHz, C_6D_6, 298 K): δ (ppm) = 305.2 (d, $^2J_{PP}$ = 56.5 Hz, P^1), 276.7 (d, $^2J_{PP}$ = 56.5 Hz, P^2). ^{15}N NMR (41 MHz, C_6D_6, 298 K): δ (ppm) = 251.2, 245.0. IR (cm^{-1}): 2967, 2910, 1648, 1599, 1586, 1452, 1388, 1356, 1288, 1180, 1149, 1041, 974, 931, 882, 874, 843, 808, 786, 714, 690, 678, 580, 547, 502, 480, 437, 410. UV-Vis-NIR: = 326 nm (ε = 100790 L mol^{-1} cm^{-1}). Elem. Anal.(%) calc. for $C_{74}H_{108}N_6P_2S_2Zr_2$: C 63.93 H 7.83 N 6.05; found: C 63.65 H 8.05, N 5.96.

Synthesis of 3-AsAs

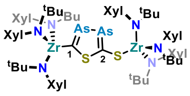

The solids of **1-I** (1 eq., 220 µmol, 164 mg) and [Na(Diox)$_3$][SCAs] (1.5 eq., 330 µmol, 134 mg) were solved in 4 ml of THF to immediately observe a red solution. After stirring for 2 h at ambient temperatures, all volatiles were removed *in vacuo* (crude yield: 91%). The residue was extracted using hexane (6 ml), and the filtrate concentrated to approximately half its volume. Over the course of 24 hours, yellow-orange crystals (88 mg, 119 µmol, 60%) were collected from the concentrated solution. Yellow-orange crystals suitable for X-ray diffraction could be obtained from slowly evaporating a concentrated hexane solution at ambient temperature over one to two days. ^1H NMR (400 MHz, C_6D_6, 298 K): δ (ppm)

=7.06 (s, 6H, H_{Ar}), 7.05 (s, 6H, H_{Ar}), 6.81 (s, 3H, H_{Ar}), 6.70 (s, 3H, H_{Ar}), 2.30 (s, 36H, CH_{3Ar}), 1.36 (s, 27H, H_{tBu}), 1.30 (s, 27H, H_{tBu}). $^{13}C\{^{1}H\}$ NMR (101 MHz, C_6D_6, 298 K): δ (ppm) = 241.3 (s, C^1), 206.1 (s, C^2), 144.8 (s, C_{Ar}), 143.3 (s, C_{Ar}), 138.0 (s, C_{Ar}), 137.4 (s, C_{Ar}), 132.2 (s, C_{Ar}), 131.1 (s, C_{Ar}), 129.0 (s, C_{Ar}), 127.9 (s, C_{Ar}), 59.8 (s, CMe_3), 59.6 (s, CMe_3), 30.5 (s, CH_{3Ar}), 30.3 (s, CH_{3Ar}), 21.6 (s, CH_{3tBu}), 21.6 (CH_{3tBu}). ^{15}N NMR (41 MHz, C_6D_6, 298 K): δ (ppm) = 252.2, 242.7. IR (cm^{-1}): 2967, 2916, 2863, 1599, 1586, 1521, 1454, 1388, 1374, 1358, 1288, 1180, 1149, 1041, 1021, 974, 933, 904, 874, 843, 823, 786, 763, 714, 688, 678, 580, 559, 549, 490, 437, 414. UV-Vis-NIR: = 326 nm (ε = 57130 L mol^{-1} cm^{-1}), 373 nm (ε = 60430 L mol^{-1} cm^{-1}), 379 nm (ε = 60860 L mol^{-1} cm^{-1}), 398 nm (ε = 66390 L mol^{-1} cm^{-1}). Elem. Anal.(%) calc. for $C_{74}H_{108}N_6As_2S_2Zr_2$: C 60.13 H 7.36 N 5.69; found: C 59.22 H 7.33 N 5.32. *(The low carbon content can be explained by potential carbide formation, which is typical in early transition metal chemistry)*

Synthesis of 3-SAs (in a mixture with 3-AsAs)

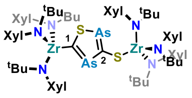

The solids of **1-I** (1 eq., 220 µmol, 164 mg) and [Na(Diox)$_3$][SCAs] (1.5 eq., 330 µmol, 134 mg) were suspended in toluene (5 ml) to immediately observe an intense red suspension. After 16 h, the mixture was filtrated over celite and the filtrate was evaporated to dryness (crude yield: 87%). Extraction with hexane (5 ml) yielded a red solution which was left undisturbed at ambient temperature for 2 days. The supernatant solution was removed and the red crystals as well as the yellow-orange precipitate was carefully washed with hexane (2x 1 ml) and subsequently dried *in vacuo*. Yield: 31 mg (41.9 µmol, 21%) of a **51:49** mixture of **3-SAs** and **3-AsAs**.^{1}H NMR (400 MHz, C_6D_6, 298 K): δ (ppm) = 7.05 (s, 6H, H_{Ar}), 7.01 (s, 6H, H_{Ar}), 6.78 (s, 3H, H_{Ar}), 6.71 (s, 3H, H_{Ar}), 2.30 (s, 18H, CH_{3Ar}), 2.28 (s, 18H, CH_{3Ar}), 1.33 (s, 27H, H_{tBu}), 1.28 (s, 27H, H_{tBu}). $^{13}C\{^{1}H\}$ NMR (101 MHz, C_6D_6, 298 K): δ (ppm) = 263.37 (s, C^1), 229.93 (s, C^2), 145.22 (s, C_{Ar}), 143.71 (s, C_{Ar}), 137.92 (s, C_{Ar}), 137.47 (s, C_{Ar}), 131.89 (s, C_{Ar}), 130.95 (s, C_{Ar}), 128.75 (s, C_{Ar}), 127.94 (s, C_{Ar}), 59.78 (s, CMe_3), 59.60 (s, CMe_3), 30.51 (s, CH_{3Ar}), 30.27 (s, CH_{3Ar}), 21.65 (s, CH_{3tBu}), 21.62 (s, CH_{3tBu}). ^{15}N NMR (41 MHz, C_6D_6, 298 K): δ (ppm) = 242.97, 252.09.
*Note, the crude reaction mixture prior to crystallization is 47:53, while after crystallization the described 51:49 ratio is obtained. Nevertheless, no pure material of 4-SAs was obtained. Thus, only one-dimensional ^{1}H and ^{13}C spectra are shown (*Figure S 43 and Figure S 45*) as the resolution of the two-dimensional spectra is not sufficient to differentiate between correlations of **3-AsAs** and **3-SAs**.*

Synthesis of 4-PP

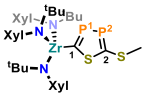

At ambient temperature, to a solution of **2-PP** (1 eq., 250 µmol, 348 mg) in toluene (4 ml) was added a solution of methyl triflate in toluene (1.2 eq., 300 µmol, 0.05 M, 6 ml). The resulting off-white solution was stirred for 3 h at ambient temperature before all volatiles were removed *in vacuo*. Two consecutive steps of crystallising the product from its concentrated hexane solution at -40 °C and washing the crystals with small amounts of cold hexane (-40 °C) yielded pure **4-PP** (41 mg, 52 µmol, 21%) as a colorless solid. ^{1}H NMR (500 MHz, C_6D_6, 298 K): δ (ppm) = 7.03 (s, 6H, H_{Ar}), 6.75 (s, 3H, H_{Ar}), 2.45 (s, 3H, SCH_3), 2.24 (s. 18H, CH_{3Ar}), 1.32 (s, 27H, H_{tBu}). $^{13}C\{^{1}H\}$ NMR (151 MHz, C_6D_6, 298 K): δ (ppm) 228.0 (d, $^{1}J_{CP}$ = 120 Hz, C^1), 194.1 (dd, $^{1}J_{CP}$ = 103 Hz, $^{2}J_{CP}$ = 7.0 Hz, C^2), 141.8 (s, C_{Ar}), 138.3 (s, C_{Ar}), 132.5 (s, C_{Ar}), 129.4 (s, C_{Ar}), 59.7 (CMe_3), 30.4 (s, CH_{3Ar}), 24.5 (d, $^{3}J_{CP}$ = 13.2 Hz, SCH_3), 21.4 (s, CH_{3tBu}). $^{31}P\{^{1}H\}$ NMR (202 MHz, C_6D_6, 298 K): δ (ppm) = 341.7 (d, $^{1}J_{PP}$ = 426.3 Hz, P^1), 280.4 (d, $^{1}J_{PP}$ = 426.3 Hz, P^2). ^{15}N NMR (51 MHz, C_6D_6, 298 K): δ (ppm) = 245.0. IR (cm^{-1}): 2987, 2933, 2876, 1618, 1489, 1421, 1330, 1278, 1225, 1064, 1014, 919, 859, 760, 599, 531, 476. UV-Vis-NIR: = 319 nm (ε = 4710 L mol^{-1} cm^{-1}), 353 nm (ε = 5035 L mol^{-1} cm^{-1}). Elem. Anal.(%) calc. for $C_{39}H_{57}N_3P_2S_2Zr_1$: C 59.66 H 7.32 N 5.35; found: C 59.83 H 7.45 N 5.17.

Synthesis of 5-AsAs

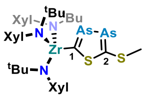

At ambient temperature, to a solution of **3-AsAs** (1 eq., 135 µmol, 200 mg) in toluene (3 ml) was added a solution of methyl triflate in toluene (2 eq., 300 µmol, 0.2 M,1.35 ml). The resulting yellow solution was stirred for 3 h at ambient temperature before all volatiles were removed *in vacuo*. Two consecutive steps of crystallizing the product from its concentrated hexane solution at -40 °C and washing the crystals with small amounts of cold hexane (-40 °C) yielded pure **5-AsAs** (4 mg, 5 µmol, 3 %) as a yellow solid. ^{1}H NMR (500 MHz, C_6D_6, 298 K): δ (ppm) = 7.04 (s, 6H, H_{Ar}), 6.75 (s, 6H, H_{Ar}), 2.49 (s, 3H, SCH_3), 2.24 (s. 18H, CH_{3Ar}), 1.32 (s, 27H, H_{tBu}). $^{13}C\{^{1}H\}$ NMR (126 MHz, C_6D_6, 298 K): δ (ppm) = 142.39 (s, C_{Ar}), 138.21 (s, C_{Ar}), 132.36 (s, C_{Ar}), 129.26 (s, C_{Ar}), 59.72 (s, CMe_3), 30.45 (s, CH_{3Ar}), 26.90 (s, SCH_3), 21.47 (s, CH_{3tBu}). ^{15}N NMR (51 MHz, C_6D_6, 298 K): δ (ppm) = 244.2.

NMR spectroscopy

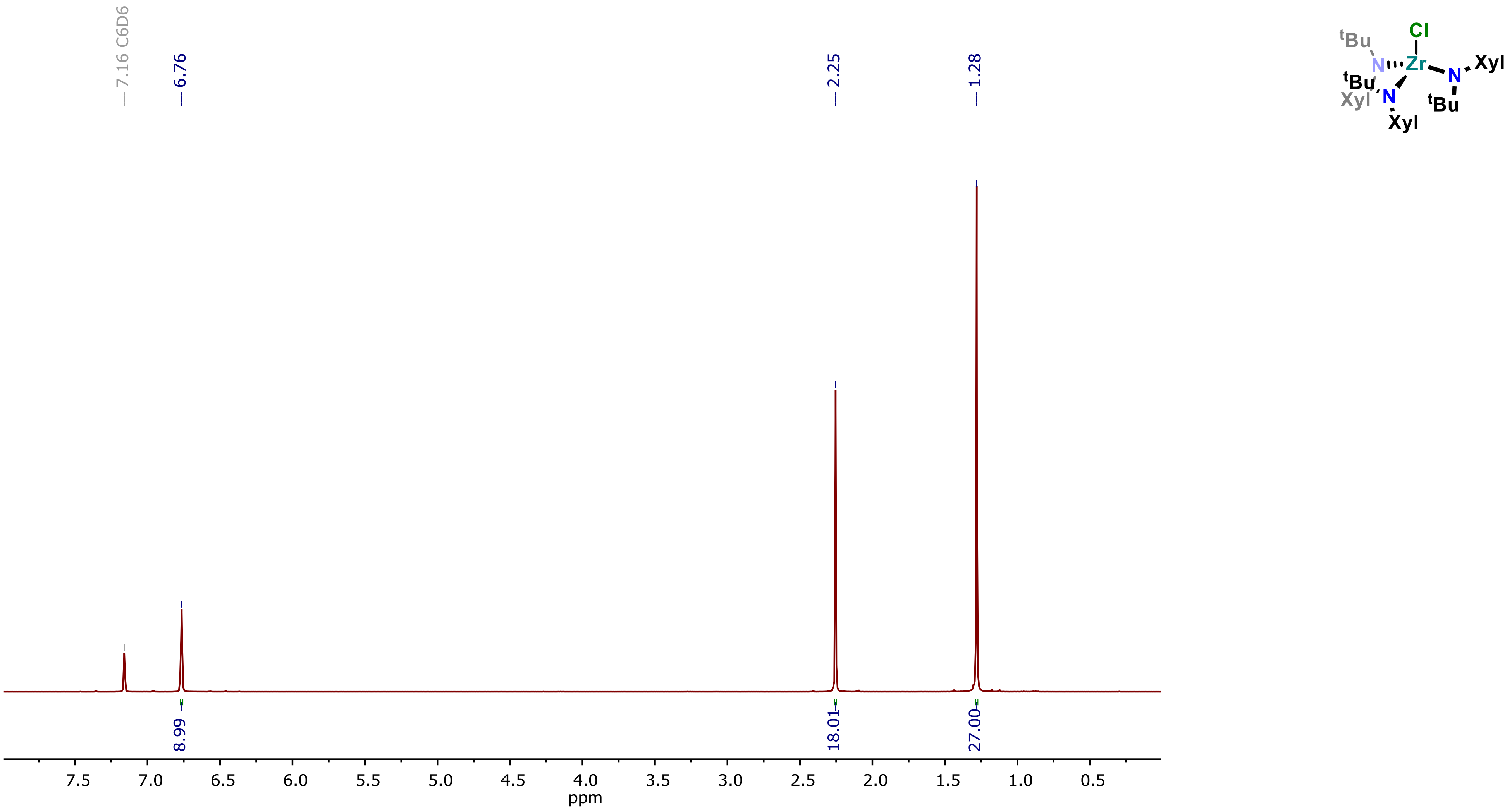

*Figure S 1: ^{1}H NMR spectrum of **1-Cl** in C_6D_6 at 298 K.*

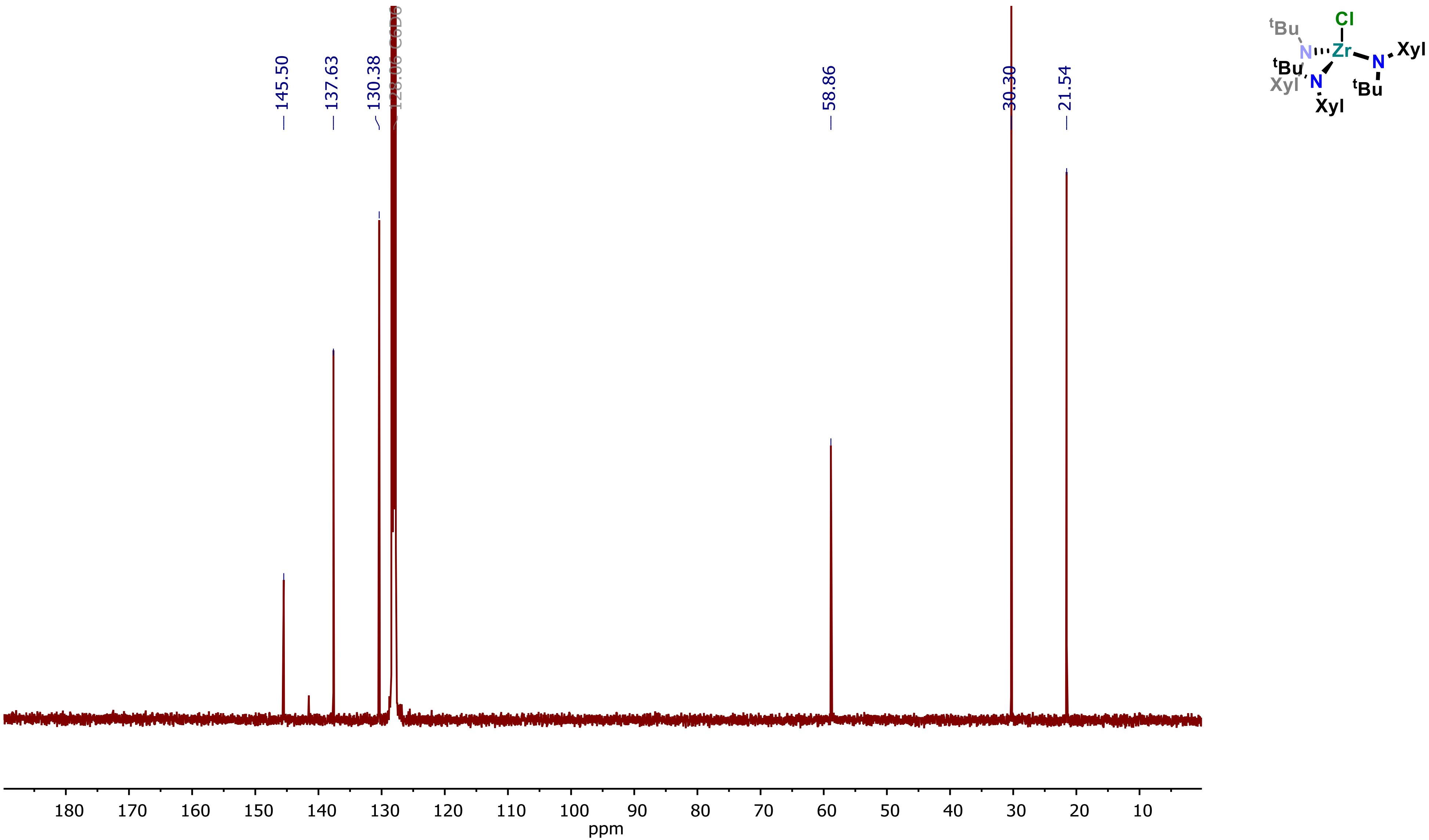

*Figure S 2: $^{13}C\{^{1}H\}$ NMR spectrum of **1-Cl** in C_6D_6 at 298 K.*

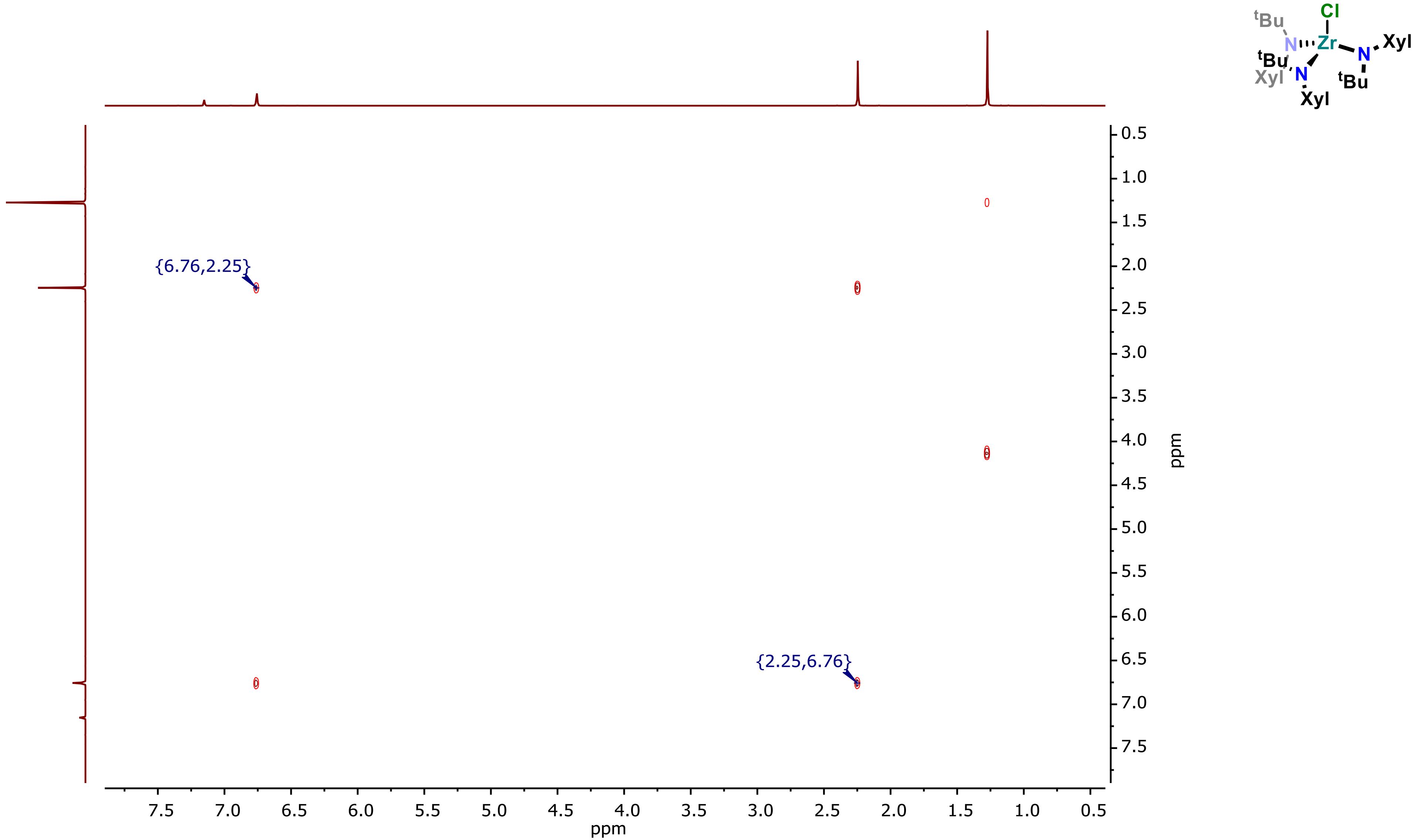

*Figure S 3: ^{1}H-^{1}H COSY NMR spectrum of **1-Cl** in C_6D_6 at 298 K.*

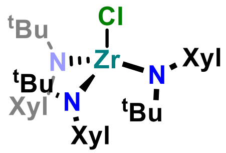

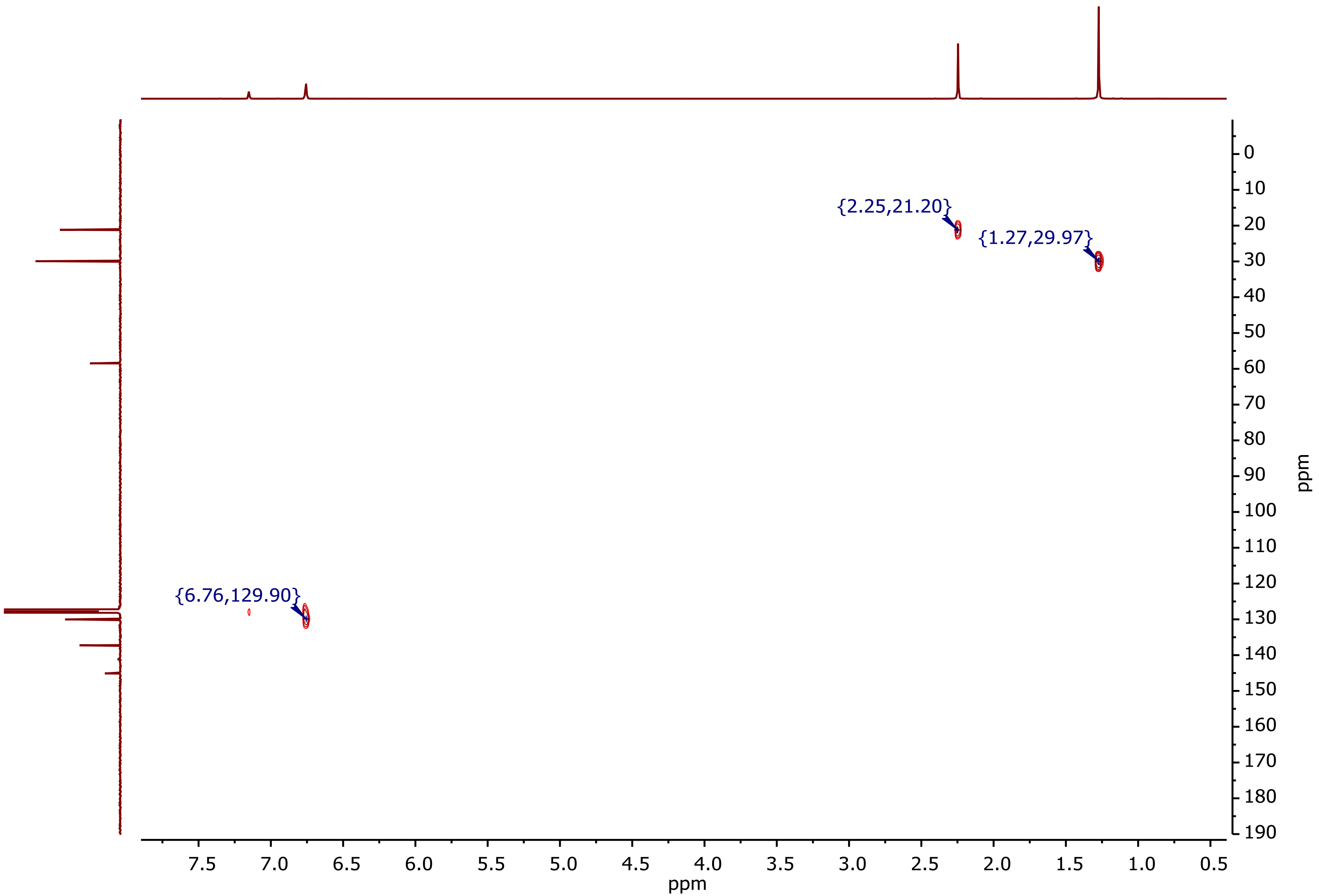

*Figure S 4: 1H-13C HSQC NMR spectrum of **1-Cl** in C_6D_6 at 298 K.*

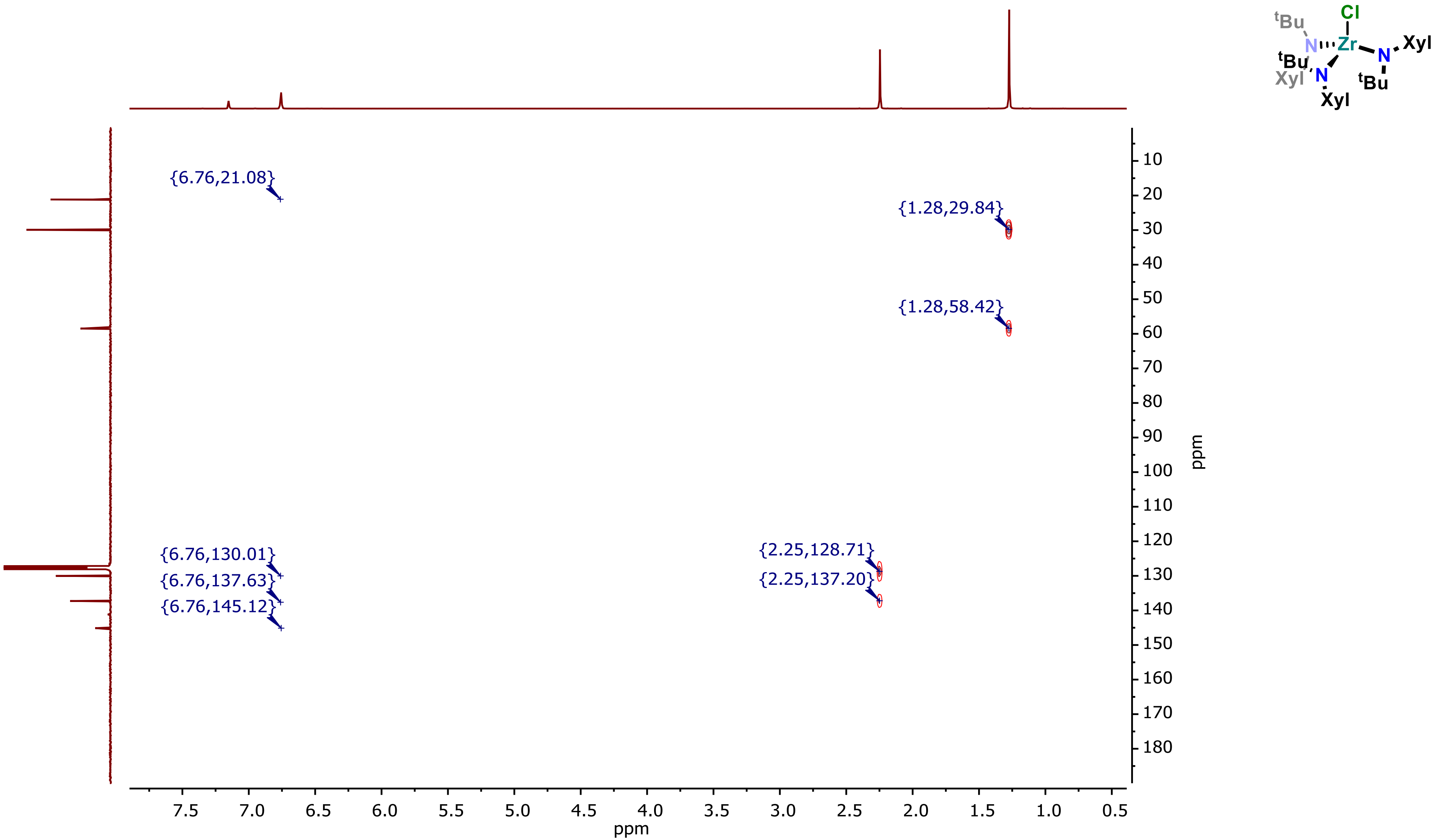

*Figure S 5: ^{1}H-^{13}C HMBC NMR spectrum of **1-Cl** in C_6D_6 at 298 K.*

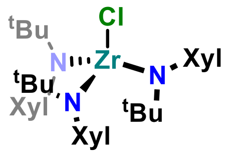

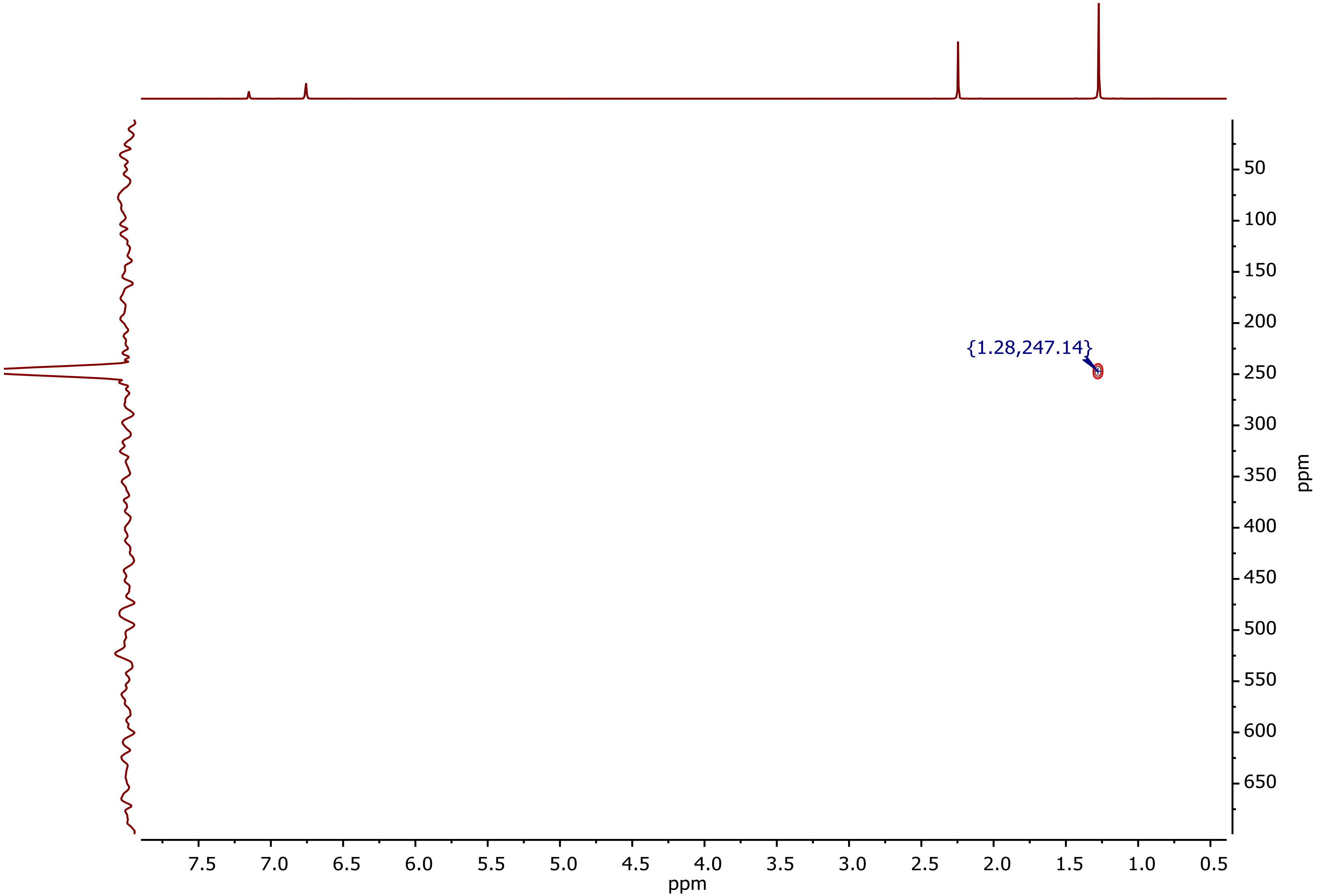

*Figure S 6: ^{1}H-^{15}N HMBC NMR spectrum of **1-Cl** in C_6D_6 at 298 K.*

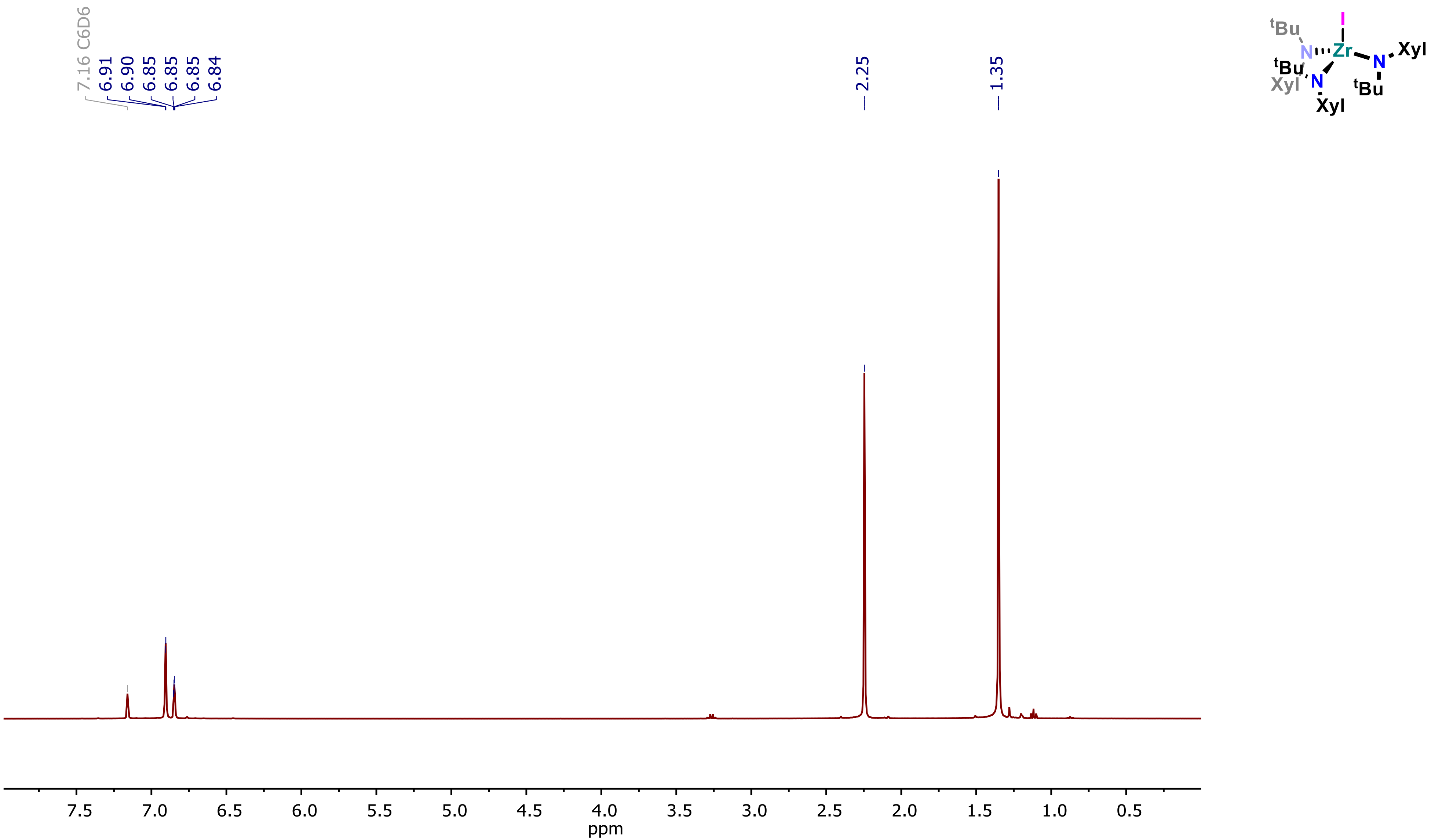

*Figure S 7: ^{1}H NMR spectrum of **1-I** in C_6D_6 at 298 K.*

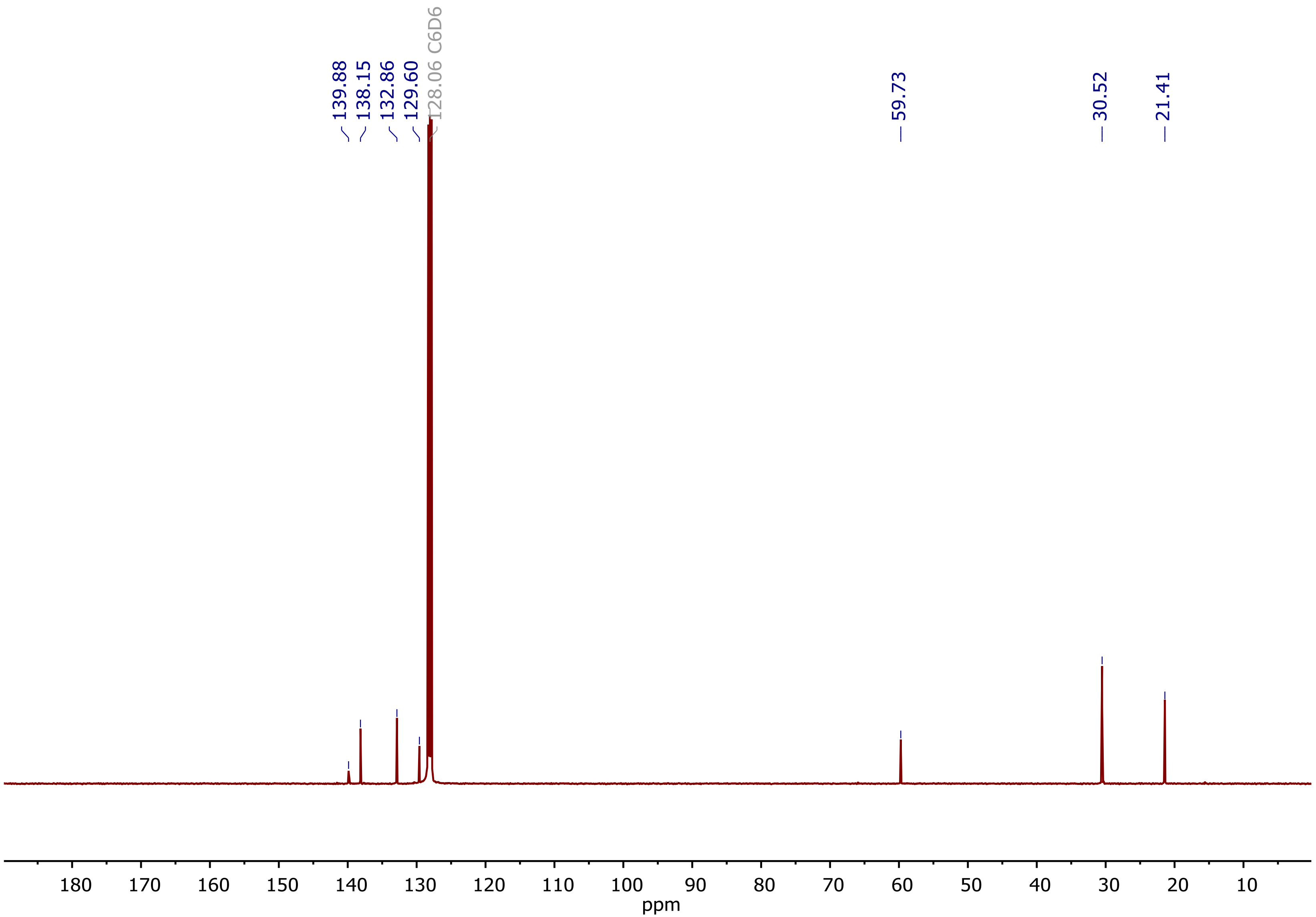

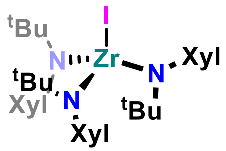

*Figure S 8: $^{13}C\{^{1}H\}$ NMR spectrum of **1-I** in C_6D_6 at 298 K.*

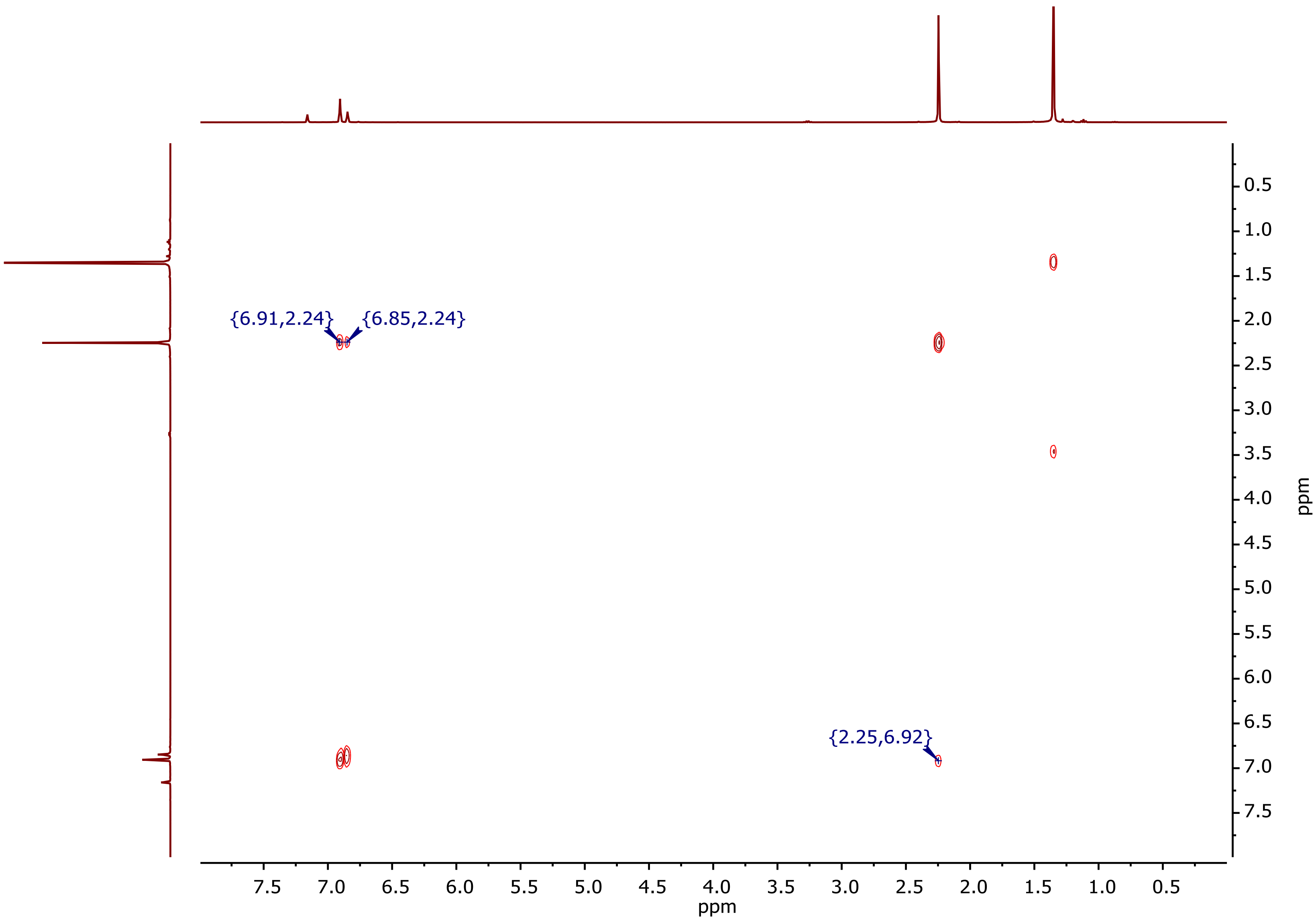

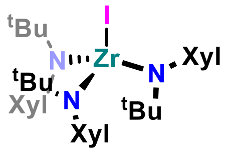

*Figure S 9: ^{1}H-^{1}H COSY NMR spectrum of **1-I** in C_6D_6 at 298 K.*

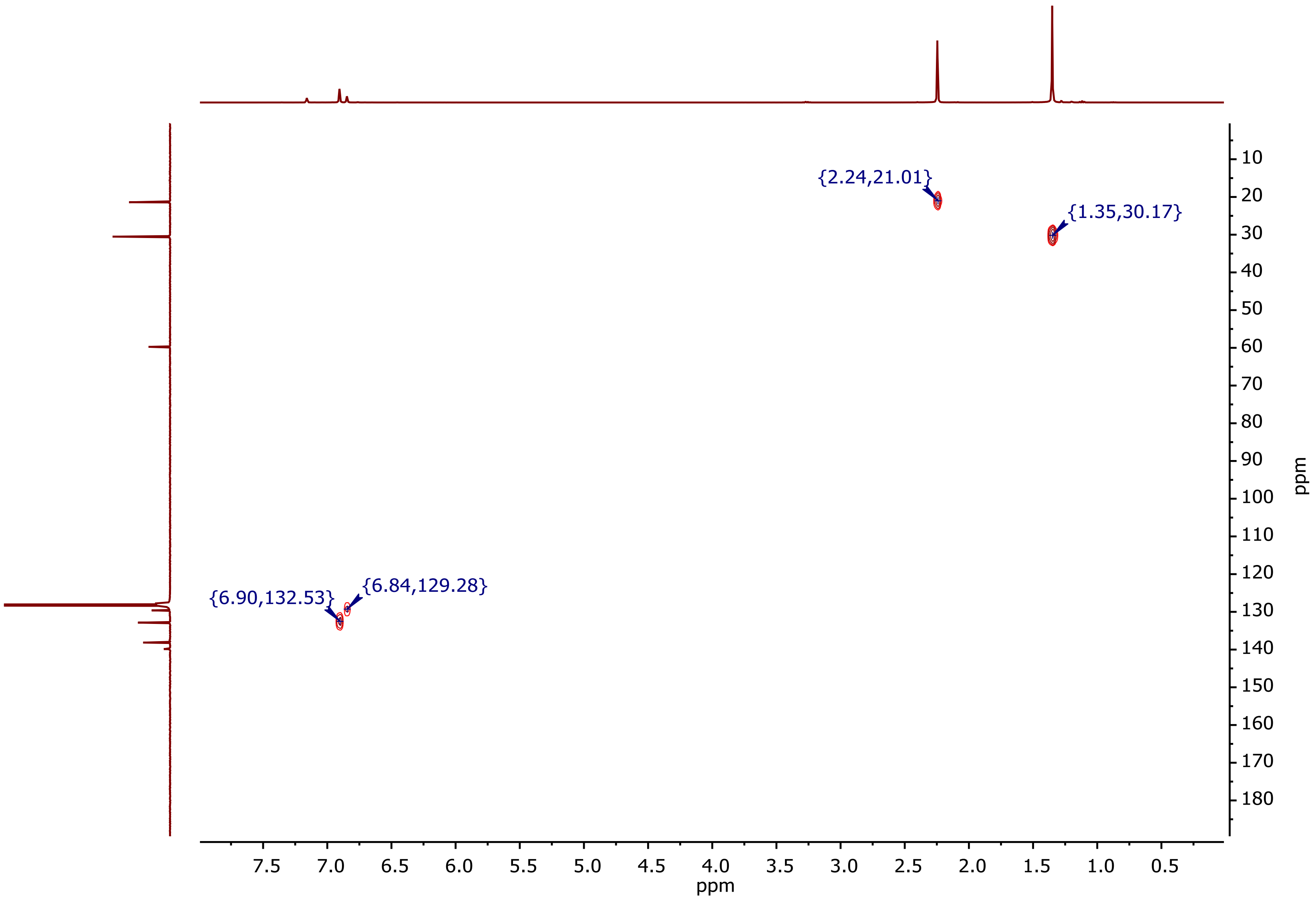

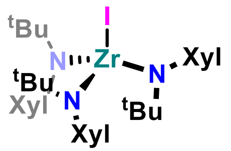

*Figure S 10: ^{1}H-^{13}C HSQC NMR spectrum of **1-I** in C_6D_6 at 298 K.*

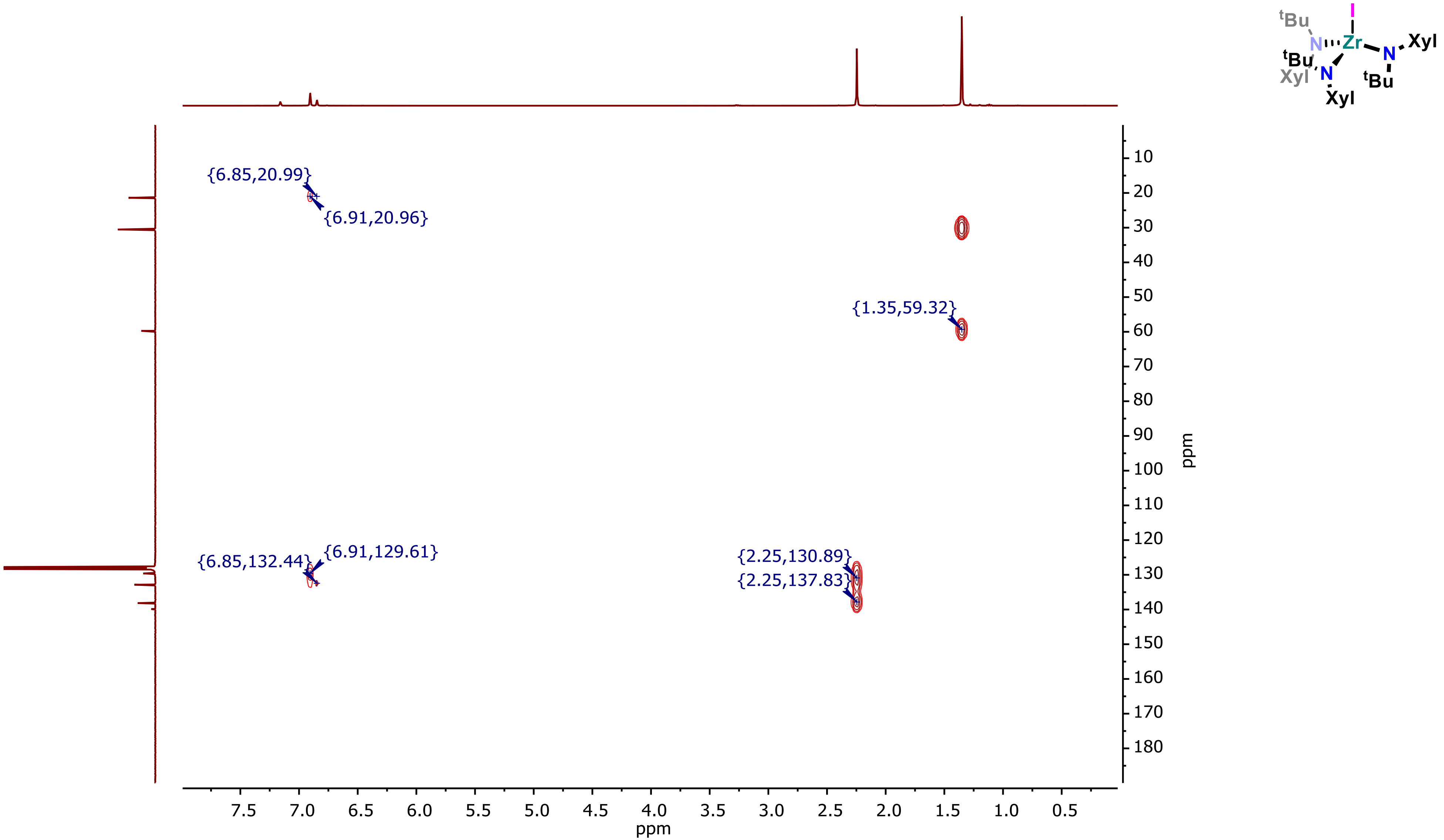

*Figure S 11: ^{1}H-^{13}C HMBC NMR spectrum of **1-I** in C_6D_6 at 298 K.*

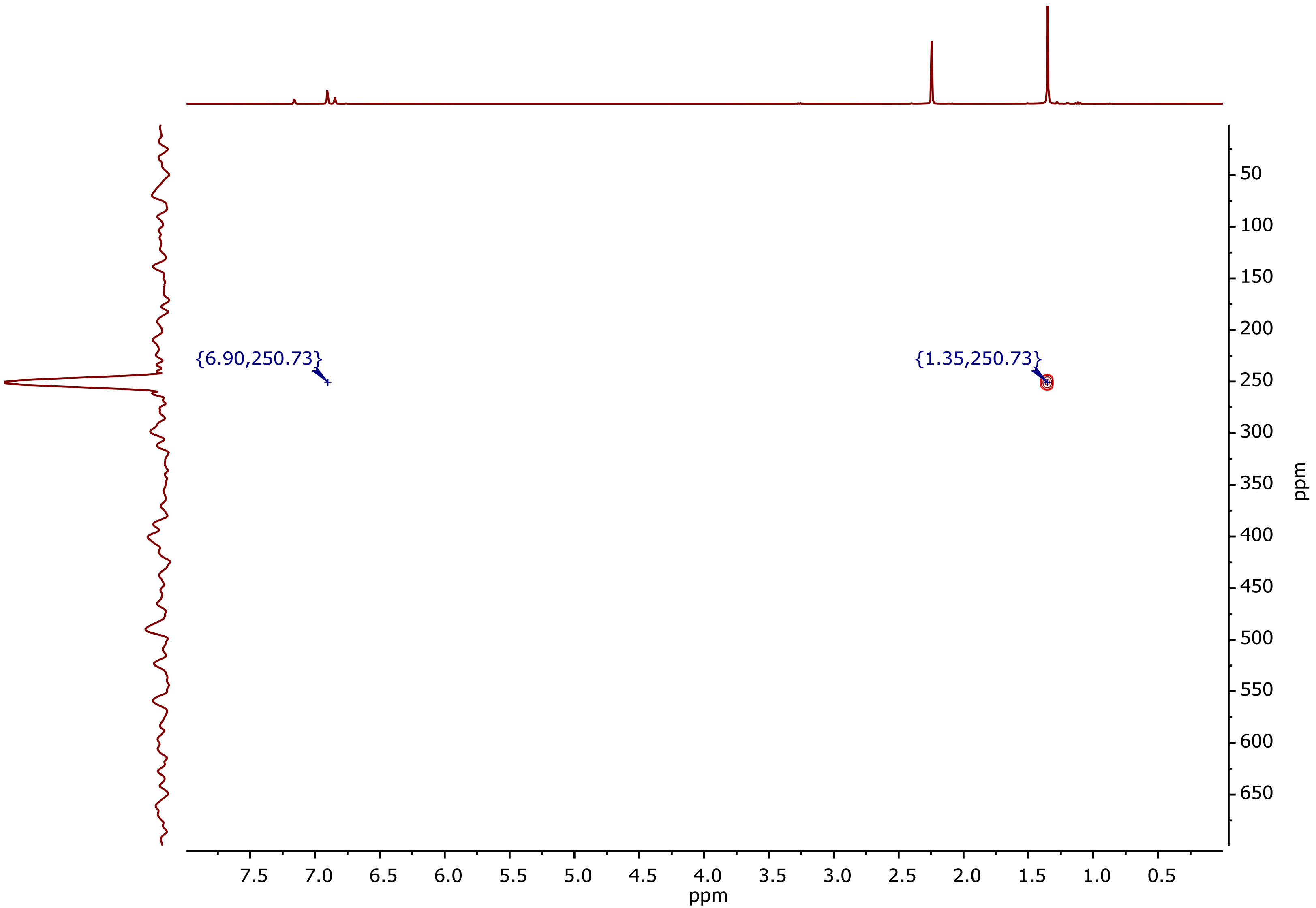

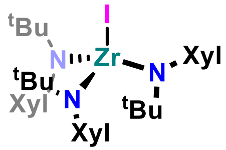

*Figure S 12: ^{1}H-^{15}N HMBC NMR spectrum of **1-I** in C_6D_6 at 298 K.*

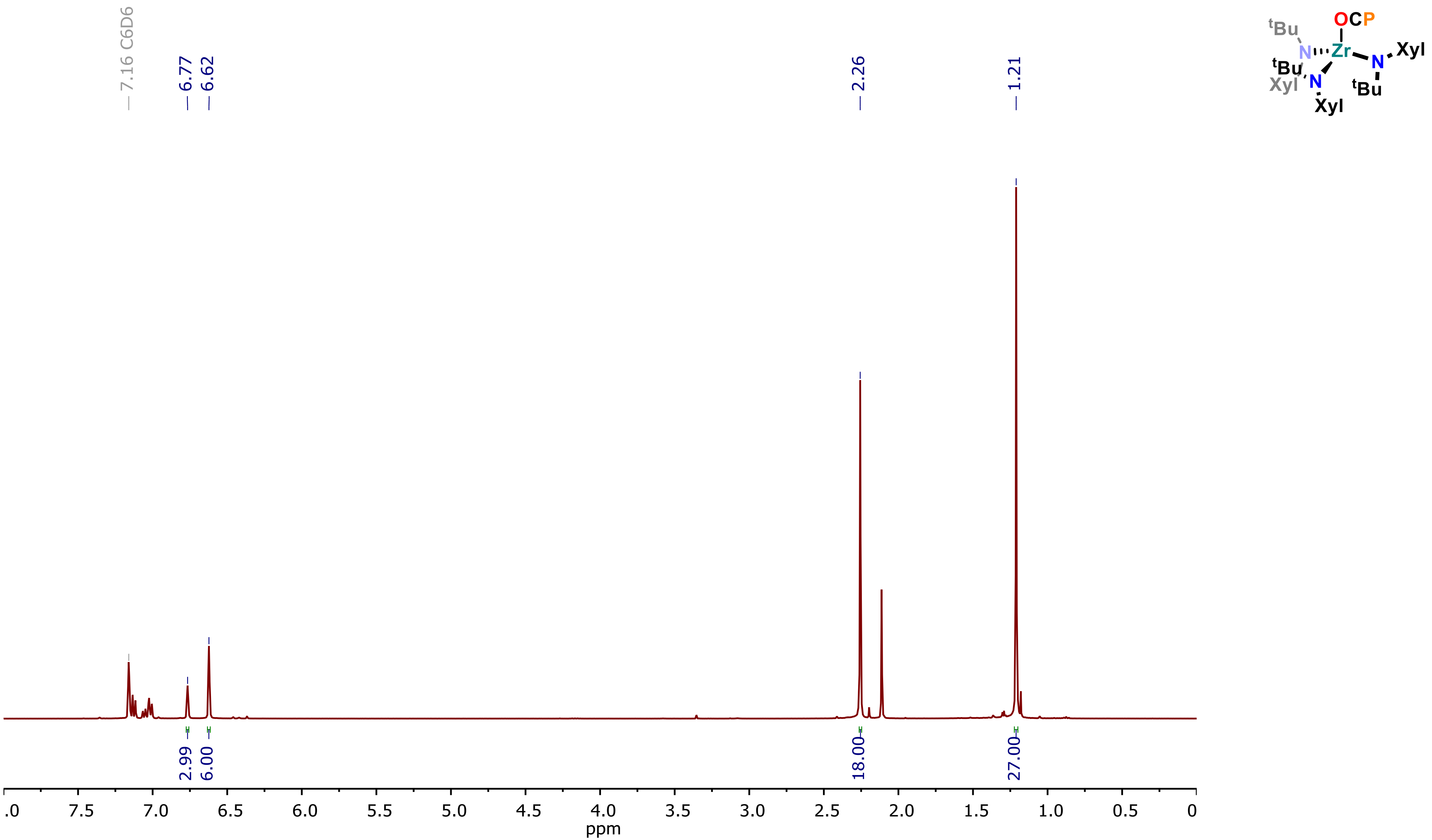

*Figure S 13: ^{1}H NMR spectrum of **1-OCP** in C_6D_6 at 298 K.*

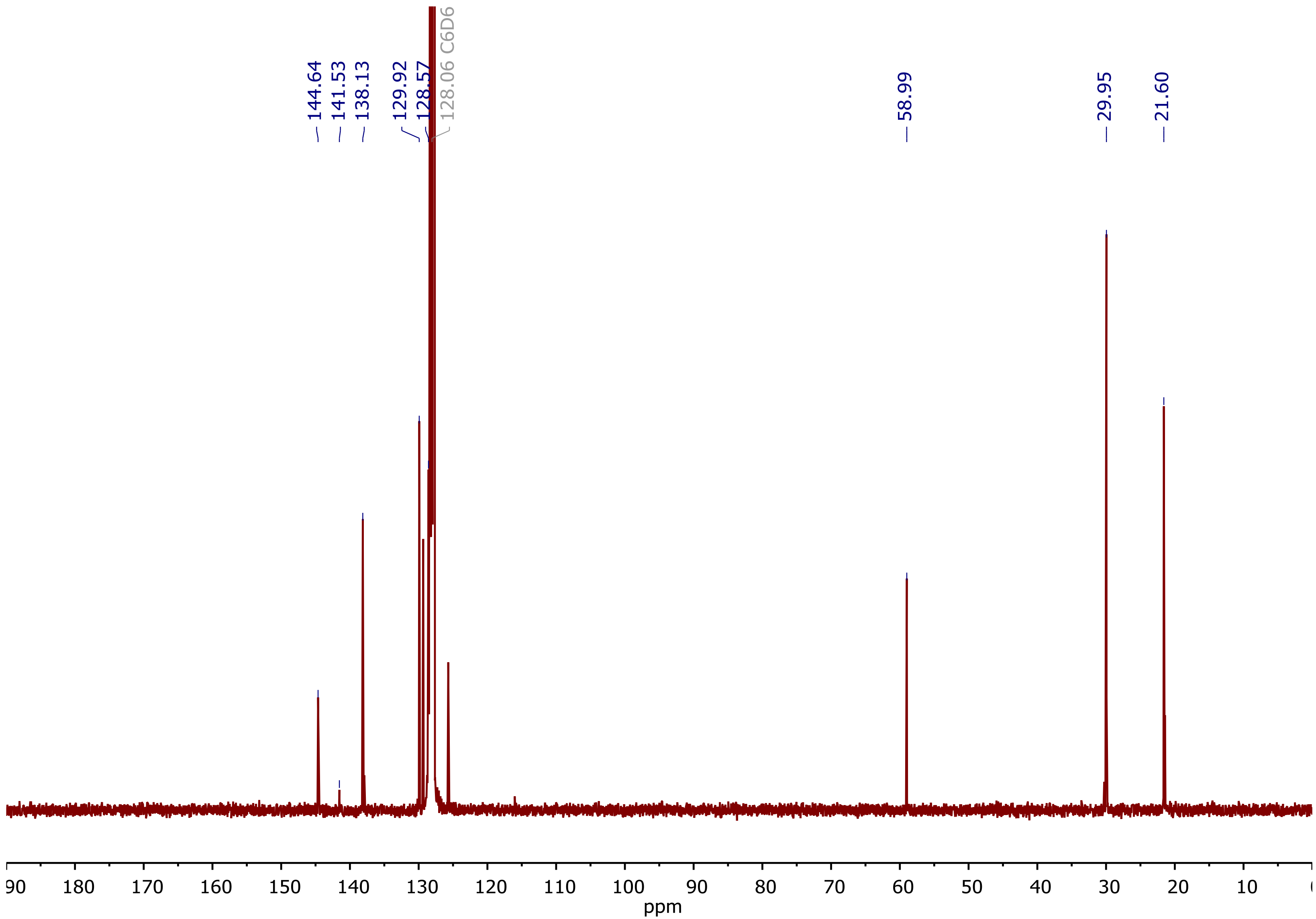

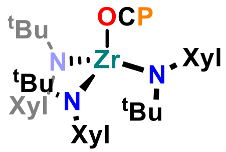

*Figure S 14: ${}^{13}C\{{}^{1}H\}$ NMR spectrum of **1-OCP** in C_6D_6 at 298 K.*

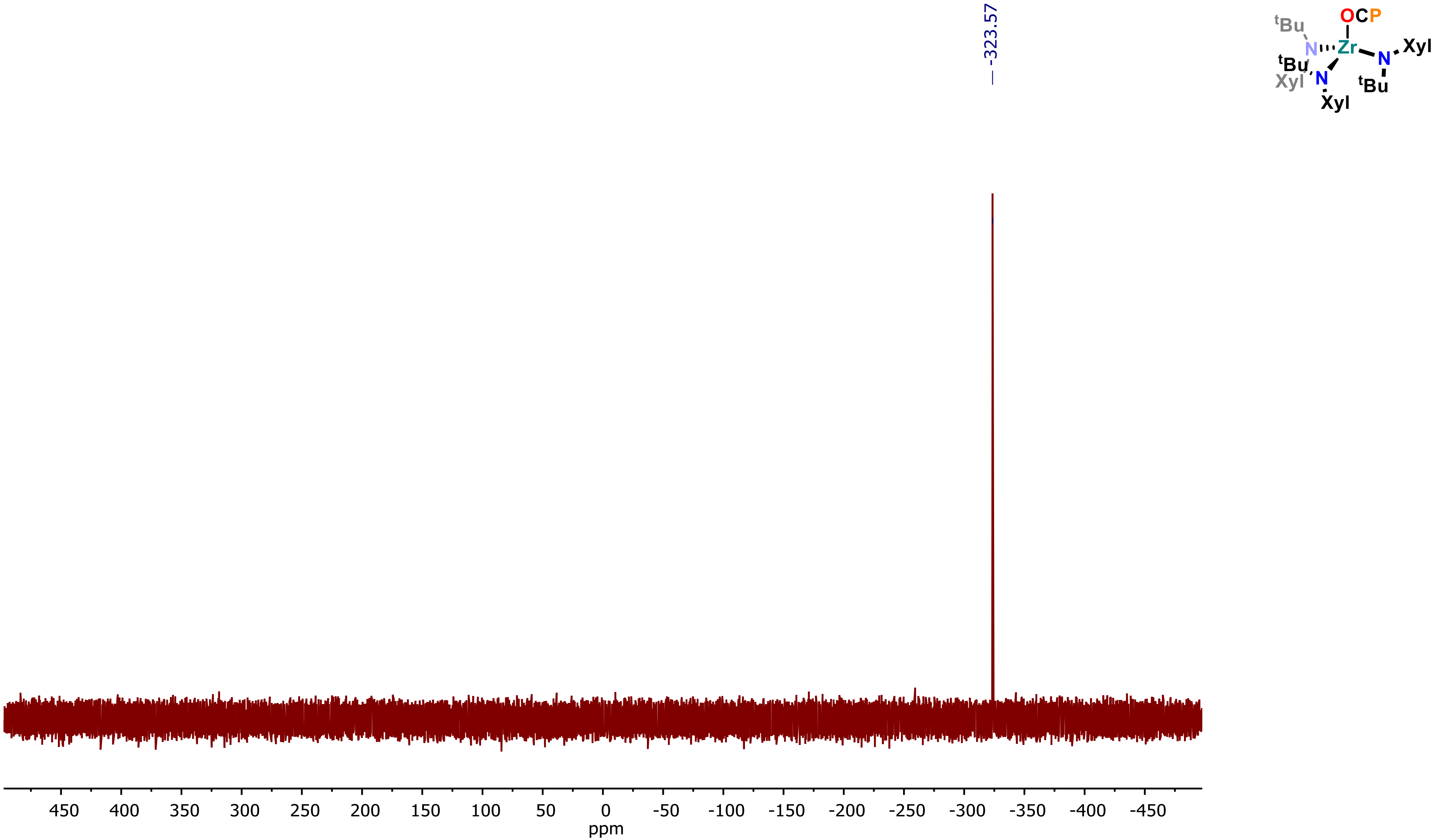

*Figure S 15: $^{31}P\{^{1}H\}$ NMR spectrum of **1-OCP** in C_6D_6 at 298 K.*

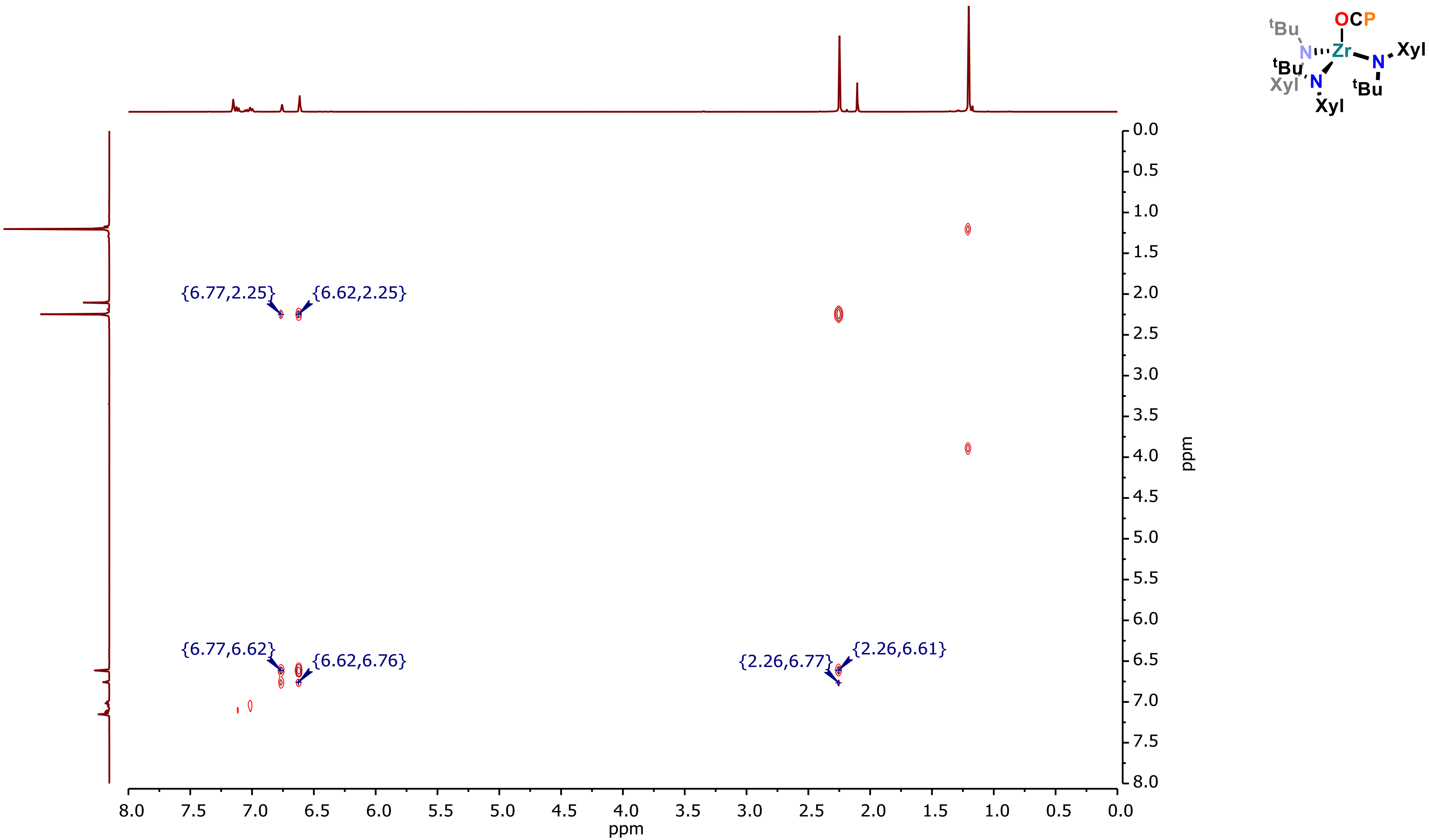

*Figure S 16: ^{1}H-^{1}H COSY NMR spectrum of **1-OCP** in C_6D_6 at 298 K.*

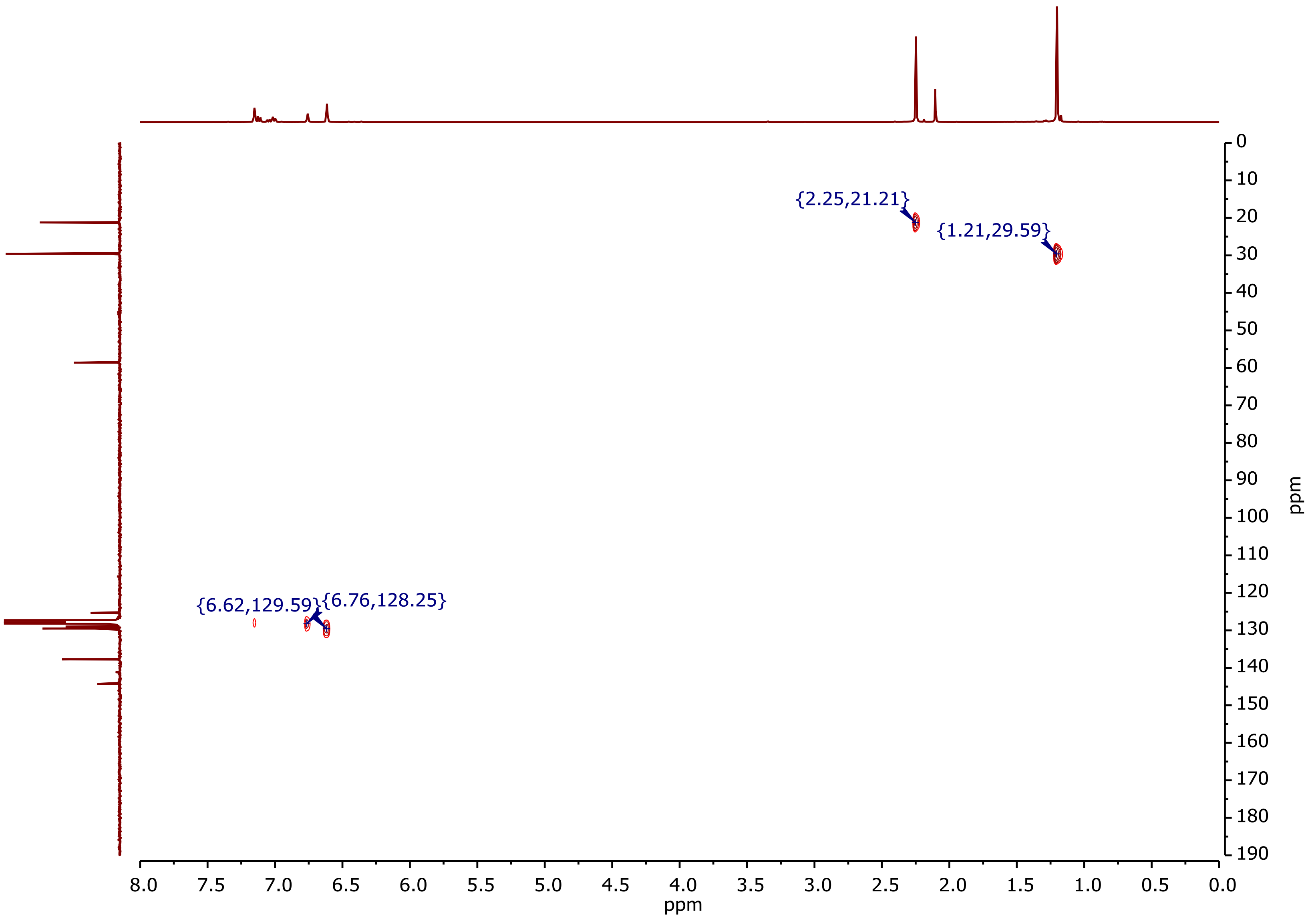

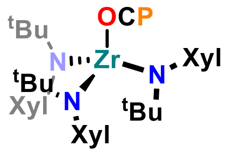

*Figure S 17: ^{1}H-^{13}C HSQC NMR spectrum of **1-OCP** in C_6D_6 at 298 K.*

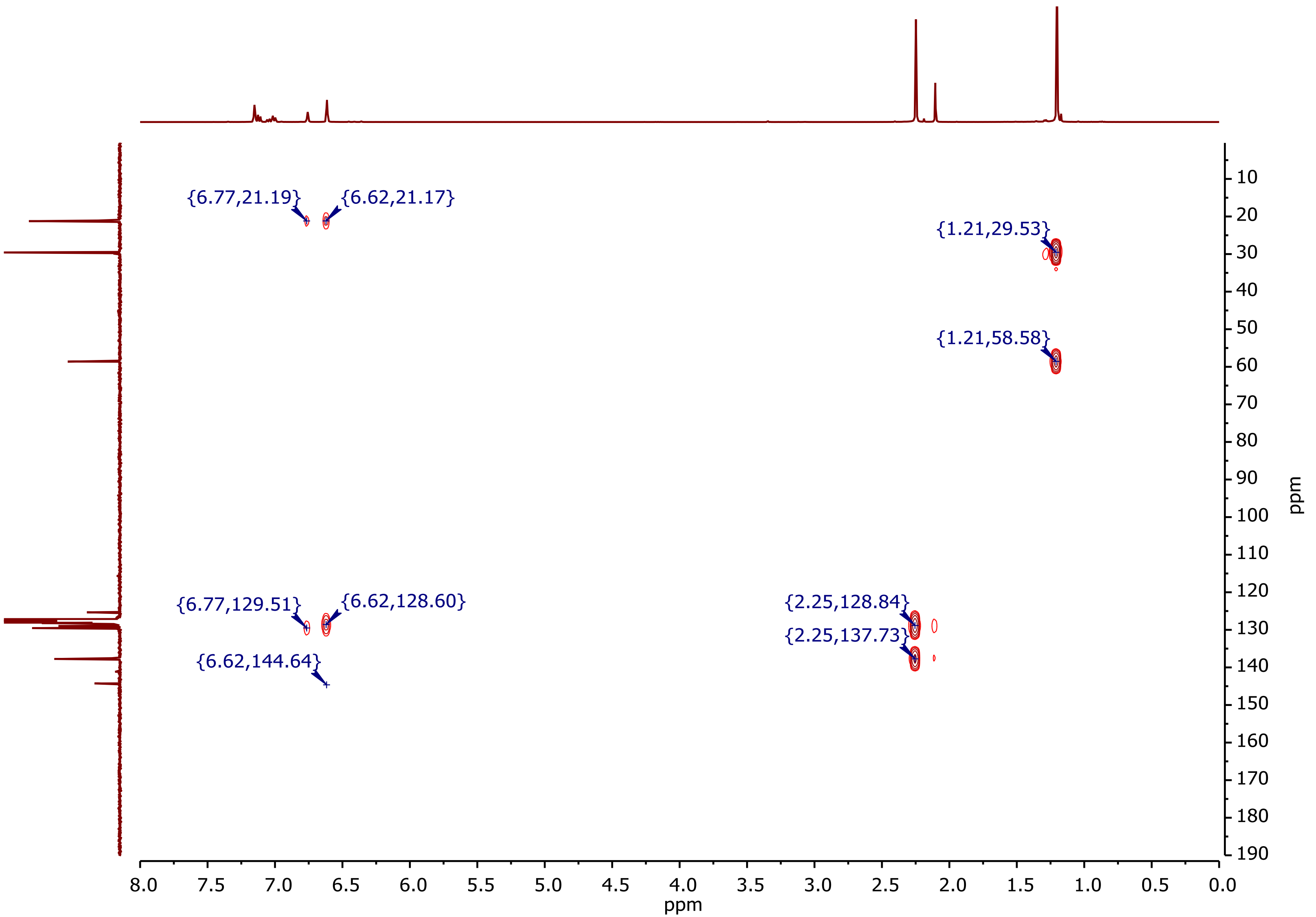

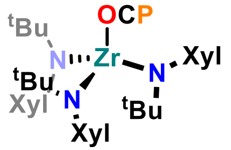

*Figure S 18: ^{1}H-^{13}C HMBC NMR spectrum of **1-OCP** in C_6D_6 at 298 K.*

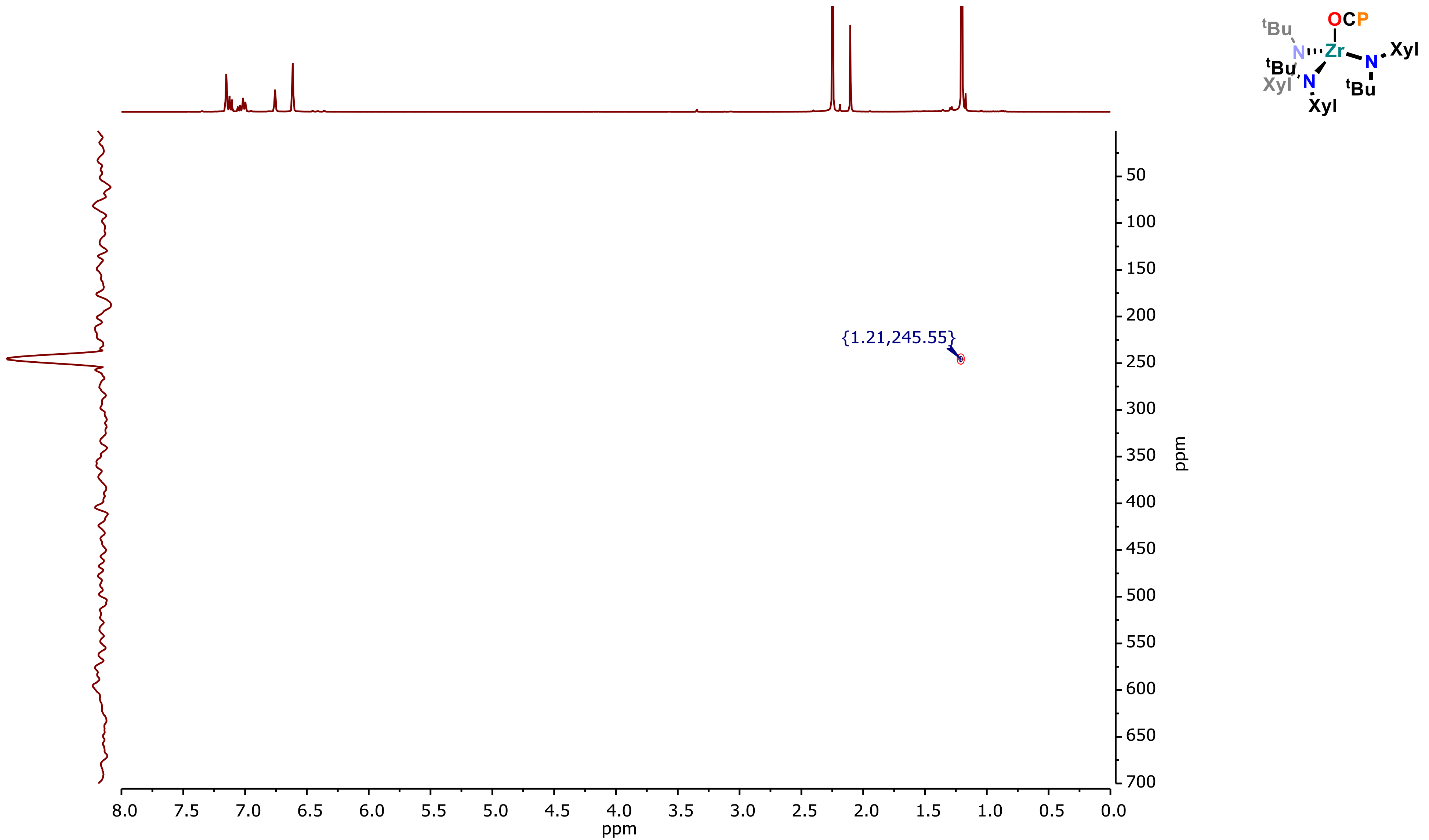

*Figure S 19: ^{1}H-^{15}N HMBC NMR spectrum of **1-OCP** in C_6D_6 at 298 K.*

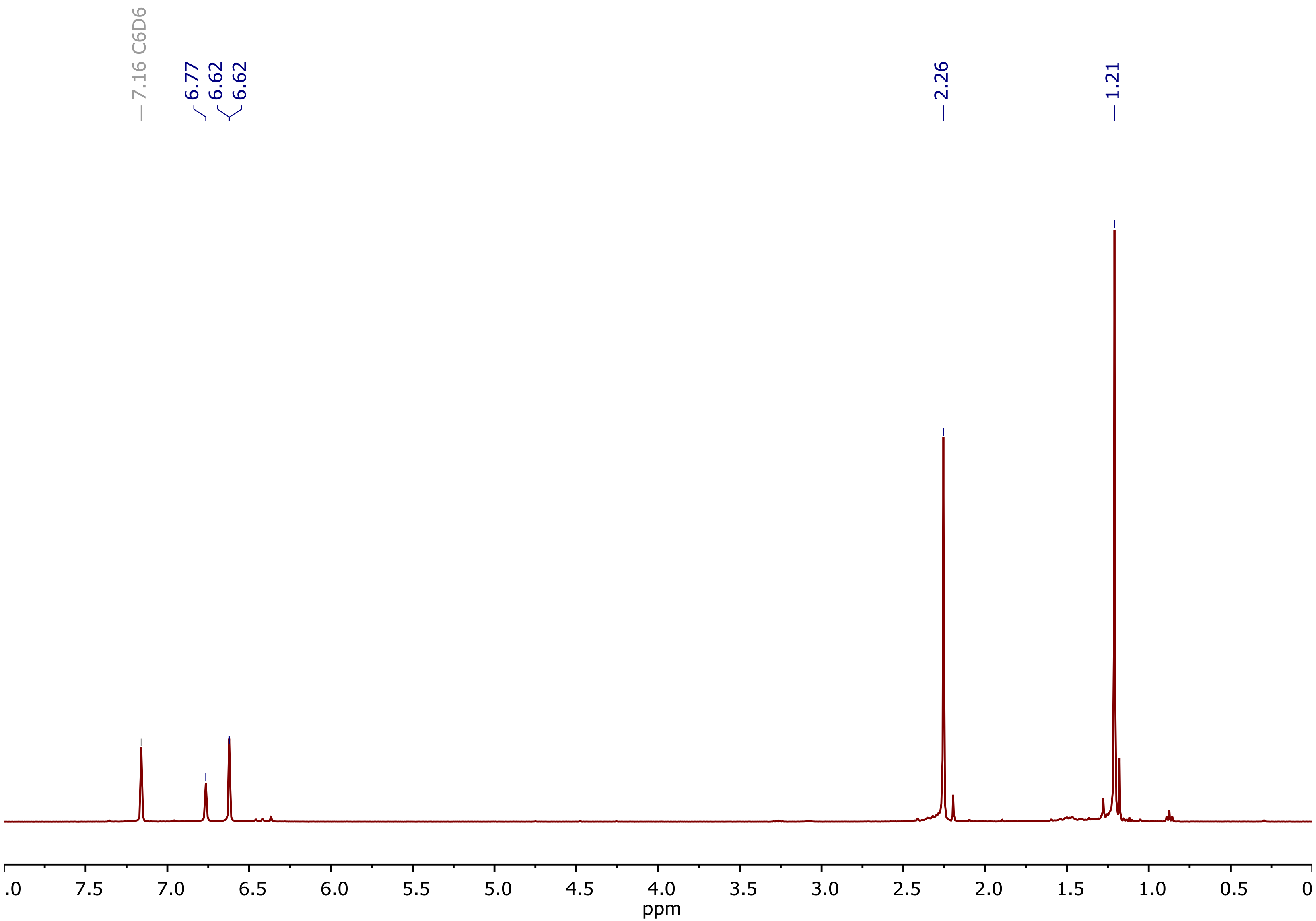

*Figure S 20: ^{1}H NMR spectrum after heating **1-OCP** for 24 h to 100 °C, in C_6D_6 at 298 K.*

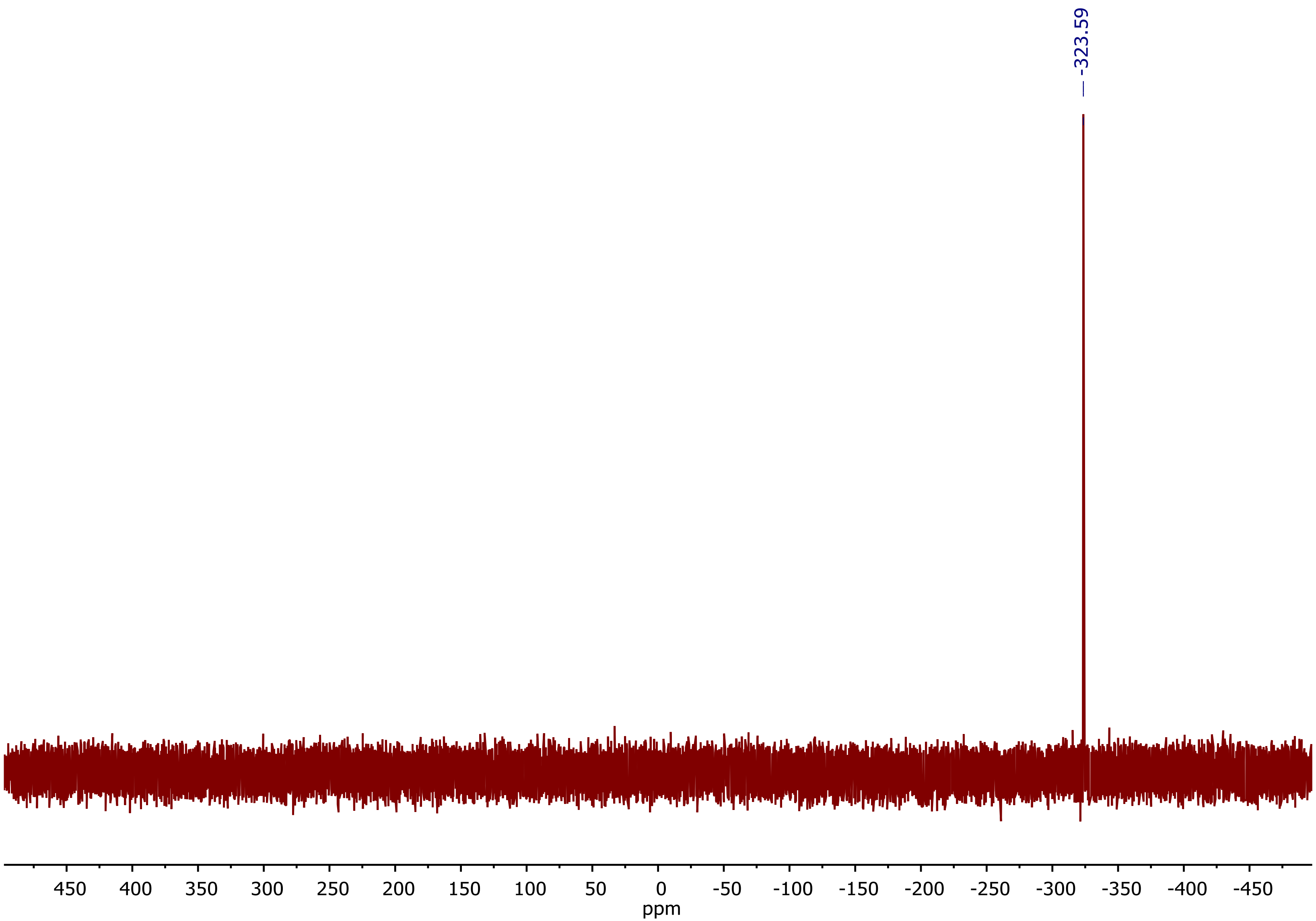

*Figure S 21: $^{31}P\{^{1}H\}$ NMR spectrum after heating **1-OCP** for 24 h to 100 °C, in C_6D_6 at 298 K.*

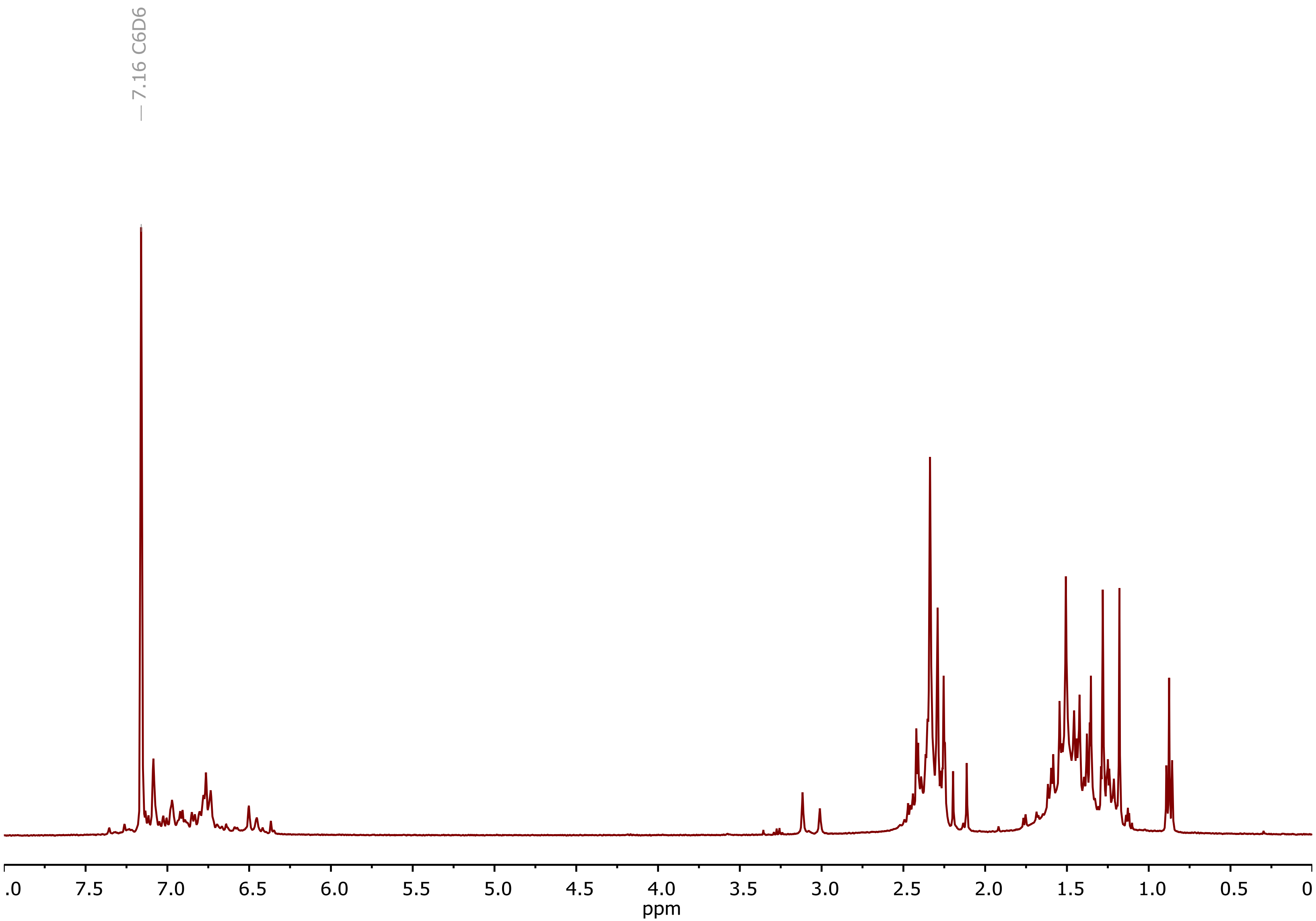

*Figure S 22: Complex ^{1}H NMR spectrum after treating **1-I** with [Na(Diox)$_3$][OCAs] at ambient temperature in toluene. Spectrum of crude product in C_6D_6 at 298 K.*

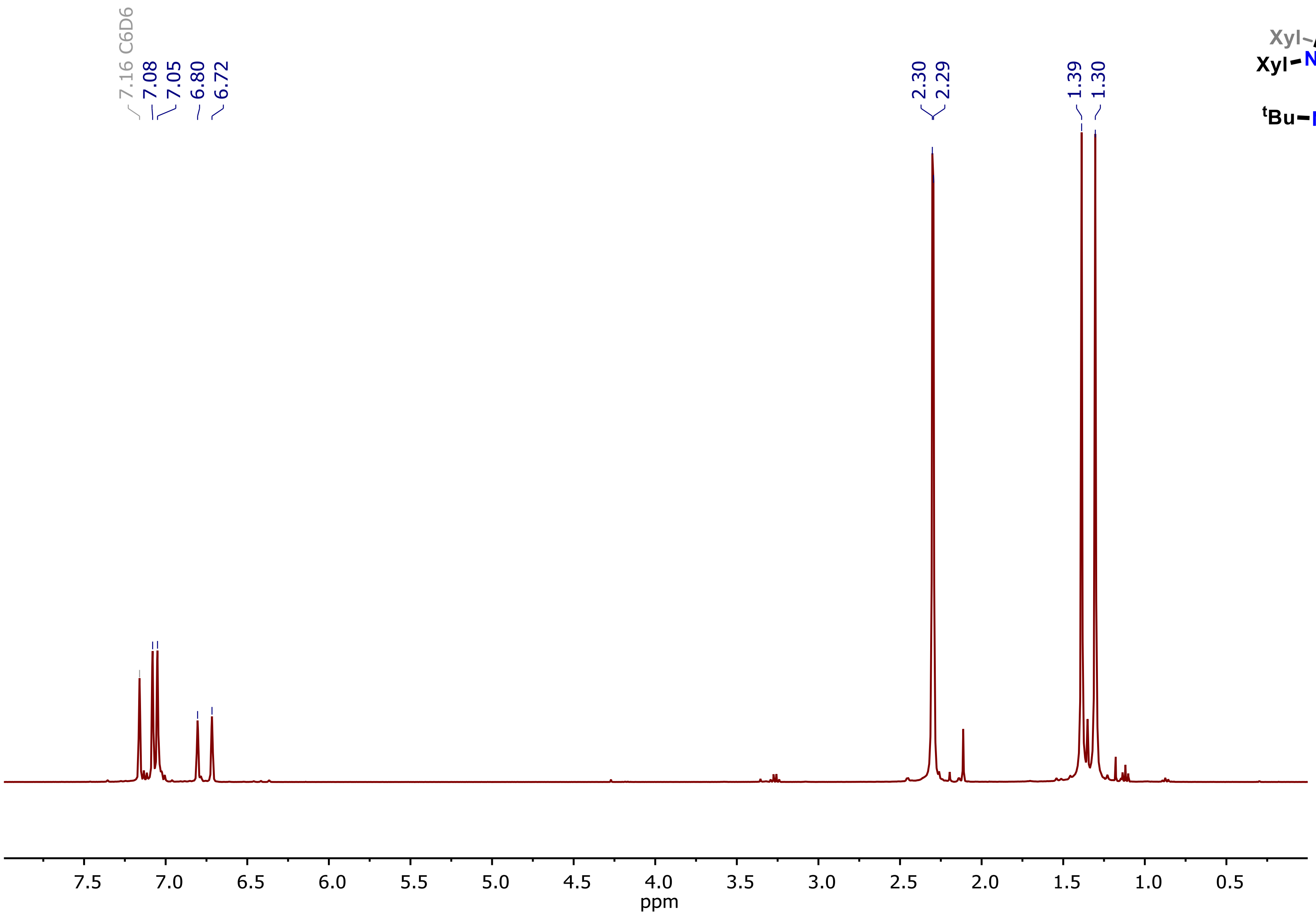

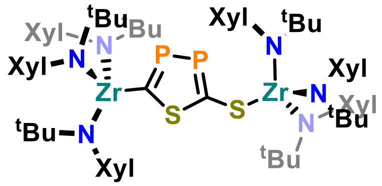

*Figure S 23: ^{1}H NMR spectrum of **2-PP** in C_6D_6 at 298 K.*

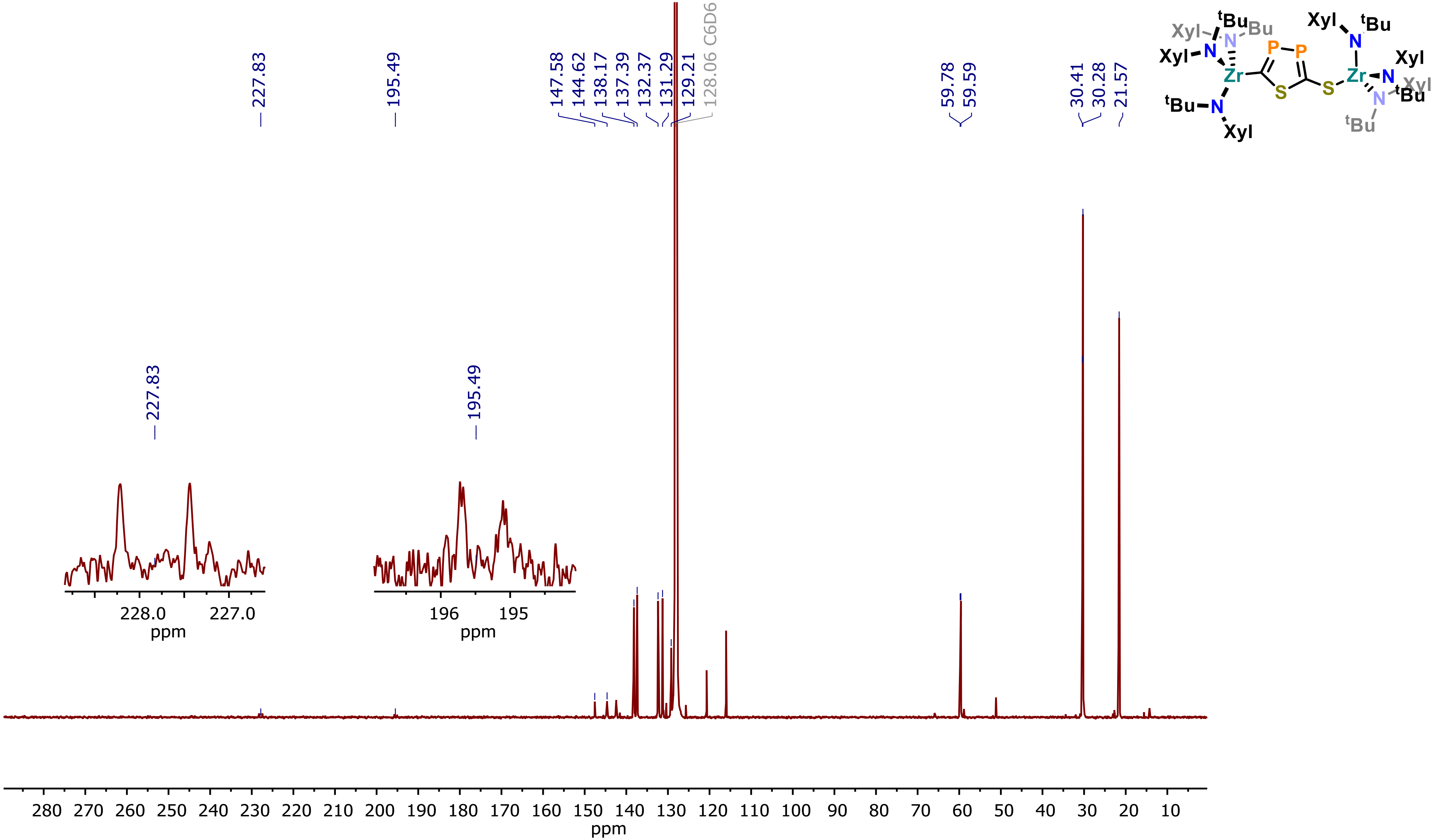

Figure S 24: $^{13}C\{^1H\}$ NMR spectrum of ***2-PP*** *in C_6D_6 at 298 K. The inlets show the enlarged sections 228.8 – 226.5 and 197 – 194 ppm for better visibility of both signals related to the heterocyclic carbon atoms. While a $^2J_{CP}$ coupling can be observed for the S-bound carbon of the heterocycle, it is not visible for the Zr-bound carbon.*

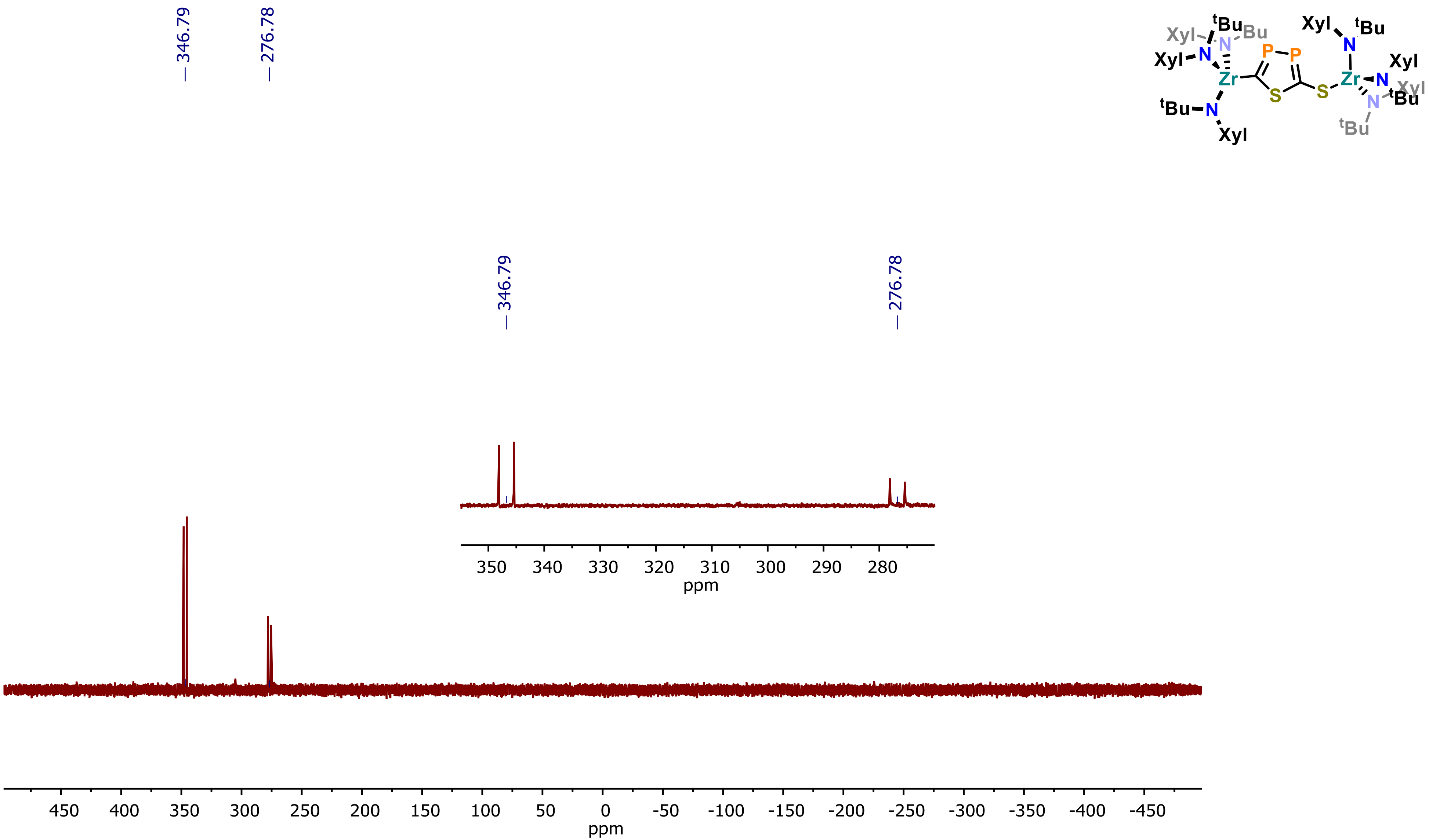

*Figure S 25: $^{31}P\{^1H\}$ spectrum of **2-PP** in C_6D_6 at 298 K. The inlet shows the enlarged section 355 –270 ppm for better visibility of both doublets related to the heterocyclic phosphorus atoms.*

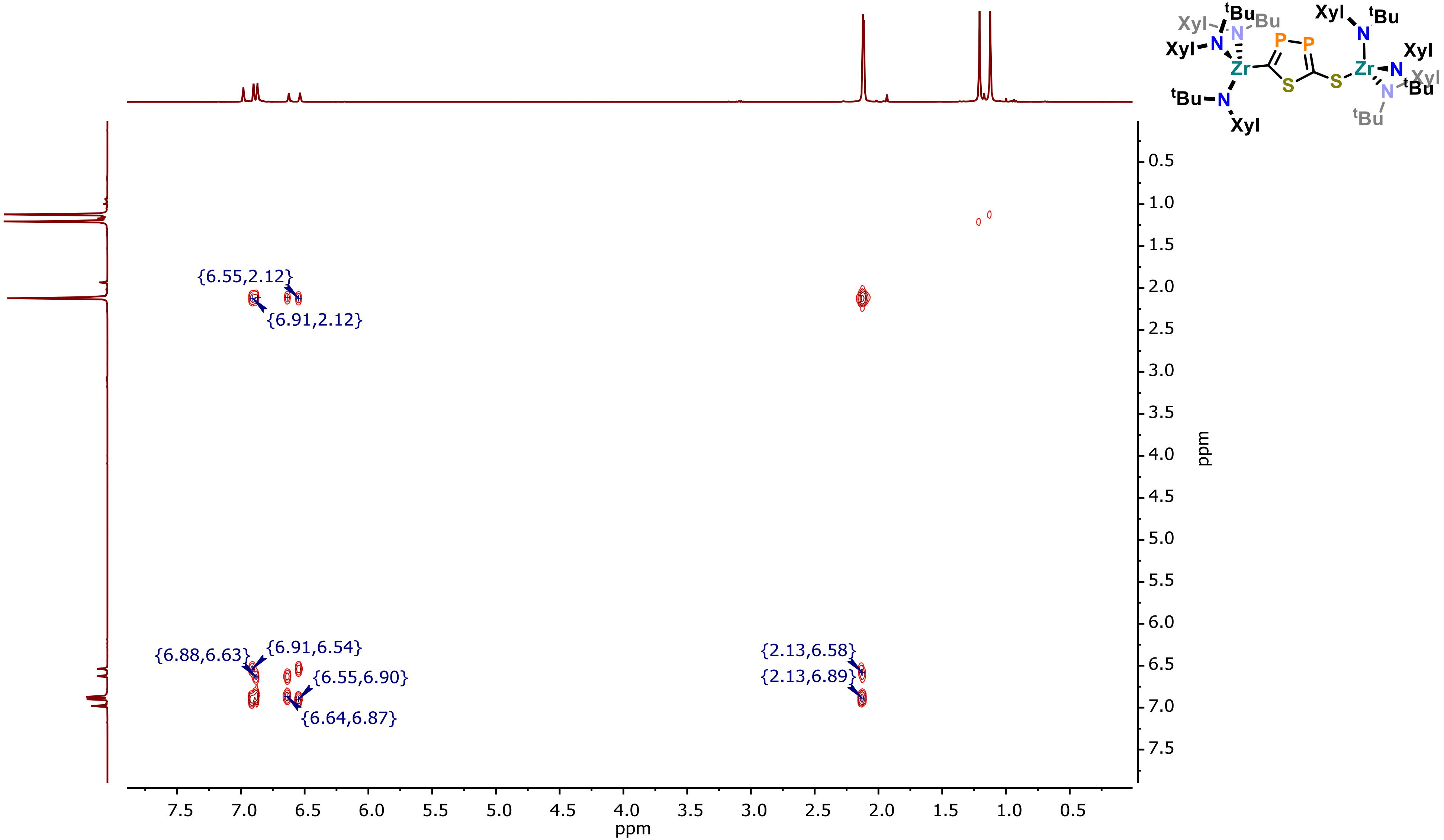

*Figure S 26: ^{1}H-^{1}H COSY NMR spectrum of **2-PP** in C_6D_6 at 298 K.*

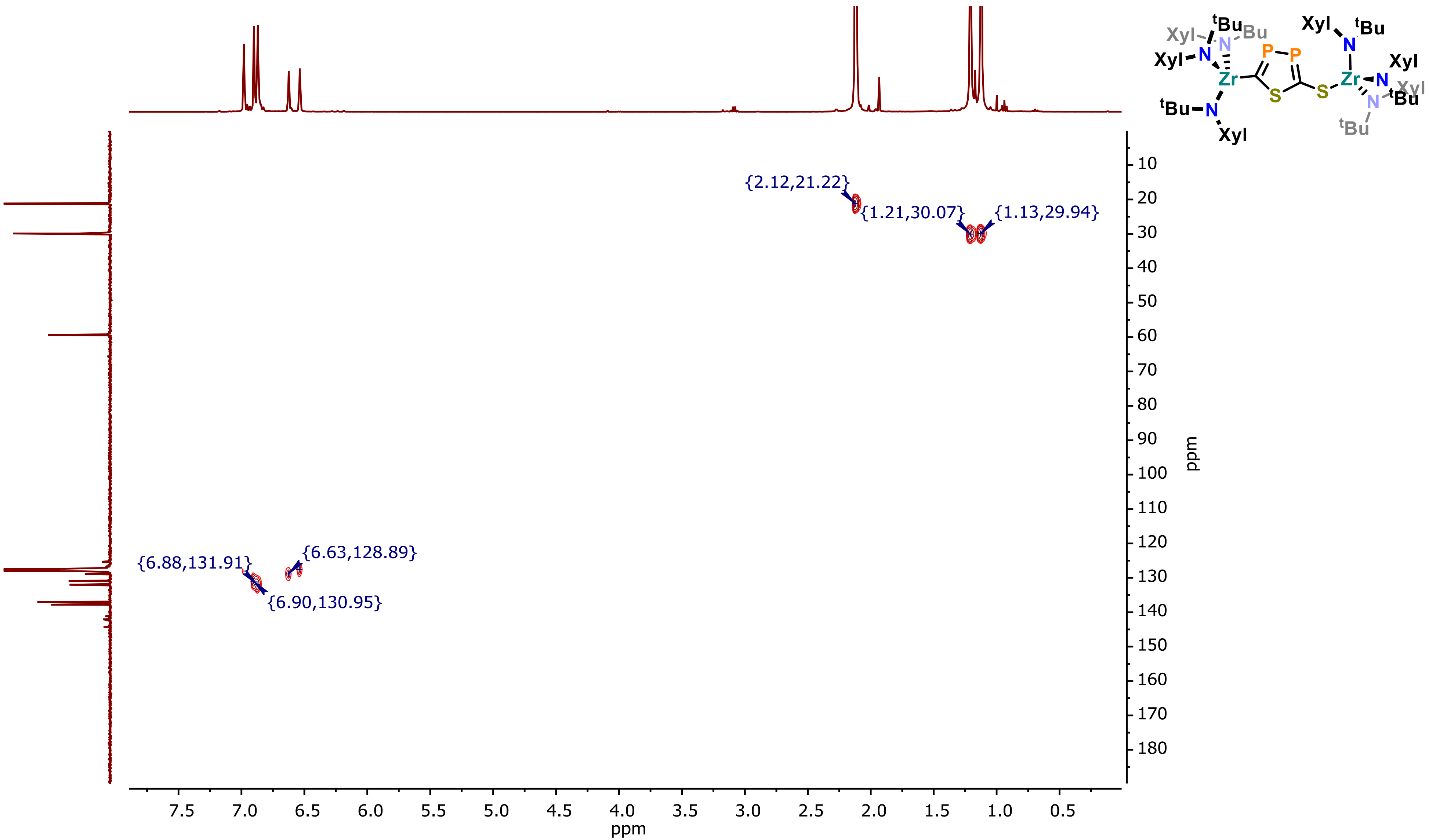

*Figure S 27: ^{1}H-^{13}C HSQC NMR spectrum of **2-PP** in C_6D_6 at 298 K.*

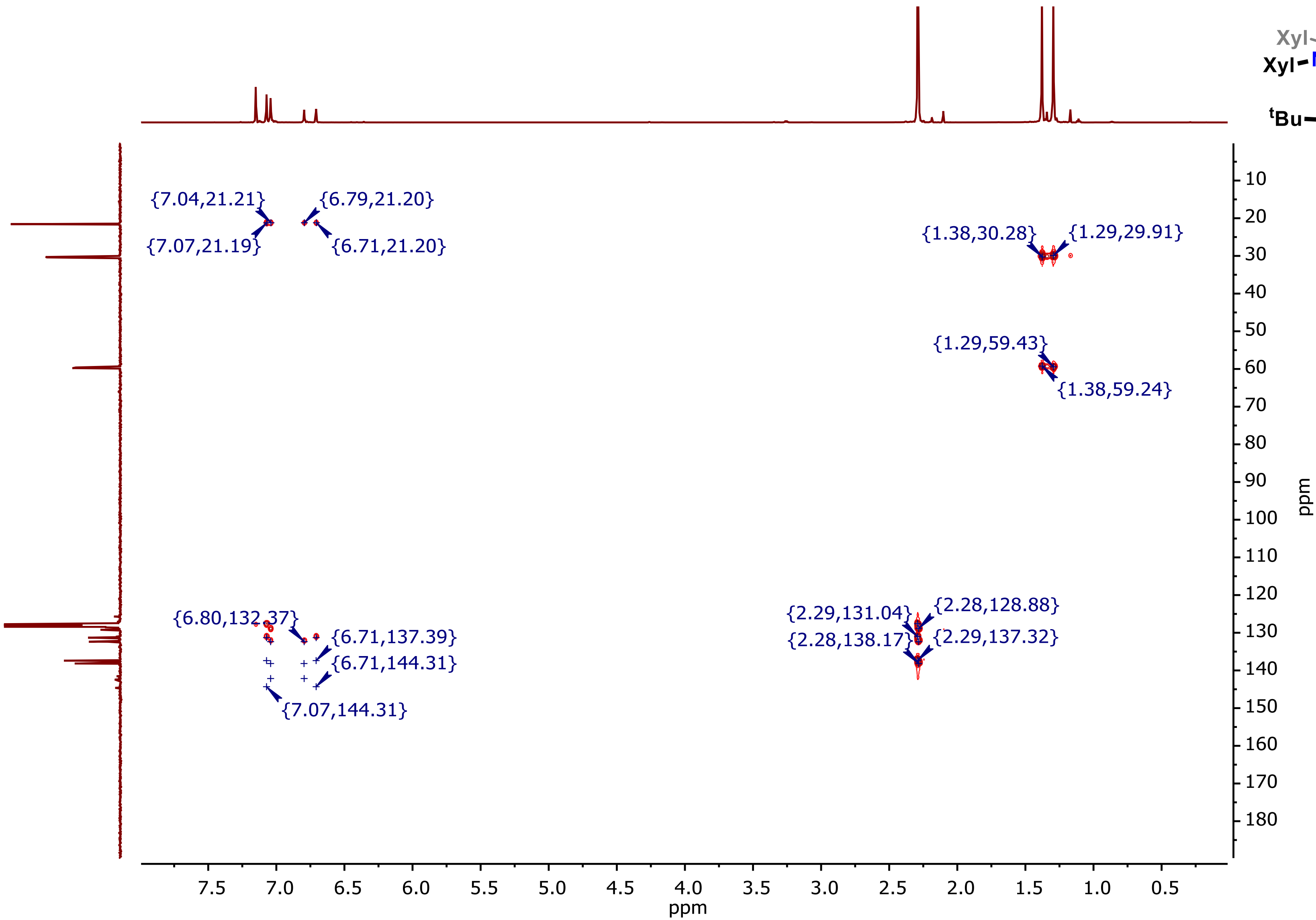

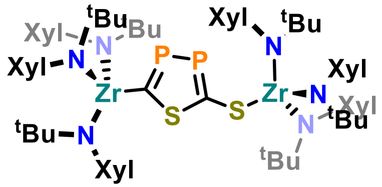

Figure S 28: ^{1}H-^{13}C HMBC NMR spectrum of **2-PP** in C_6D_6 at 298 K.

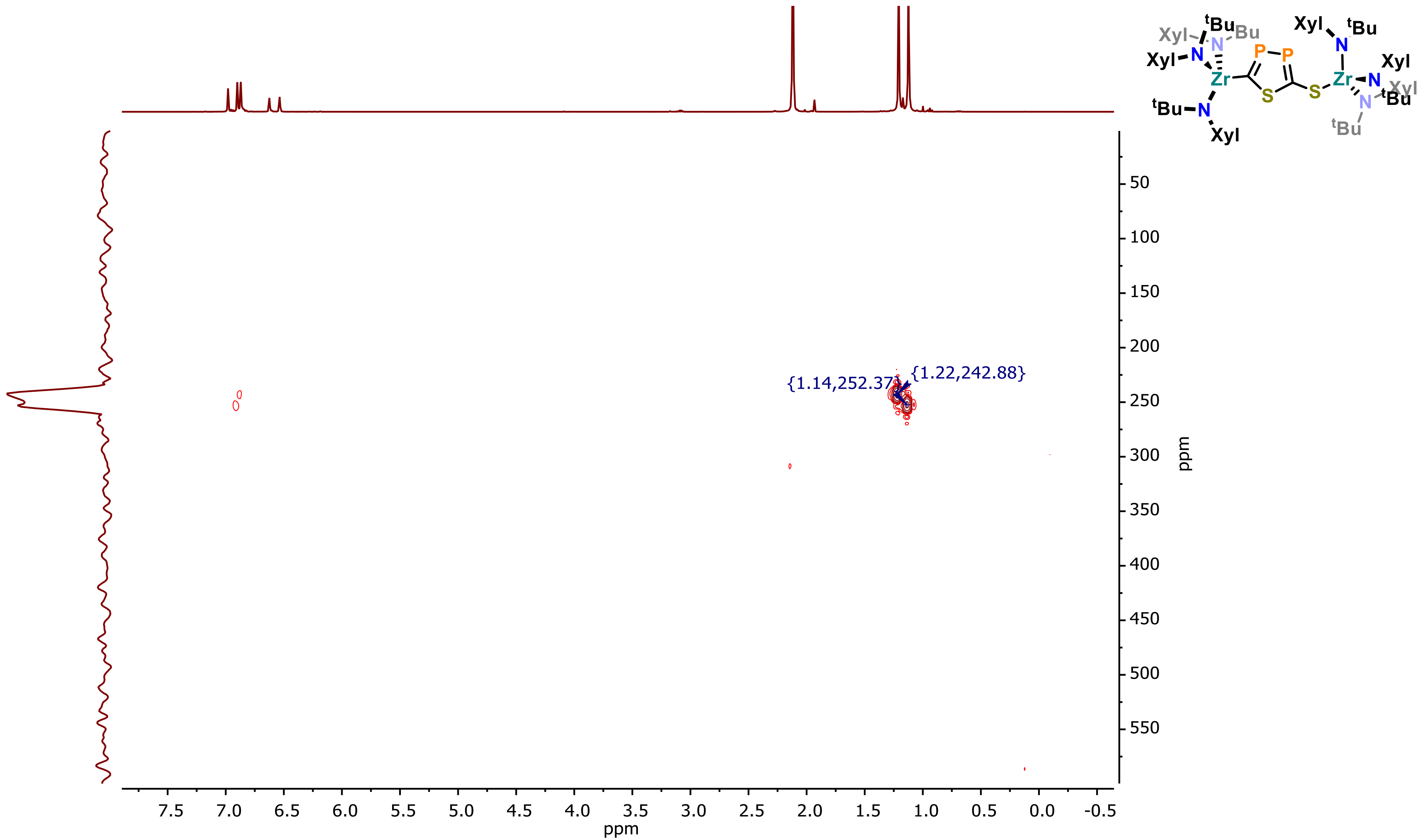

*Figure S 29: ^{1}H-^{15}N HMBC NMR spectrum of **2-PP** in C_6D_6 at 298 K.*

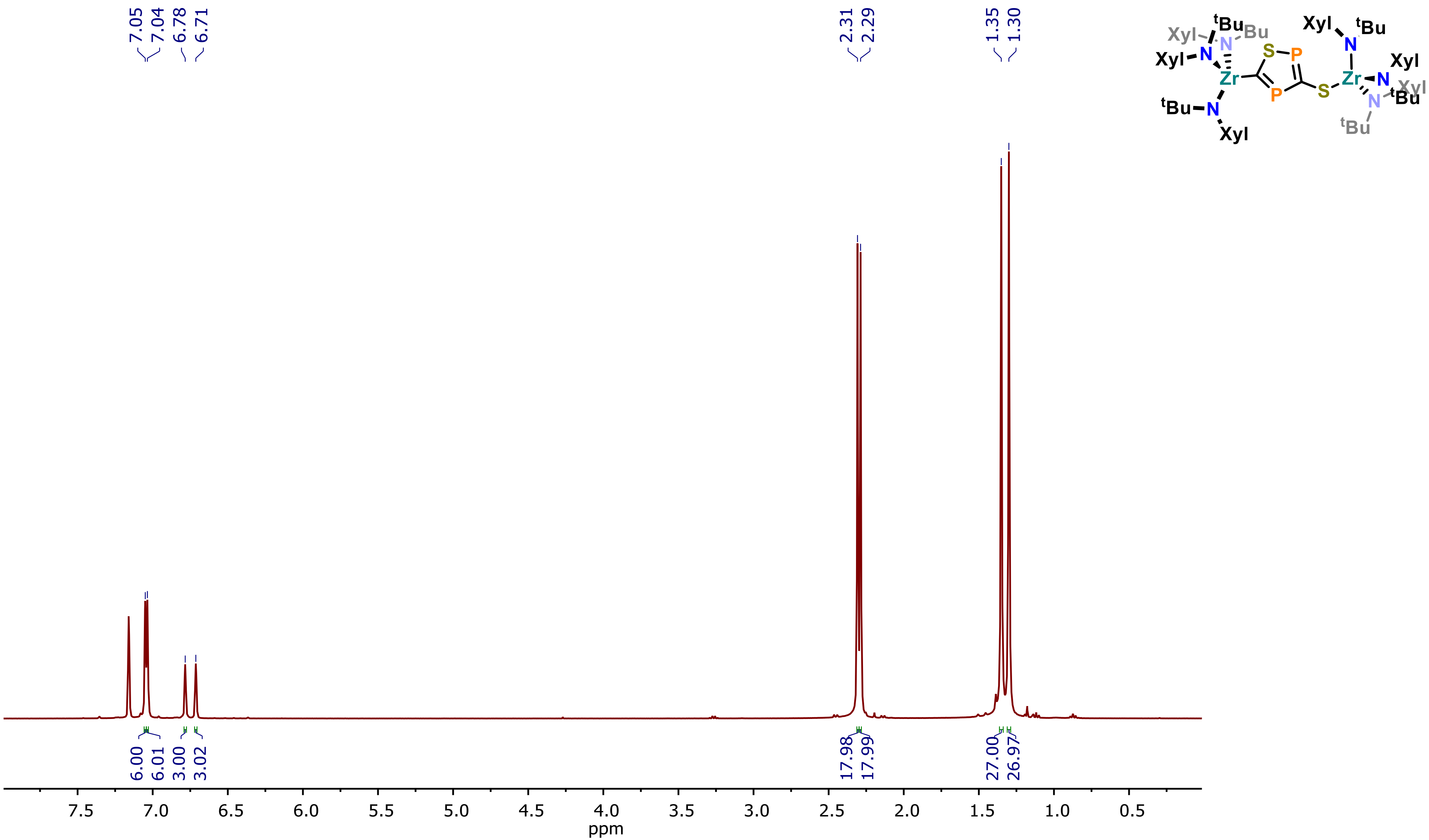

*Figure S 30: ^{1}H NMR spectrum of **2-SP** in C_6D_6 at 298 K.*

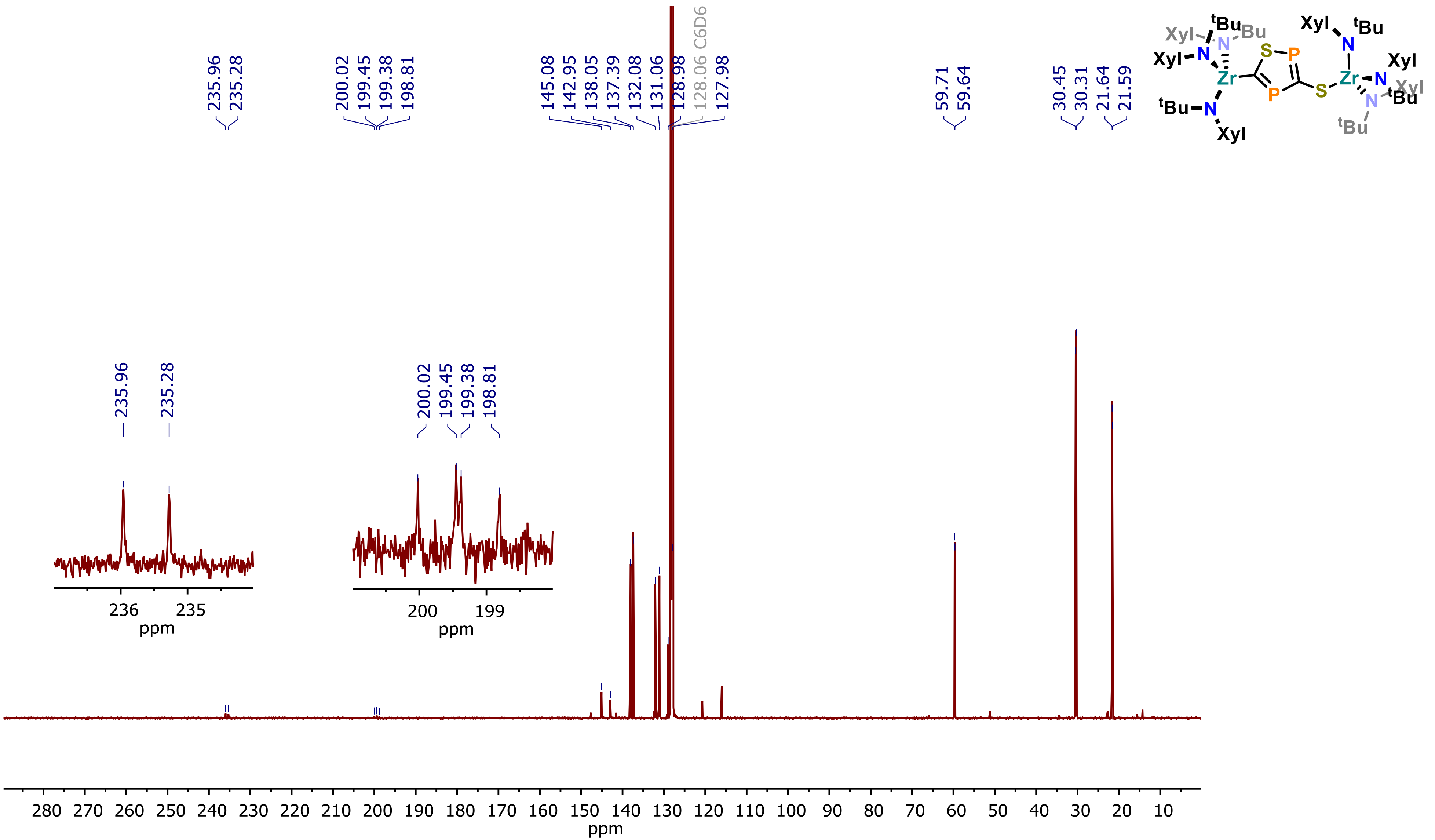

*Figure S 31: $^{13}C\{^{1}H\}$ NMR spectrum of **2-SP** in C_6D_6 at 298 K. The inlets show the enlarged section 237 - 234 ppm and 201 - 198 ppm for better visibility of the weak signals related to the heterocyclic carbon atoms. While the Zr-bound carbon atom shows only a doublet caused by a single $^{1}J_{CP}$ coupling, two adjacent phosphorus atoms cause the S-bound carbon atom to give rise to a doublet of doublets.*

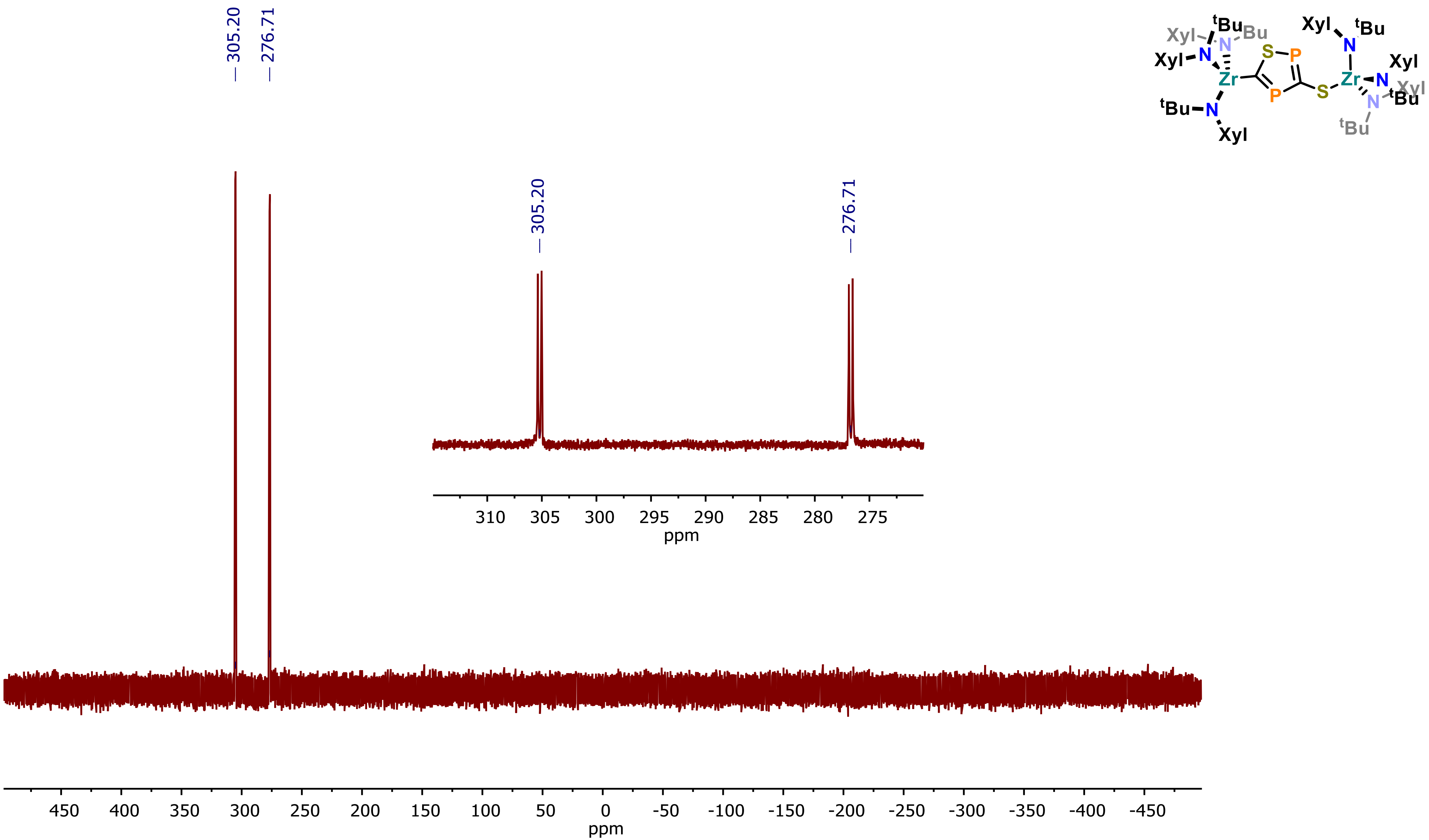

*Figure S 32: $^{31}P\{^{1}H\}$ NMR spectrum of **2-SP** in C_6D_6 at 298 K. The inlet shows the enlarged section 320 –270 ppm for better visibility of both doublets related to the heterocyclic phosphorus atoms.*

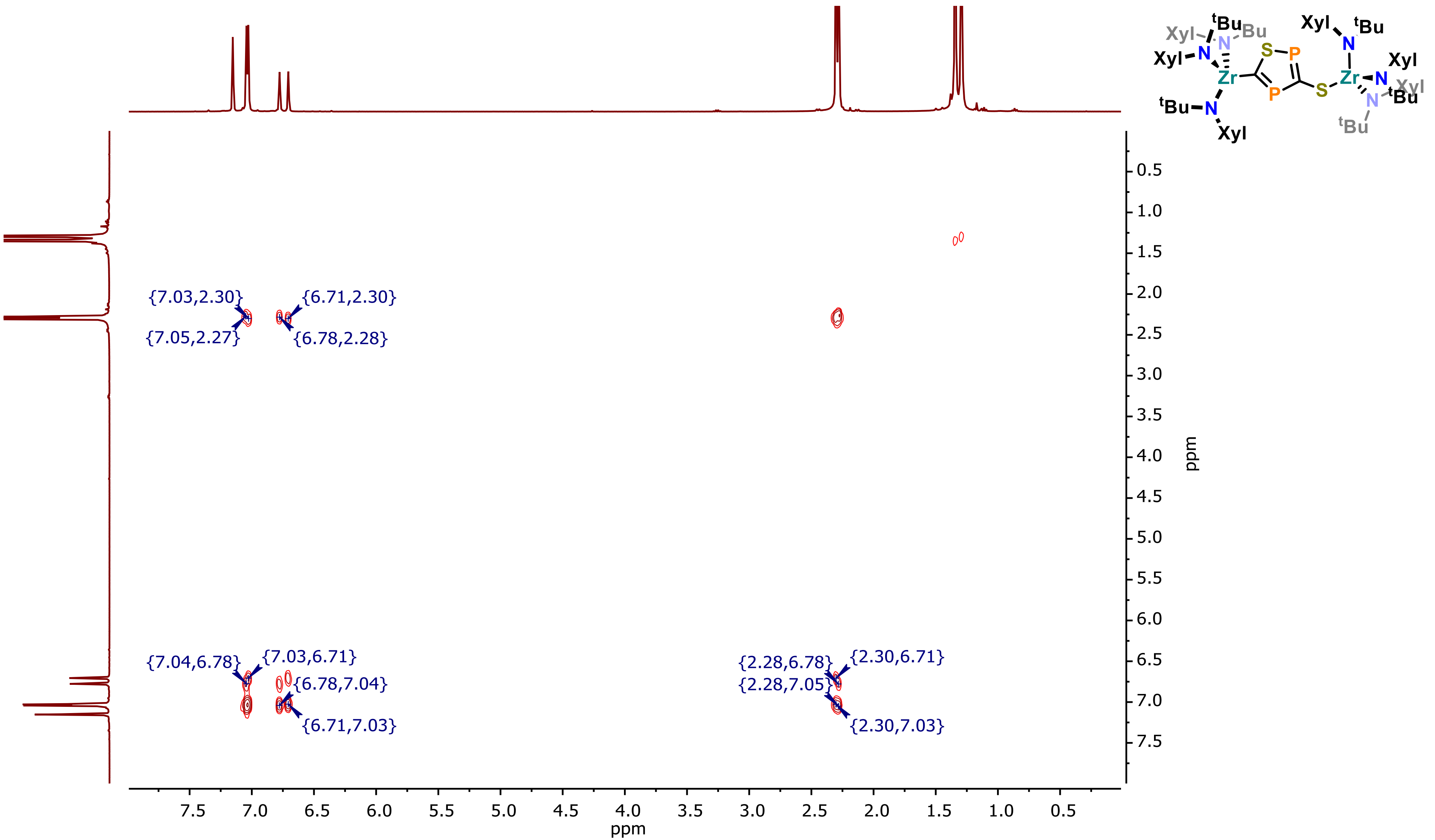

*Figure S 33: ^{1}H-^{1}H COSY NMR spectrum of **2-SP** in C_6D_6 at 298 K.*

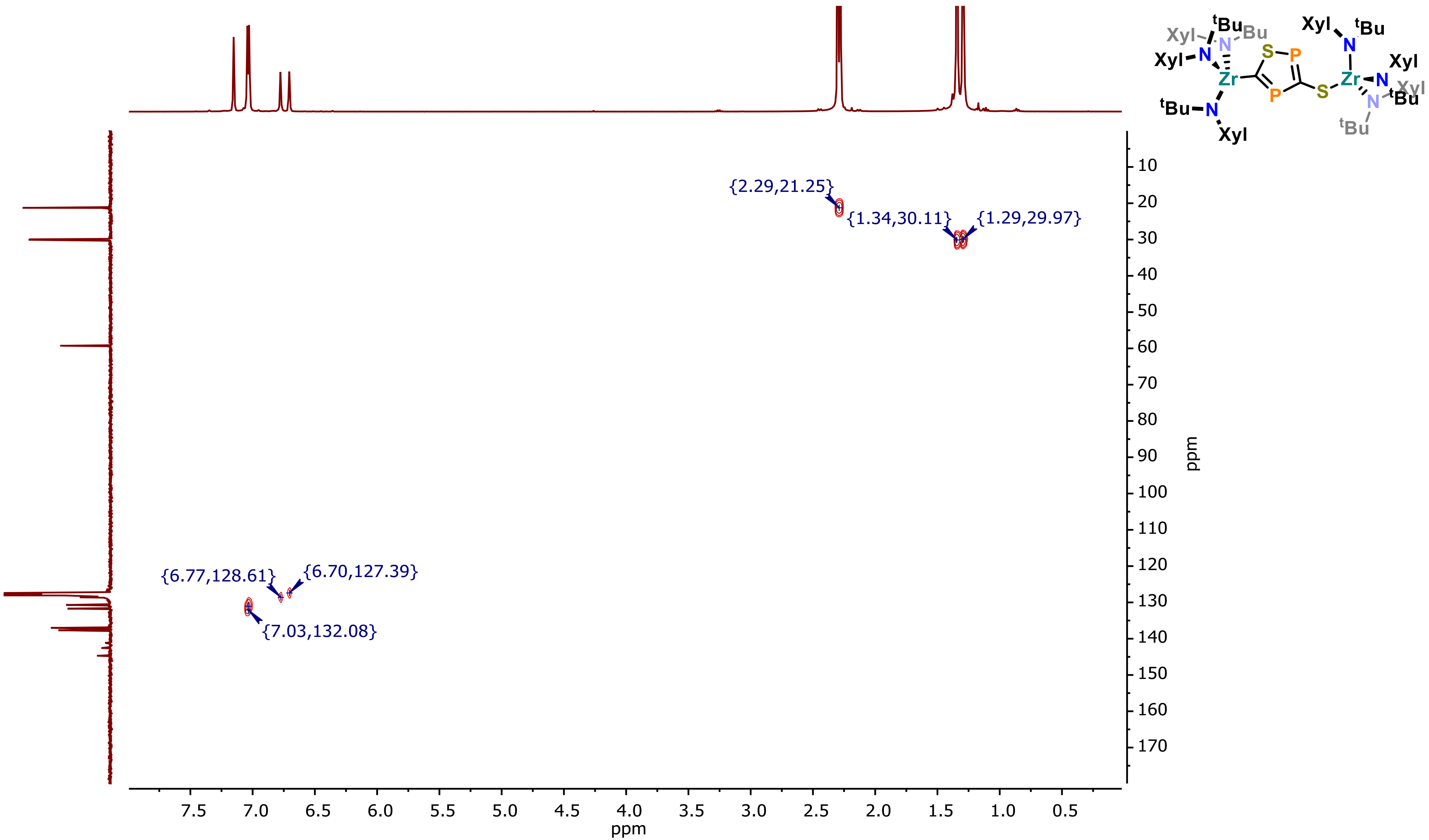

*Figure S 34: ^{1}H-^{13}C HSQC NMR spectrum of **2-SP** in C_6D_6 at 298 K.*

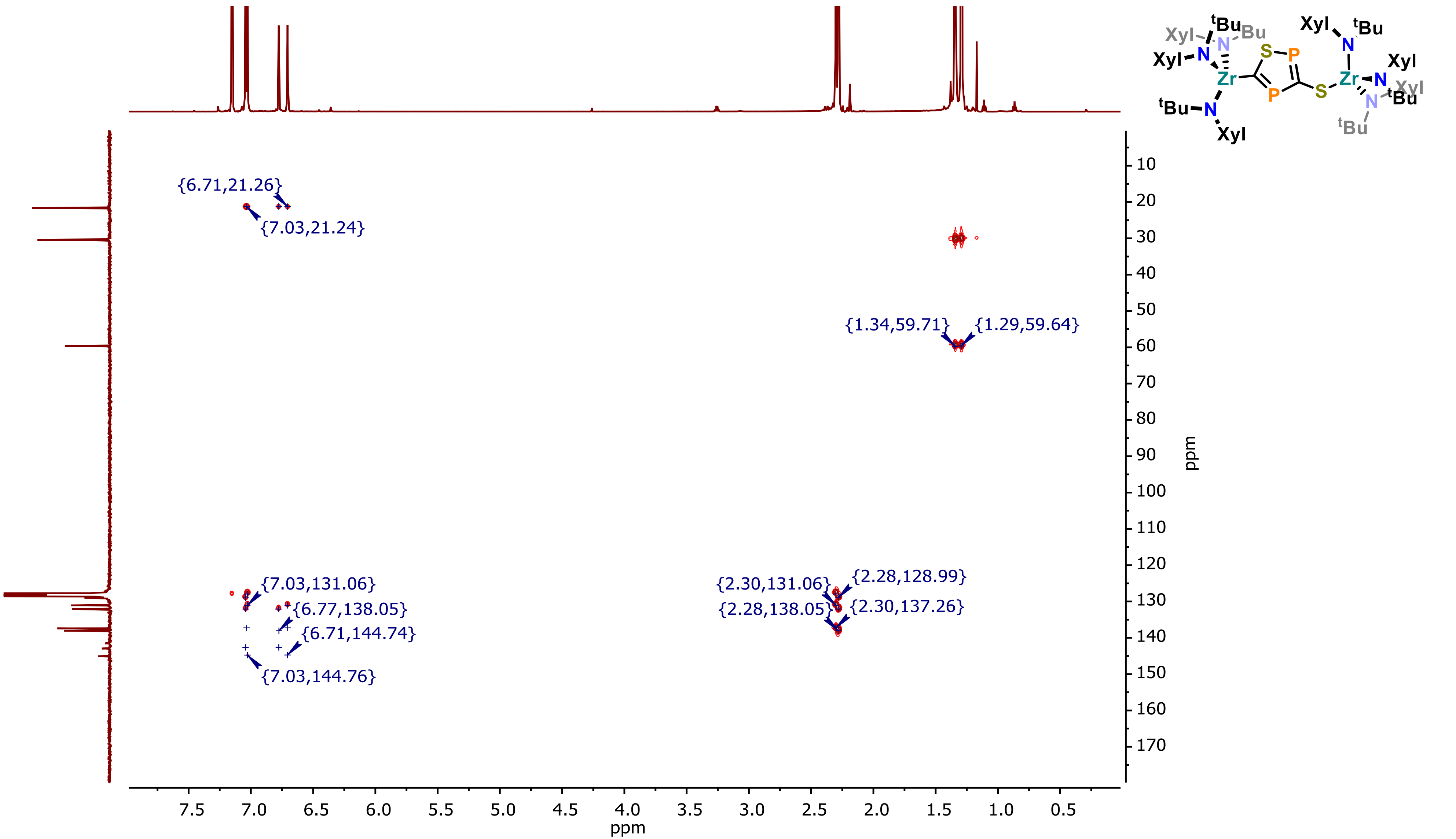

*Figure S 35: ^{1}H-^{13}C HMBC NMR spectrum of **2-SP** in C_6D_6 at 298 K.*

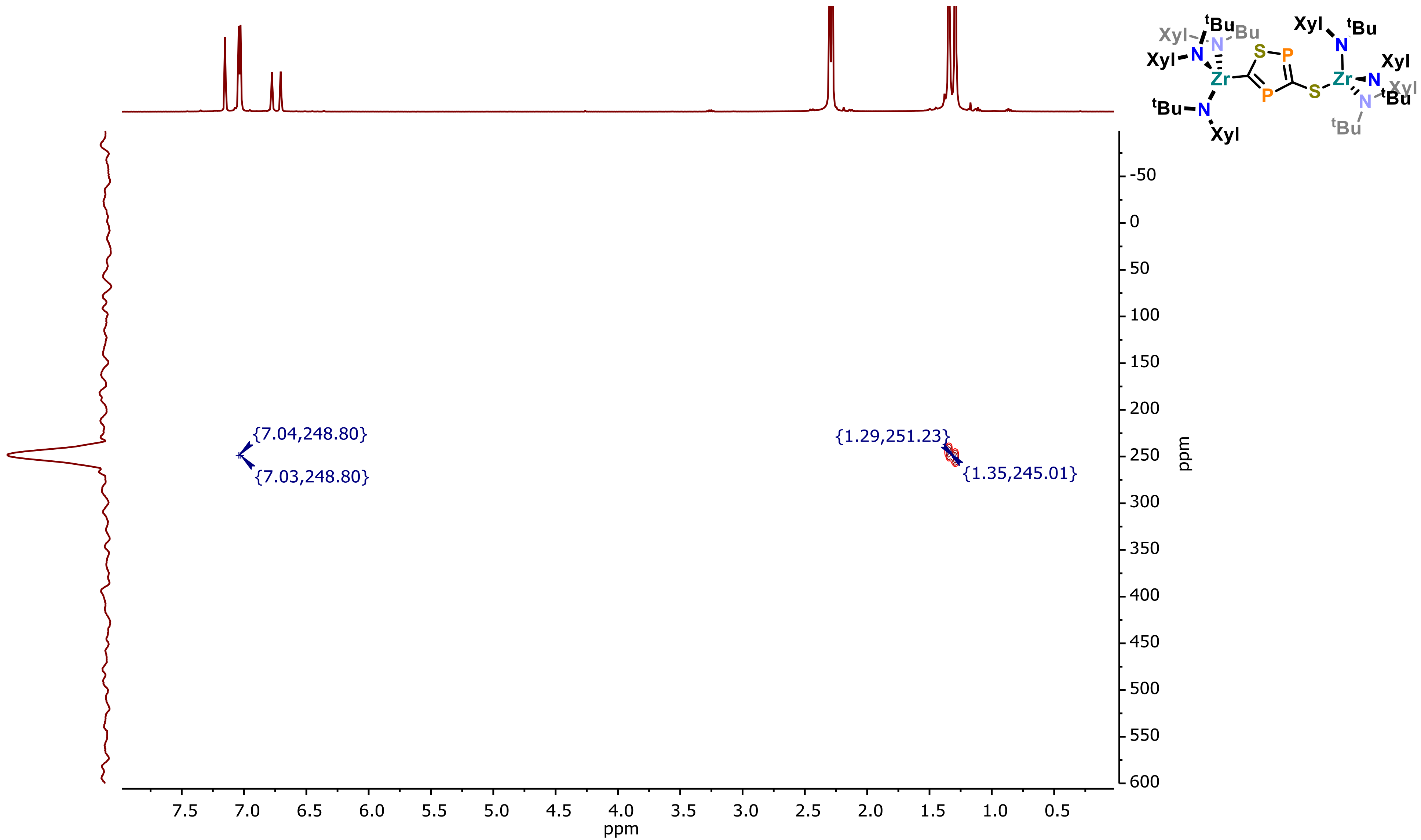

*Figure S 36: ^{1}H-^{15}N HMBC NMR spectrum of **2-SP** in C_6D_6 at 298 K.*

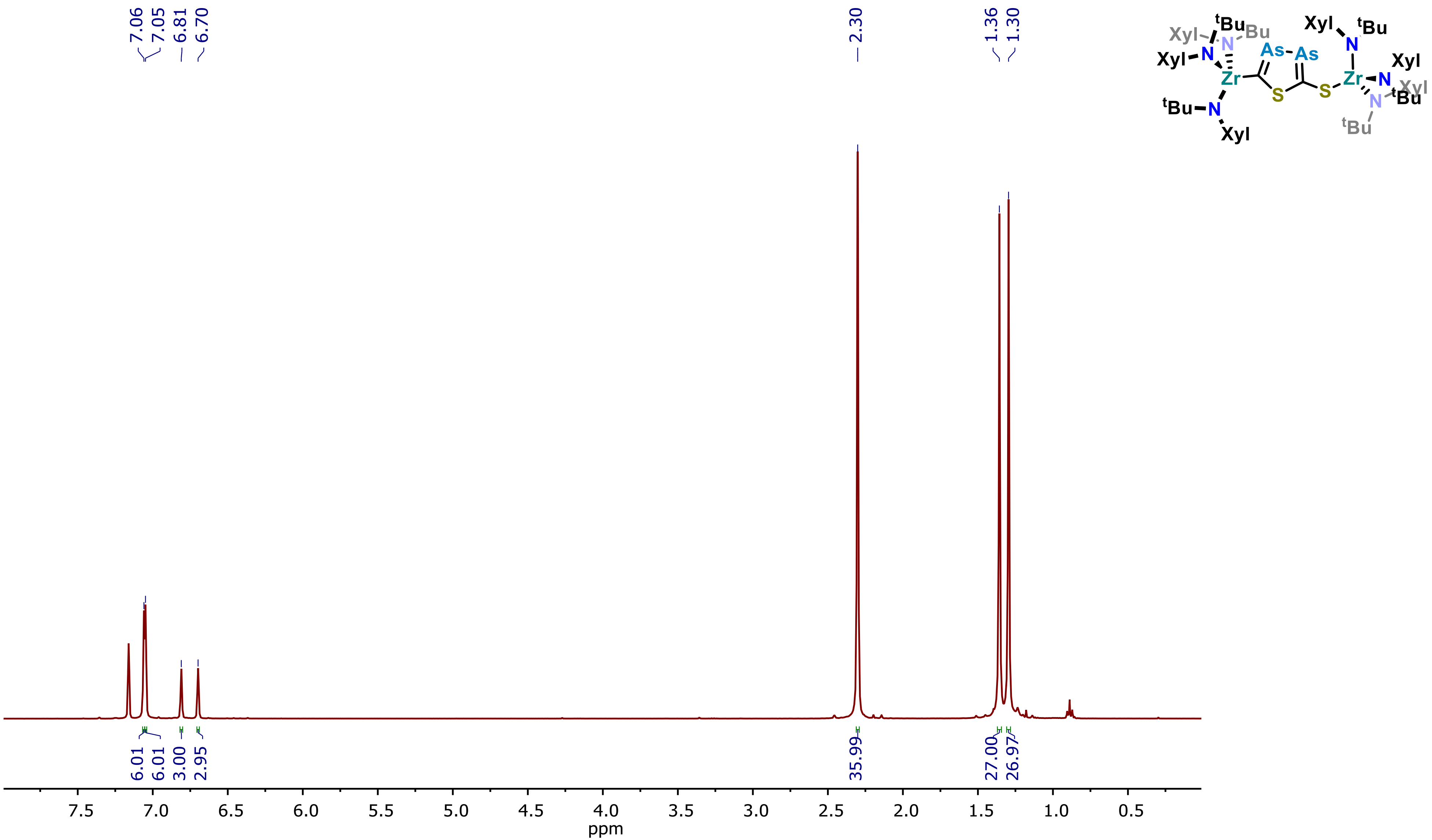

*Figure S 37: ^{1}H NMR spectrum of **3-AsAs** in C_6D_6 at 298 K.*

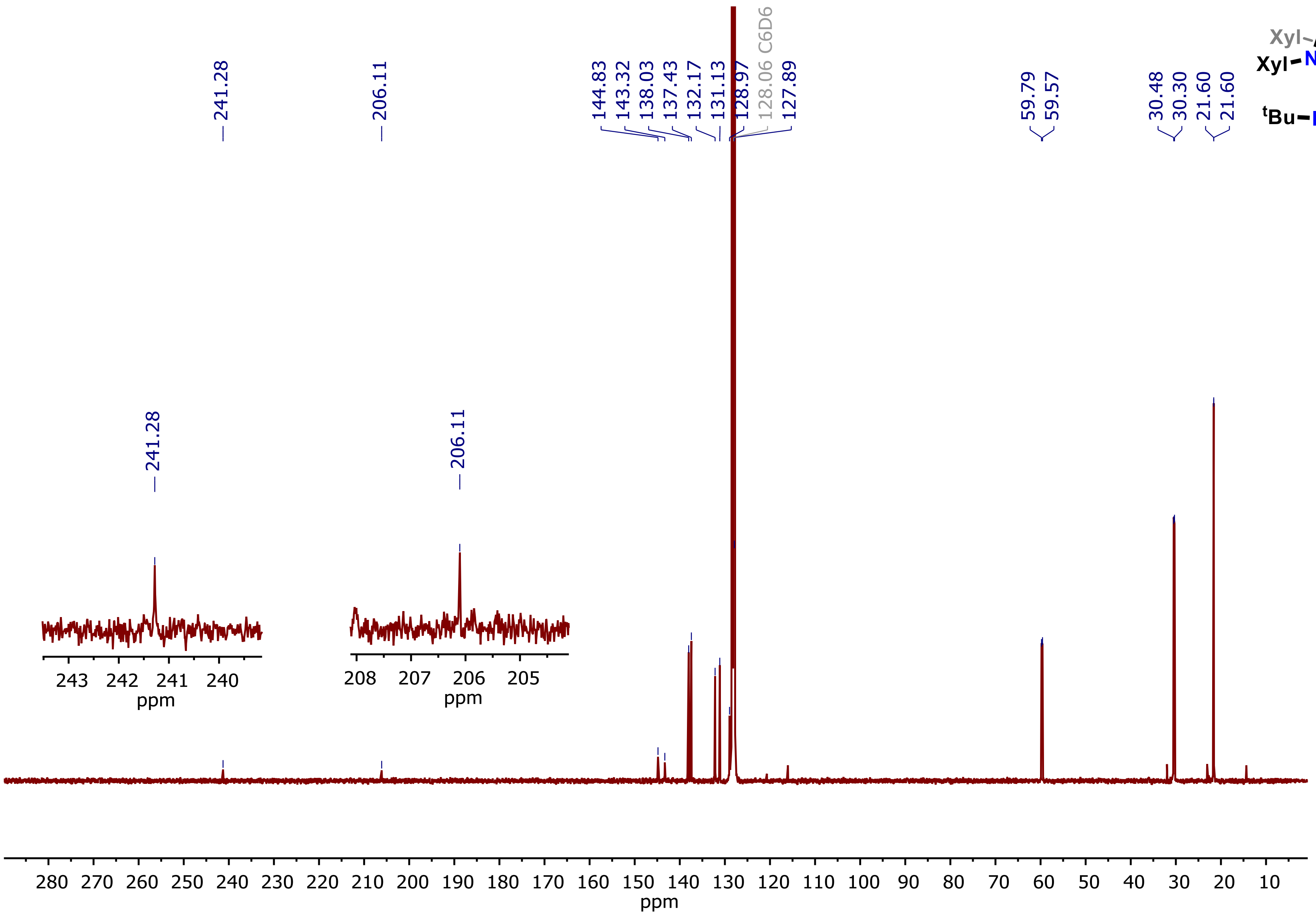

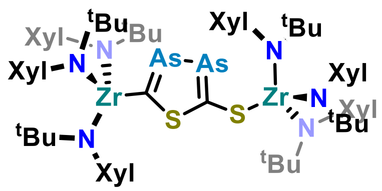

*Figure S 38: $^{13}C\{^1H\}$ NMR spectrum of **3-AsAs** in C_6D_6 at 298 K. The inlets show the enlarged sections 243.5 -239 and 208 - 204 ppm of the weak signals related to the heterocyclic carbons.*

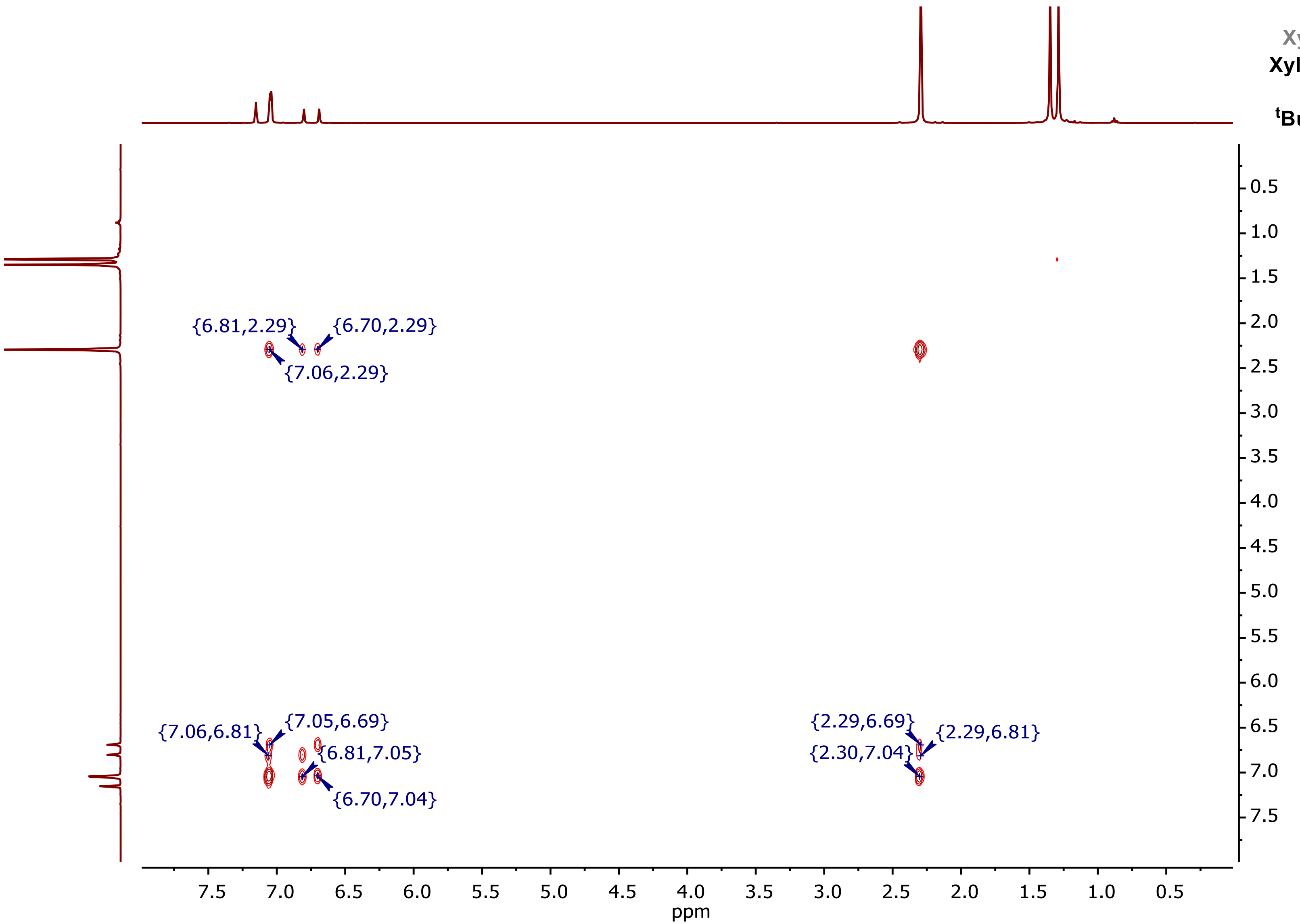

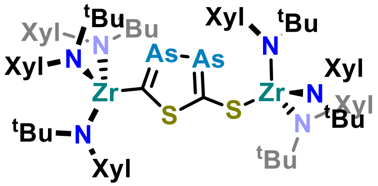

*Figure S 39: ¹H-¹H COSY NMR spectrum of **3-AsAs** in C_6D_6 at 298 K.*

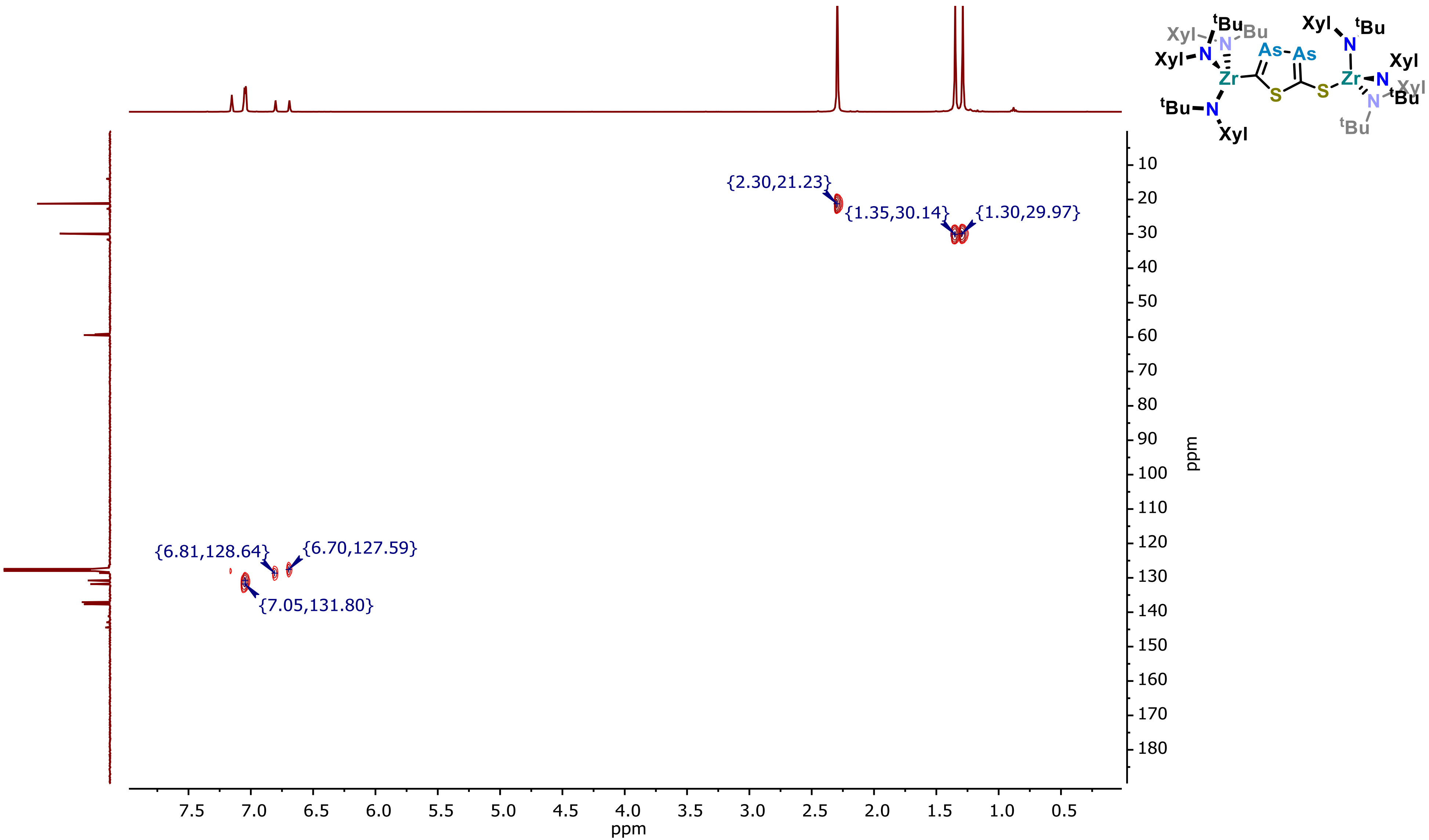

*Figure S 40: ^{1}H-^{13}C HSQC NMR spectrum of **3-AsAs** in C_6D_6 at 298 K.*

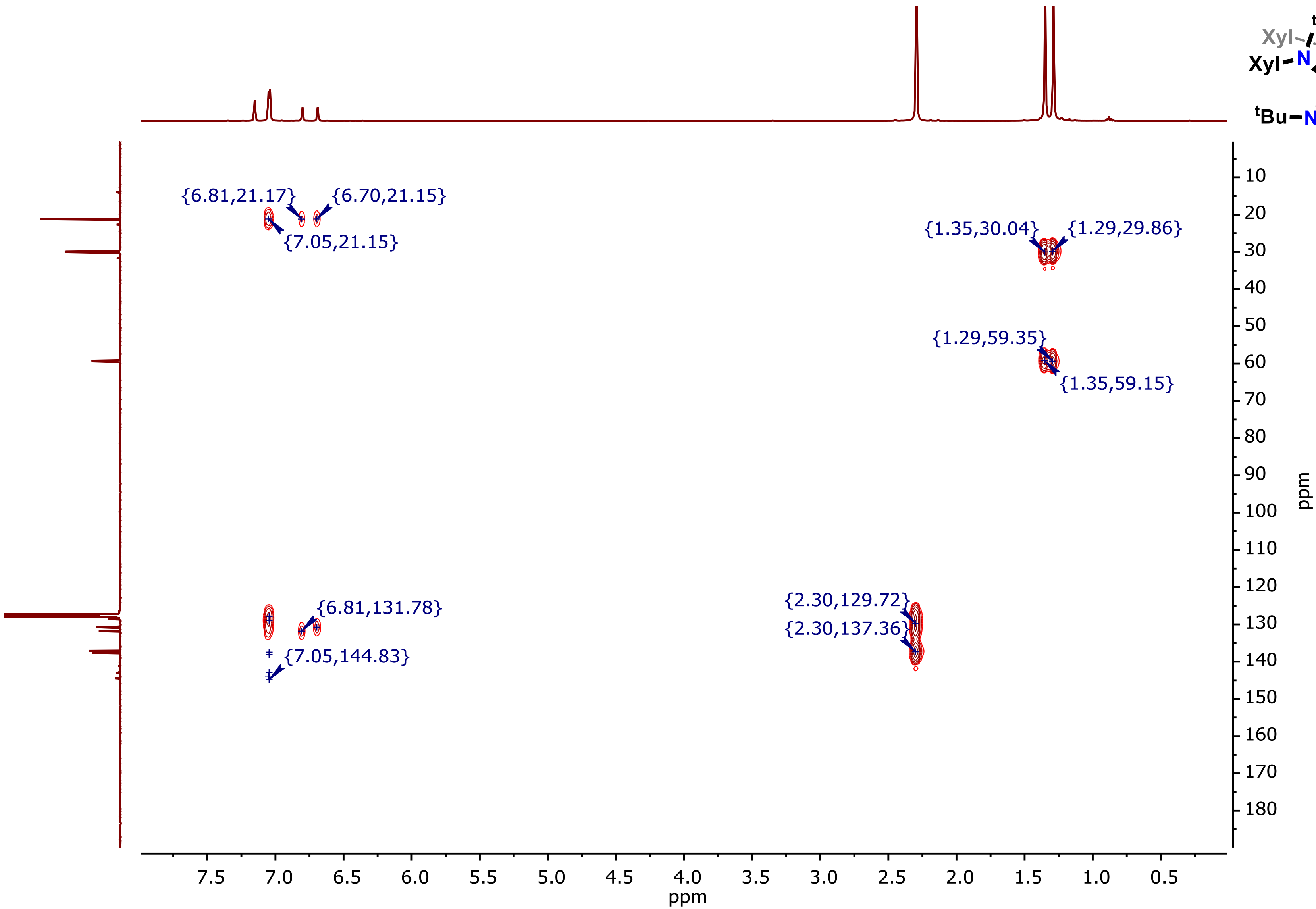

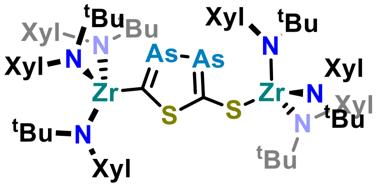

*Figure S 41: ^{1}H-^{13}C HMBC NMR spectrum of **3-AsAs** in C_6D_6 at 298 K.*

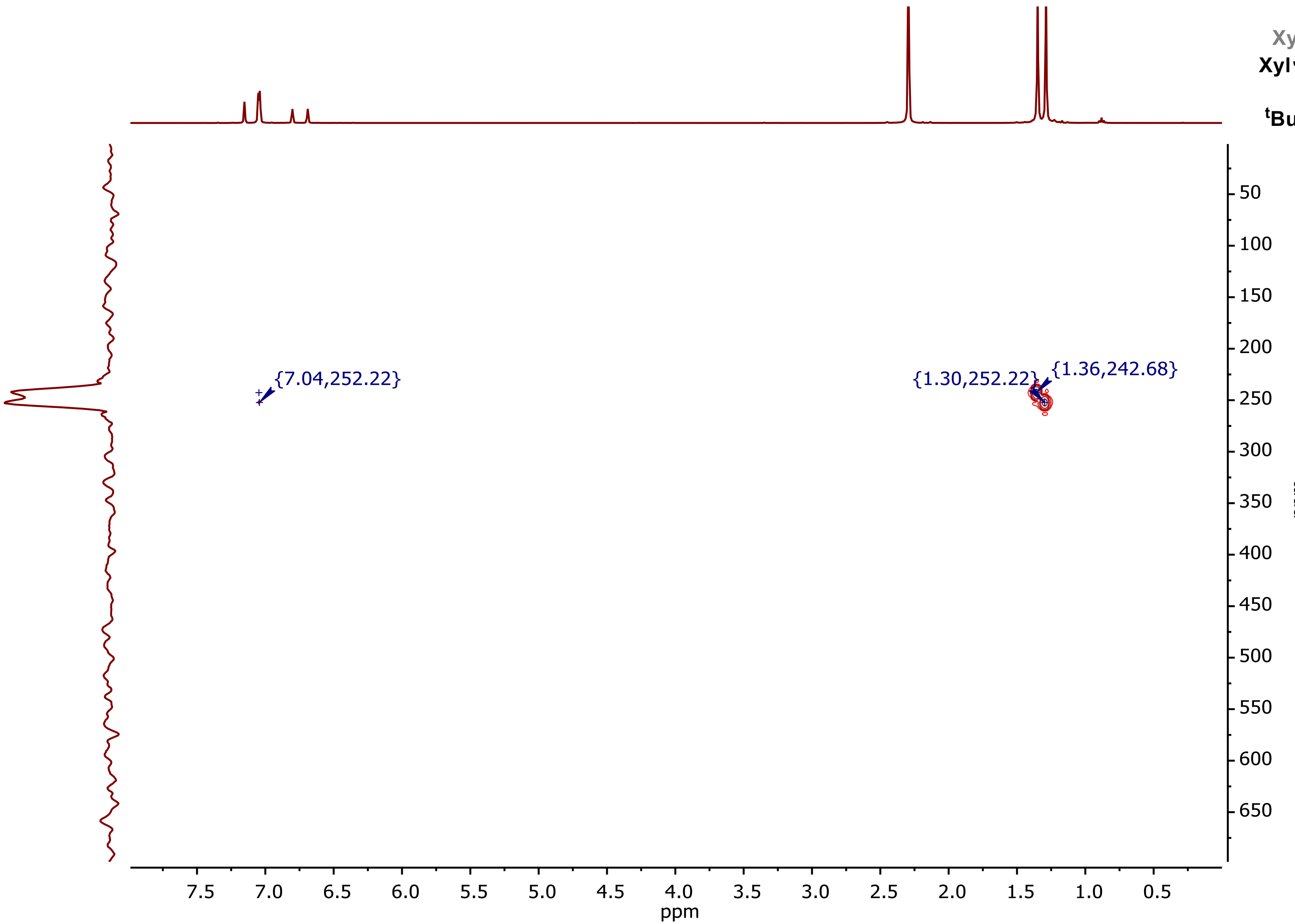

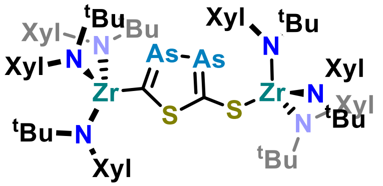

*Figure S 42: ^{1}H-^{15}N HMBC NMR spectrum of **3-AsAs** in C_6D_6 at 298K.*

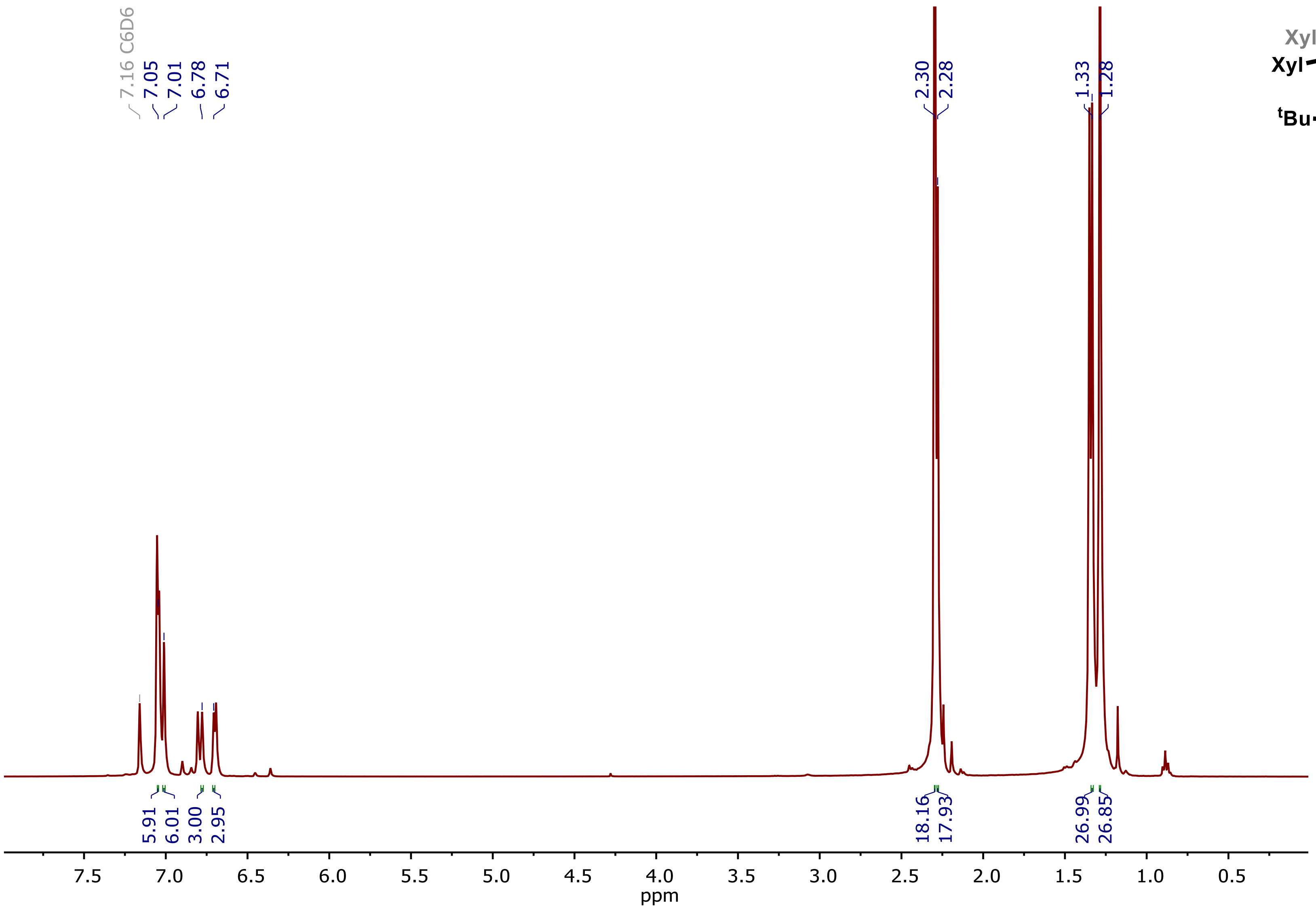

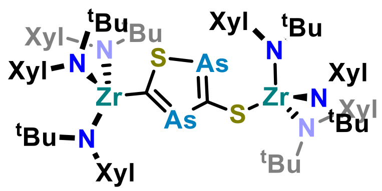

*Figure S 43: ^{1}H NMR spectrum of **3-SAs** (in a 51:49 mixture with **3-AsAs**) in C_6D_6 at 298 K. The selected peaks could be accounted for **4-SAs**.*

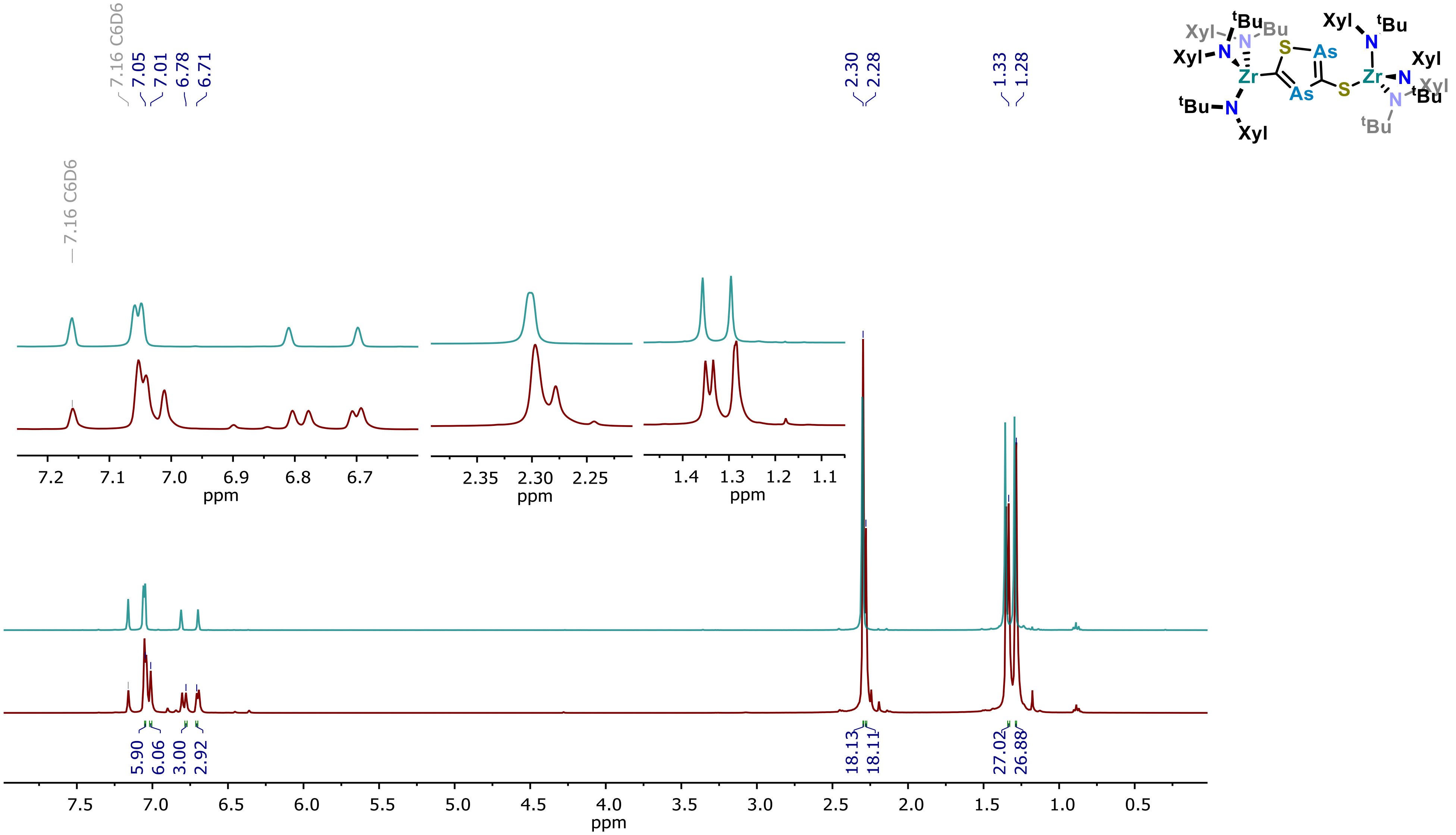

Figure S 44: Stack Plot of ^{1}H NMR spectra of isolated ***3-AsAs*** *(top, blue) and the mixture obtained on the synthesis of* ***3-SAs/3-AsAs*** *(bottom, red) to further highlight the differences between the two species. The inlets show the enlarged of areas 7.25 – 6.60, 2.40 – 2.20 and 1.50 – 1.15 ppm to further enhance clarity of the different signals.*

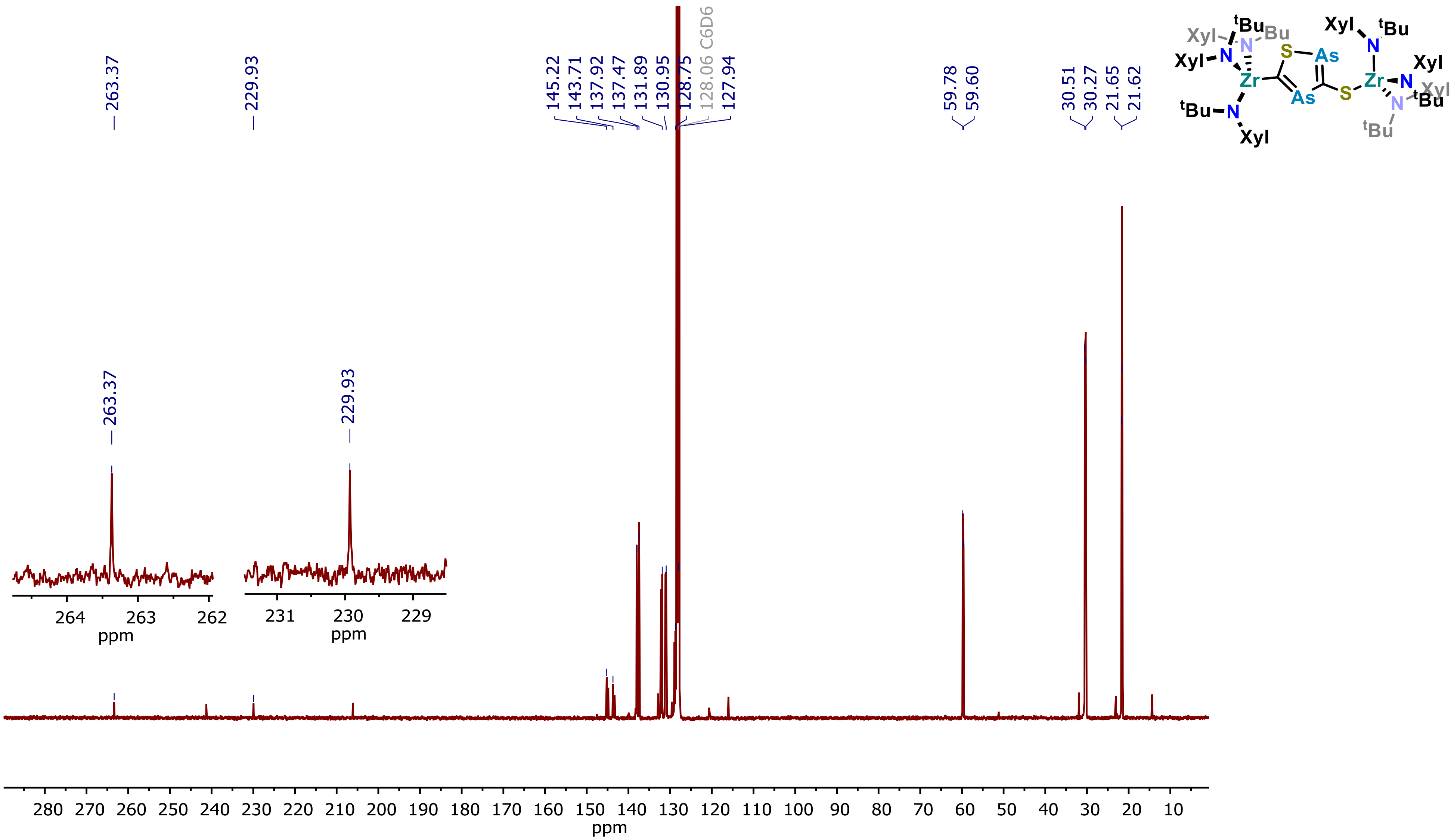

*Figure S 45: $^{13}C\{^{1}H\}$ NMR spectrum of **3-SAs** (in a 51:49 mixture with **3-AsAs**) in C_6D_6 at 298 K. The selected peaks could be accounted for **3-SAs**. The inlets show the enlarged sections 264.8 -262 and– 228.5 - 231.5 ppm for better visibility of both signals related to the heterocyclic carbon atoms.*

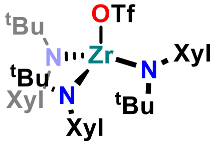

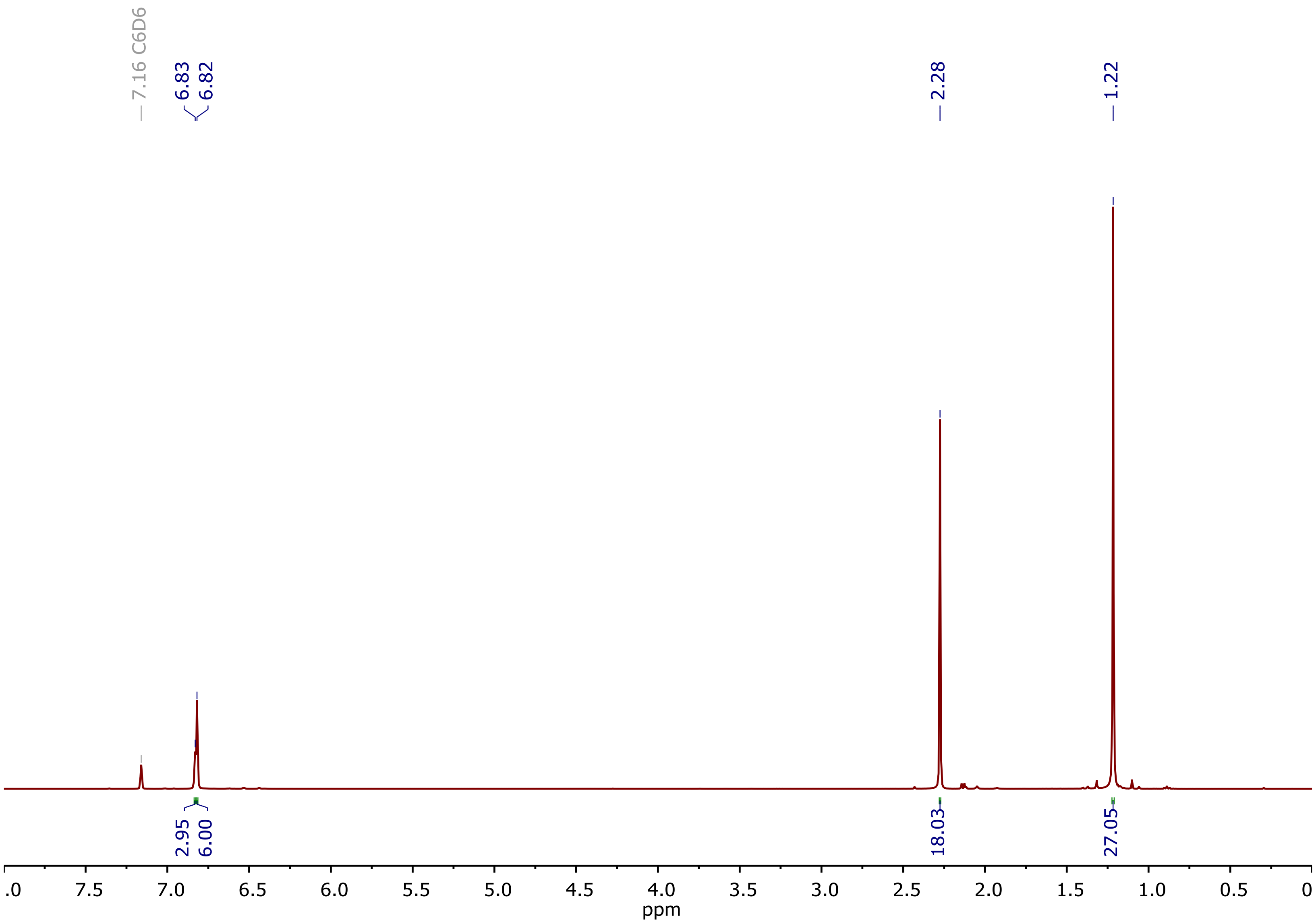

*Figure S 46: ^{1}H NMR spectrum of **1-OTf** in C_6D_6 at 298 K.*

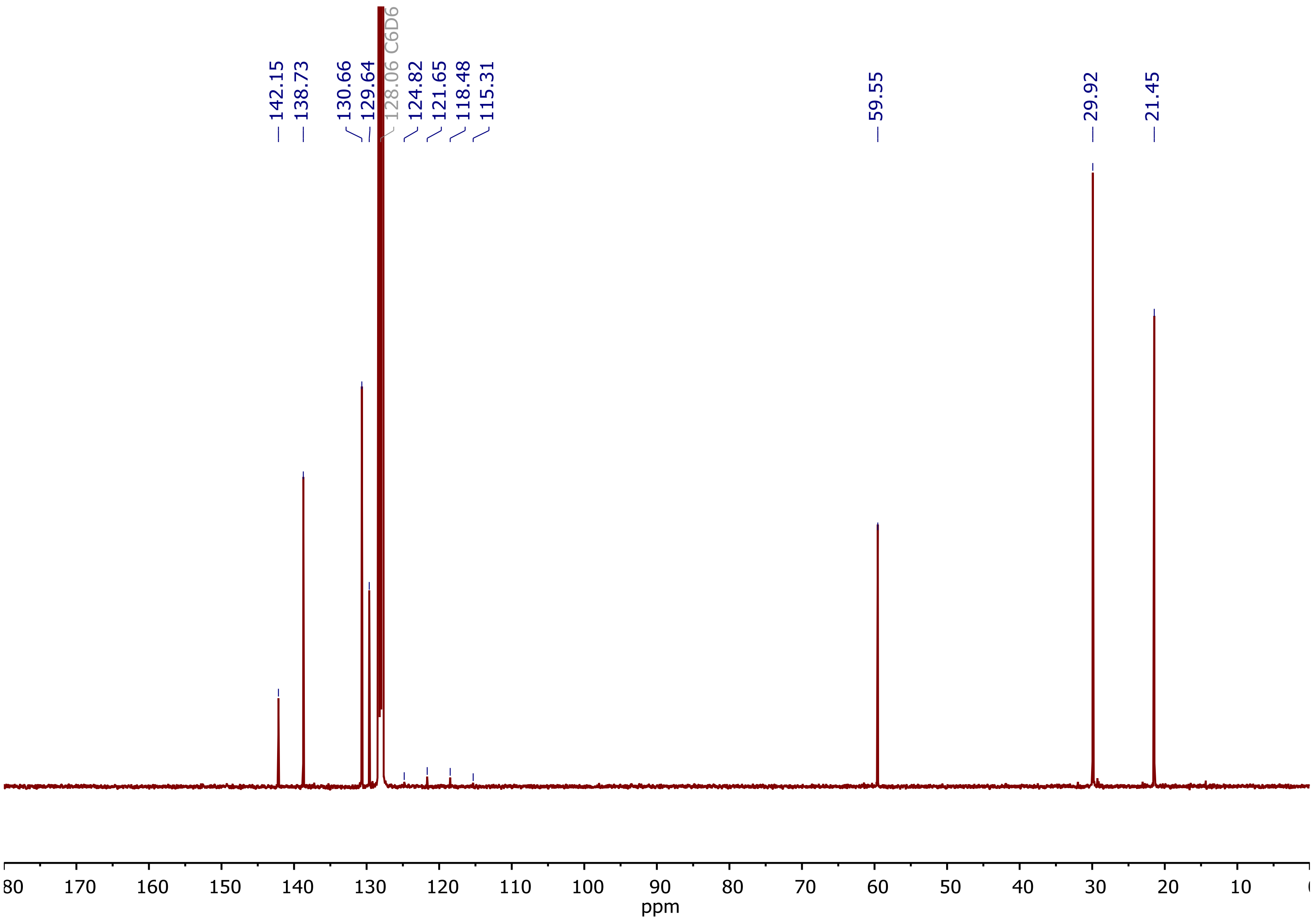

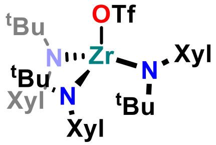

*Figure S 47: ^{13}C NMR spectrum of **1-OTf** in C_6D_6 at 298 K.*

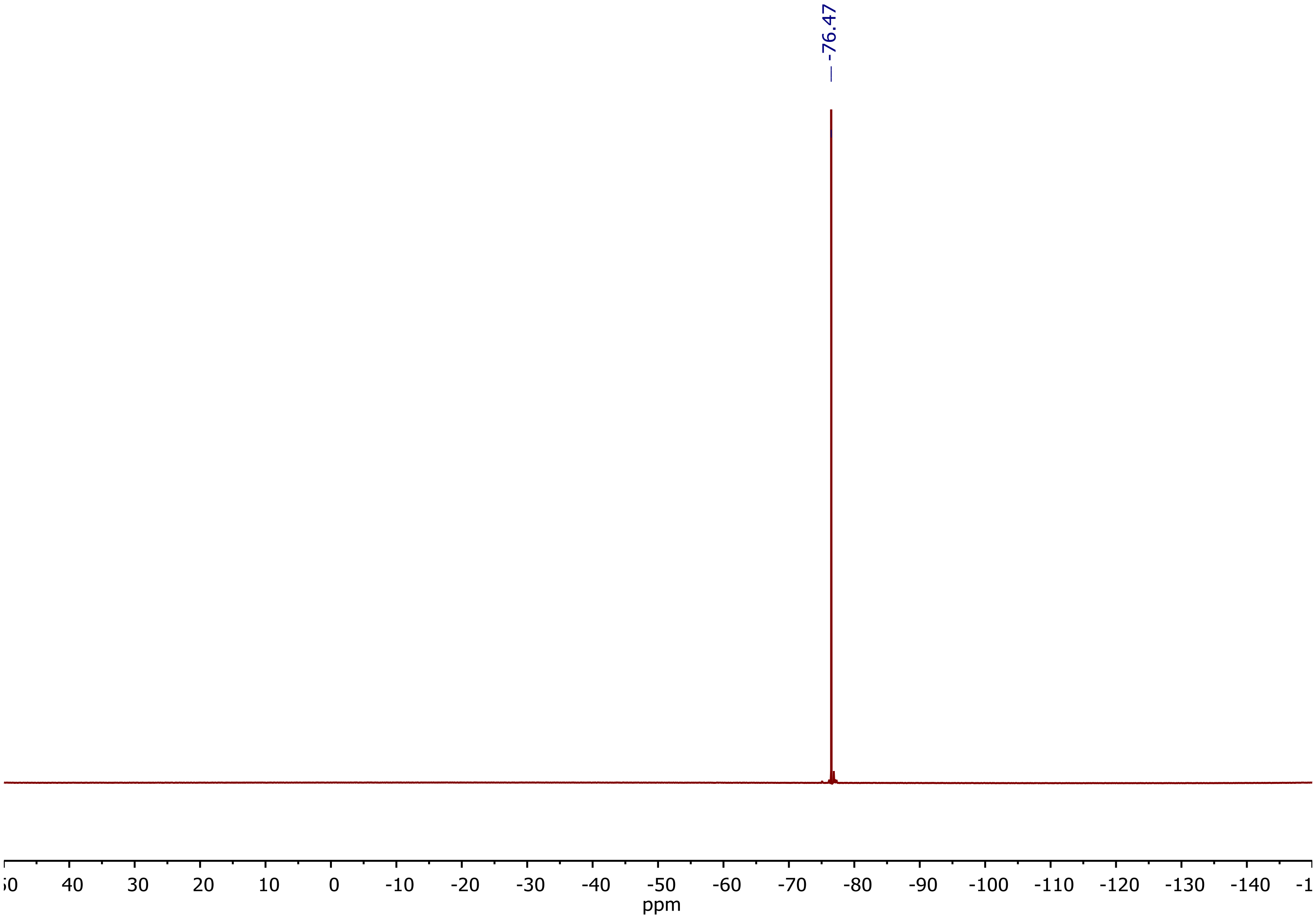

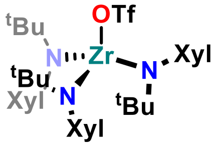

*Figure S 48: ^{19}F NMR spectrum of **1-OTf** in C_6D_6 at 298 K.*

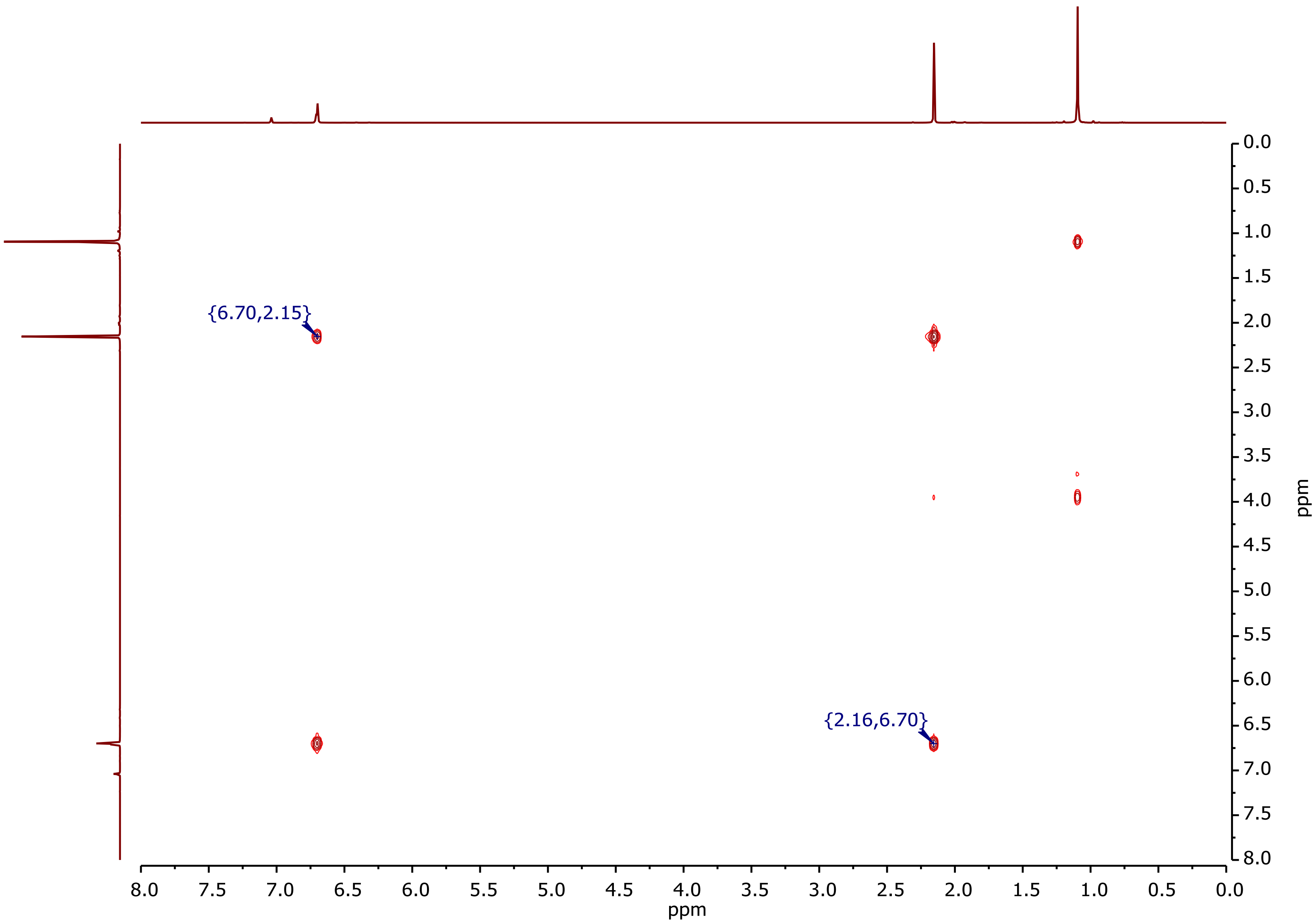

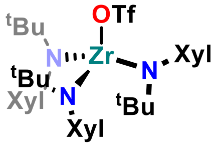

*Figure S 49: ${}^{1}H$-${}^{1}H$ COSY NMR spectrum of **1-OTf** in C_6D_6 at 298 K.*

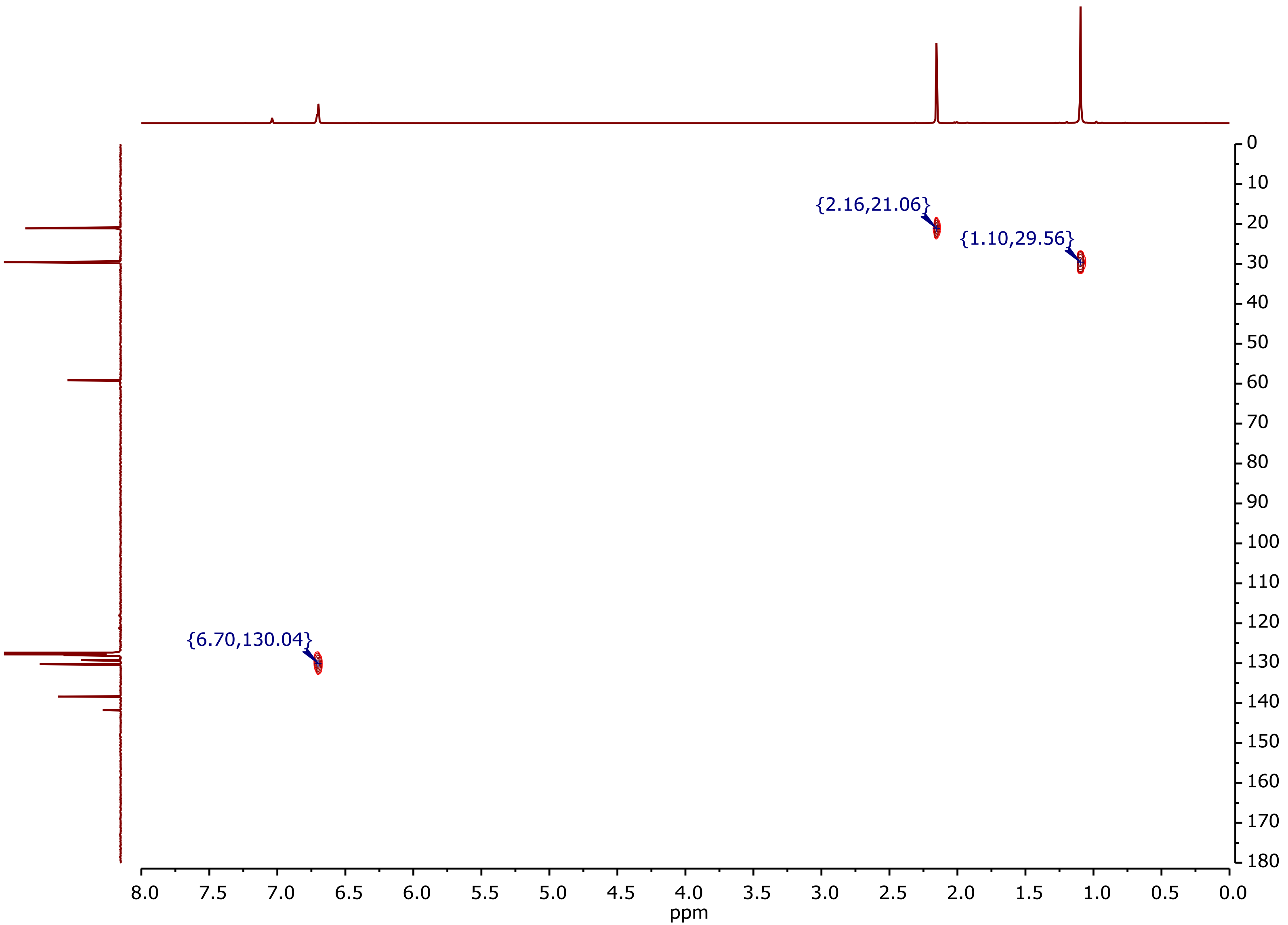

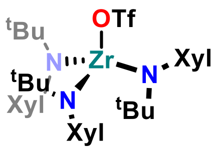

*Figure S 50: ^{1}H-^{13}C HSQC NMR spectrum of **1-OTf** in C_6D_6 at 298 K.*

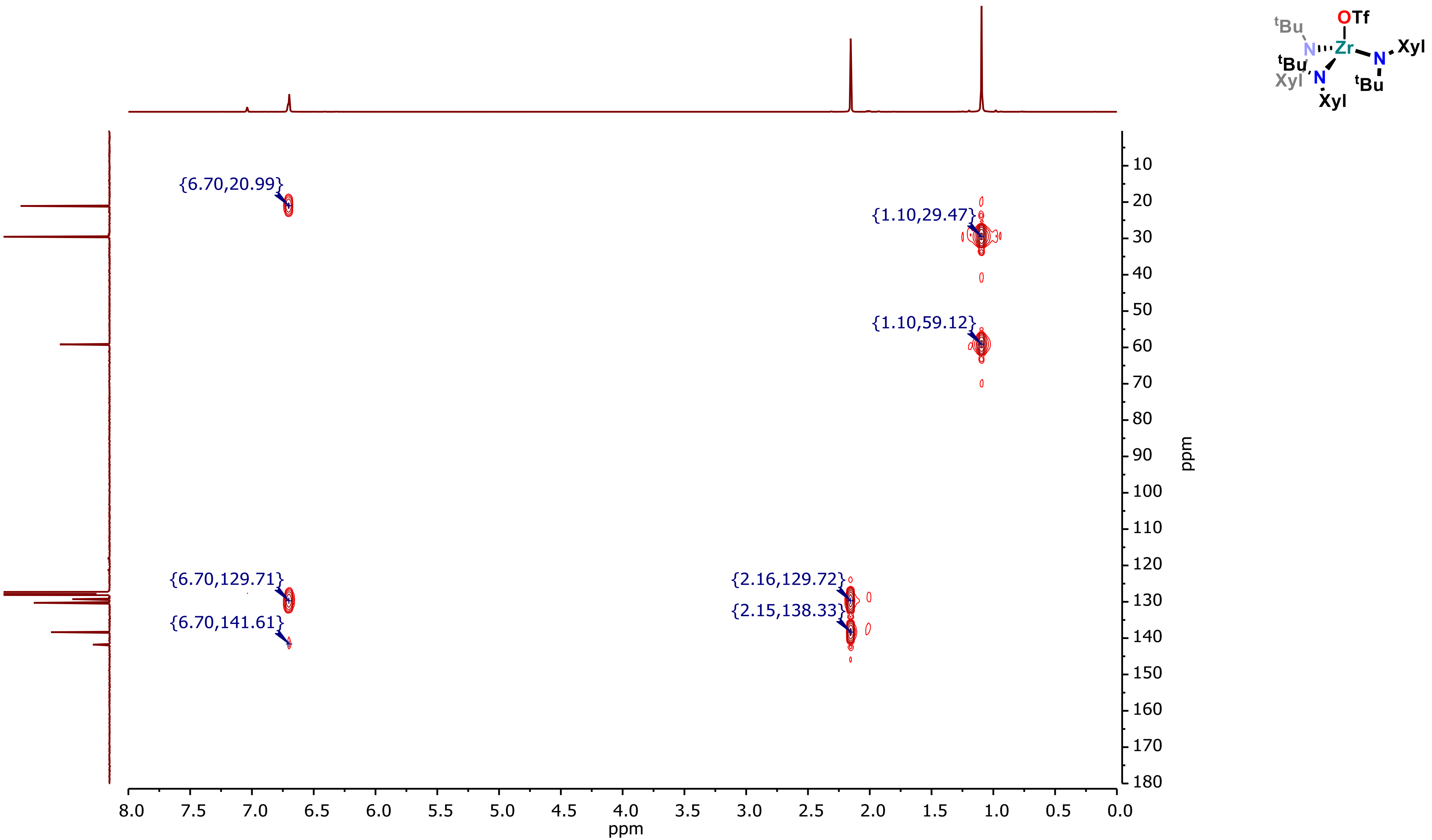

*Figure S 51: ^1H-^{13}C HMBC NMR spectrum of **1-OTf** in C_6D_6 at 298 K.*

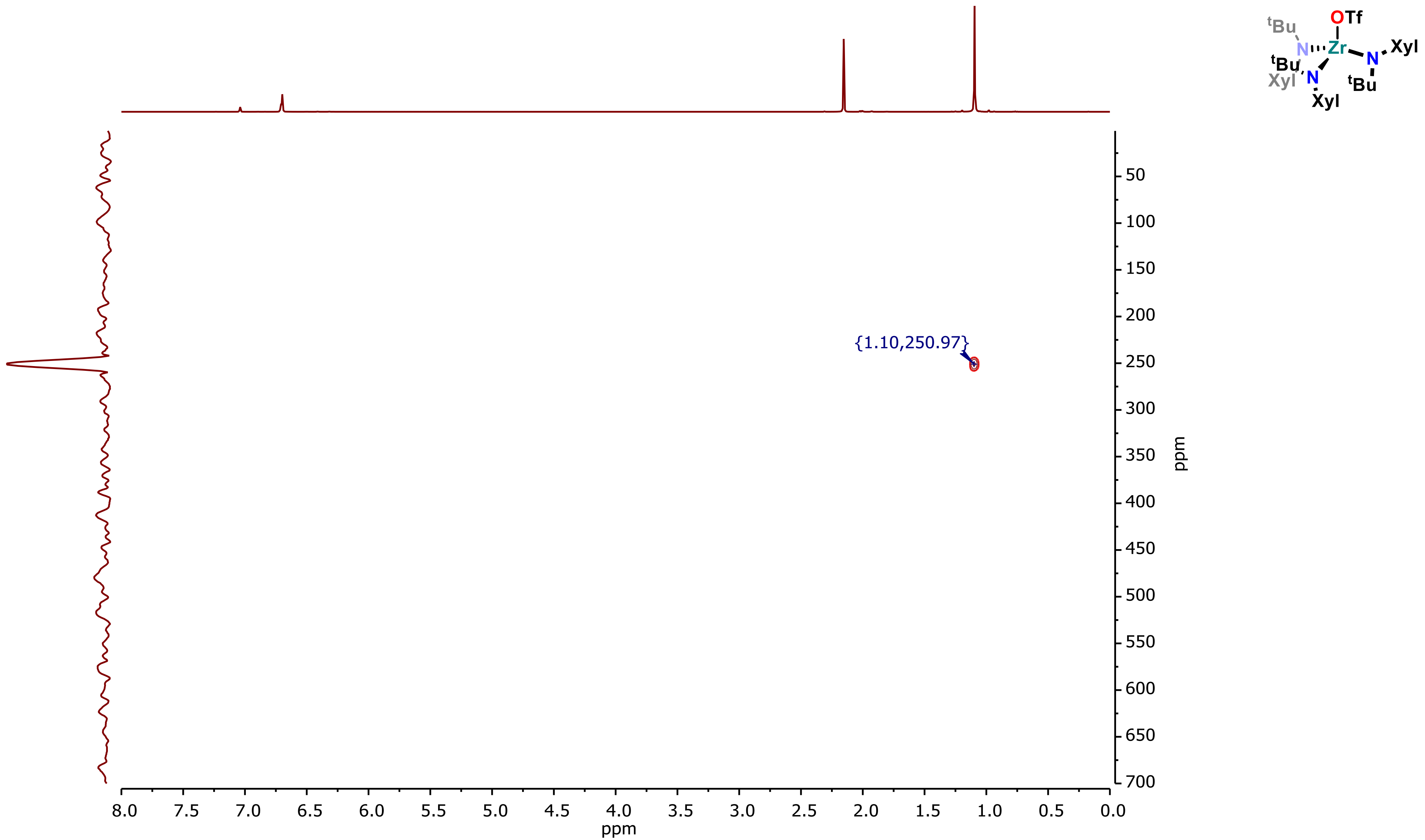

*Figure S 52: ^{1}H-^{15}N HMBC NMR spectrum of **1-OTf** in C_6D_6 at 298 K.*

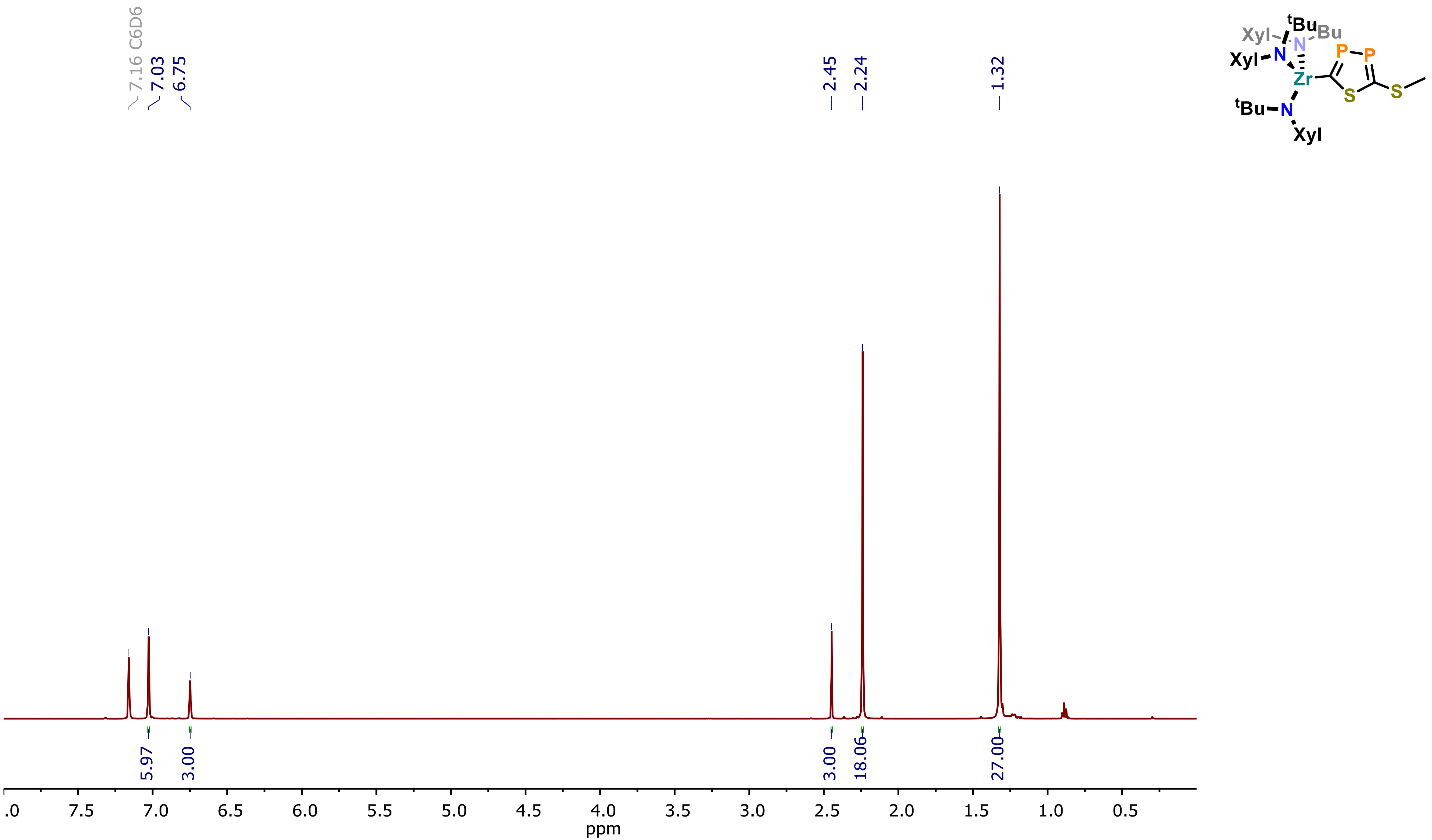

*Figure S 53: ^{1}H NMR spectrum of **4-PP** in C_6D_6 at 298 K.*

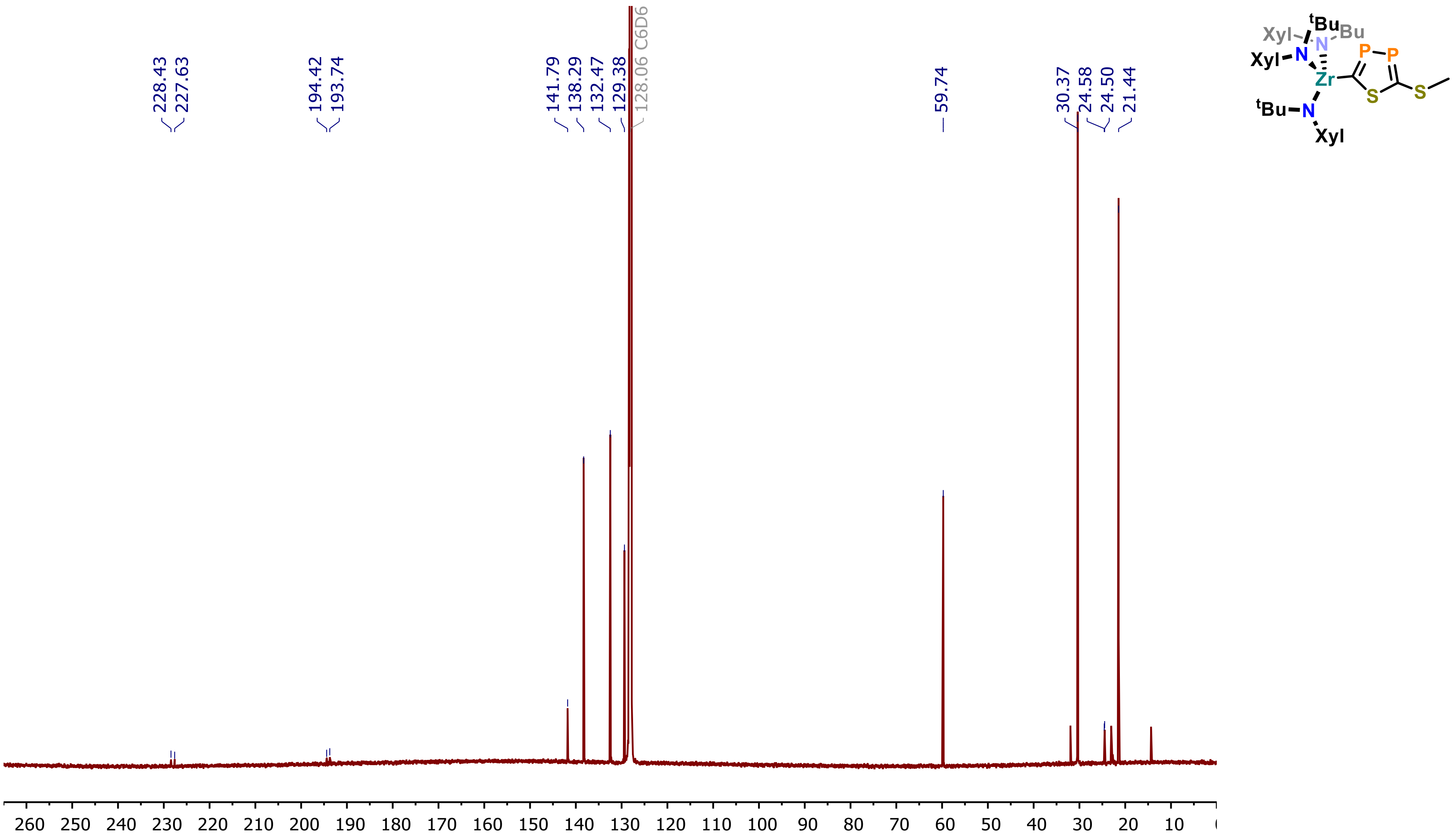

*Figure S 54: $^{13}C\{^{1}H\}$ NMR spectrum of **4-PP** in C_6D_6 at 298 K. Three unchecked signals in the aliphatic region can be assigned to small impurities of* n*-hexane.*

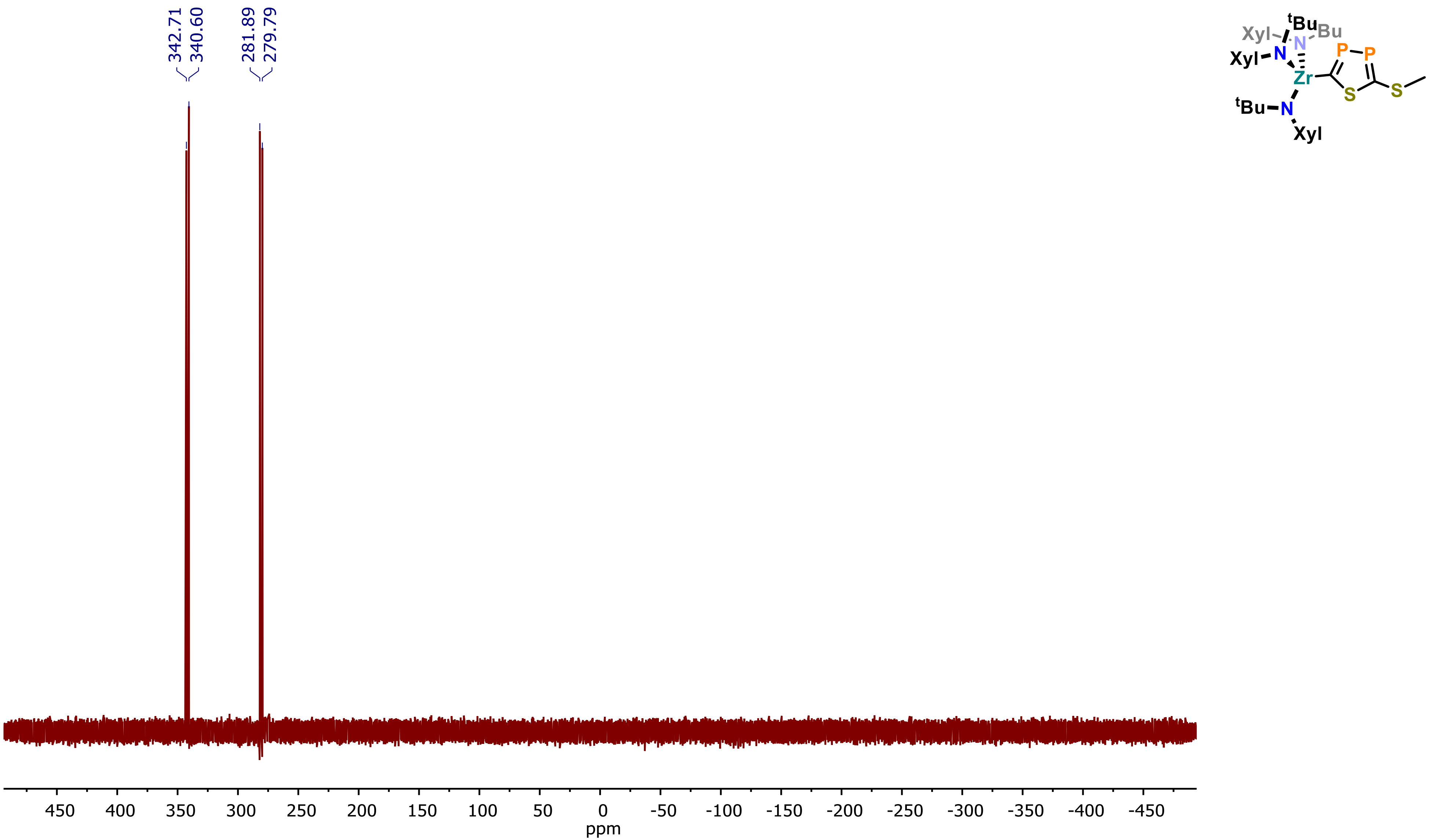

*Figure S 55: $^{31}P\{^{1}H\}$ NMR spectrum of **4-PP** in C_6D_6 at 298 K.*

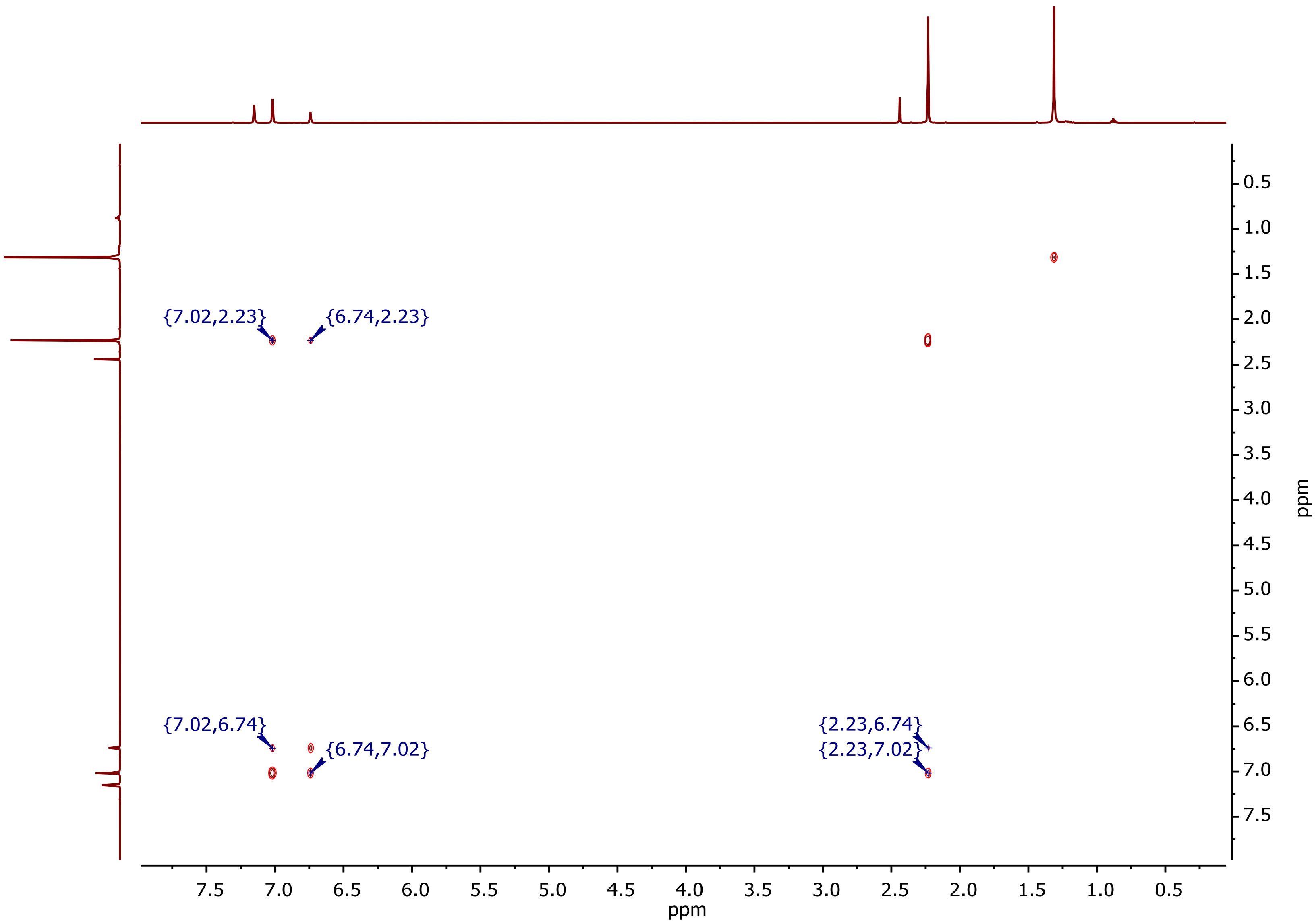

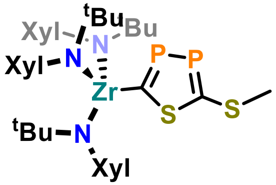

*Figure S 56: ^{1}H-^{15}N COSY NMR spectrum of **4-PP** in C_6D_6 at 298 K.*

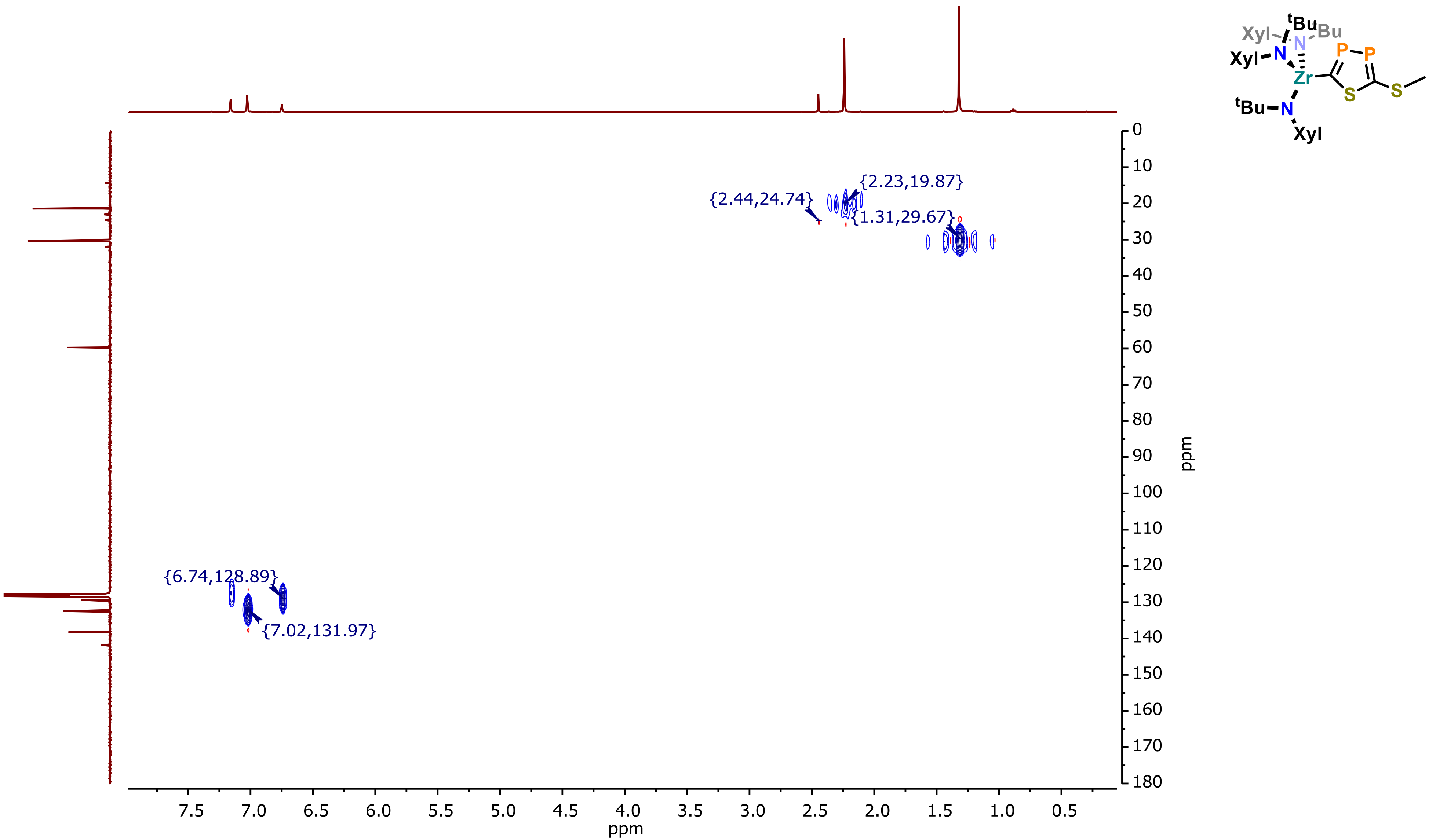

*Figure S 57: ^{1}H-^{13}C HSQC NMR spectrum of 4-**PP** in C_6D_6 at 298 K.*

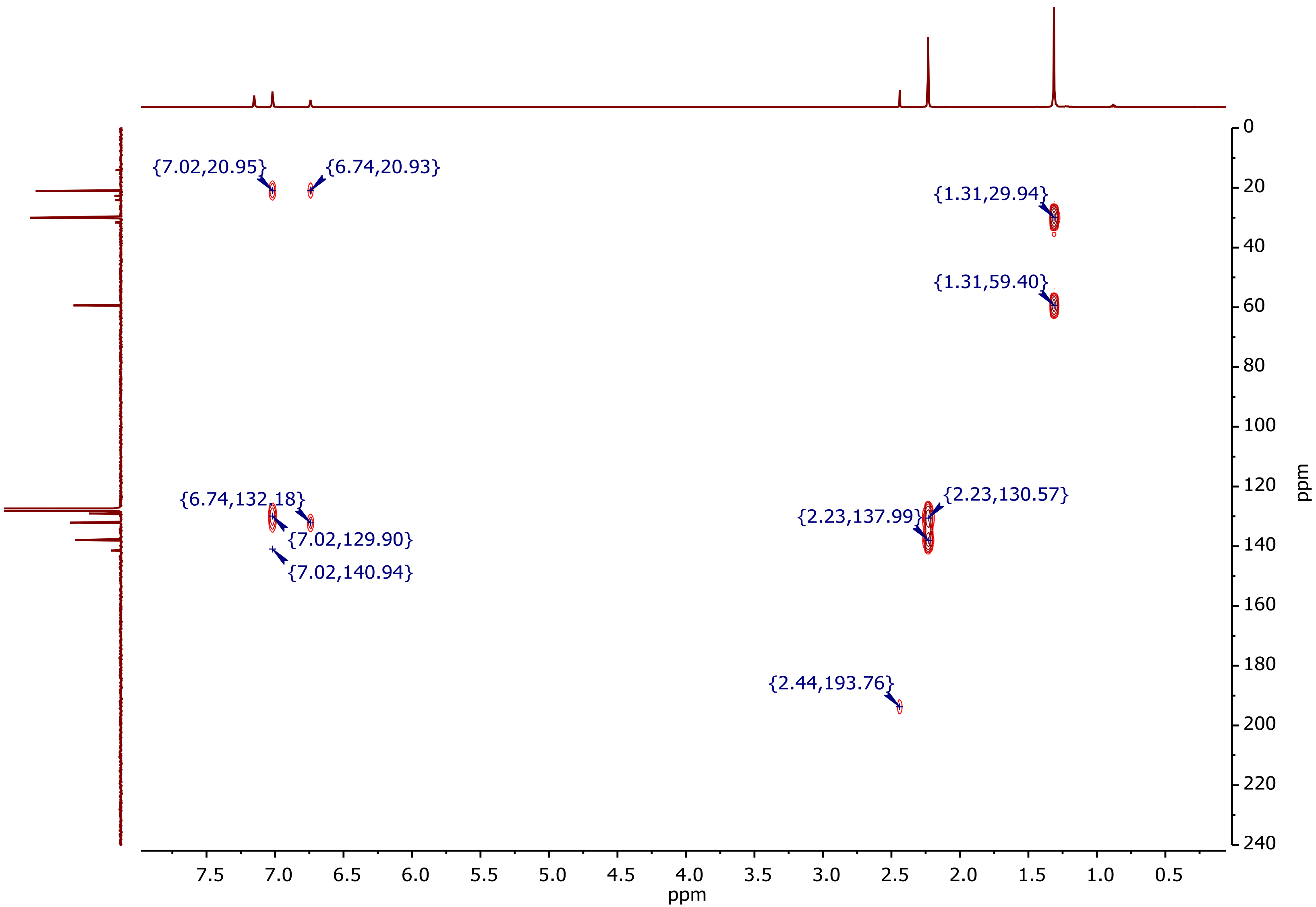

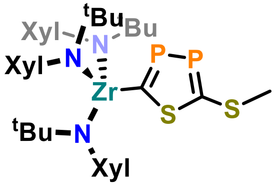

*Figure S 58: ^{1}H-^{13}C HMBC NMR spectrum of **4-PP** in C_6D_6 at 298 K.*

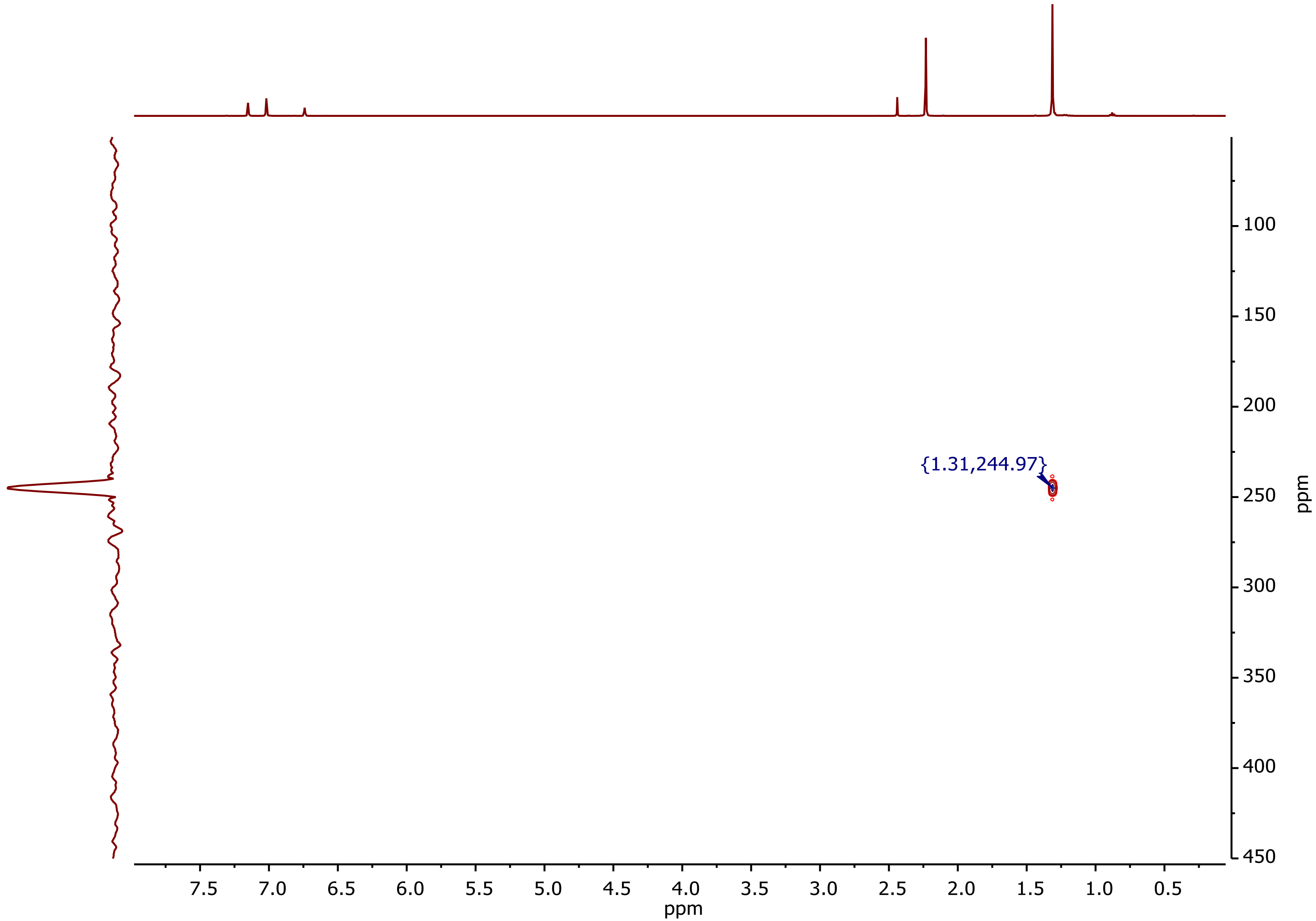

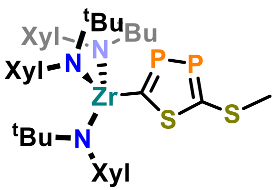

*Figure S 59: ^{1}H-^{15}N HMBC NMR spectrum of **4-PP** in C_6D_6 at 298 K.*

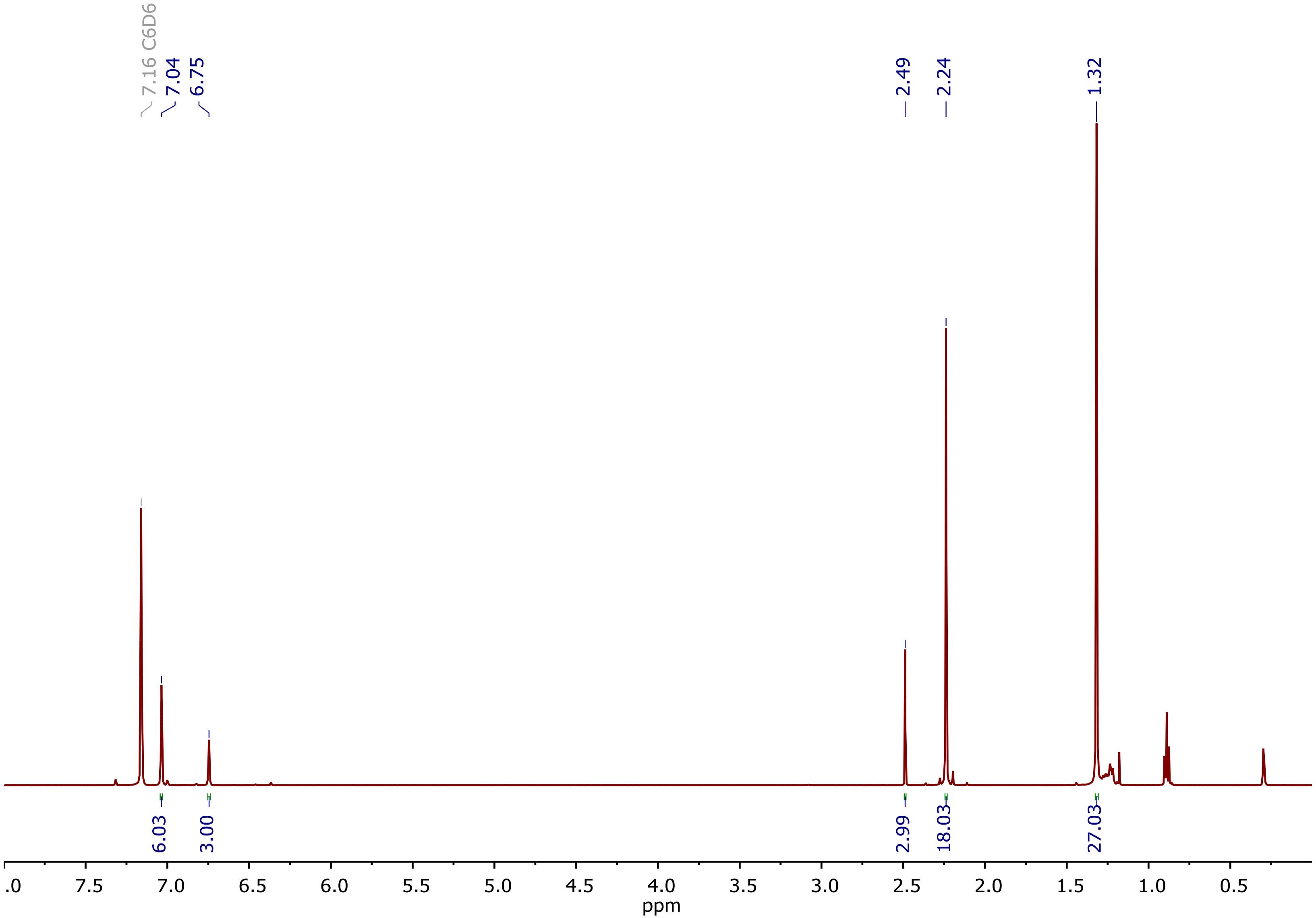

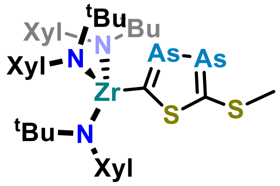

*Figure S 60: ^{1}H NMR spectrum of **5-AsAs** in C_6D_6 at 298 K.*

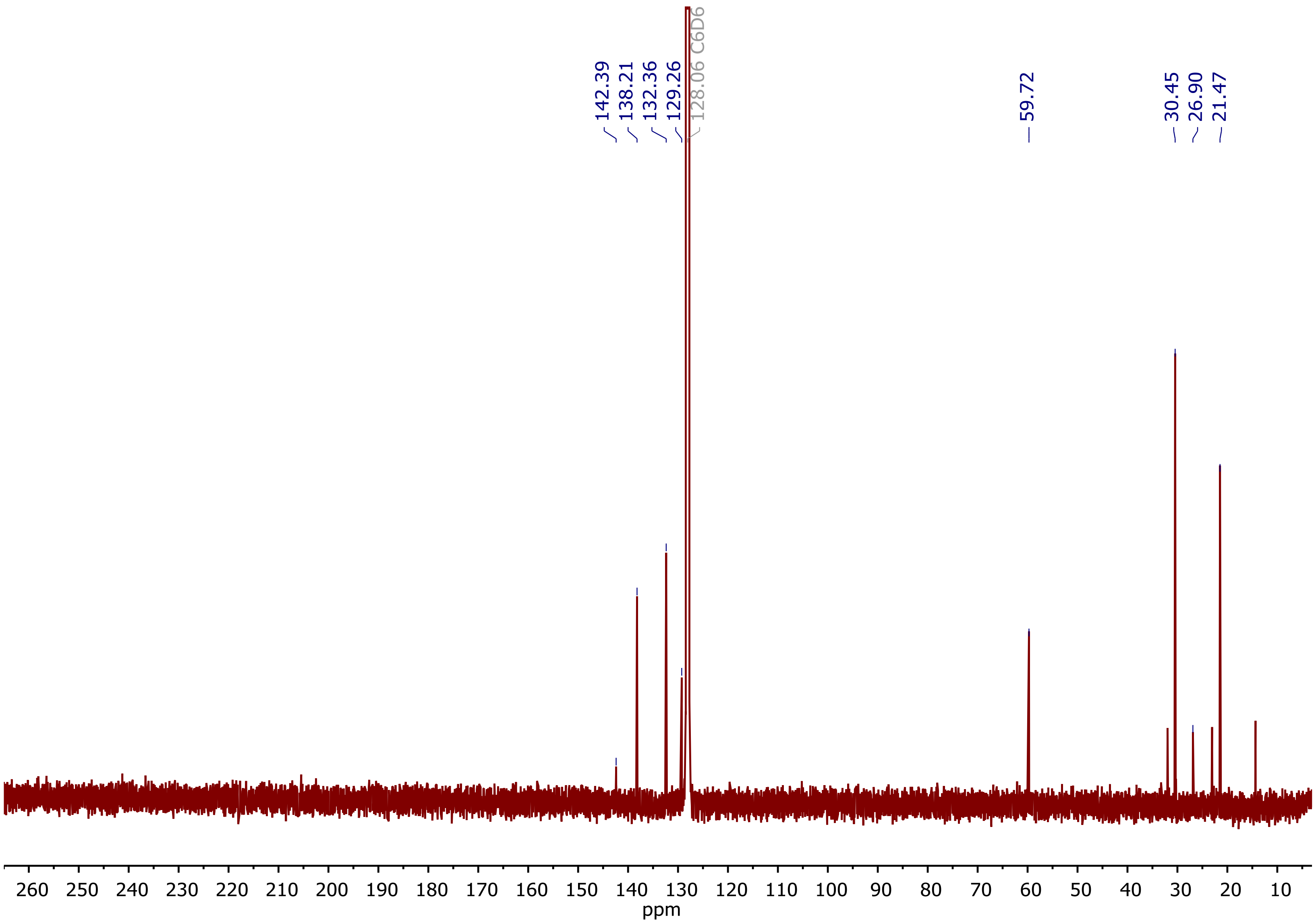

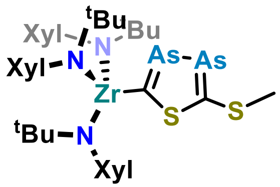

*Figure S 61: $^{13}C\{^1H\}$ NMR spectrum of **5-AsAs** in C_6D_6 at 298 K. Unmarked signals in the aliphatic region can be assigned to residual* n*-hexane from the crystallization.*

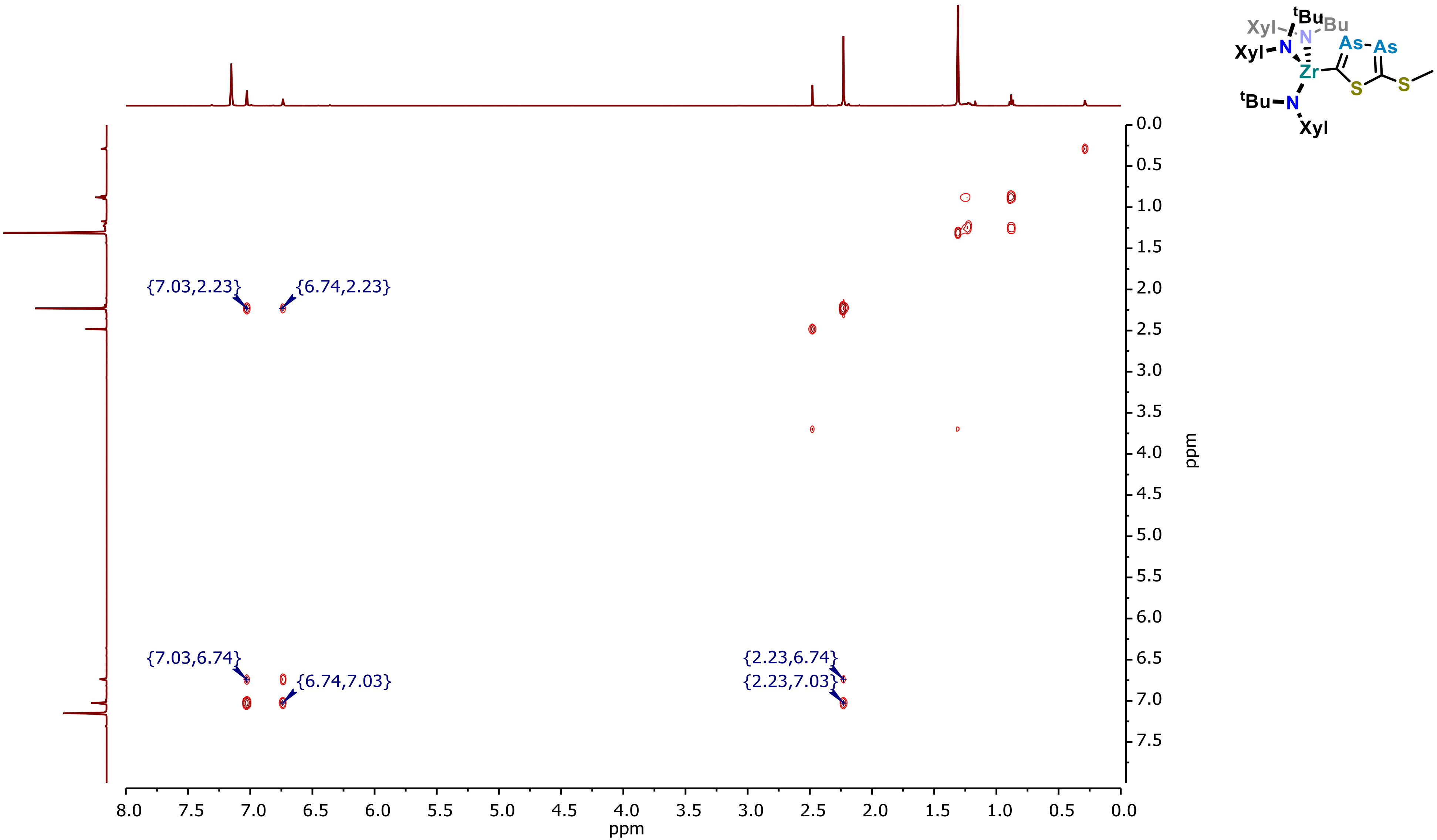

*Figure S 62: ^{1}H-^{1}H COSY NMR spectrum of **5-AsAs** in C_6D_6 at 298 K.*

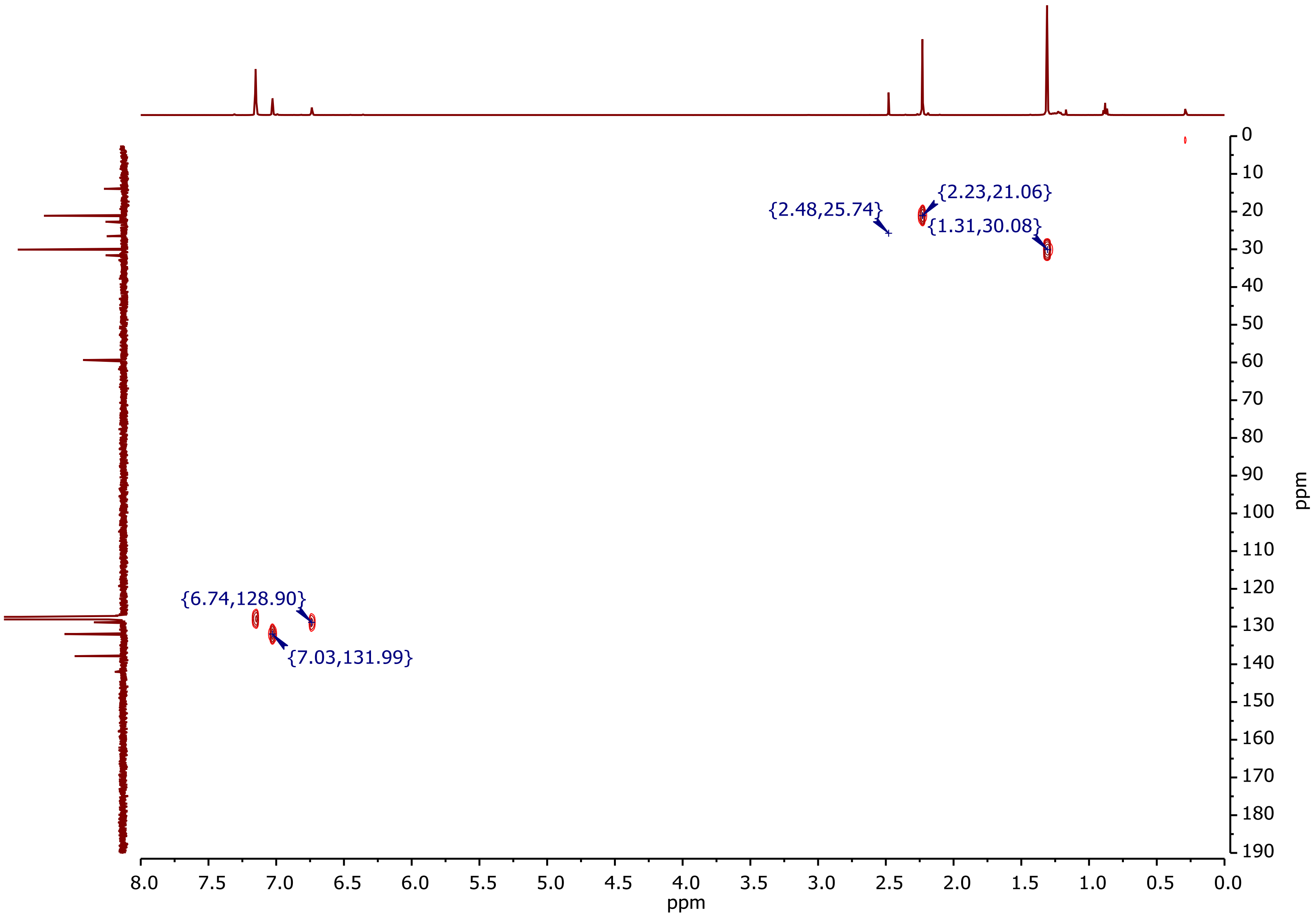

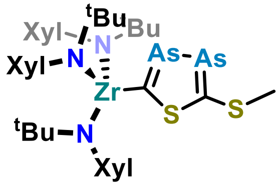

*Figure S 63: ^{1}H-^{13}C HSQC NMR spectrum of **5-AsAs** in C_6D_6 at 298 K.*

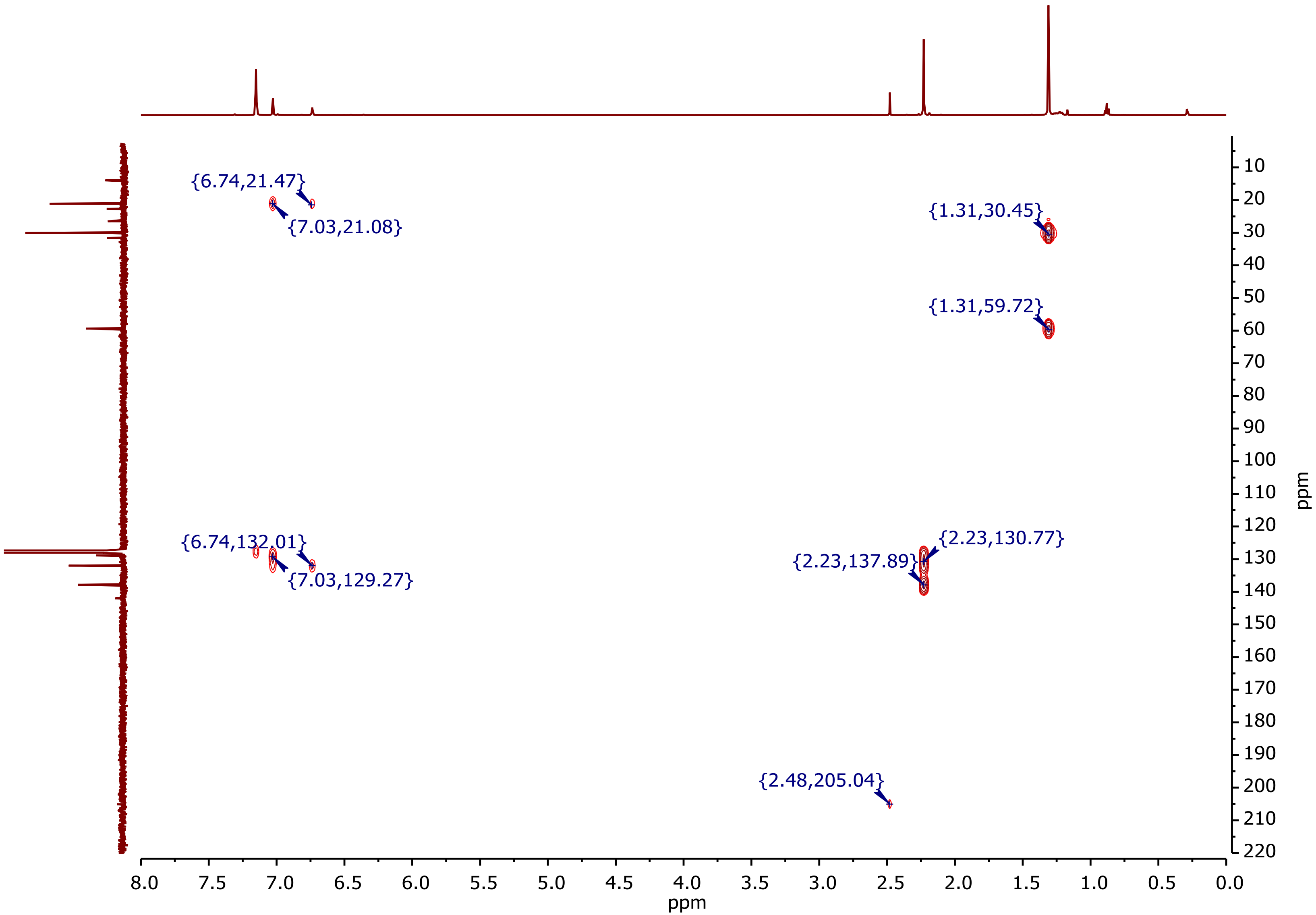

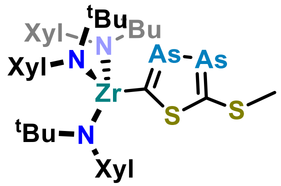

*Figure S 64:^{1}H-^{13}C HMBC NMR spectrum of **5-AsAs** in C_6D_6 at 298 K. On the vertical scale, the signal at 205 ppm indicates one of the two heteroaromatic carbon atoms which cannot be seen in the 1D ^{13}C NMR spectrum (compare S61X).*

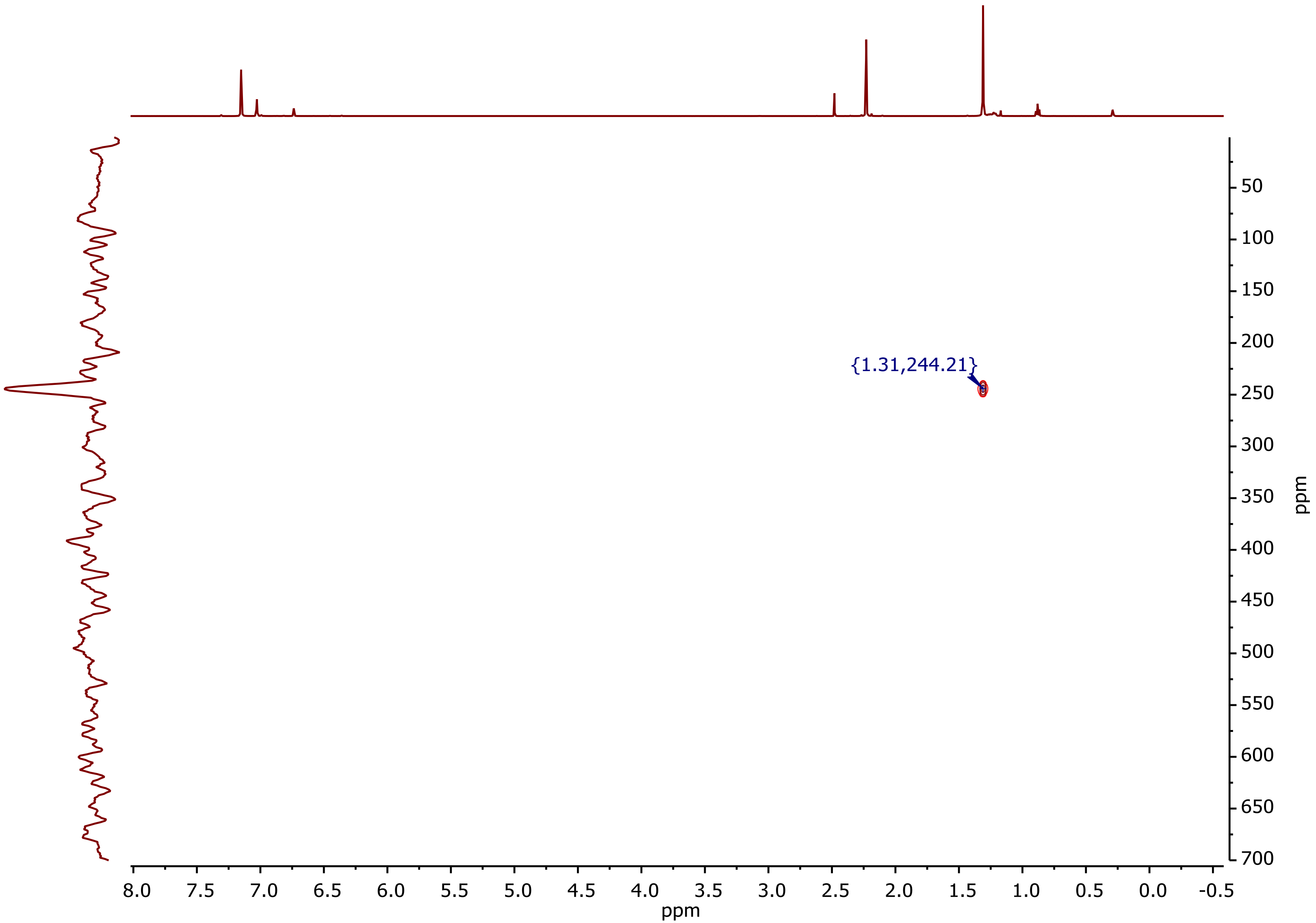

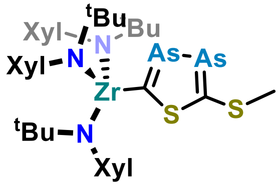

*Figure S 65: ^{1}H-^{15}N HMBC NMR spectrum of **5-AsAs** in C_6D_6 at 298 K.*

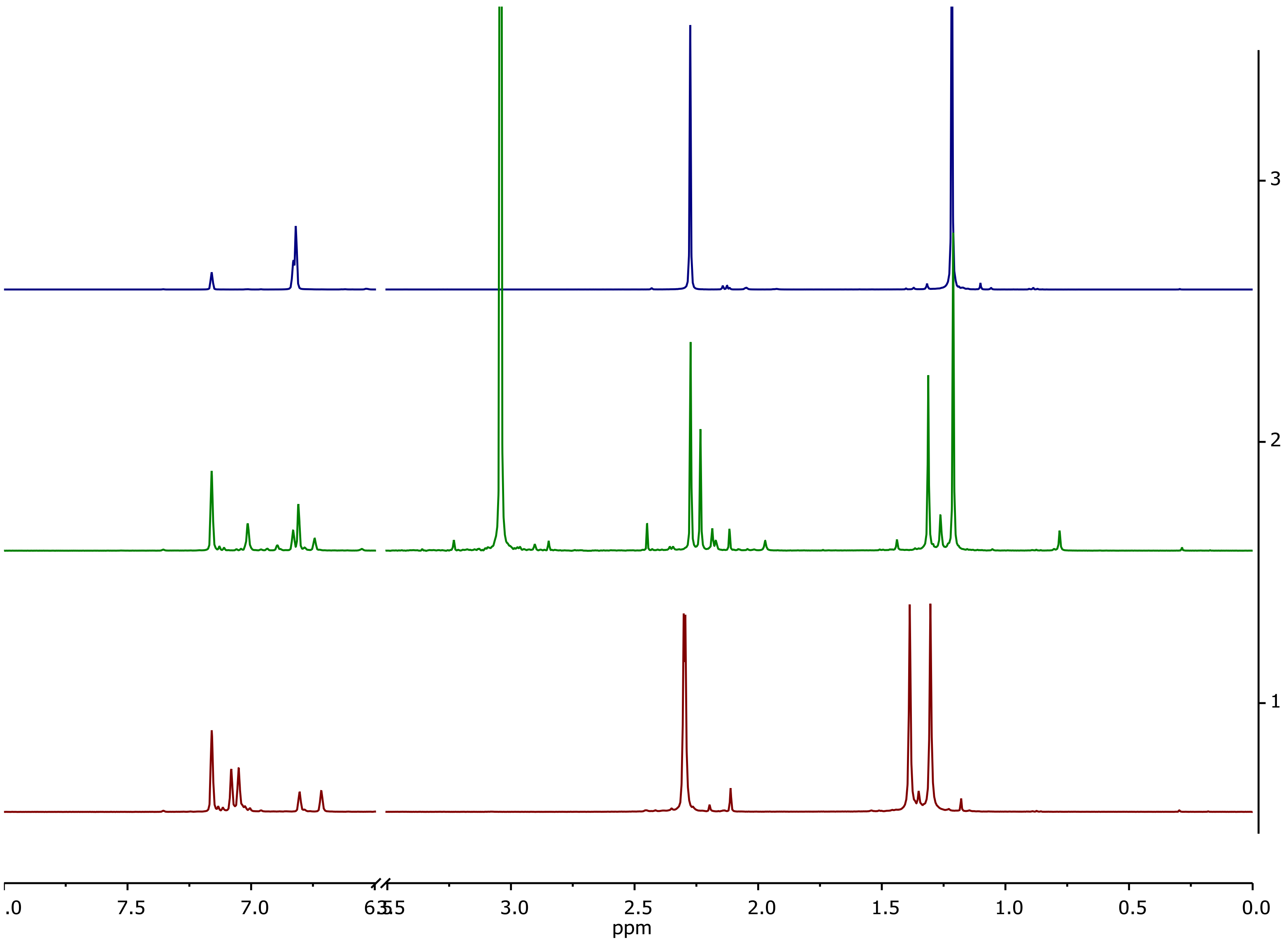

*Figure S 66: Stacked ^{1}H NMR spectra of **2-PP** (bottom, red), a NMR test reaction of **2-PP** with methyl triflate in C_6D_6 after 30min at ambient temperature (middle, green), and isolated **1-OTf** (top, blue). The region between 6.5 and 3.5 ppm was emitted for clarity. The signal at 3 ppm in the green spectra belongs to unreacted methyl triflate.*

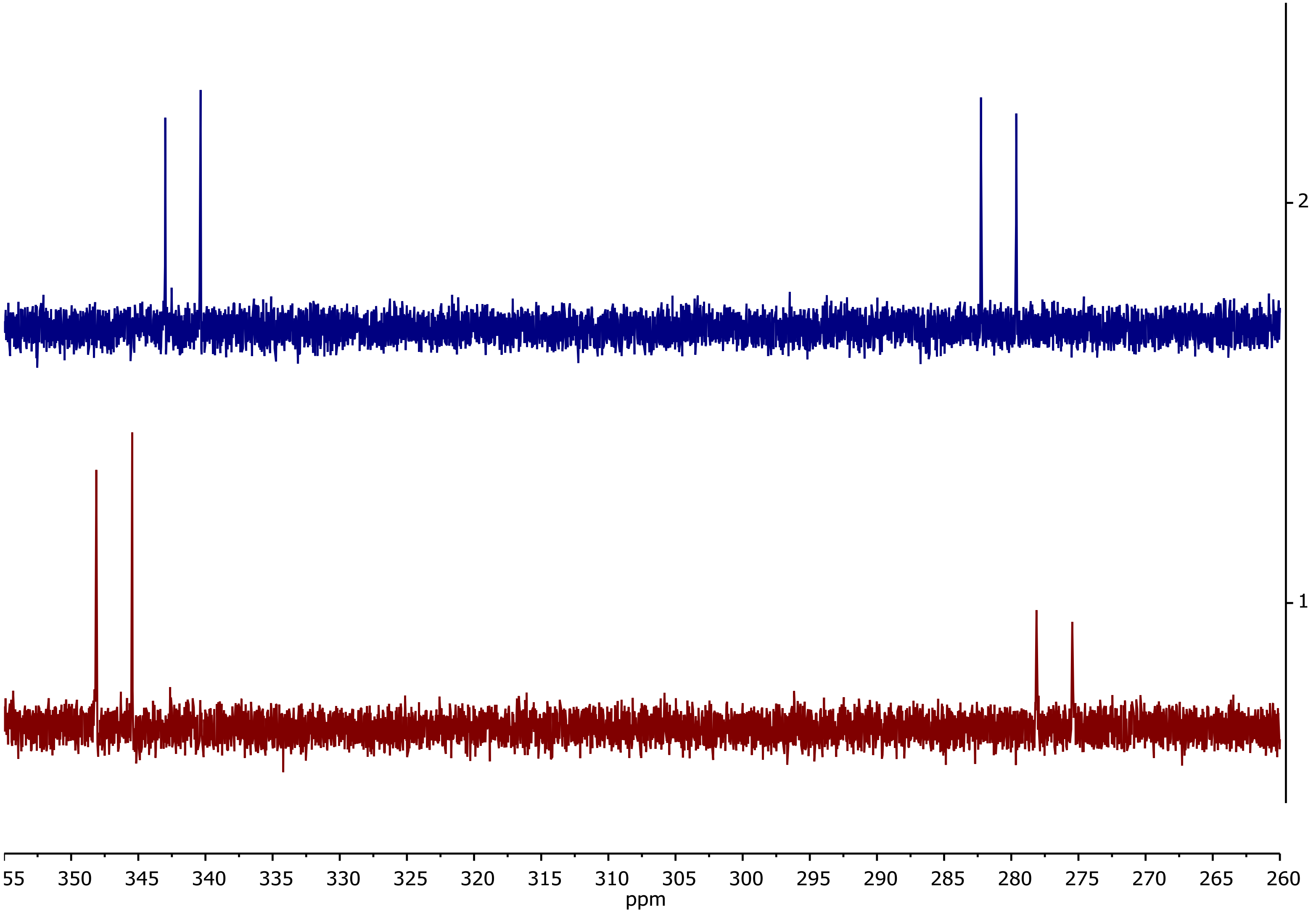

*Figure S 67: Stacked ^{31}P NMR spectra of **2-PP** (bottom, red), and a NMR test reaction of **2-PP** with methyl triflate in C_6D_6 after 30min at ambient temperature (top, blue).*

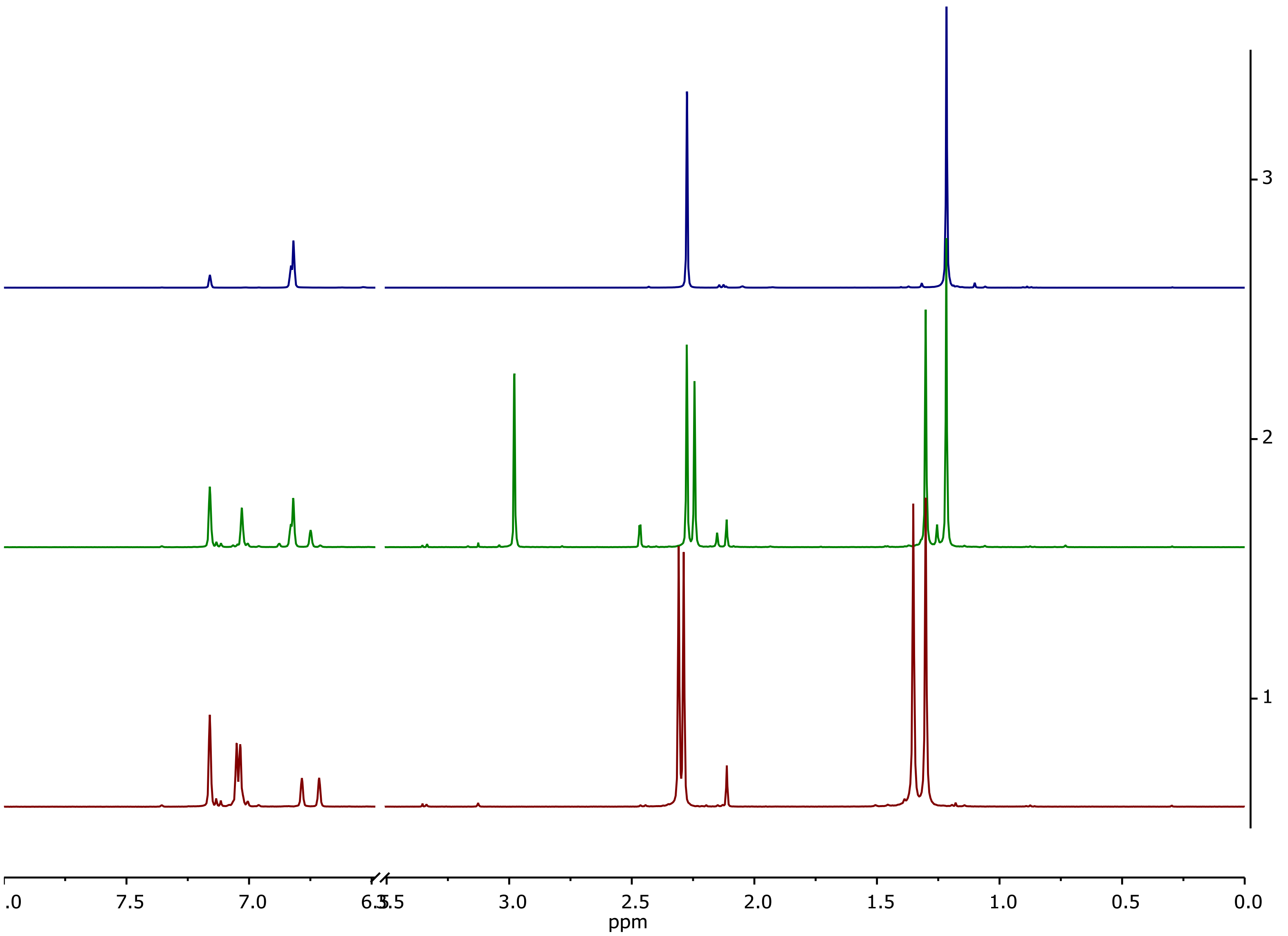

*Figure S 68: Stacked ^{1}H NMR spectra of **2-SP** (bottom, red), a NMR test reaction of **2-SP** with methyl triflate in C_6D_6 after 30min at ambient temperature (middle, green), and isolated **1-OTf** (top, blue). The region between 6.5 and 3.5 ppm was emitted for clarity.*

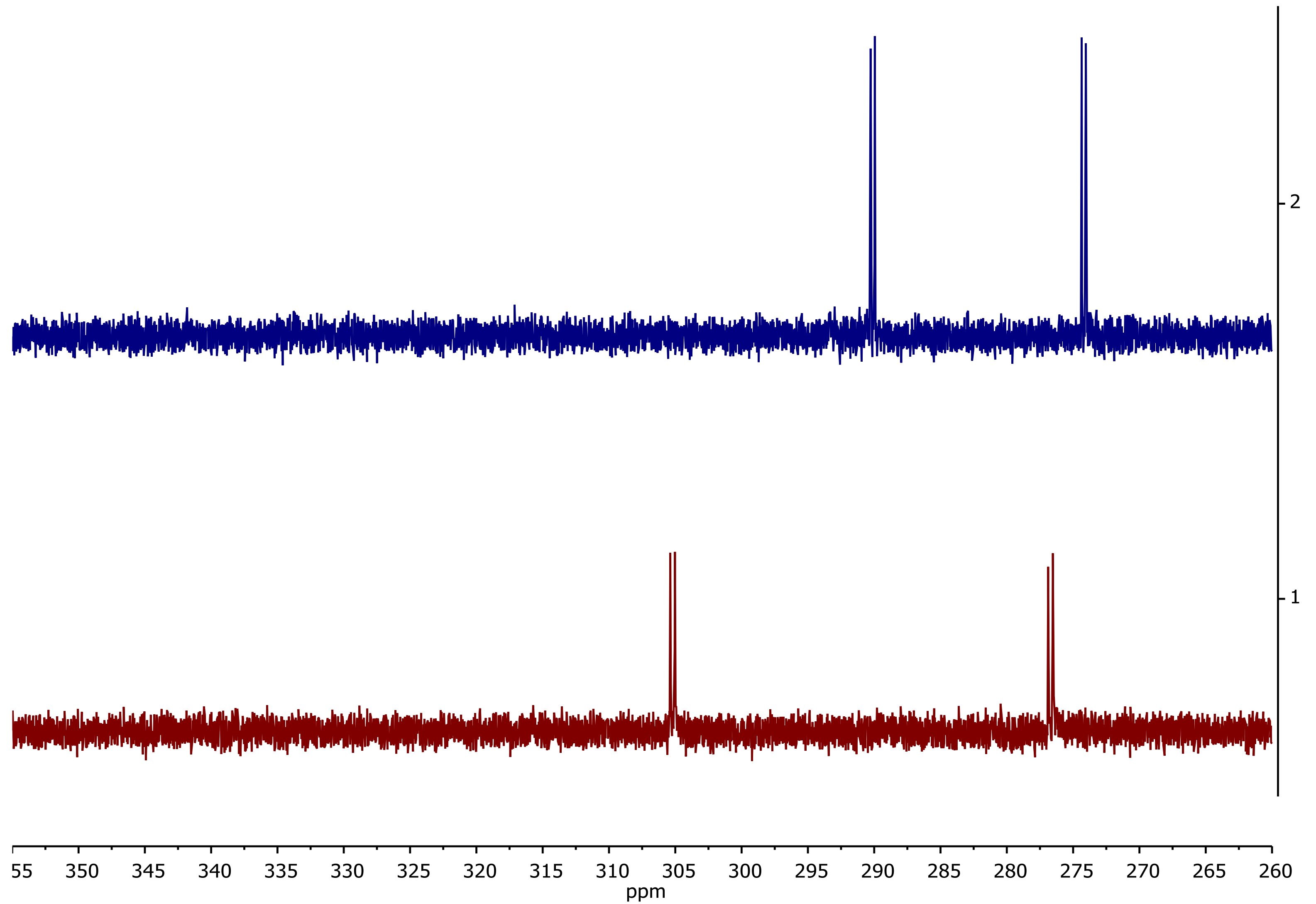

*Figure S 69: Stacked ^{31}P NMR spectra of **2-SP** (bottom, red), and a NMR test reaction of **2-SP** with methyl triflate in C_6D_6 after 30min at ambient temperature (top, blue).*

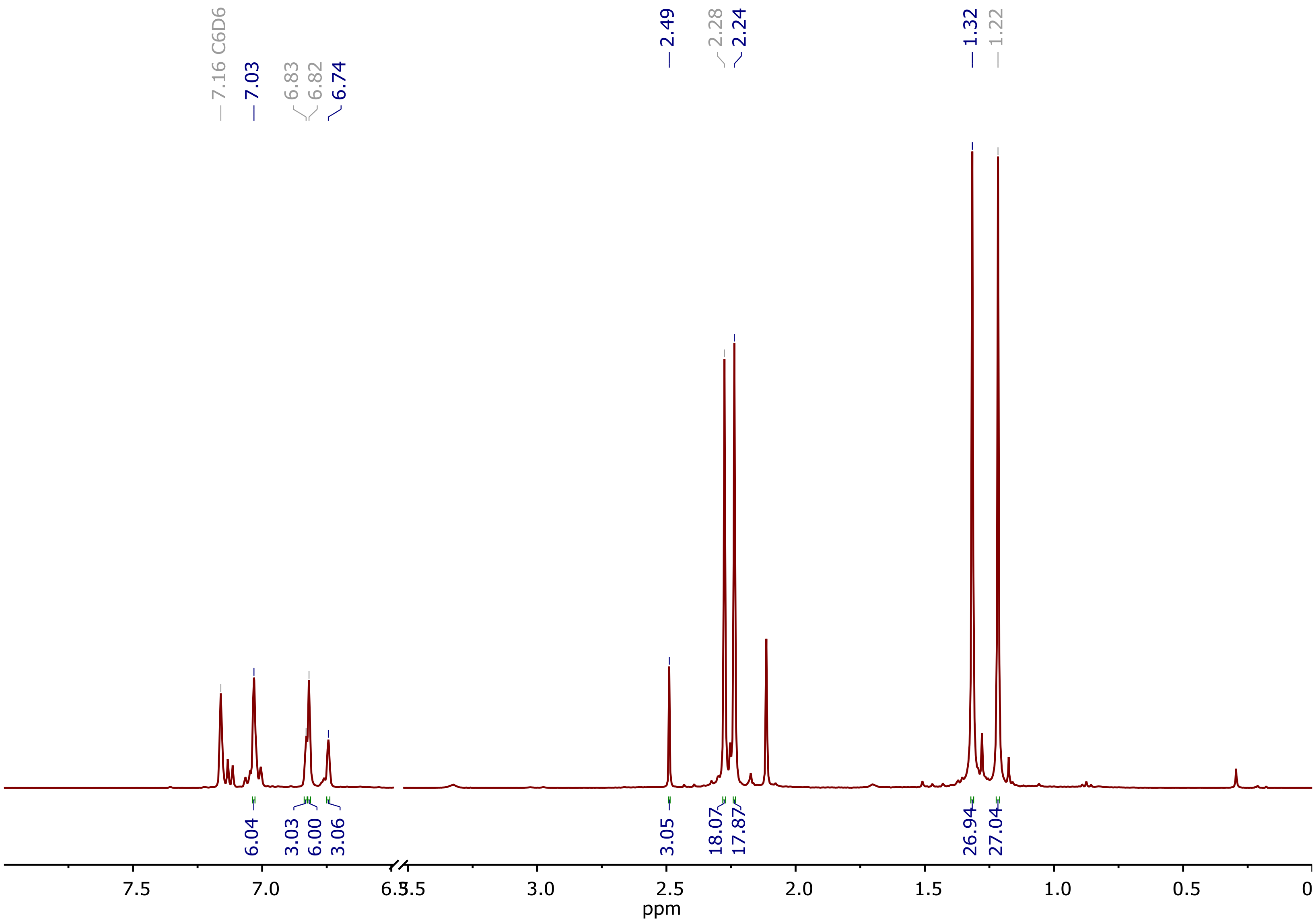

*Figure S 70: Crude ^{1}H NMR spectrum of the reaction of **3-AsAs** with methyl triflate. Signals resulting from **1-OTf** (6.83, 6.82, 2.28, and 1.22 ppm) are marked in grey. The region between 6.5 and 3.5 is emitted for clarity.*

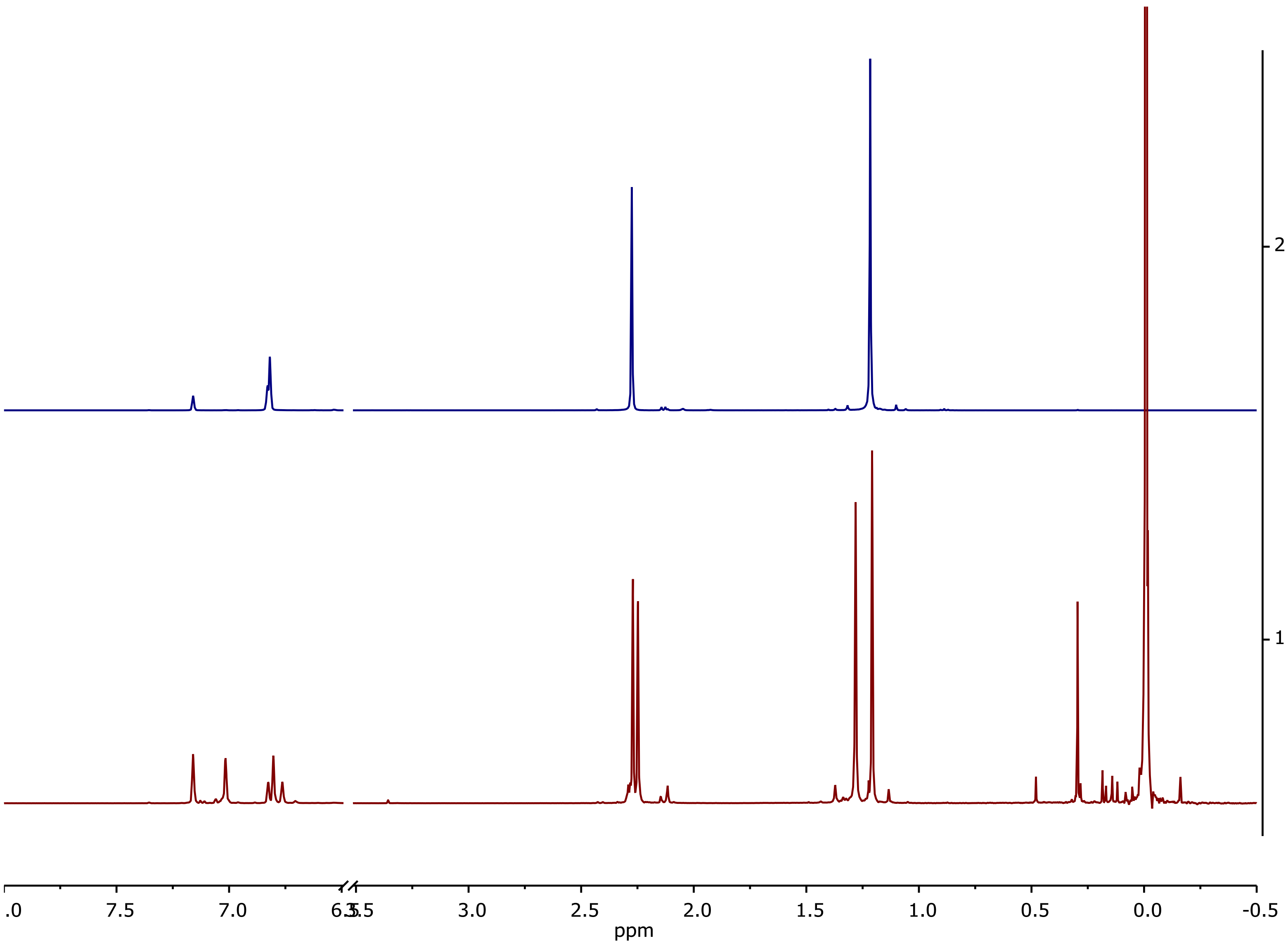

*Figure S 71: Stacked ^{1}H NMR spectra of a mixture of **2-PP** and TMS-OTf after 16 h (bottom, red), and isolated **1-OTf** (top,blue) indicating cleavage of the Zr-S bond.*

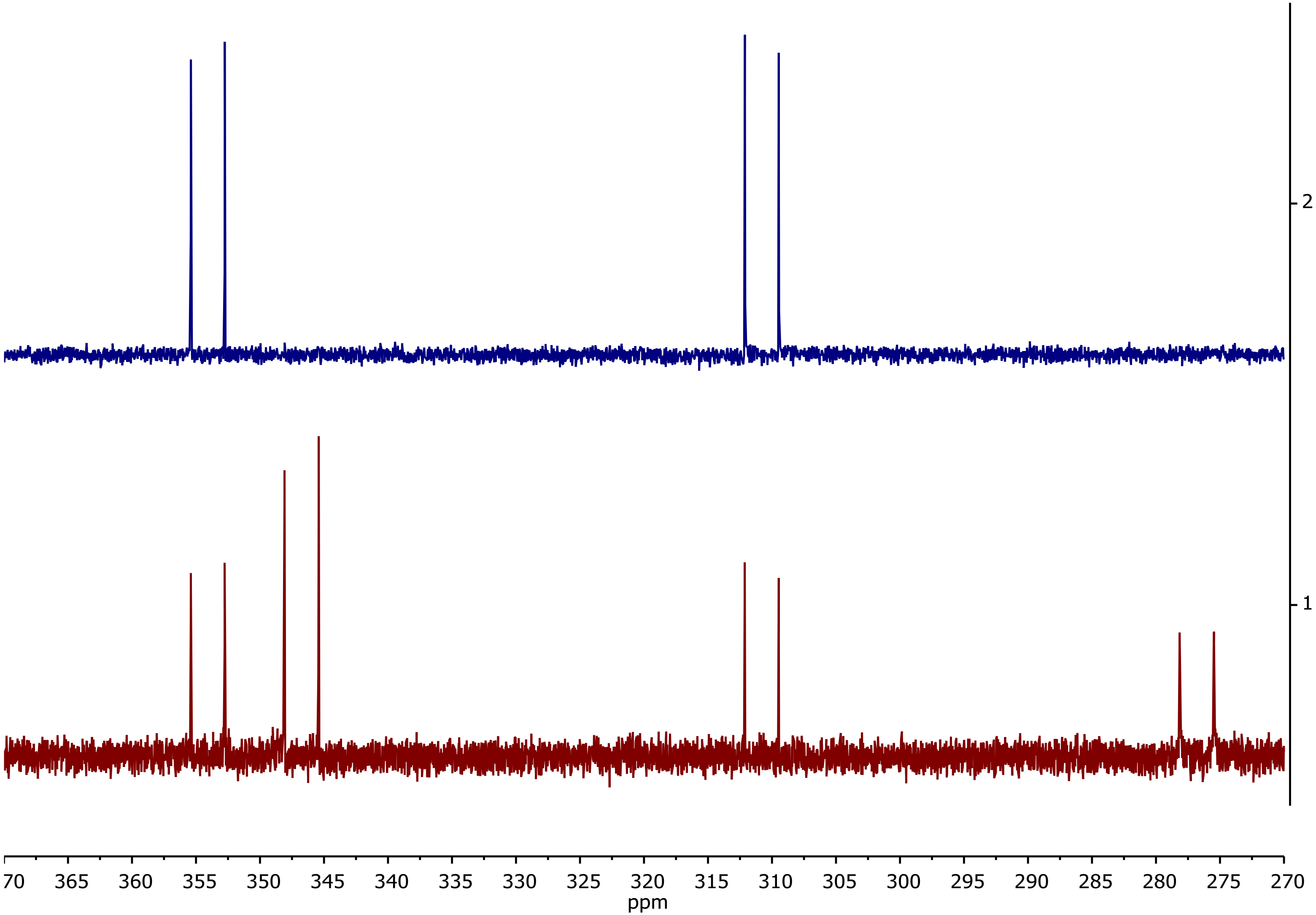

*Figure S 72: Stacked ^{31}P NMR spectra of a mixture of **2-PP** and TMS-OTf after 2 h (bottom, red) and 16 h (top,blue).*

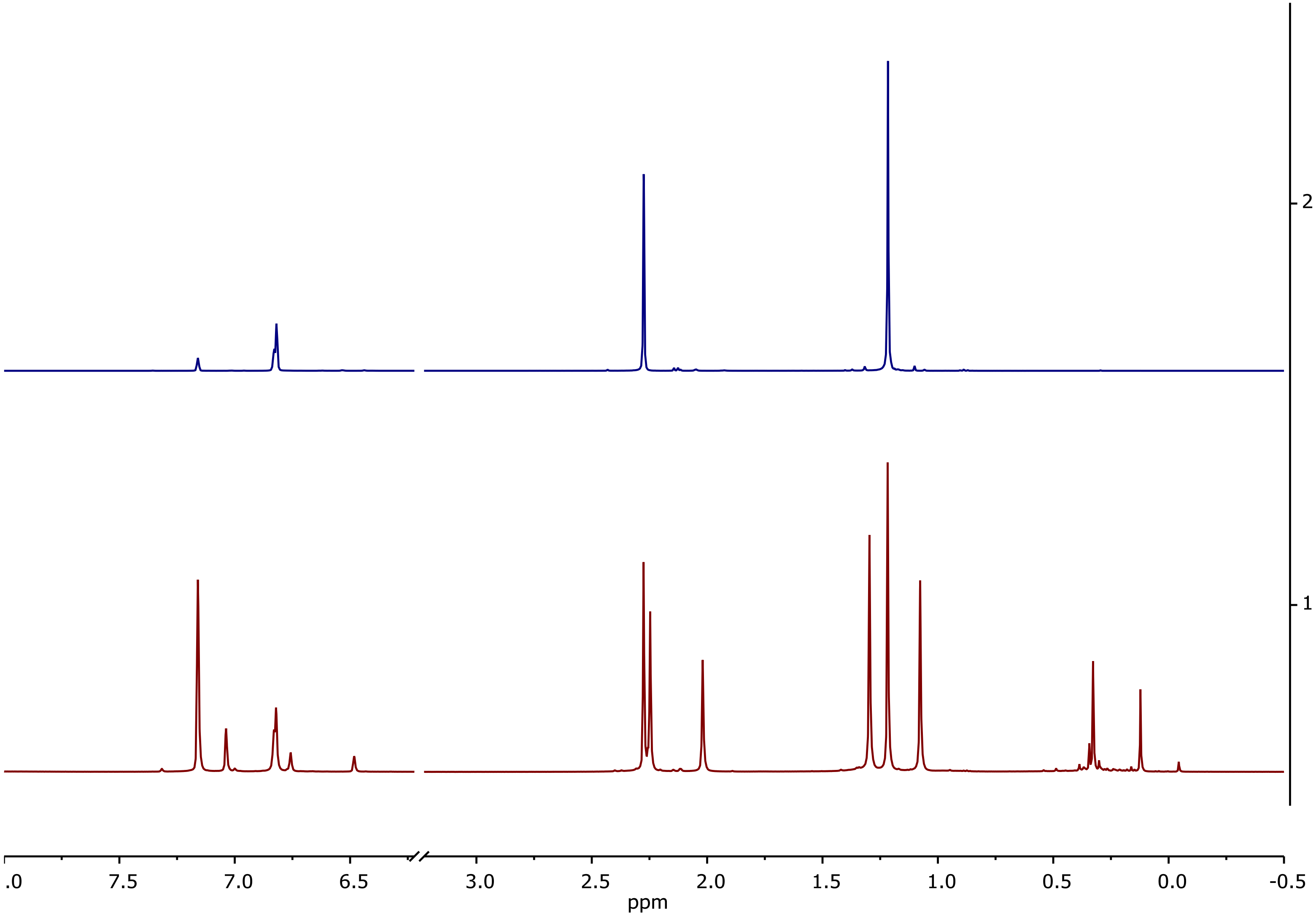

*Figure S 73: Stacked ¹H NMR spectra of the crude reaction product of **2-SP** and TMS-OTf (bottom, red), and isolated **1-OTf** (top,blue) indicating cleavage of the Zr-S bond.*

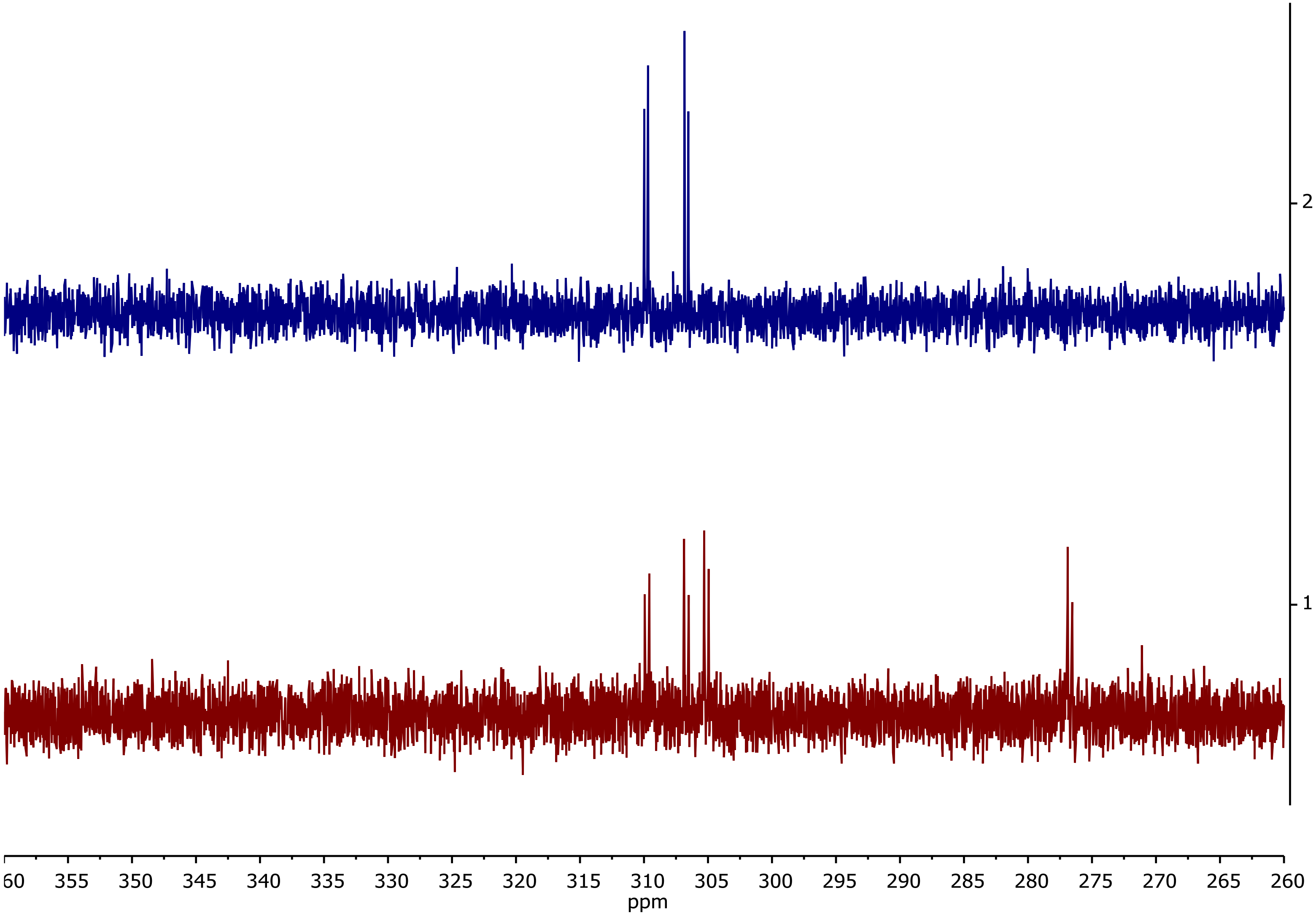

*Figure S 74: Stacked ^{31}P NMR spectra of a mixture of **2-SP** and TMS-OTf after 16 h (bottom, red) and its crude reaction product (top,blue).*

IR spectroscopy

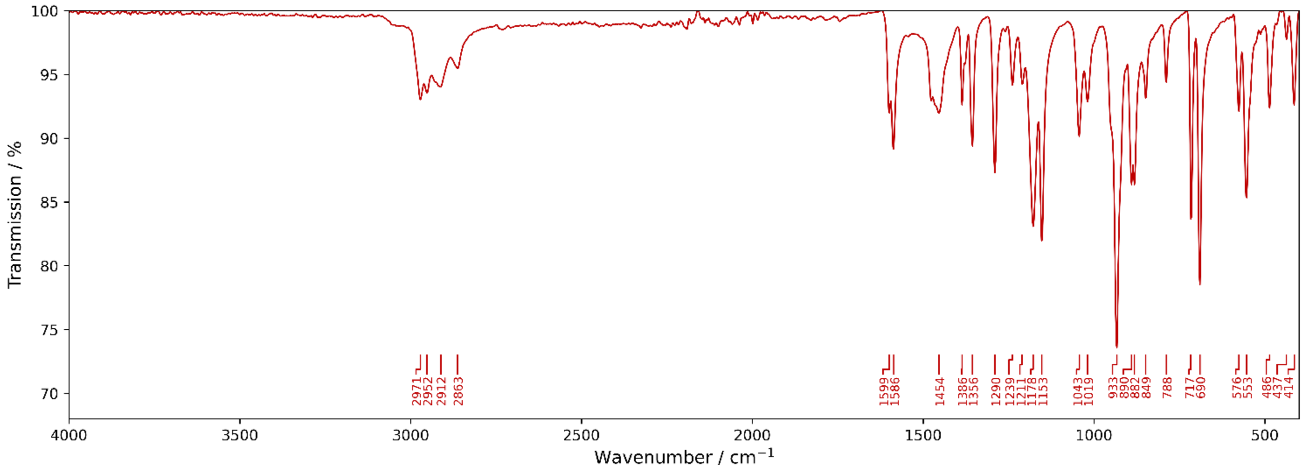

*Figure S 75: IR spectrum of **1-Cl**.*

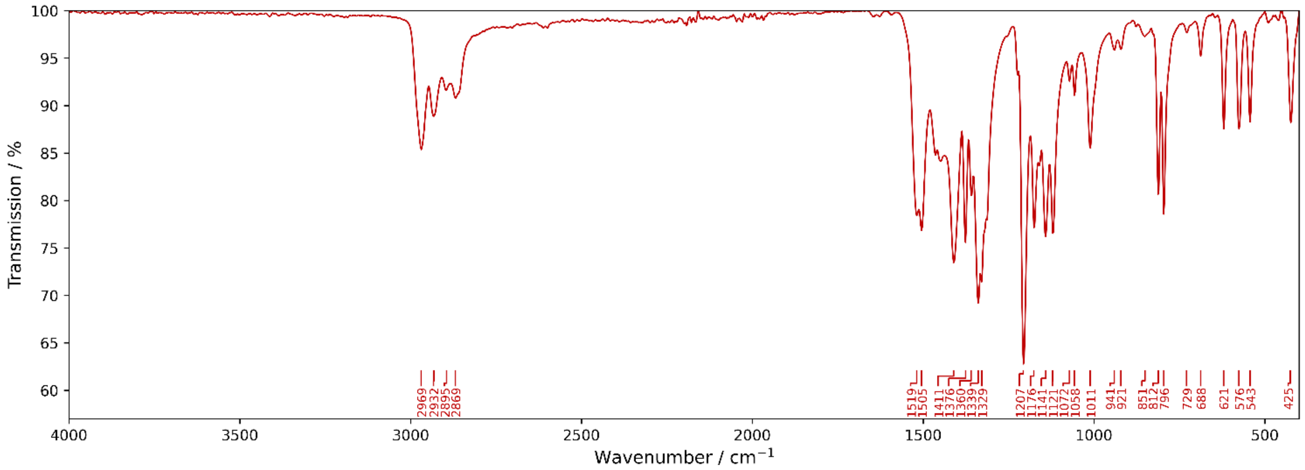

*Figure S 76: IR spectrum of **1-I**.*

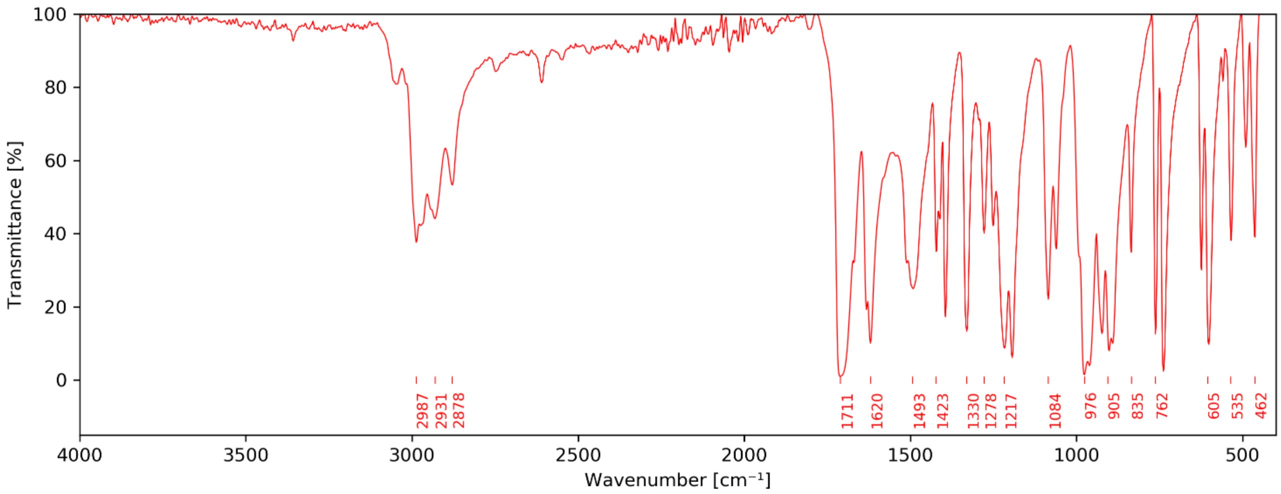

*Figure S 77: IR spectrum of **1-OCP**.*

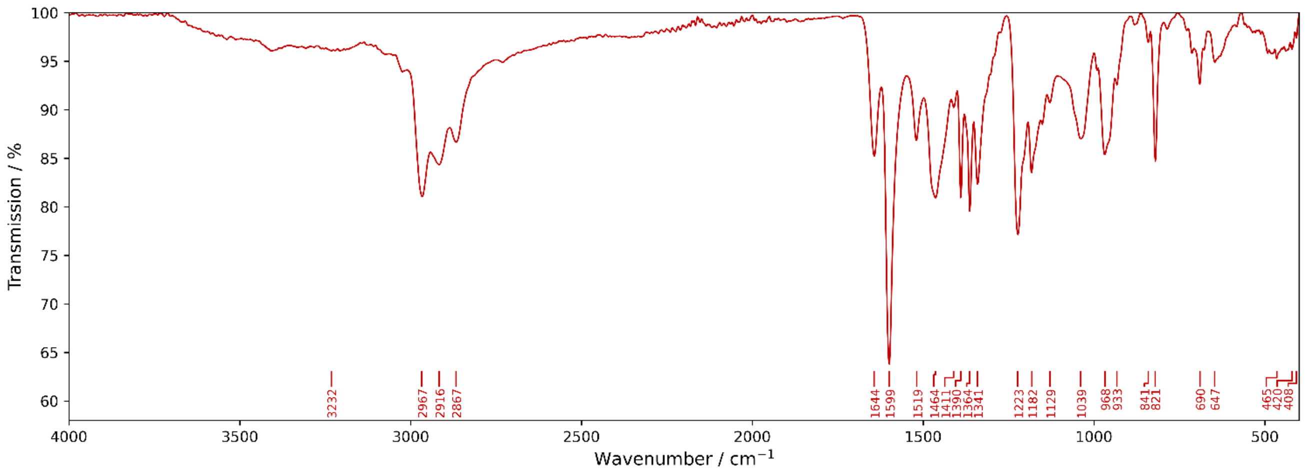

*Figure S 78: IR spectrum of **2-PP**.*

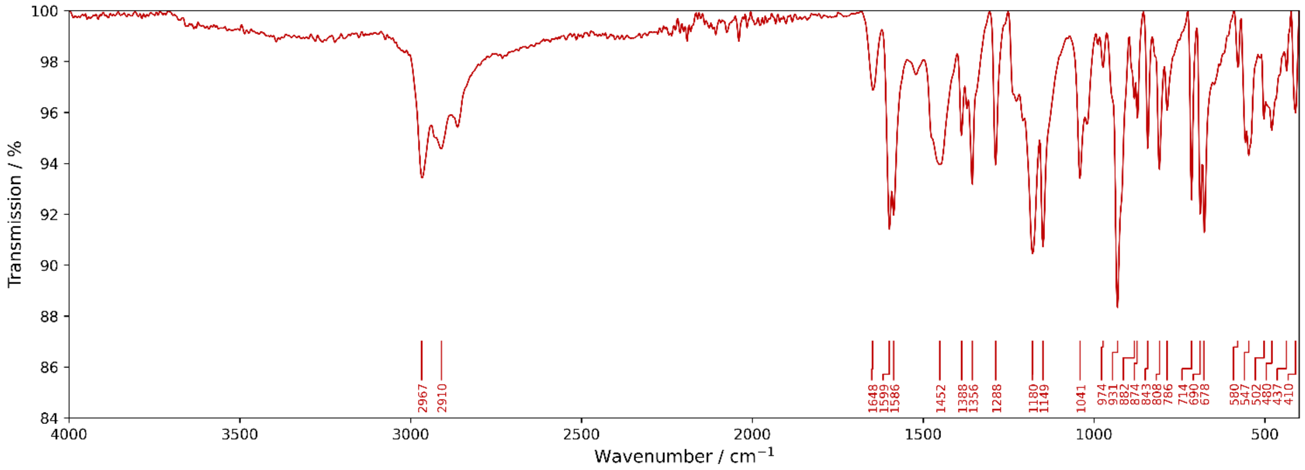

*Figure S 79: IR spectrum of **2-SP**.*

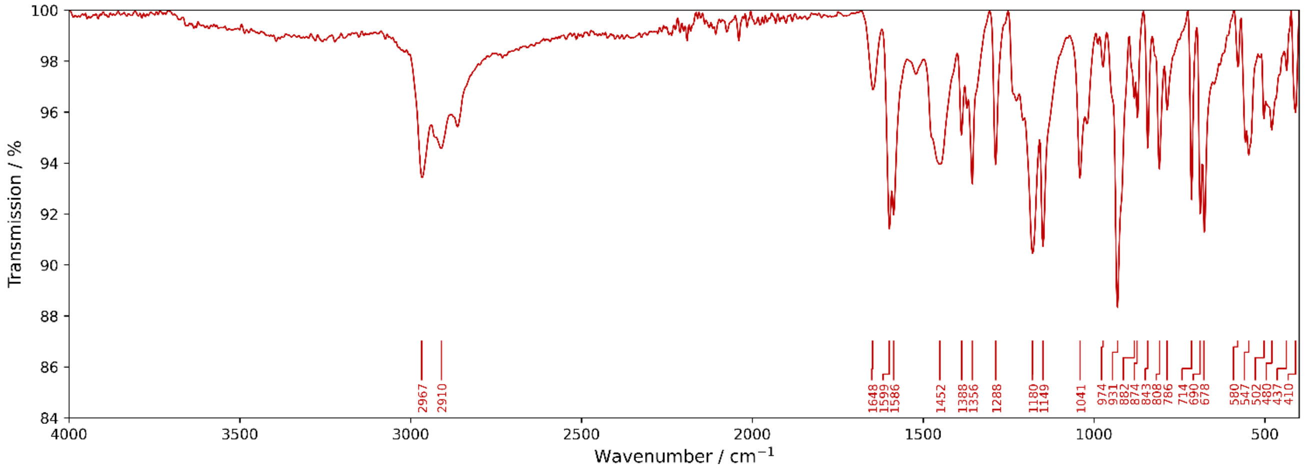

*Figure S 80: IR spectrum of **3-AsAs**.*

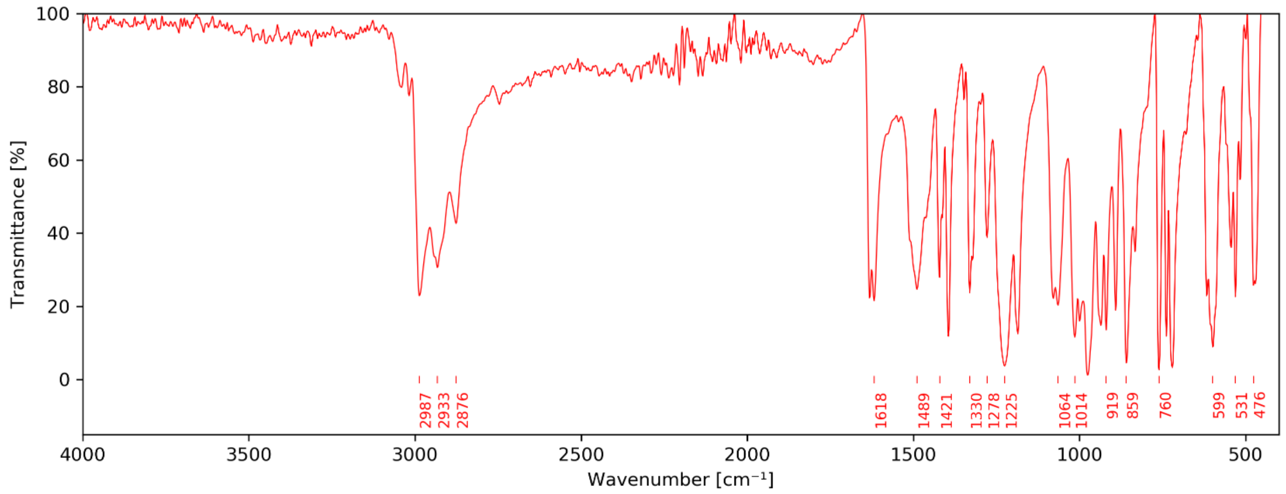

*Figure S 81: IR spectrum of **4-PP**.*

UV-VIS-NIR Spectroscopy

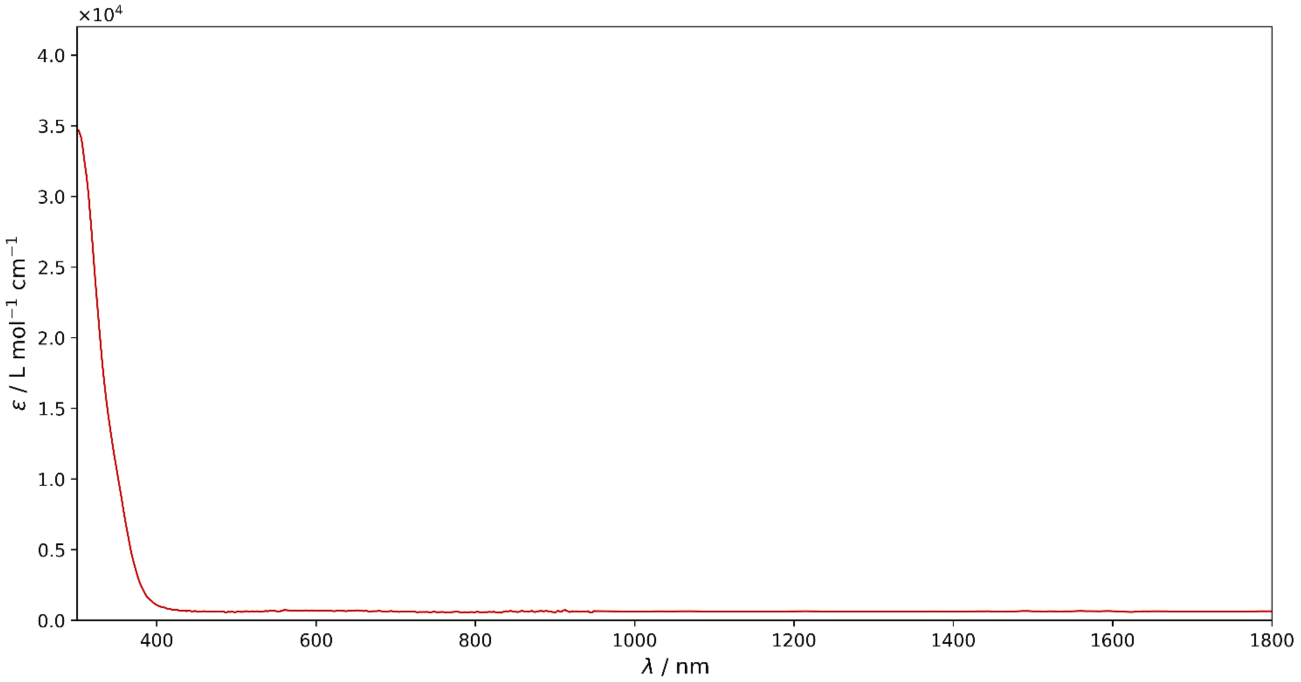

*Figure S 82: UV-Vis-NIR spectrum of **1-Cl** in toluene at 298 K.*

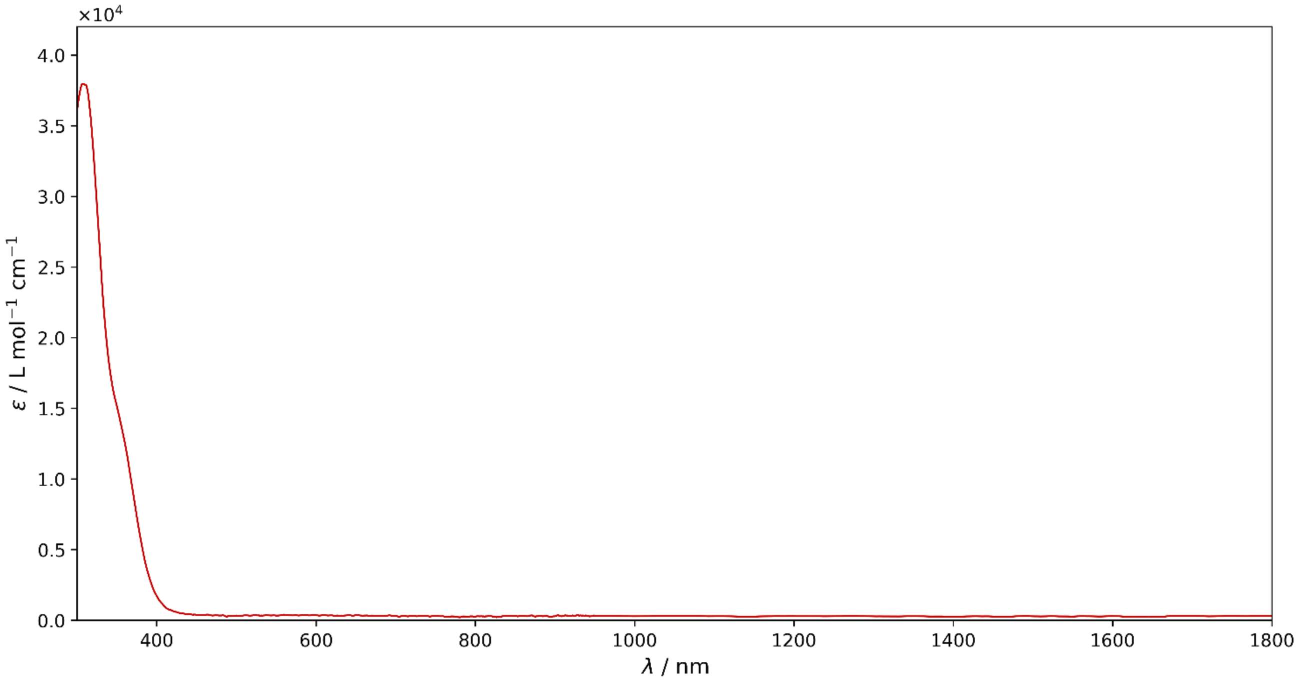

*Figure S 83: UV-Vis-NIR spectrum of **1-I** in toluene at 298 K.*

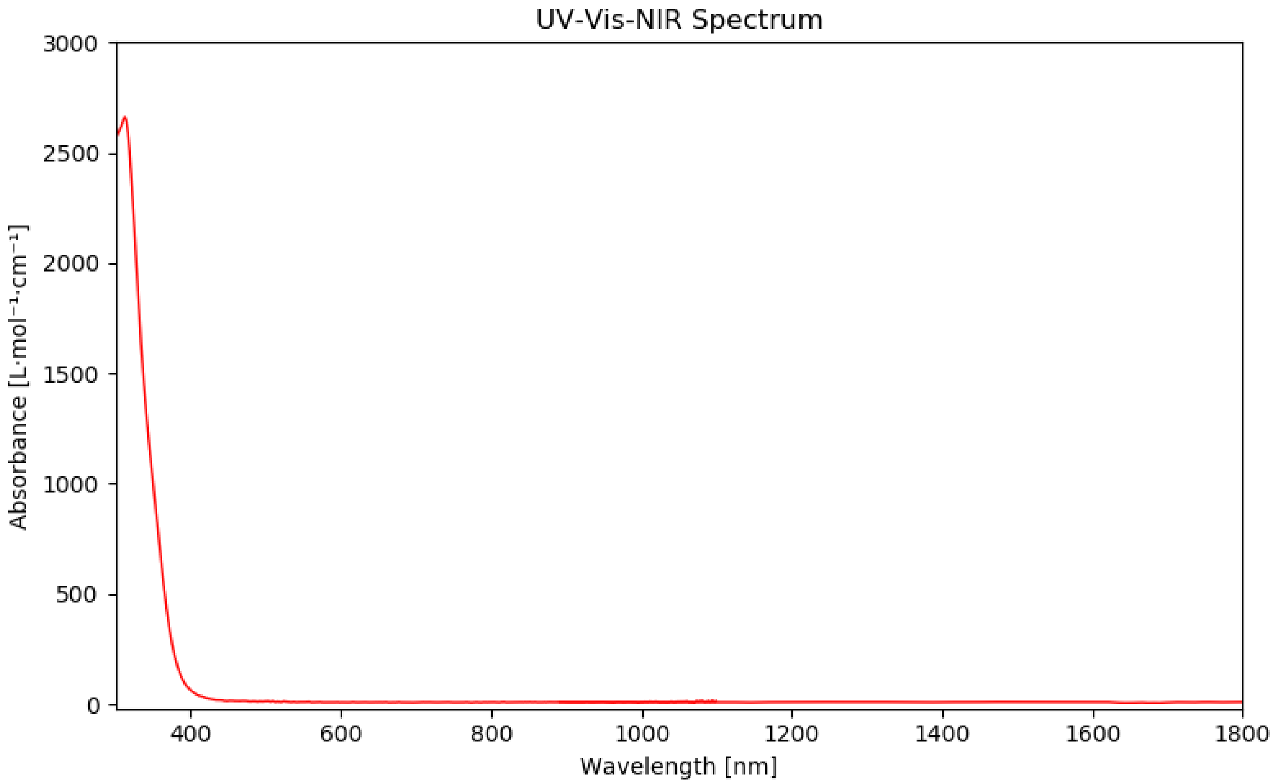

*Figure S 84: UV-Vis-NIR spectrum of **1-OCP** in toluene at 298 K.*

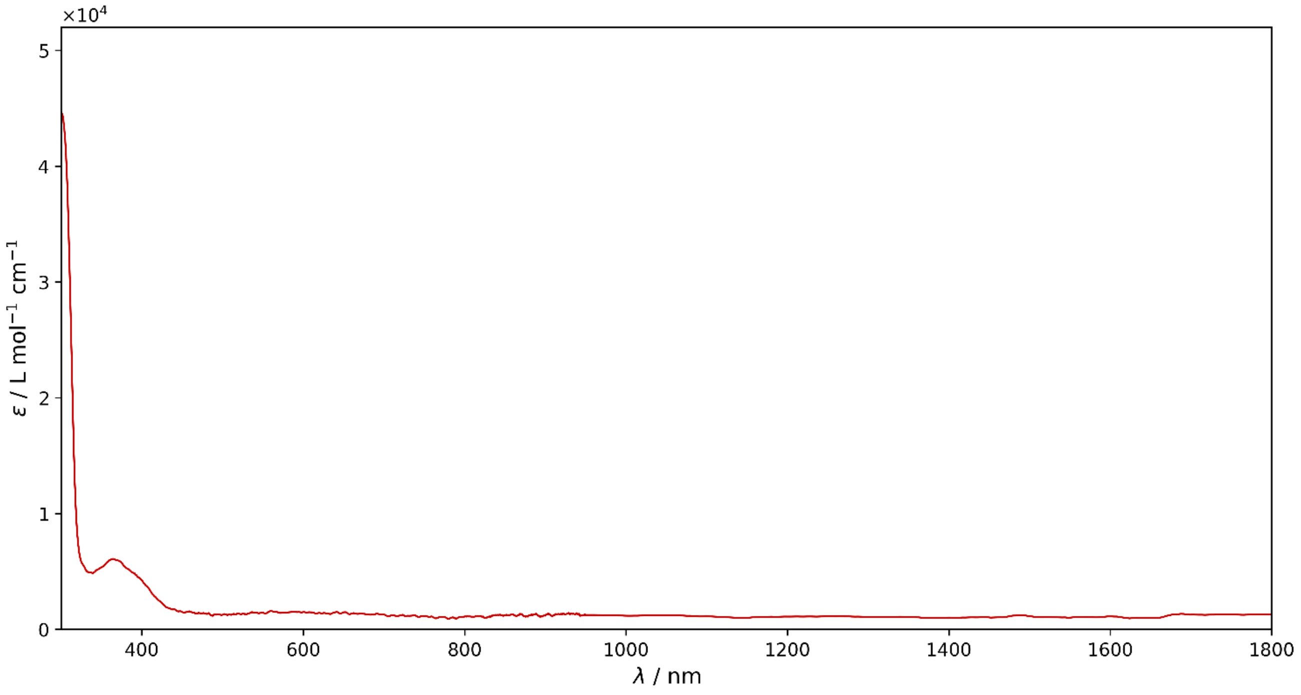

*Figure S 85: UV-Vis-NIR spectrum of **2-PP** in toluene at 298 K.*

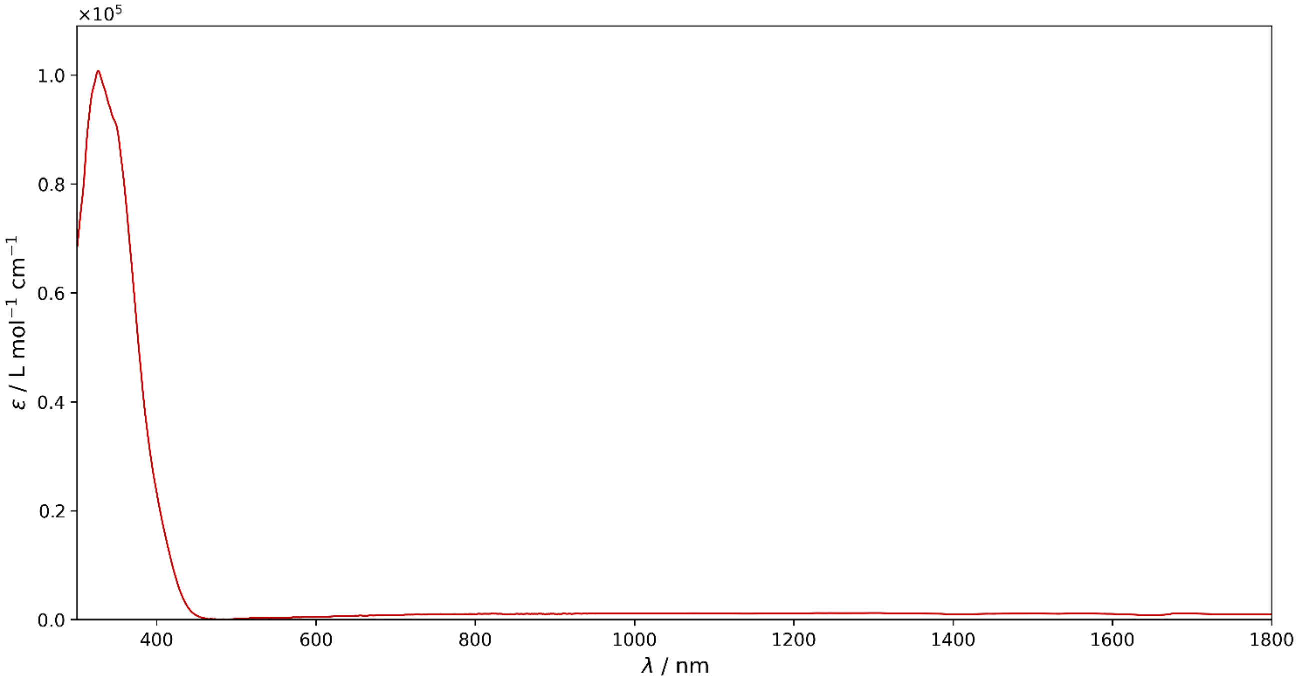

*Figure S 86: UV-Vis-NIR spectrum of **2-SP** in toluene at 298 K.*

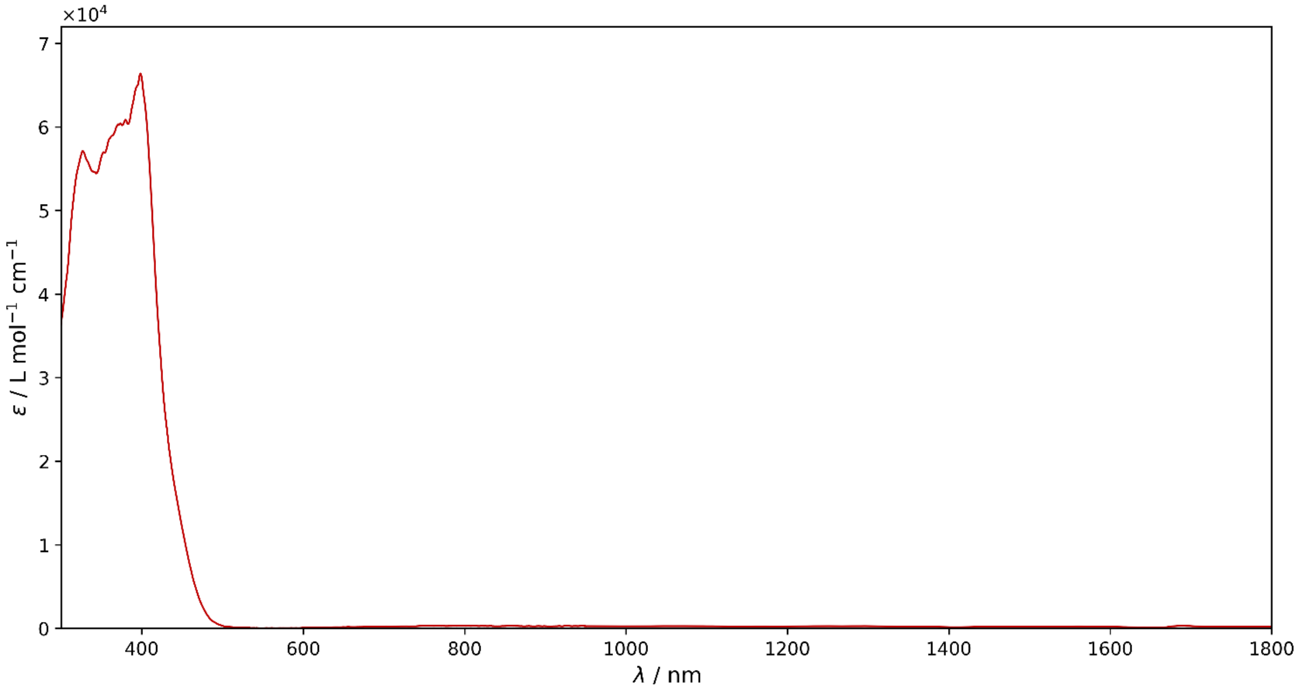

*Figure S 87: UV-Vis-NIR spectrum of **3-AsAs** in toluene at 298 K.*

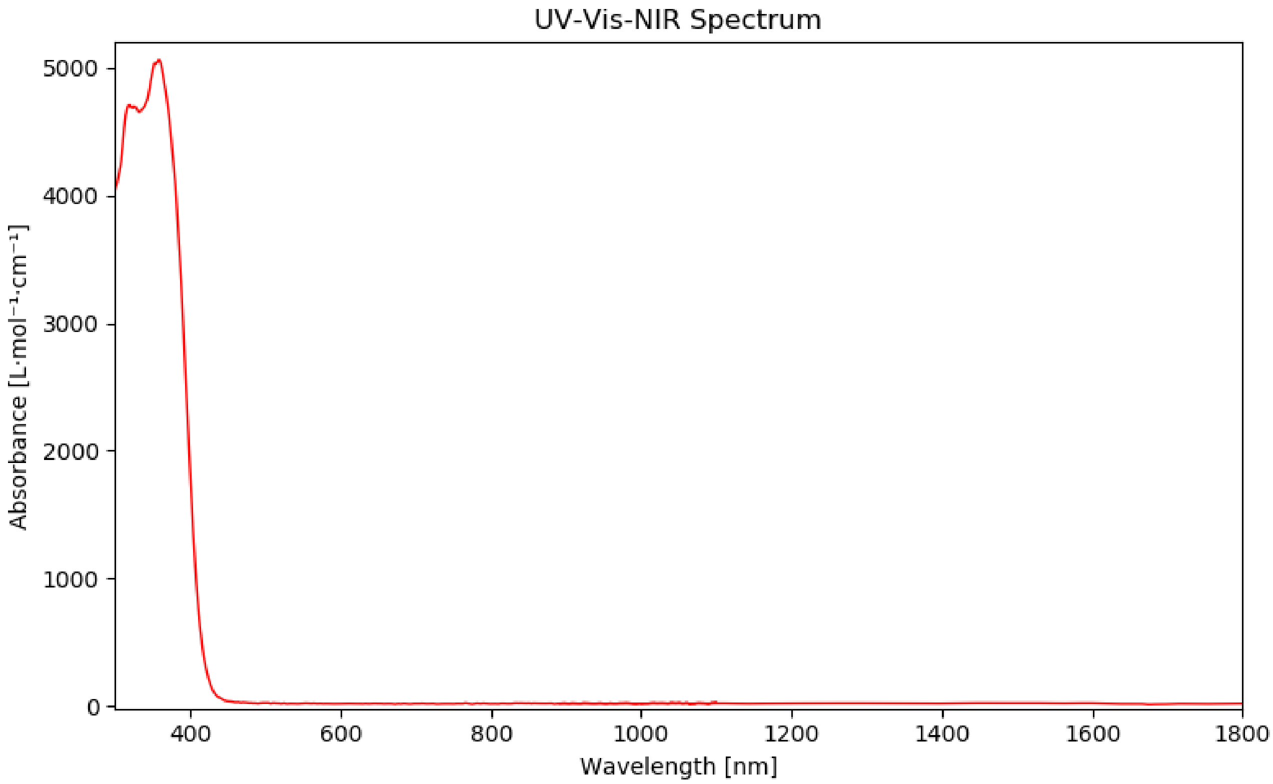

*Figure S 88: UV-Vis-NIR spectrum of **4-PP** in toluene at 298 K.*

Crystallographic Details

*Table S 1: Crystallographic Details of Complexes **1-Cl, 1-I, 2-PP, 2-SP, 3-AsAs** and **3-SAs**.*

	**1-Cl**	**1-I**	**1-OCP**	**1-OTf**	**2-PP**	**2-SP**	**3-AsAs**	**3-SAs**	**4-PP**	**5-AsAs**
Chemical Formula	$C_{36}H_{54}N_3Cl_1Zr_1$	$C_{36}H_{54}N_3I_1Zr_1$	$C_{37}H_{54}N_3O_1P_1Zr_1$	$C_{37}H_{54}N_3O_3F_3S_1Zr_1$	$C_{74}H_{108}N_6P_2S_2Zr_2$	$C_{74}H_{108}N_6P_2S_2Zr_2$	$C_{74}H_{108}N_6S_2As_2Zr_2$	$C_{74}H_{108}N_6S_2As_2Zr_2$	$C_{39}H_{57}N_3P_2S_2Zr_1$	$C_{39}H_{57}N_3S_2As_2Zr_1$
M_r (g mol^{-1})	655.49	746.94	679.02	769:11	1390.16	1390.16	1478.06	1478.145	785.15	873.05
Crystal System	Monoclinic	Triclinic	Monoclinic	Triclinic	Triclinic	Triclinic	Triclinic	Triclinic	Monoclinic	Monoclinic
Space Group	$P2_1/c$	*P*-1	$C2/c$	*P*-1	*P*-1	*P*-1	*P*1	*P*-1	$P2_1/c$	$P2_1/c$
a (Å)	19.1293(7)	9.5380(3)	37.524(3)	10.7189(2)	11.0494(4)	11.0749(16)	11.1162(4)	11.1089(5)	11.2482(3)	11.3021(2)
b (Å)	11.0969(3)	11.3387(4)	10.6629(9)	15.5552(4)	12.8302(5)	12.782(2)	12.8946(6)	12.8559(6)	19.3079(7)	19.3382(5)
c (Å)	18.0617(6)	16.5498(6)	23.695(2)	24.4893(5)	13.8673(4)	13.9799(19)	13.8469(6)	13.9037(7)	19.0553(7)	19.0991(6)
α (°)	90	80.7500(10)	90	93.485(2)	89.010(3)	88.595(4)	89.101(2)	88.663(2)	90	90
β (°)	111.1120(10)	88.1760(10)	128.132(2)	90.244(2)	84.982(3)	84.512(4)	85.031(2)	84.689(2)	92.7490(10)	92.8780(10)
γ (°)	90	89.1030(10)	90	107.966(2)	74.140(3)	74.066(4)	74.376(2)	74.352(2)	90	90
V (Å^3)	3576.5(2)	1765.57(11)	7457.6(11)	3875.79(15)	1883.78(12)	1894.2(5)	1904.22(14)	1903.85(16)	4133.7(2)	4169.08(19)
Z	4	2	8	4	1	1	1	1	4	4
Density (g cm^{-3})	1.217	1.405	1.210	1.318	1.225	1.219	1.289	1.289	1.262	1.391
F(000)	1392	768	2880	1616	736	736	772	766.859	1656	1800
Radiation Type	MoKα	MoKα	MoKα	MoKα	MoKα	MoKα	MoKα	MoKα	MoKα	MoKα
μ (mm^{-1})	0.409	1.213	0.368	0.389	0.417	0.415	1.233	1.233	0.474	1.968
Crystal Size (mm)	0.20x0.18x0.10	0.12x0.11x0.01	0.37x0.34x0.33	0.23x0.19x0.19	0.11x0.07x0.03	0.08x0.07x0.02	0.24x0.20x0.15	0.15x0.09x0.08	0.36x0.18x0.07	0.17x0.16x0.11
Meas. Refl.	89309	91367	110612	34141	18758	54778	86819	99582	141569	57901
Indep. Refl.	7898	7866	14935	34141	9217	6706	13501	8141	9491	8845
Obsvd. $[I > 2\sigma(I)]$	6419	7092	10319	26958	7535	4784	12783	7390	8246	7948
R_{int}	0.1112	0.0500	0.0913	-	0.0380	0.1635	0.0439	0.0875	0.0785	0.0466
R [$F^2 > 2\sigma(F^2)$]	0.0391	0.0376	0.0450	0.0613	0.0470	0.0493	0.0258	0.0445	0.0279	0.00321
wR(F^2)	0.0927	0.0943	0.1034	0.1310	0.1094	0.1218	0.0548	0.1052	0.0734	0.0868
S	1.050	1.094	1.023	1.106	1.091	1.011	1.027	1.0058	1.044	1.068
$\Delta\rho_{max}$	0.389	1.099	0.996	0.715	0.554	0.689	0.269	0.6409	0.393	0.883
$\Delta\rho_{min}$	-0.460	-1.731	-1.068	-0.976	-0.867	-0.504	-0.175	-0.7089	-0.387	-0.737
CCDC	2365792	2365790	2518191	2526018	2365786	2365793	2365791	2365789	2526017	2530439

*Table S 2: Selected bond lengths and angles for the complexes **1-Cl, 1-I, 2-PP, 2-SP, 3-AsAs** and **3-SAs**.*

**Atoms**	**1-Cl**	**1-I**	**1-OCP**	**1-OTf**	**2-PP**	**2-SP**	**3-AsAs**	**3-SAs**	**4-PP**	**5-AsAs**
Zr1 – N10	2.0518(19)	2.067(3)	2.0483(14)	2.041(2)	2.046(2)	2.073(3)	2.078(4)	2.074(2)	2.0682(13)	2.072(2)
Zr1 – N30	2.0523(19)	2.072(3)	2.0533(13)	2.041(2)	2.072(2)	2.050(3)	2.046(4)	2.055(3)	2.0624(13)	2.062(2)
Zr1 – N50	2.0665(18)	2.047(3)	2.0510(14)	2.031(2)	2.048(2)	2.057(3)	2.057(4)	2.057(2)	2.0440(14)	2.051(2)
Zr1 – X1*	2.4226(6)	2.8490(4)	2.0590(13)	2.101(2)	-	-	-	-	-	
Zr1 – S1	-	-	-	-	2.589(1)	2.554(1)	2.570(2)	2.467(16)	-	
Zr1 – C2	-	-	-	-	2.294(5)	2.297(8)	2.294(8)	2.43(3)	2.3194(16)	2.305(2)
S1 – C1	-	-	-	-	1.753(5)	1.752(8)	1.736(8)	1.77(2)	1.7491(17)	1.748(3)
C1 – E1**	-	-	-	-	1.710(6)	1.728(8)	1.862(10)	1.77(2)	1.7131(17)	1.846(3)
E1 – E2**	-	-	-	-	2.123(3)	-	2.3353(11)	-	2.1378(6)	2.3460(4)
E1 – S2**	-	-	-	-	-	2.074(5)	-	2.243(15)	-	-
E2 – C2**	-	-	-	-	1.712(6)	1.685(10)	1.839(10)	1.82(2)	1.7117(17)	1.849(2)
S2 – C2	-	-	-	-	1.684(6)	1.727(10)	1.675(10)	1.80(3)	1.6882(16)	1.673(2)
E2 – C1	-	-	-	-	-	1.764(9)	-	1.933(17)	-	-
S2 – C1	-	-	-	-	1.736(5)	-	1.712(8)	-	1.7219(17)	1.707(3)
S1 – C3									1.787(2)	1.780(3)
Zr1/C3 – S1 – C1	-	-	-	-	104.1(1)	109.0(1)	105.2(3)	109.1(12)	102.13(69)	102.74(15)
S1 – C1 – E1**	-	-	-	-	125.5(3)	122.5(5)	124.5(5)	122.0(14)	126.90(10)	125.74(15)
S2 – C2 – E2**	-	-	-	-	118.3(3)	117.7(5)	120.1(5)	131.3(17)	117.48(9)	118.41(13)
τ_4'	0.98	0.92	0.96	0.93	0.93/0.88	0.92/0.90	0.95/0.89	0.94/0.89	0.95	0.94

*X = Cl ,I, O
** E = P, As

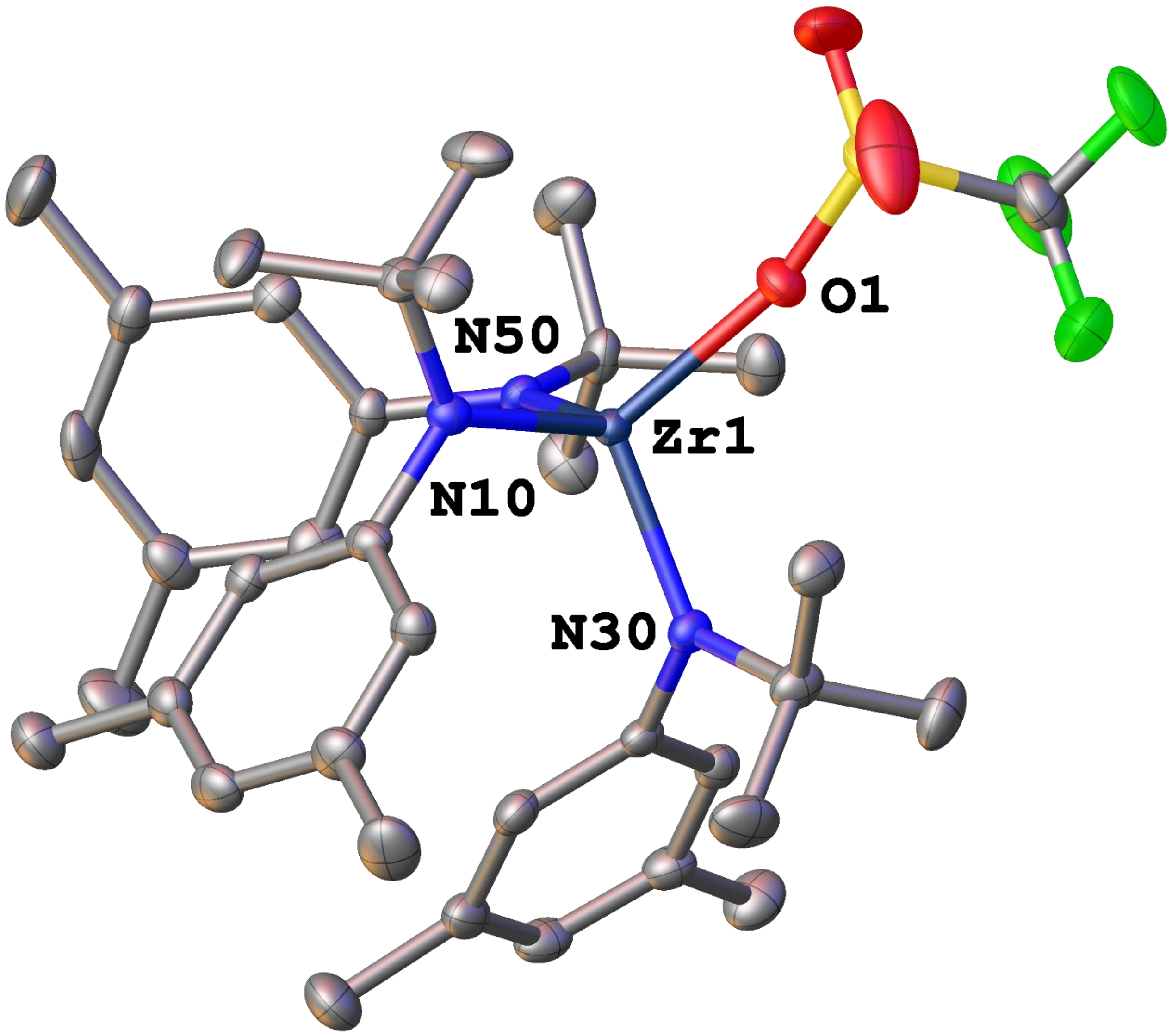

*Figure S 89: Molecular structure of the triflato complex **1-OTf**. Hydrogen atoms are omitted for clarity and ellipsoids are shown at a probability level of 50%.*

Computational Details / Methods

Calculations were done with TURBOMOLE[9] employing the PBE functional.[10] All structure optimizations were done with dhf-SV(P) basis sets,[11] with fine grids (gridsize 5)[12] and using weight derivatives. Localized molecular orbitals (LMOS) were obtained by the Pipel-Mezey procedure,[13] atomic contributions to the individual LMOs by Mulliken population analyses.[14] Results are shown in Fig. S46. ^{31}P NMR shifts[15] (w.r.t. H_3PO_4) and coupling constants[16] were calculated with the two-component exact decoupling approach (X2C)[17] including spin-orbit coupling and employing the shielded-nucleus (SNSO) model[18] with x2c-TZVPall-2c basis sets.[19] The conductor-like screening model[20] (dielectric constant of 7.6, corresponding to THF) was used for all NMR calculations. The reaction pathways between optimized minima of monomers and dimers were optimized with a generalized Newton method proposed by Plessow.[21] The resulting maxima were used as starting points for transition state optimizations (with the module statpt). The resulting stationary points are shown in Fig. S47, exemplarily for the pnictogen being As. It was confirmed by frequency calculations that the local minima (1, 3, VI, VIII) have no imaginary modes and that the transition states (2, VII) have exactly one imaginary mode. Cartesian coordinates are collected in the file structures.xyz. Afterwards, pathways between each pair of neighboured stationary points were re-optimized in order to ensure that no additional maxima are present. These finally resulting pathways are also shown in Fig. S78. Corresponding movies are deposited in the files reaction_as-as.gif and reaction_as-s.gif. The stationary points of the corresponding P-P and P-S system formations were determined analogously. Finally, for all stationary points single point calculations were done with dhf-TZVP basis[11] sets and additionally employing the dispersive correction D3-BJ.[22] and/or the conductor-like screening model (dielectric constant of 7.6, corresponding to THF), also in its improved form accounting for polarity of the solvent, dCOSMO-RS[23], by employing the sigma-potential for THF at 25°C, as available in TURBOMOLE. Energies are collected in Table S3.

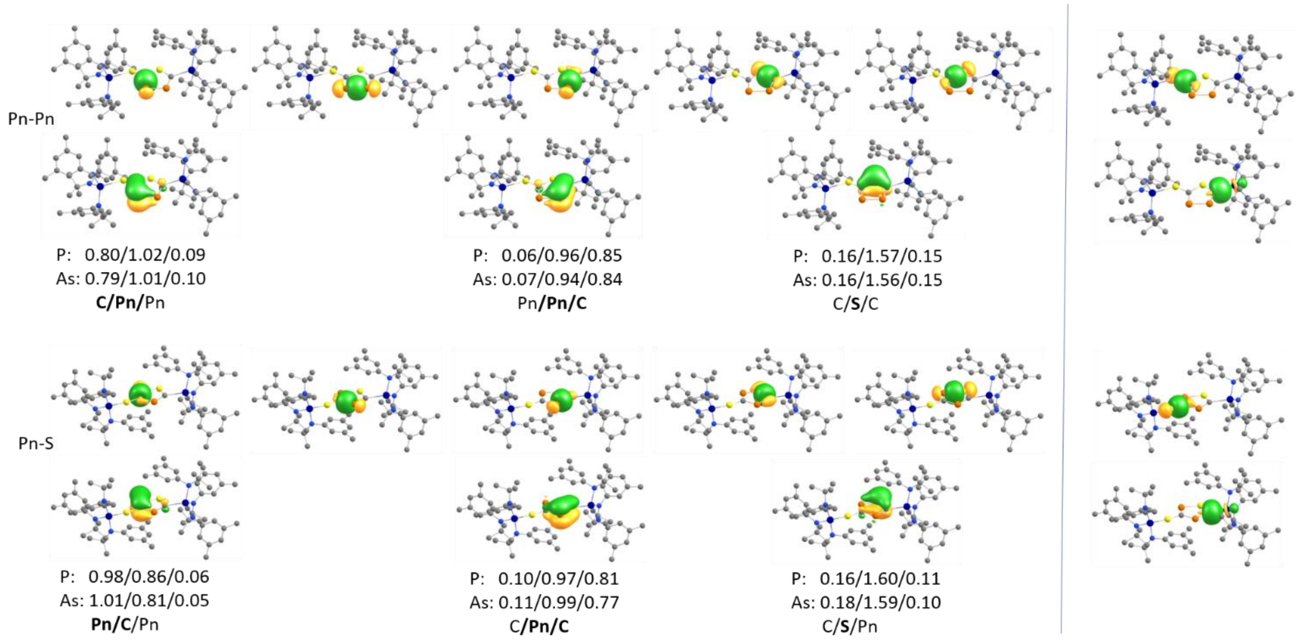

*Figure S 90: Images of calculated localized molecular orbitals (LMOs) (in the 5-membered ring, left of the blue line) for the regioisomers **2-PP** (upper two rows), **2-SP**(lower two rows), and, for the π-type LMOs (second and fourth row) atomic Mulliken contributions for these as well as for their counterparts in **3-SAs** and **3-AsAs**. The LMOs right of the blue line represent the bonds from the ring to the ligands. Hydrogen atoms are omitted.*

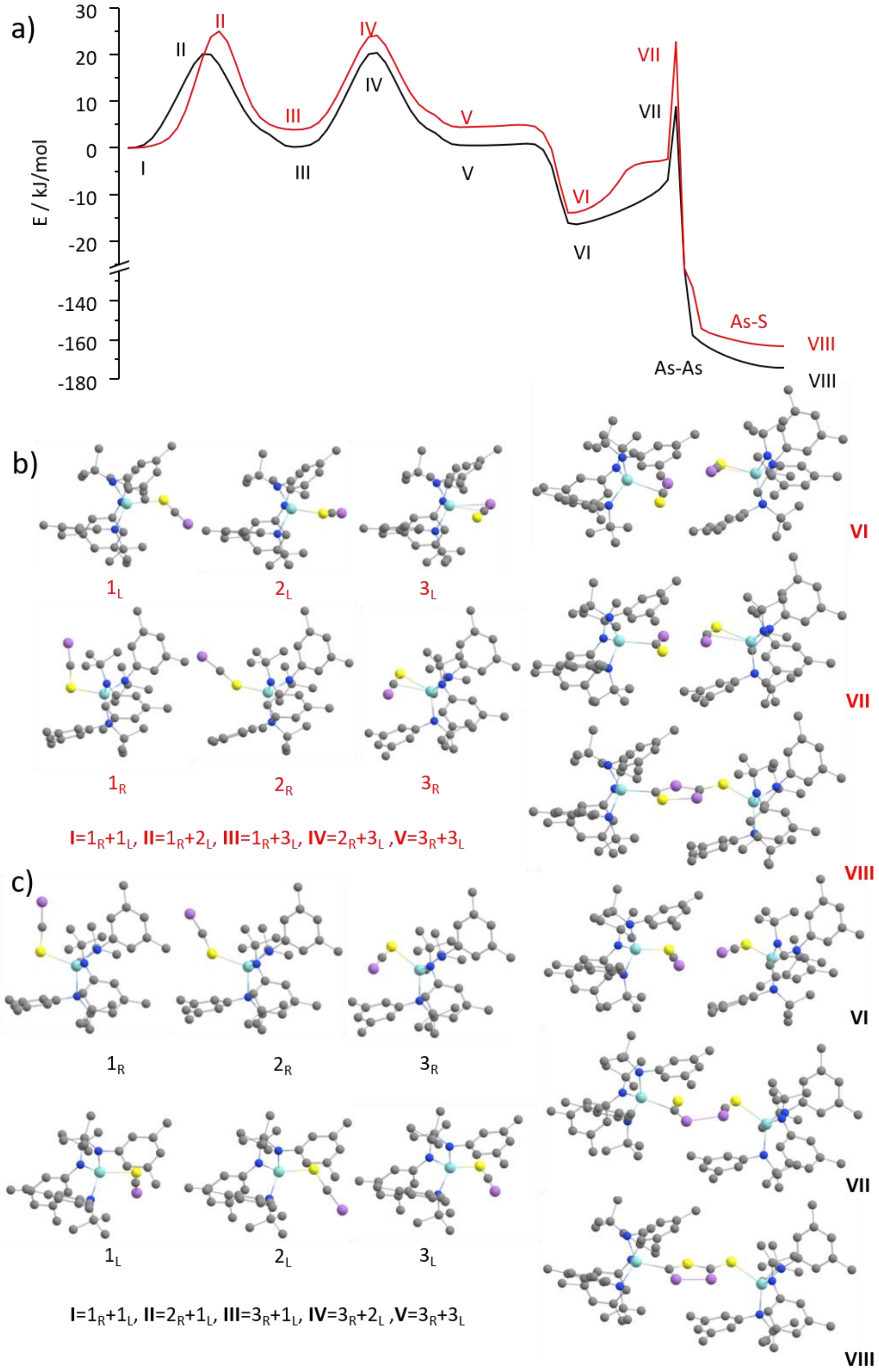

Figure S 91: Energy profile of the reaction pathway to the As-S and the As-As ring compound (a) and images of the stationary points of the path to the As-S (b) and the As-As (c) compound. For I-V only the transforming monomer is shown, i.e. first the left-hand monomer (L), then the right-hand monomer (R) for the As-S compound and the other way for the As-As compound.

Table S 3: Energies in kJ/mol relative to state I for the stationary points along the pathways to the P-P, the P-S, the As-As, and the As-S ring compounds.

**P-P**	I	II	III	IV	V	VI	VII	VIII
SV(P)	0.0	21.1	2.2	23.7	4.4	-9.8	18.1	-142.8
TZVP	0.0	20.2	-0.2	20.4	-0.2	-6.2	24.3	-131.2
COSMO	0.0	17.3	6.2	23.7	12.4	8.7	39.2	-119.3
D3-BJ	0.0	25.2	-13.7	12.1	-27.2	-71.6	-47.2	-197.3
D3-BJ+COSMO	0.0	22.3	-7.4	15.3	-14.7	-56.7	-32.4	-185.4
D3-BJ+COSMO-RS	0.0	21.1	8.1	15.3	-15.3	-35.0	-10.0	-162.0
**P-S**	I	II	III	IV	V	VI	VII	VIII
SV(P)	0.0	26.6	6.3	28.8	8.8	-2.2	37.0	-153.5
TZVP	0.0	24.8	4.3	25.9	4.5	0.9	52.0	-143.4
COSMO	0.0	28.7	10.4	29.2	16.8	14.0	63.6	-132.4
D3-BJ	0.0	16.2	-8.6	18.5	-21.9	-57.2	-16.4	-207.5
D3-BJ+COSMO	0.0	20.1	-2.5	21.8	-9.5	-44.1	-4.8	-196.5
D3-BJ+COSMO-RS	0.0	20.7	-2.7	22.1	-9.8	-22.1	19.7	-174.4
**As-As**	I	II	III	IV	V	VI	VII	VIII
SV(P)	0.0	17.9	0.2	20.3	0.5	-16.5	8.9	-174.1
TZVP	0.0	17.6	-3.0	16.8	-5.8	-16.8	17.4	-164.1
COSMO	0.0	14.8	3.7	20.4	7.6	-0.1	32.2	-150.9
D3-BJ	0.0	22.8	-17.5	7.5	-34.8	-85.6	-55.8	-231.8
D3-BJ+COSMO	0.0	20.0	-10.8	11.1	-21.5	-68.9	-40.9	-218.5
D3-BJ+COSMO-RS	0.0	18.0	-11.2	11.1	-21.7	-47.9	-17.7	-196.0
**As-S**	I	II	III	IV	V	VI	VII	VIII
SV(P)	0.0	25.0	3.9	24.1	4.5	-14.1	22.7	-162.8
TZVP	0.0	22.9	1.9	21.8	-0.7	-12.9	35.0	-150.4
COSMO	0.0	27.1	8.4	25.4	12.5	3.5	48.1	-138.8
D3-BJ	0.0	13.9	-11.9	13.2	-29.0	-74.1	-34.4	-215.6
D3-BJ+COSMO	0.0	18.2	-5.4	16.8	-15.8	-57.7	-21.3	-203.9
D3-BJ+COSMO-RS	0.0	18.3	-6.0	17.3	-15.3	-37.7	4.2	-180.4

References

[1] R. E. H. Kuveke, L. Barwise, Y. van Ingen, K. Vashisth, N. Roberts, S. S. Chitnis, J. L. Dutton, C. D. Martin, R. L. Melen, *ACS Cent. Sci.* **2022**, *8*, 855.
[2] F. Tambornino, A. Hinz, R. Köppe, J. M. Goicoechea, *Angew. Chem. Int. Ed.* **2018**, *57*, 8230.
[3] a) A. Fürstner, C. Mathes, C. W. Lehmann, *Chem. Eur. J.* **2001**, *7*, 5299; b) A. Sinha, R. R. Schrock, P. Müller, A. H. Hoveyda, *Organometallics* **2006**, *25*, 4621; c) Y.-C. Tsai, F. H. Stephens, K. Meyer, A. Mendiratta, M. D. Gheorghiu, C. C. Cummins, *Organometallics* **2003**, *22*, 2902.
[4] a) M. Wedler, F. Knösel, M. Noltemeyer, F. T. Edelmann, U. Behrens, *J. Organomet. Chem.* **1990**, *388*, 21; b) C. Camp, N. Settineri, J. Lefèvre, A. R. Jupp, J. M. Goicoechea, L. Maron, J. Arnold, *Chem. Sci.* **2015**, *6*, 6379; c) N. S. Settineri, M. E. Garner, J. Arnold, *J. Am. Chem. Soc.* **2017**, *139*, 6261; d) T. M. Cook, C. A. Steren, Z.-L. Xue, *Dalton Trans.* **2018**, *47*, 11030.
[5] G. M. Sheldrick, *Acta Cryst A* **2015**, *71*, 3.
[6] O. V. Dolomanov, L. J. Bourhis, R. J. Gildea, J. A. K. Howard, H. Puschmann, *J Appl Crystallogr* **2009**, *42*, 339.
[7] G. M. Sheldrick, *Acta Cryst C* **2015**, *71*, 3.
[8] A. L. Spek, *Acta Cryst C* **2015**, *71*, 9.
[9] V. 7. 2. TURBOMOLE, a development of University of Karlsruhe and Forschungszentrum Karlsruhe GmbH 1989-2007, TURBOMOLE GmbH since 2007, available via https://www.turbomole.org.
[10] J. P. Perdew, K. Burke, M. Ernzerhof, *Phys. Rev. Lett.* **1996**, *77*, 3865.
[11] F. Weigend, A. Baldes, *J. Chem. Phys.* **2010**, *133*, 174102.
[12] O. Treutler, R. Ahlrichs, *J. Chem. Phys.* **1995**, *102*, 346.
[13] J. Pipek, P. G. Mezey, *J. Chem. Phys.* **1989**, *90*, 4916.
[14] R. S. Mulliken, *J. Chem. Phys.* **1955**, *23*, 1833.
[15] Y. J. Franzke, C. Holzer, *J. Chem. Phys.* **2023**, *159*.
[16] Y. J. Franzke, F. Mack, F. Weigend, *J. Chem. Theory Comput.* **2021**, *17*, 3974.
[17] D. Peng, N. Middendorf, F. Weigend, M. Reiher, *J. Chem. Phys.* **2013**, *138*, 184105.
[18] M. Filatov, W. Zou, D. Cremer, *J. Chem. Phys.* **2013**, *139*, 14106.
[19] P. Pollak, F. Weigend, *J. Chem. Theory Comput.* **2017**, *13*, 3696.
[20] A. Pausch, *J. Chem. Theory Comput.* **2024**, *20*, 3169.
[21] P. Plessow, *J. Chem. Theory Comput.* **2013**, *9*, 1305.
[22] S. Grimme, S. Ehrlich, L. Goerigk, *J. Comp. Chem.* **2011**, *32*, 1456.
[23] A. Klamt, M. Diedenhofen, *J. Phys. Chem.* **2015**, *119*, 5439.
